# The interaction mechanism of nickel ions with L929 cells based on integrative analysis of proteomics and metabolomics data

**DOI:** 10.1093/rb/rbac040

**Published:** 2022-06-23

**Authors:** Yajing Zhang, Yan Huang, Rong Chen, Shulin Chen, Xiaoying Lü

**Affiliations:** State Key Laboratory of Bioelectronics, School of Biological Science and Medical Engineering, Southeast University, 2# Si Pailou, Nanjing 210096, China; State Key Laboratory of Bioelectronics, School of Biological Science and Medical Engineering, Southeast University, 2# Si Pailou, Nanjing 210096, China; State Key Laboratory of Bioelectronics, School of Biological Science and Medical Engineering, Southeast University, 2# Si Pailou, Nanjing 210096, China; State Key Laboratory of Bioelectronics, School of Biological Science and Medical Engineering, Southeast University, 2# Si Pailou, Nanjing 210096, China; State Key Laboratory of Bioelectronics, School of Biological Science and Medical Engineering, Southeast University, 2# Si Pailou, Nanjing 210096, China

**Keywords:** nickel ion (Ni^2+^), proteomics, metabolomics, protein–metabolite–metabolic pathway network

## Abstract

The aim of this article was to study the toxicity mechanism of nickel ions (Ni^2+^) on L929 cells by combining proteomics and metabolomics. First, iTRAQ-based proteomics and LC/MS metabolomics analyses were used to determine the protein and metabolite expression profiles in L929 cells after treatment with 100 μM Ni^2+^ for 12, 24 and 48 h. A total of 177, 2191 and 2109 proteins and 40, 60 and 74 metabolites were found to be differentially expressed. Then, the metabolic pathways in which both differentially expressed proteins and metabolites were involved were identified, and three pathways with proteins and metabolites showing upstream and downstream relationships were affected at all three time points. Furthermore, the protein–metabolite–metabolic pathway network was constructed, and two important metabolic pathways involving 4 metabolites and 17 proteins were identified. Finally, the functions of the important screened metabolic pathways, metabolites and proteins were investigated and experimentally verified. Ni^2+^ mainly affected the expression of upstream proteins in the glutathione metabolic pathway and the arginine and proline metabolic pathway, which further regulated the synthesis of downstream metabolites, reduced the antioxidant capacity of cells, increased the level of superoxide anions and the ratio of GSSG to GSH, led to oxidative stress, affected energy metabolism and induced apoptosis.

## Graphical Abstract

**Figure:**
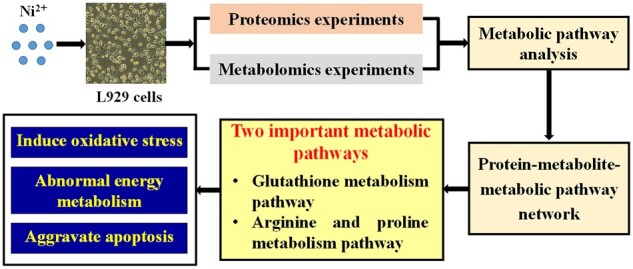

## Introduction

NiTi alloy is a potential biomaterial due to its shape memory effect, good mechanical properties and high elastic modulus. However, when NiTi alloy is applied to the human body, nickel ion (Ni^2+^) dissolution will occur. Nickel is an essential trace element in humans and has many physiological functions, such as stimulating blood production, promoting the regeneration of red blood cells and participating in the synthesis of insulin and various enzymes and proteins [1]. However, Ni^2+^ can lead to allergies, inflammation and even cancer when excess Ni^2+^ is present. Ni^2+^ is a competitive inhibitor of calcium ions (Ca^2+^) that bind to calmodulin to form a complex to inhibit Ca^2+^-ATP activity, causing a continuous increase in the concentration of intracellular Ca^2+^ and leading to an abnormal calcium balance. Ni^2+^ can form complexes with different ligands to affect the function of cells and generate reactive oxygen species (ROS) through the iron ion catalysed by the Fenton reaction, causing oxidative damage to cells, generating DNA damage and disrupting the DNA damage repair system by binding to histones, which induce DNA strand breaks [2–4]. Therefore, the poor biocompatibility of the NiTi alloy is an issue.

At present, most biocompatibility studies on Ni^2+^ have used cytological methods or traditional molecular biology methods, while in-depth and comprehensive studies using high-throughput methods such as genomics, transcriptomics, proteomics and metabolomics are still relatively rare. Our group previously used cDNA microarrays combined with bioinformatics analysis to study the gene expression profiles in L929 cells after treatment with 100 and 200 μM Ni^2+^ for 12, 24, 48 and 72 h, and the cytotoxicity mechanism of Ni^2+^ was determined at the gene level [5, 6].

Genomics technology can elucidate the cytotoxicity mechanism of Ni^2+^ at the global level of genetic variation compared with traditional molecular biology methods. However, proteins and metabolites are the ultimate executors and mediators of physiological activities in organisms, but there is no one-to-one correspondence between genes and proteins/metabolites, so studies at the protein and metabolic levels may provide a deeper understanding of the cytotoxicity mechanism of Ni^2+^. In recent years, novel proteomic and metabolomics technologies are developing rapidly [7–12], which make the detection of proteins and metabolites more sensitive, efficient and selective.

There are few reports on the toxicity mechanism of Ni^2+^ using proteomics or metabolomics methods. Jakob *et al*. [13] revealed that 250 μM Ni^2+^ significantly affected 56 protein species in monocytes after 16 h of treatment using proteomics technology, and the functions of these proteins were related to cell death, metal ion binding and cytoskeletal remodelling. Ni^2+^ was shown to trigger cytoskeleton remodelling and nuclear condensation, inducing monocyte death [13]. Kwon *et al*. [14] found that 69 protein entities were differentially expressed in 20 μM nickel-exposed RKO cells following a 24-h treatment via functional proteomics analysis, and proteins related to apoptosis, cell differentiation and cell proliferation (FASN, HSP90AA1, etc.) were identified through signalling pathway analysis. A metabonomics approach was applied by Tyagi *et al*. [15] to determine the acute biochemical effects of NiCl_2_ on the renal tissues of rats, and increased lactate and the arrest of tricarboxylic acid at the succinate level were detected, resulting in oxidative stress-induced changes in energy metabolism. While the above studies were all carried out using proteomics or metabolomics methods alone, no studies have examined the cytotoxicity mechanism of Ni^2+^ with integrative analysis of proteomics and metabolomics.

The aim of this article is to first study the effects of Ni^2+^ on L929 cells at the protein level and metabolite level and then conduct an integrative analysis of the proteomics and metabolomics results to determine the metabolic pathways in which proteins and metabolites participate together and show upstream and downstream relationships. Functional analysis and experimental verification are further carried out to clarify the internal relationship between protein expression and metabolite synthesis and cytotoxicity and to reveal the mechanism by which Ni^2+^ affects cells at the protein and metabolite levels.

## Materials and methods

### Culture and treatment of L929 cells

L929 cells were purchased from the Shanghai Cell Bank of the Chinese Academy of Sciences. The cells were cultured in RPMI 1640 complete medium (89% RPMI 1640 (Gibco, USA), 10% foetal bovine serum (Sijiqing, China) and 1% (v/v) penicillin–streptomycin (Gibco)) and were incubated in a 37°C and 5% CO_2_ incubator with saturated humidity (Thermo Forma 3111, Thermo Fisher Scientific, USA). The experiments were performed with cells in the logarithmic growth phase. According to our previous results on cytotoxicity and cDNA microarray experiments [6], 100 μM Ni^2+^ was selected for the proteomics experiments, metabolomics experiments and verification experiments in this article. Hexahydrate nickel chloride powders (National Medicine Group Chemical Reagent Co., China) were dissolved in RPMI 1640 complete medium to the final concentration of Ni^2+^ to 100 μM.

### Proteomics experiments

L929 cells were cultured in a cell culture flask with a bottom area of 75 cm^2^. The number of seeded cells was 4 × 10^6^, 3 × 10^6^ or 1.5 × 10^6^ for the experimental groups (cells treated with 100 μM Ni^2+^ for 12, 24 and 48 h) and 10^6^ for the control group (cells were cultured with complete medium for 72 h). After 24 h, the culture medium in the experimental groups was replaced with 100 μM Ni^2+^-containing medium and cultured for another 12, 24 and 48 h.

After culture for a given time, the cells were lysed, the protein was extracted with ultrasound and the contents were quantified using the BCA method. Proteomics experiments were performed [16] by Shanghai Omicsspace Biotech Co., Ltd. (Shanghai, China). The iTRAQ data were searched with Protein Discovery 1.4 software (UniProt_mouse_20150515_77267.fasta). The experiment was carried out in three independent runs.

### Metabolomics experiments

L929 cells were cultured in a cell culture flask with a bottom area of 25 cm^2^ and 10^7^ cells per flask. After treatment with 100 μM Ni^2+^ for 12, 24 and 48 h, the cells were collected, and the total intracellular metabolites were extracted. Metabolomics experiments were performed [17] at Shanghai Sensichip Hightech, China. The experiments were replicated five times. Cells cultured for the corresponding time in complete medium were used as controls.

### Bioinformatics analysis of the results from the proteomics and metabolomics experiments

Metabolic pathway analysis was performed by the online programme MetaboAnalyst (https://www.metaboanalyst.ca/) for differentially expressed proteins and metabolites at various time points. The metabolic pathways involving both the proteins and metabolites were first identified. Then, the pathways including proteins and metabolites with upstream and downstream relationships that were also affected in the Ni^2+^-12 h, Ni^2+^-24 h and Ni^2+^-48 h groups were filtered, and the protein–metabolite–metabolic pathway network was generated with Cytoscape software (version 3.7.2).

### Verification experiments

#### Determination of cellular oxidative stress

Intracellular superoxide radical (O_2_^–^) levels in L929 cells before and after 100 µM Ni^2+^ treatment was detected with a dihydroethidium fluorescence probe (Beyotime Institute of Biotechnology, China) as previously described [18]. L929 cells cultured in medium without Ni^2+^ were used as controls.

#### Measurement of GSSG to GSH ratio

The total glutathione and GSSG contents in L929 cells before and after 100 µM Ni^2+^ treatment were detected according to the instructions of the GSH and GSSG test kits (Beyotime Institute of Biotechnology, China) [19]. The GSH content was calculated according to the following formula, and then the ratio of GSSG to GSH was calculated. L929 cells cultured in medium without Ni^2+^ were used as controls.

GSH = total glutathione − GSSG × 2

#### Measurement of ATP contents

The intracellular ATP contents in the L929 cells and Ni^2+^-treated L929 cells for 12, 24 and 48 h were measured with an ATP Analysis Kit (Beyotime, China) [20]. L929 cells cultured in medium without Ni^2+^ were used as controls.

#### Detection of mitochondrial membrane potential

After the L929 cells were treated with 100 μM Ni^2+^ for 12, 24 and 48 h, they were washed once with PBS, and 100 μl of cell culture medium and 100 μl of JC-1 staining working solution (C2006, Beyotime, China) were added and mixed. After incubation at 37°C for 20 min, the cells were washed twice with JC-1 staining buffer (1×) and then stained with Hoechst 33342 for 30 min. The cells were washed three times with PBS, and the average fluorescence intensity of the JC-1 aggregates (which produce red fluorescence) and monomers (which produce green fluorescence) was analysed with a high-content cell analysis system. L929 cells cultured in medium without Ni^2+^ were used as controls.

### Statistical analysis

All experimental data are expressed as the mean ± standard deviation (SD). Student’s *t*-test was applied unless otherwise noted. *P *<* *0.05 indicates a significant difference, and *P *<* *0.01 indicates a very significant difference. All experiments were repeated at least three times.

## Results and discussion

L929 cells are a kind of fibroblasts isolated from mouse subcutaneous tissue. The advantages of this cell are as follows: simple culture environment requirements, rapid proliferation, easy passage and storage, relatively insensitive, and conducive to the stability of the experimental system. So it is not only recommended as the preferred standard cell lines in cytotoxicity experiments by several biological evaluation standards of medical devices (ISO 10933, EN 30993, GB/T 16886, etc.), but also used in Ni^2+^ cytotoxicity studies by many research groups at home and abroad. Therefore, L929 cells were chosen as experimental cells, which is conducive to the integration of our research results with standards and other researches. In addition, L929 cells were also used in our previous researches [5, 6]. In order to compare with the previous results, L929 cells were also selected in this article. Moreover, our previous studies showed that the effects of Ni^2+^ with different concentrations on L929 cells were significantly different at the cellular and molecular levels [5, 6]. This article focuses on the toxicity mechanism of 100 μM Ni^2+^ to L929 cells.

### Results of the proteomics experiments

**Figure 1.**
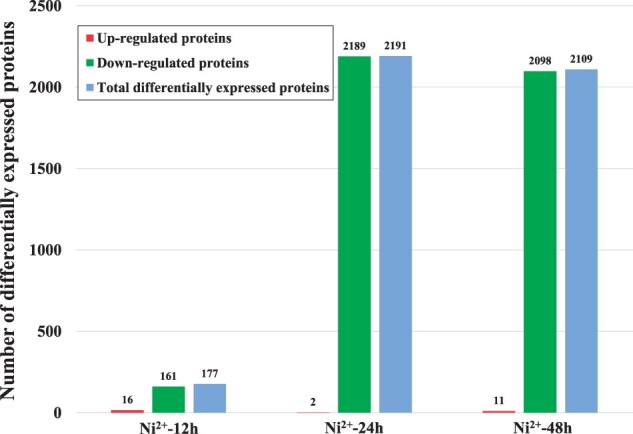
Number of differentially expressed proteins in the L929 cells treated with 100 μM Ni^2+^ for 12, 24 and 48 h.

Compared with those of the control group, 177, 2191 and 2109 proteins were differentially expressed in L929 cells after treatment with 100 μM Ni^2+^ for 12, 24 and 48 h, respectively (Fig. 1, Supplementary Tables S1–S3). The differentially expressed proteins mainly showed downregulated expression in all three Ni^2+^-treated groups, and the order of the number of differentially expressed proteins was: Ni^2+^-24 h group > Ni^2+^-48 h group > Ni^2+^-12 h group. It was found that the number of differentially expressed proteins did not increase linearly with time. The possible reasons were as follows: when L929 cells were treated with 100 μM Ni^2+^ for 12 h, the effect of Ni^2+^ on the cells was just produced due to the short action time, so there were fewer differentially expressed proteins. When the action time reached 24 h, the stress response of cells to the ‘foreign matter’ Ni^2+^ started, so the number of differentially expressed proteins increased significantly. However, when the action time was 48 h, the toxic effects of Ni^2+^ increased gradually (shown as the cell proliferation rate continued to decline), and the stress response weakened, so the number of differentially expressed proteins decreased. This phenomenon was also observed in the data of differentially expressed genes induced by Ni^2+^ in our previous study [6]. Such a phenomenon was specifically reported in the literature, which was called gene oscillation [21].

### Results of the metabolomics experiments

**Figure 2.**
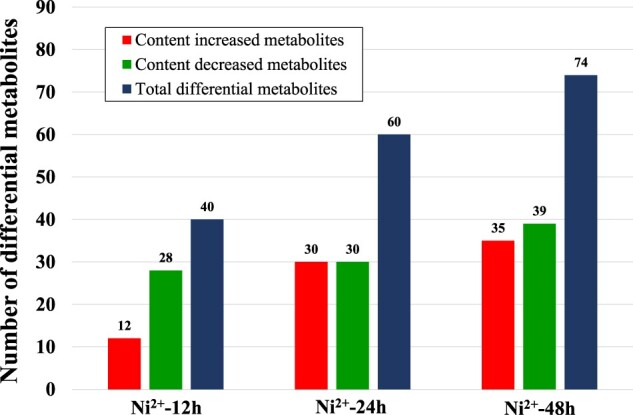
Number of differentially abundant metabolites in the L929 cells treated with 100 μM Ni^2+^ for 12, 24 and 48 h.

Compared with those in the control groups, 40, 60 and 74 types of differentially abundant metabolites were detected in L929 cells after treatment with 100 μM Ni^2+^ for 12, 24 and 48 h, respectively (Fig. 2, and the details are shown in Supplementary Tables S4–S6). The number of differentially abundant metabolites increased gradually with the prolongation of Ni^2+^ treatment, indicating that the effect of Ni^2+^ on the metabolic activity in L929 cells was gradually enhanced.

### Bioinformatics analysis of the proteomics and metabolomics results

#### Screening of metabolic pathway proteins and metabolites

There were 18, 30 and 33 metabolic pathways involving both proteins and metabolites in the Ni^2+^-12 h, Ni^2+^-24 h and Ni^2+^-48 h groups, respectively (Supplementary Tables S7–S9); among them, there were 8, 23 and 27 pathways in which the proteins and metabolites had upstream and downstream relationships (Table 1, Supplementary Tables S10–12). Three pathways (glutathione metabolism, arginine and proline metabolism, and glycerophospholipid metabolism) were common to all three time points, 18 pathways were common to two time points, and 13 pathways appeared at only one time point.

**Table 1.** Metabolic pathways in which proteins and metabolites had upstream and downstream relationships in the Ni^2+^-12 h, Ni^2+^-24 h and Ni^2+^-48 h groups

Type	Pathways	Ni^2+^-12 h	Ni^2+^-24 h	Ni^2+^-48 h
Pathways common to all three time points	1) Glutathione metabolism	√	√	√
	2) Arginine and proline metabolism	√	√	√
	3) Glycerophospholipid metabolism	√	√	√
Pathways common to two time points	1) Cysteine and methionine metabolism	√		√
	2) Alanine, aspartate and glutamate metabolism		√	√
	3) Arginine biosynthesis		√	√
	4) Citrate cycle (TCA cycle)		√	√
	5) D-glutamine and D-glutamate metabolism		√	√
	6) Fructose and mannose metabolism		√	√
	7) Galactose metabolism		√	√
	8) Glycerolipid metabolism		√	√
	9) Glyoxylate and dicarboxylate metabolism		√	√
	10) Histidine metabolism		√	√
	11) Nitrogen metabolism		√	√
	12) Pantothenate and CoA biosynthesis		√	√
	13) Phenylalanine metabolism		√	√
	14) Phenylalanine, tyrosine and tryptophan biosynthesis		√	√
	15) Pyrimidine metabolism		√	√
	16) Tyrosine metabolism		√	√
	17) Valine, leucine and isoleucine biosynthesis		√	√
	18) Valine, leucine and isoleucine degradation		√	√
Pathways appear at one time point	1) Glycine, serine and threonine metabolism	√		
	2) Inositol phosphate metabolism	√		
	3) One carbon pool by folate	√		
	4) Phosphatidylinositol signalling system	√		
	5) Beta-alanine metabolism		√	
	6) Purine metabolism		√	
	7) Sphingolipid metabolism		√	
	8) Biosynthesis of unsaturated fatty acids			√
	9) Primary bile acid biosynthesis			√
	10) Butanoate metabolism			√
	11) Synthesis and degradation of ketone bodies			√
	12) Pentose phosphate pathway			√
	13) Propanoate metabolism			√

Table 2 shows the three pathways common to all three time points and the included proteins and metabolites with upstream and downstream relationships, involving a total of 33 proteins and 9 metabolites. Among them, the glutathione metabolic pathway and the arginine and proline metabolic pathway involved 24 proteins and 5 metabolites (glutathione, spermidine, L-glutamate, pyroglutamic acid and L-proline). The glycerophospholipid metabolic pathway involved nine proteins and four metabolites [acetylcholine chloride, PE(36:6), LysoPC(22:5) and PC(28:0)], and the relationship between these four metabolites and cytotoxicity has not previously been reported in the literature. Therefore, this finding is reported for the first time in our study, and further research is needed in the future.

**Table 2.** Metabolic pathways in which proteins and metabolites had upstream and downstream relationships common to all three Ni^2+^-treated groups

No.	Pathway	Ni^2+^-12h		Ni^2+^-24h		Ni^2+^-48h	
		Protein	Metabolite	Protein	Metabolite	Protein	Metabolite
1	Glutathione metabolism pathway	**Lap3↓**	**Glutathione↓**	**20** (Gsr↓, Gsta4↓, Gstm1↓, Gstm2↓, Gstm7↓, Gsto1↓, Gstp1↓, Gstt3↓, Gpx1↓, Gpx4↓, Gpx7↓, Gpx8↓, Gclc↓, Gclm↓, Idh1↓, Idh2↓, G6pdx↓, Lap3↓, Pgd↓, Txndc12↓)	L-glutamate↓	**14 (Gsr↓, Gstm1↓, Gstm2↓, Gsto1↓, Gstp1↓, Gpx4↓, Gpx7↓, Gclm↓, Idh1↓, Idh2↓, G6pdx↓, Lap3↓, Pgd↓, Txndc12↓)**	**L-glutamate↑**
		Srm↓	Spermidine↓			**10 (Gsr↓, Gpx4↓, Gpx7↓, Gclm↓, Idh1↓, Idh2↓, G6pdx↓, Lap3↓, Pgd↓, Txndc12↓)**	**Pyroglutamic acid↑**
2	Arginine and proline metabolism pathway	Srm↓	Spermidine↓	4 (Aldh18a1↓, Lap3↓, Pycrl↓, Pycr2↓)	L-glutamate↓	**4 (Aldh18a1↓, Lap3↓, Pycrl↓, Pycr2↓)**	**L-glutamate↑**
						**3 (Lap3↓, Pycrl↓, Pycr2↓)**	**L-proline↓**
3	Glycerophospholipid metabolism pathway	Lpin3↓	Acetylcholine chloride↓	8 (Agpat3↓, Gpd1↓, Gpd1l↓, Gpd2↓, Lpcat3↓, Lpin3↓, Pcyt1a↓, Pisd↓)	PE(36:6) ↑	8 (Agpat3↓, Gpd1↓, Gpd1l↓, Gpd2↓, Lpcat3↓, Lpin3↓, Pcyt2↓, Pisd↓)	PE(36:6) ↓ PC(28:0) ↓
				7 (Gpd1↓, Gpd2↓, Gpd1l↓, Lpcat3↓, Lpin3↓, Pcyt1a↓, Pisd↓)	LysoPC(22:5) ↓		

The following studies mainly analysed the glutathione metabolic pathway, arginine and proline metabolic pathway and the functions of the proteins and metabolites participating in these two pathways.

#### Construction of the protein–metabolite–metabolic pathway network

**Figure 3.**
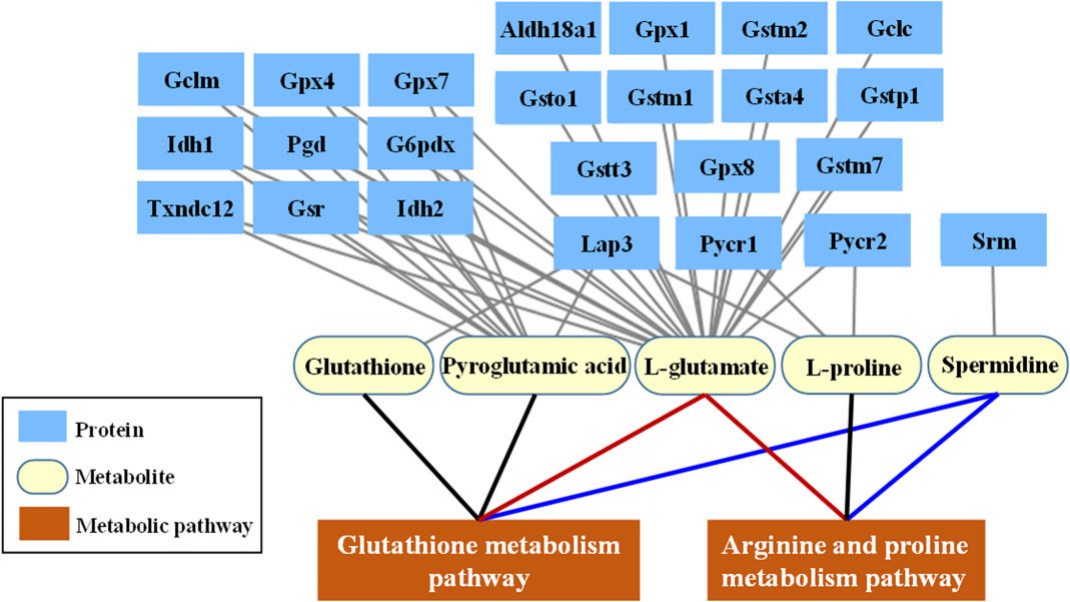
The protein–metabolite–metabolic pathway in the interaction between Ni^2+^ and L929 cells included 24 upstream proteins, 5 downstream metabolites and 2 metabolic pathways.

Figure 3 shows the protein–metabolite–metabolite pathway network constructed according to the proteins and metabolites involved in the glutathione metabolic pathway and the arginine and proline metabolic pathway, as shown in Table 2, including 24 proteins and 5 metabolites. L-glutamate and spermidine are involved in both pathways, thus linking the two pathways.

##### Glutathione metabolic pathway

**Figure 4.**
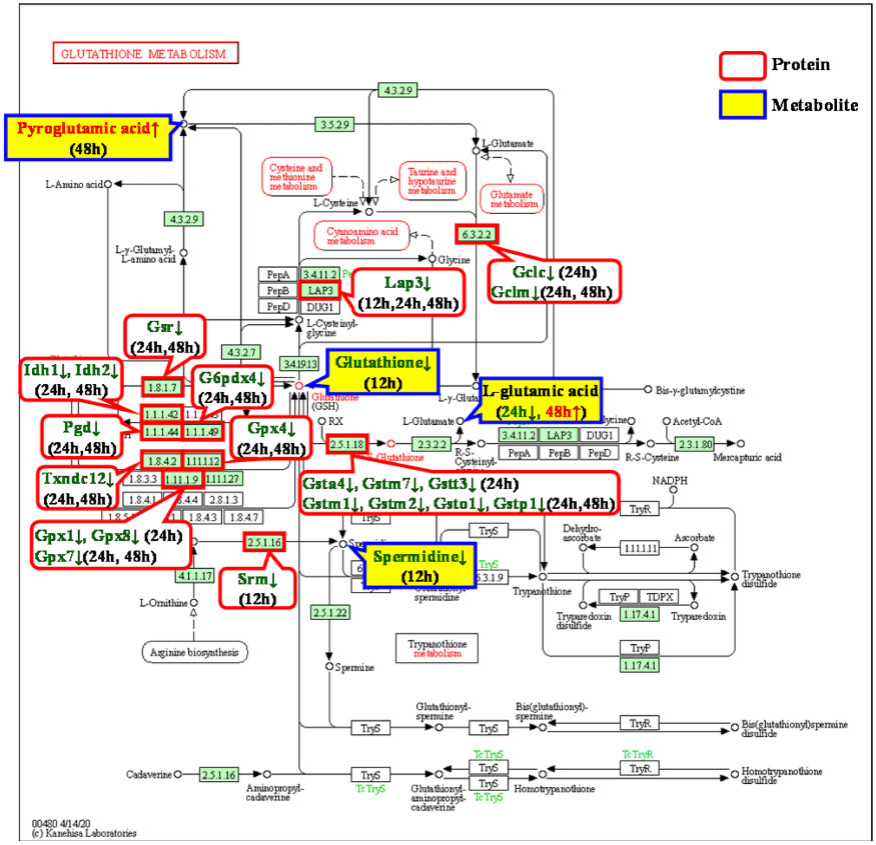
Glutathione metabolic pathway [22] and the included differentially expressed proteins and metabolites with upstream and downstream relationships in the Ni^2+^-treated groups.

Figure 4 shows the glutathione metabolic pathway. This pathway is mainly the process of glutamate synthesis from glutathione. There were four metabolites involved in the pathway: glutathione, pyroglutamic acid, L-glutamate and spermidine.

The first metabolite, glutathione, is the most widespread antioxidant in cells. This metabolite is a tripeptide comprised of glutamic acid, cysteine and glycine. This molecule not only effectively resists the damage to cells caused by free radicals, peroxides, lipid peroxides and heavy metals [23] but also plays a role in metabolic processes and biochemical reactions such as DNA synthesis and repair and the regulation of enzyme activity. Glutathione includes reduced (GSH) and oxidized (GSSG) forms, which can donate electrons through reduced coenzyme II (NADPH) and can be converted to GSH by glutathione reductase (Gsr) [24]. GSSG is often used as a marker for the degree of cytotoxic damage due to its effects on oxygen free radicals [25]. In this article, the glutathione content was found to be decreased in the Ni^2+^-12 h group, and glutathione was extremely important for the removal of ROS produced in cells. Therefore, Ni^2+^ affected the process of intracellular oxidative stress and decreased the cellular antioxidant capacity. Oxidative stress further affected mitochondrial energy metabolism, resulting in a decrease in TCA cycle metabolites [15].

The second metabolite involved in this pathway is pyroglutamic acid. This molecule is a product of the cyclization of free amino groups of glutamic acid or glutamate and can be converted to glutamate by hydroxyprolinase [26], while glutamate is a key metabolite in a variety of intracellular metabolic processes. Pyroglutamic acid plays a vital role in defending against the intracellular oxidative damage caused by ROS and can be induced if the external stress is not too severe [27]. The pyroglutamic acid content was increased in the Ni^2+^-48 h group, indicating that L929 cells generate increased levels of pyroglutamic acid to oppose the oxidative damage induced by Ni^2+^.

The third metabolite involved in this pathway was L-glutamate, which also participates in the arginine and proline metabolic pathway. As one of the basic amino acids, glutamate is involved in many crucial chemical reactions in the body and plays an important role in protein metabolism in organisms [28]. Glutamate was shown to effectively promote the proliferation of hair papilla cells, while glutamate at high concentrations caused calcium overload that interfered with mitochondrial respiratory chain function, thereby inhibiting cystine uptake and causing GSH deprivation and ROS accumulation, ultimately leading to cell necrosis or apoptosis [29, 30]. The glutamate content decreased in the Ni^2+^-24 h group but increased in the 48 h group, indicating that glutamate might not have adverse effects on cells at low concentrations but might cause mitochondrial dysfunction, affecting ATP synthesis, causing oxidative damage and eventually leading to cell apoptosis or death at high concentrations.

The fourth metabolite involved in this pathway was spermidine, which also participates in the arginine and proline metabolic pathway. Spermidine is a polyamine widely present in living tissues and ribosomes, and polyamines are essential for cell growth and viability [31]. Excessive accumulation of spermidine (4 mM) could inhibit cell growth and significantly reduce cell viability [31], while low concentrations of spermidine (<2 μM) were not found to be cytotoxic [32]. The spermidine content in the Ni^2+^-12 h group was lower than that in the control group, which was consistent with our previous results indicating that 100 μM Ni^2+^ did not result in cytotoxicity of L929 cells after 12 h [6]. This result indicated that the low content of spermidine did not play a role in Ni^2+^-induced cytotoxicity.

##### Arginine and proline metabolic pathway

**Figure 5.**
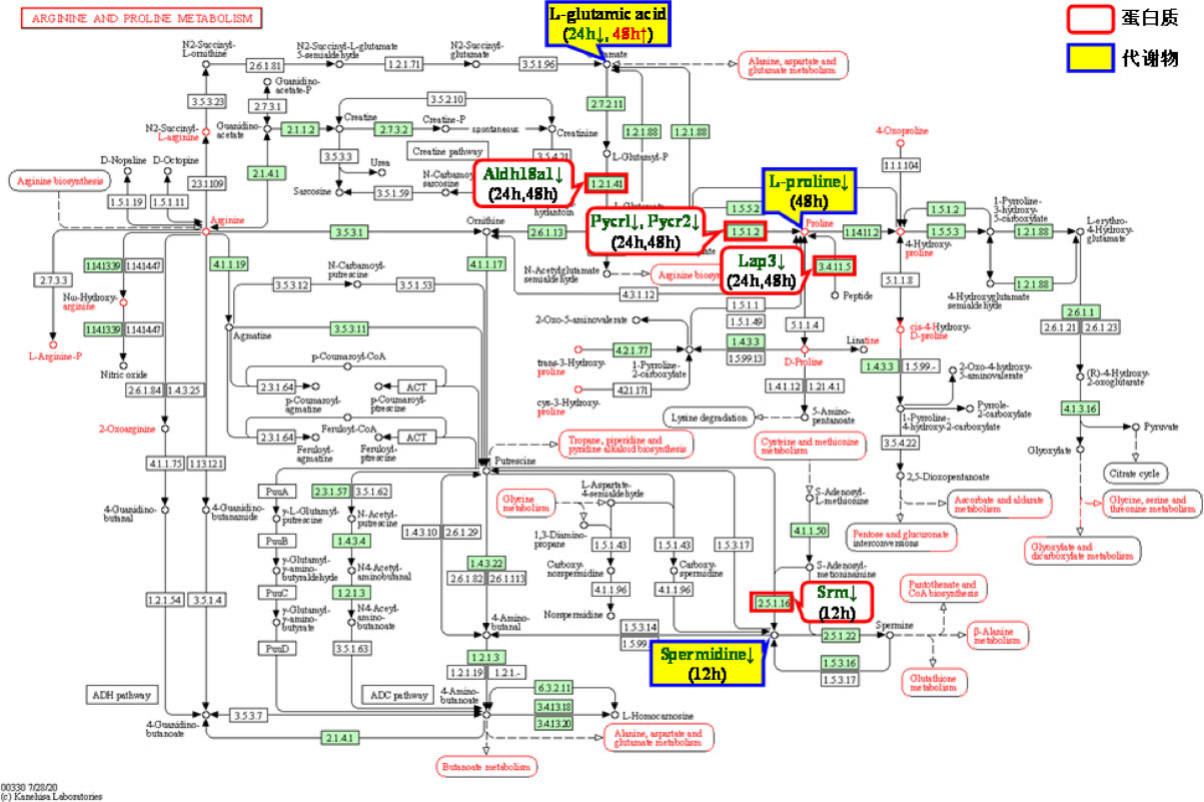
Arginine and proline metabolic pathway [22] and the differentially expressed proteins and metabolites with upstream and downstream relationships in the Ni^2+^-treated groups.

Figure 5 shows the arginine and proline metabolic pathway. This pathway mainly involves the conversion of arginine to proline. Arginine is finally converted into putrescine, proline and glutamine under the action of related enzymes. Putrescine can generate spermidine and spermine, and glutamine can enter the TCA cycle and is oxidized to supply energy to generate CO_2_.

We identified three metabolites involved in the arginine and proline metabolic pathway. In addition to L-glutamate and spermidine, L-proline was also identified. Proline is an alpha amino acid during protein synthesis that can be oxidized to glutamate by two mitochondrial enzymes, proline dehydrogenase and Δ^1^-pyrroline-5-carboxylate dehydrogenase. Proline is a widespread antioxidant that effectively attacks ROS, which are frequently elevated in stressful environments and suppress apoptosis [33]. The decrease in the L-proline content in the Ni^2+^-48 h group indicated that the antioxidant capacity of L929 cells decreased at this time, and apoptosis might have occurred.

From the above analysis, the decreased glutathione and L-proline levels and the increased pyroglutamic acid and glutamate levels were shown to play an important role in Ni^2+^-induced cytotoxicity by affecting the process of intracellular oxidative stress, causing mitochondrial dysfunction and oxidative damage. Therefore, only the proteins that regulated the synthesis of the above-mentioned four metabolites are discussed below. Table 2 shows that 17 proteins were involved (bold and underlined). A literature survey of these 17 proteins showed that 7 of them (Aldh18a1, G6pdx, Lap3, Pgd, Pycrl, Pycr2 and Txndc12) had not been previously reported to be related to cytotoxicity. These proteins were also our new findings and require further research in the future. The remaining 10 proteins are listed in Table 3 and are discussed below.

**Table 3.** Classification of 10 upstream differentially expressed proteins

Type	Protein
Glutathione reductase	1 (Gsr)
Glutathione S-transferases	4 (Gstm1, Gstm2, Gsto1, Gstp1)
Glutathione peroxidase	2 (Gpx4, Gpx7)
Glutamate-cysteine ligase	1 (Gclm)
Isocitrate dehydrogenase	2 (Idh1, Idh2)

Glutathione reductase (Gsr) catalyses the regeneration of GSH from GSSG, utilizing NADPH generated by the hexose monophosphate shunt. Therefore, by catalysing the GSH/GSSG cycle to facilitate electron transfer from glucose to H_2_O_2_, Gsr can prevent the accumulation of excessive amounts of ROS and the resulting oxidative damage. Therefore, Gsr is a major cellular antioxidant [34]. The downregulation of Gsr expression in the Ni^2+^-24 h and Ni^2+^-48 h groups indicated that Ni^2+^ might lead to an increase in ROS and a weakening of the cellular antioxidant capacity.

Gstm1, Gstm2, Gsto1 and Gstp1 are all glutathione S-transferases (GSTs). GSTs are key enzymes in the glutathione-binding reaction and catalyse the conjugation of GSH to hydroxyalkenals [35]. These enzymes mainly regulate the oxidative stress state of cells and inhibit cell apoptosis. Gstm1 can metabolize a broad range of ROS and xenobiotics, and a decreased expression of Gstm1 may result in excess oxidative stress [36], while silencing GSTM2 gene expression results in an increase in cell death [37]. Gsto1 is involved in glutathionylation, Toll-like receptor signalling and calcium channel regulation. This molecule has been implicated in the regulation of oxidative stress, and knockdown of Gsto1 was shown to increase ROS levels [38]. Gstp1 is the most prevalent GST in mammalian cells. This molecule is a determinant in the cellular response to oxidative stress and protects cells from apoptosis elicited by a variety of cytotoxic agents, such as H_2_O_2_, UV and arsenic trioxide [39]. Upregulated Gstp1 expression inhibited the phosphorylation of MAPKs, including ERK, JNK and p38, and the activation of NF-κB resulted in a decrease in tumour necrosis factor alpha and nitric oxide synthesis, thus protecting cells [40]. The downregulated expression of all of the above four GSTs in the Ni^2+^-24 h and Ni^2+^-48 h groups indicated that the antioxidant capacity of the cells was weakened and the degree of oxidative stress increased after treatment with Ni^2+^.

Both Gpx4 and Gpx7 are glutathione peroxidases. Glutathione peroxidase is an important peroxide-decomposing enzyme that can convert GSH to GSSG, reduce toxic peroxides to nontoxic hydroxyl compounds and promote the decomposition of H_2_O_2_, thereby protecting the structure and function of cell membranes from peroxides. Gpx4 plays a role in apoptosis induced by oxidative stress that most likely occurs through oxidative damage to mitochondrial phospholipids [41]. Overexpression of Gpx7 could inhibit the production of ROS [42]. The downregulation of Gpx4 and Gpx7 expression in the Ni^2+^-24 h and Ni^2+^-48 h groups also suggested a decreased antioxidant capacity and increased oxidative stress after Ni^2+^ treatment.

Gclm is a kind of glutamate-L-cysteine ligase (GCL). GCL catalyses the rate-limiting step of glutathione [L-g-glutamyl-cysteinyl-glycine (GSH)] synthesis and is a key cellular antioxidant. GCL comprises the catalytically active heavy subunit (GCLC), which contains all of the substrate-binding sites, and a regulatory subunit (GCLM) that modulates the affinity of GCL for glutamate. Knockout of Gclm could significantly decrease the GSH content and increase the oxidative stress levels [43].

Idh1 and Idh2 are isocitrate dehydrogenases (IDHs). IDH is the rate-limiting enzyme in the TCA cycle, which is involved in cell energy metabolism, and has five subtypes. All IDH enzymes catalyse the oxidative decarboxylation of isocitrate to α-ketoglutarate. Idh1 is located in the cytosol and peroxisomes and protects cells from oxidative stress by regulating the intracellular NADP(+)/NADPH ratio [44]. Idh2 is located in the mitochondria and is a critical antioxidant enzyme in the regulation of redox status and the reduction of oxidative stress-induced damage [45]. Both Idh1 and Idh2 use NADP+ as their coenzyme and produce one molecule of NADPH by converting isocitrate to α-ketoglutarate [44]. NADPH is a substrate for many metabolic redox reactions, and a sufficient supply of its reducing equivalents is required for the detoxification of the ROS that routinely accumulate in cells. The downregulation of Idh1 and Idh2 expression in the Ni^2+^-24 h and Ni^2+^-48 h groups led to a decrease in NADPH and the amount of decomposable ROS, resulting in increased ROS levels, activation of NF-κB and apoptosis [45].

Since the upstream protein Srm and its regulated downstream metabolite spermidine did not play a significant role in Ni^2+^-induced cytotoxicity, Ni^2+^ might regulate the synthesis of four downstream metabolites (glutathione, pyroglutamic acid, L-glutamate, L-proline) by affecting the differential expression of 17 upstream proteins, which in turn reduced the antioxidant capacity of cells, increased the level of oxidative stress and decreased the mitochondrial function (decreased ATP content and mitochondrial membrane potential), eventually inducing apoptosis. Therefore, in this article, the results of the combined bioinformatics analysis were experimentally verified through the determination of cellular oxidative stress, the ratio of GSSG to GSH, the ATP content and the cellular mitochondrial membrane potential.

### Verification experiments

#### Determination of cellular oxidative stress

Oxidative stress refers to the tissue damage caused by excessive production of highly active molecules such as ROS and reactive nitrogen species when the body is subjected to various harmful stimuli and the degree of oxidation exceeds the removal of oxides, resulting in an imbalance between the oxidative system and the antioxidant system. Therefore, detecting the content of ROS (O_2_^–^, H_2_O_2_, etc.) can reflect the level of oxidative stress [46].

**Figure 6.**
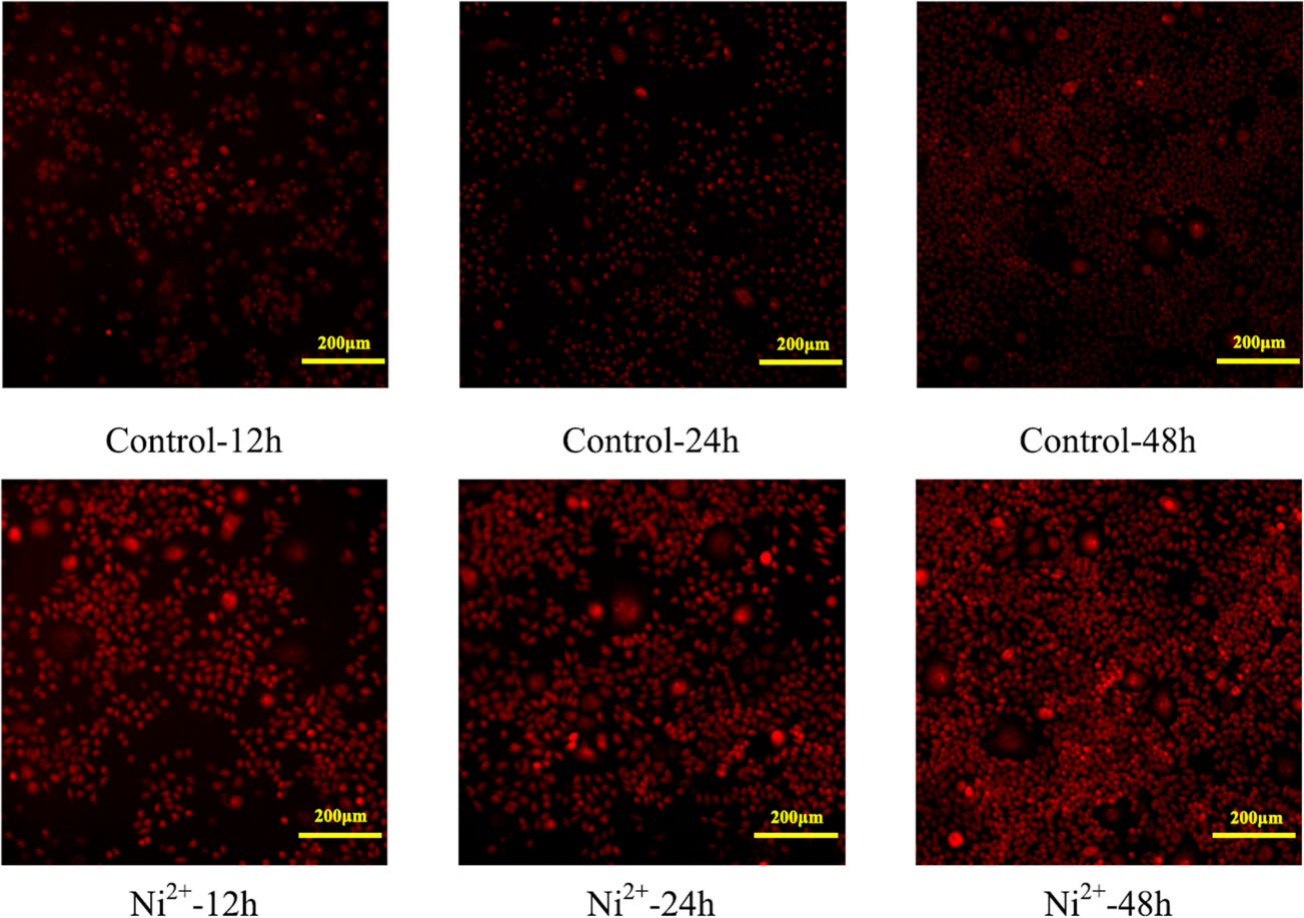
Images of $O_{2}^{–}$ stained L929 cells in the control group and the 100 μM Ni^2+^-treated groups.

**Figure 7.**
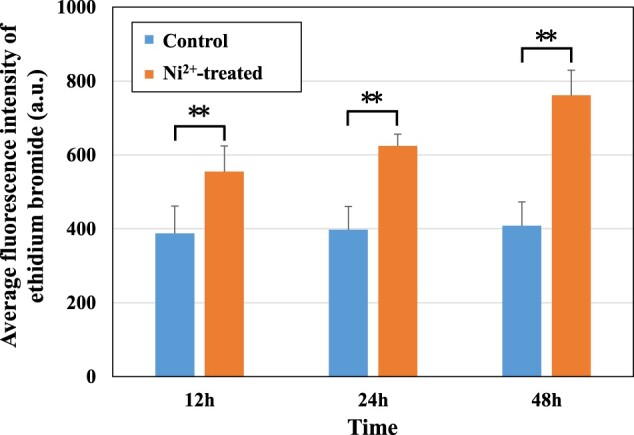
Average fluorescence intensity of ethidium bromide in the L929 cells in the control groups and the 100 μM Ni^2+^-treated groups. **P <* *0.01 indicates a very significant difference from the control group.

Figures 6 and 7 show the images of O_2_^–^ staining and the calculated O_2_^–^ levels in the control and Ni^2+^-treated L929 cells for 12, 24 and 48 h. The average fluorescence intensity for ethidium bromide represents the O_2_^–^ level. Figure 7 shows that the fluorescence intensity of ethidium bromide in Ni^2+^-treated groups increased with increasing time, and the fluorescence intensity in the Ni^2+^-treated groups was significantly higher than that in the control groups at all three time points. This finding indicated that Ni^2+^ could increase the level of oxidative stress in L929 cells.

#### Measurement of GSSG to GSH ratio

As GSSG is often used as a marker for the degree of cytotoxic oxidative damage [25], while GSH reflects the antioxidant levels of cells, so the ratio of GSSG to GSH (R_GSSG/GSH_) can be used to characterize the level of oxidative damage. The higher the R_GSSG/GSH_, the greater the degree of oxidative damage.

**Figure 8.**
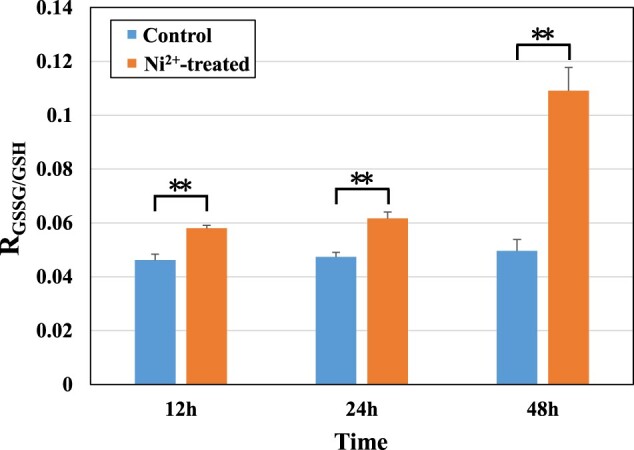
Ratio of GSSG to GSH in the L929 cells in the control groups and the 100 μM Ni^2+^-treated groups. **P <* *0.01 indicates a very significant difference from the control group.

Figure 8 show the R_GSSG/GSH_ in the control and Ni^2+^-treated L929 cells for 12, 24 and 48 h. It could be noticed that there was no significant change in R_GSSG/GSH_ in three control groups, but R_GSSG/GSH_ increased in Ni^2+^-treated group with increasing time, and all of them were very significantly higher than that in the control groups at all three time points. These findings indicated that Ni^2+^ could induce oxidative damage.

#### Measurement of ATP content

**Figure 9.**
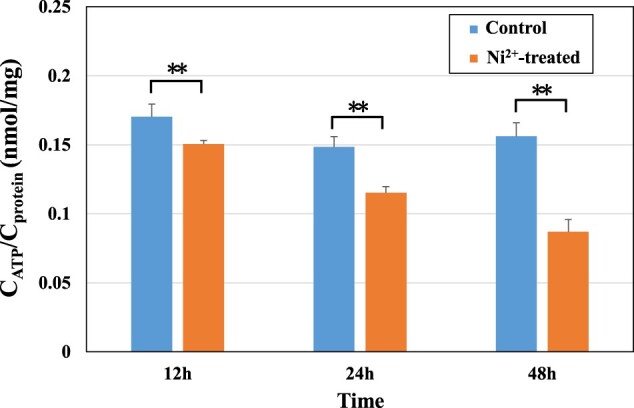
The ATP content in the L929 cells in the control groups and 100 μM Ni^2+^-treated groups. **P <* *0.01 indicates a very significant difference from the control group.

Mitochondria are the main sites of ATP synthesis, and damage to mitochondrial function leads to a decrease in ATP synthesis. The intracellular ATP contents in the control and 100 μM Ni^2+^-treated groups were measured and are shown in Fig. 9. The ATP content in all Ni^2+^-treated groups was significantly lower than that in the control group (*P *<* *0.01), which indicated that Ni^2+^ could indeed affect the process of intracellular energy metabolism and reduce the ATP content in cells. These results were consistent with previous reports that Ni^2+^ could reduce the activities of succinate dehydrogenase and nicotinamide adenine dinucleotide [47] and affected the process of intracellular energy metabolism by altering the oxidation of fatty acids [48].

#### Detection of the mitochondrial membrane potential

Apoptosis is an active cell death process regulated by genes. The hallmarks of apoptosis include cell membrane foaming, cell shrinkage, decreased mitochondrial membrane potential (MMP), chromosome condensation and DNA fragmentation. MMP has become an indicator to evaluate early apoptosis of cells [49].

**Figure 10.**
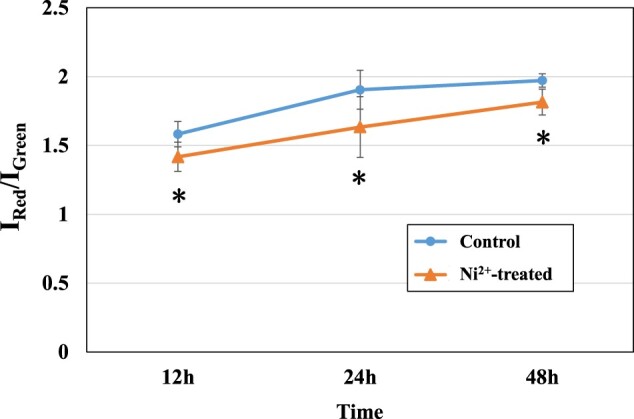
Results of the MMP of the L929 cells in the control groups and 100 μM Ni^2+^-treated groups. *P <* *0.05 indicates a significant difference from the control group. Red fluorescence indicates cells with a normal MMP, and green fluorescence indicates cells with a reduced MMP.

The MMP results in the control and 100 μM Ni^2+^-treated groups are shown in Fig. 10. Red fluorescence indicates cells with a normal MMP, and green fluorescence indicates cells with a reduced MMP. In this article, the ratio of red fluorescence intensity to green fluorescence intensity (I_Red_/I_Green_) was used to reflect the level of MMP. The lower the ratio is, the lower the MMP is. We found that all I_Red_/I_Green_ in Ni^2+^-treated groups had significantly lower values than the control groups at the corresponding time, which indicated that the MMP decreased and apoptosis increased.

In our previous study, human umbilical vein endothelial cells were selected as the research object for the cardiac occluder made of NiTi alloy, and the effects of bare and TiN-coated NiTi alloys with different Ni^2+^ dissolution characteristics on endothelial cell adhesion, growth and proliferation were compared. It was found that in stage of cell growth and proliferation, the release of Ni^2+^ from bare NiTi alloy increased with time and reached a higher level, which inhibited endothelial cell function by affecting pathways associated with actin cytoskeleton, focal adhesion, energy metabolism, inflammation and amino acid metabolism. In comparison, TiN coating not only effectively prevented release of Ni ions from NiTi alloy, but also improved endothelial cell function by promoting actin cytoskeleton and focal adhesion formation, increasing energy metabolism, enhancing regulation of inflammation and promoting amino acid metabolism [50]. Kasprzak *et al*. [51] gave an overview of possible effects of nickel compounds after entering cells obtained by traditional molecular biology methods. It was found that Ni^2+^ might catalyse ROS generation, induce oxidative stress effects, cause changes in intracellular Ca^2+^ balance, inhibit the activity of DNA damage repair enzymes and change DNA structure. The cells involved include 3T3 cells, CHO cells, HeLa cells, A549 cells, etc. Comparing the results of this article with the above research results, it was found that Ni^2+^ had similar effects on different cells, such as inducing oxidative stress and affecting energy metabolism.

## Conclusions

**Figure 11.**
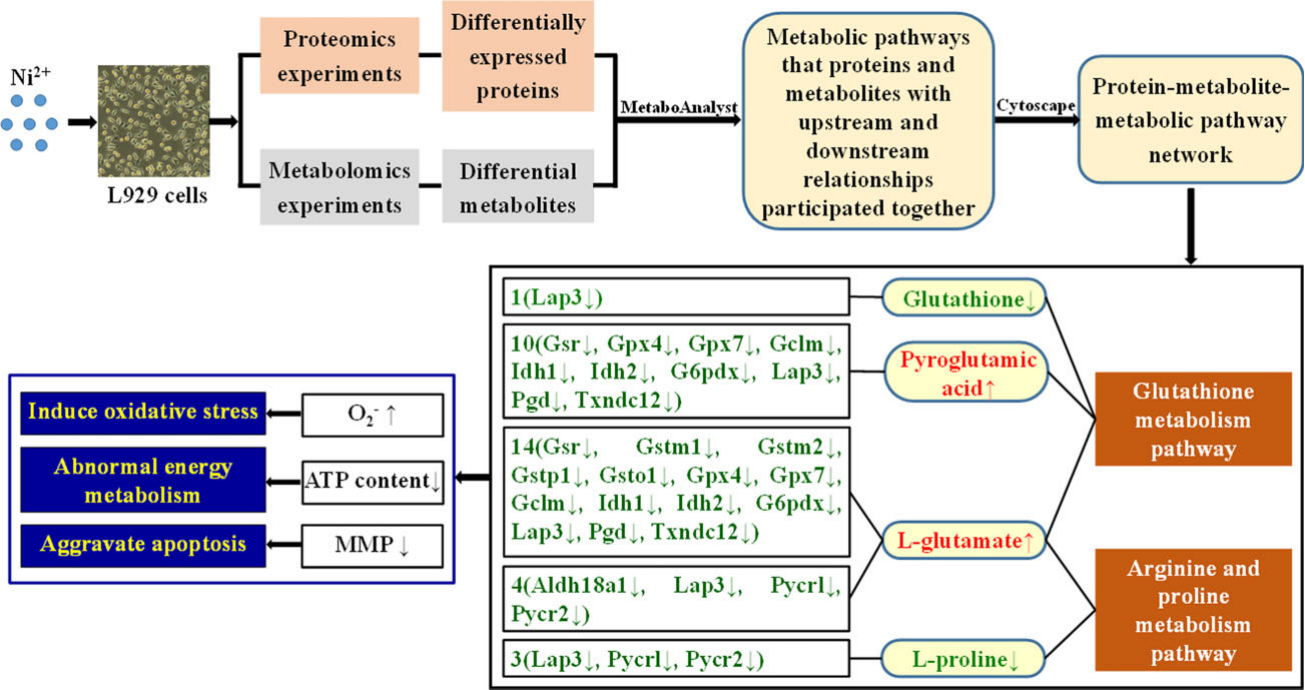
Mechanism of the effect of Ni^2+^ on L929 cells based on integrative analysis of proteomics and metabolomics.

In this article, proteomics and metabolomics techniques were used to study the effect of Ni^2+^ on L929 cells for the first time. We found that a series of proteins and metabolites were differentially expressed after Ni^2+^ treatment, indicating that the synthesis of proteins and metabolites in cells was affected by Ni^2+^. Through upstream and downstream relationship analysis and functional verification of differentially expressed proteins and metabolites for the first time, 2 important metabolic pathways, 4 metabolites and 17 proteins were identified. Ni^2+^ regulated the synthesis of downstream metabolites by affecting the expression of upstream proteins in the glutathione metabolic pathway and the arginine and proline metabolic pathway, reducing the antioxidant capacity of cells, increasing the O_2_^–^ levels and R_GSSG/GSH_, inducing oxidative stress and decreasing the ATP content, leading to abnormal energy metabolism and aggravated apoptosis (Fig. 11).

Our previous research using a cDNA microarray at the genome-wide level showed that Ni^2+^ could affect the extracellular matrix, destroy the actin cytoskeleton, increase the production of ROS, lead to oxidative stress, cause abnormal energy metabolism, induce DNA damage, affect protein synthesis, arrest the cell cycle and finally induce apoptosis and inhibit cell proliferation [5, 6]. The results of this article were basically consistent with the above findings. Furthermore, through a focused analysis of the metabolic pathways in which proteins and metabolites have upstream and downstream relationships and their functions, we found that oxidative stress, abnormal energy metabolism and apoptosis might be the key mechanisms underlying the effect of Ni^2+^ on L929 cells.

## Supplementary data

Supplementary data are available at *REGBIO* online.

## Funding

This study received the support of the National Natural Science Foundation of China (31971254).

*Conflicts of interest statement*. None declared.

## Supplementary Material

rbac040_Supplementary_Data
