# Supplementary material for: The interaction mechanism of nickel ions with L929 cells based on integrative analysis of proteomics and metabolomics data
Source: Regen Biomater. 2022 Jun 23;9:rbac040. doi: 10.1093/rb/rbac040 (PMC9258689; doi:10.1093/rb/rbac040)
Supplement: rbac040_Supplementary_Data [file rbac040_supplementary_data.docx]

**Supplementary Tables S1:** The differentially expressed protein in L929 cells after treatment with 100 μM Ni^2+^ for 12h.

| UniProt Accession | Gene ID | Gene symbol | Ratio | P-Value |
| --- | --- | --- | --- | --- |
| P18828 | 20969 | sdc1 | 1.6497942 | 0.0020945 |
| P11087 | 12842 | COL1A1 | 1.6415649 | 3.595E-05 |
| G5E898 | 19041 | PPL | 1.5665273 | 0.0008524 |
| Q99J93 | 80876 | IFITM2 | 1.4418926 | 0.0044916 |
| O88207 | 12831 | COL5A1 | 1.4142517 | 0.002773 |
| Q91YE3 | 112405 | EGLN1 | 1.4117342 | 0.0004491 |
| H3BLI9 | 21807 | TSC22D1 | 1.3885764 | 0.002332 |
| P11157 | 20135 | RRM2 | 1.3597716 | 0.0061085 |
| Q8BG60 | 56338 | TXNIP | 1.3434578 | 0.000328 |
| P17809 | 20525 | SLC2A1 | 1.3171113 | 0.0094295 |
| Q920L1 | 76267 | FADS1 | 1.283039 | 0.0053165 |
| Q922Q1 | 67247 | MARC2 | 1.281843 | 0.0082222 |
| P02463 | 12826 | COL4A1 | 1.278095 | 0.0022795 |
| P08122 | 12827 | COL4A2 | 1.2220177 | 0.0018489 |
| Q3UIS6 | 21761 | MORF4L1 | 1.2110448 | 0.0070654 |
| E9PVX6 | 17345 | MKI67 | 1.2029316 | 0.0056216 |
| Q9CPQ8 | 27425 | ATP5L | 0.8330516 | 0.0041278 |
| Q9CPT4 | 28106 | MYDGF | 0.8329551 | 0.0050936 |
| P35979 | 269261 | RPL12 | 0.8329535 | 0.0041944 |
| Q9D0S9 | 68917 | HINT2 | 0.8329316 | 0.007755 |
| H3BKE1 | 269437 | PLCH1 | 0.8326601 | 0.0029112 |
| Q922Q8 | 98238 | LRRC59 | 0.8325011 | 0.00298 |
| Q61753 | 236539 | PHGDH | 0.8323986 | 0.0001171 |
| Q8VE88 | 67726 | FAM114A2 | 0.8323271 | 0.0052163 |
| Q9ES97 | 20168 | RTN3 | 0.8322043 | 0.0046296 |
| P54116 | 13830 | STOM | 0.8319634 | 0.002513 |
| Q9JKR6 | 12282 | HYOU1 | 0.8318104 | 1.176E-05 |
| E9Q035 |  |  | 0.8310922 | 0.0006613 |
| P70279 | 20935 | SURF6 | 0.8309217 | 0.0086719 |
| P61161 | 66713 | ACTR2 | 0.8308405 | 0.0032229 |
| Q8CCJ3 | 67490 | UFL1 | 0.8306752 | 0.0066509 |
| P62806 | 69386 | Hist1h4h | 0.8306231 | 0.0010333 |
| Q91V55 | 20103 | RPS5 | 0.8305753 | 0.0002283 |
| O54984 | 56495 | ASNA1 | 0.8300476 | 0.0070089 |
| P97384 | 11744 | ANXA11 | 0.8300415 | 0.0069357 |
| Q9JIF7 | 70349 | COPB1 | 0.8288586 | 0.0008056 |
| Q9EQQ9 | 76055 | MGEA5 | 0.8285716 | 0.0007381 |
| P23591 | 22122 | TSTA3 | 0.828327 | 0.0003861 |
| Q8BH59 | 78830 | SLC25A12 | 0.8280578 | 0.0054014 |
| P62918 | 26961 | RPL8 | 0.8280317 | 0.0037772 |
| Q9CZD3 | 353172 | GARS | 0.8277297 | 0.0019737 |
| Q8R5L1 | 12261 | C1QBP | 0.8270686 | 0.0002141 |
| Q8R509 | 19205 | PTBP1 | 0.8265955 | 0.0016541 |
| P62717 | 76808 | RPL18A | 0.8262494 | 0.0032437 |
| Q8CHW4 | 224045 | EIF2B5 | 0.8259087 | 0.0032199 |
| Q3UD67 | 234734 | AARS | 0.8256984 | 0.0003815 |
| Q9JMD0 | 22680 | Zfp207 | 0.8256009 | 0.0057684 |
| Q8K2H2 | 72201 | OTUD6B | 0.8246988 | 0.000958 |
| P35293 | 19330 | RAB18 | 0.82433 | 0.002228 |
| Q91W09 | 14151 | FECH | 0.8231991 | 0.0049771 |
| P97351 | 20091 | Rps3a1 | 0.8230265 | 0.0009065 |
| Q60676 | 19060 | PPP5C | 0.8228042 | 0.0016841 |
| P51410 | 20005 | RPL9 | 0.8227784 | 0.0005637 |
| Q9D967 | 67881 | MDP1 | 0.8221333 | 0.0035756 |
| Q3U6F1 | 19664 | RBPJ | 0.8216263 | 0.0045589 |
| Q7TT37 | 230233 | IKBKAP | 0.8216094 | 0.0031821 |
| Q8BML9 | 97541 | QARS | 0.8215556 | 0.0048532 |
| A7ISP9 | 78798 | EML4 | 0.8207443 | 0.0004851 |
| Q3TUQ7 | 105787 | prkaa1 | 0.8205891 | 0.0010292 |
| Q8C1X9 | 11745 | ANXA3 | 0.8198751 | 0.0018077 |
| Q14DR9 | 67475 | ERO1LB | 0.8198221 | 0.0067065 |
| Q3UF82 | 26413 | MAPK1 | 0.8197903 | 0.0010393 |
| Q8BJW5 | 68979 | NOL11 | 0.8197605 | 0.0065265 |
| Q5SWU9 | 107476 | ACACA | 0.8191764 | 0.0037198 |
| Q8K0C4 | 13121 | CYP51 | 0.8189474 | 0.0060608 |
| D3Z0G0 | 65102 | NIF3L1 | 0.8189211 | 0.009065 |
| Q6ZWZ6 | 20042 | RPS12 | 0.8189001 | 0.0003812 |
| P70698 | 51797 | Ctps | 0.8188487 | 0.0004515 |
| Q9D8E6 | 67891 | RPL4 | 0.8183392 | 0.0017387 |
| Q3UA17 | 56428 | MTCH2 | 0.8178208 | 0.0002525 |
| O88477 | 140486 | IGF2BP1 | 0.817569 | 0.0078206 |
| Q99JI6 | 215449 | RAP1B | 0.8173671 | 0.004296 |
| P28271 | 11428 | ACO1 | 0.8172115 | 0.0066798 |
| Q8QZY1 | 223691 | EIF3L | 0.8170641 | 0.002055 |
| Q91WQ3 | 107271 | YARS | 0.8166136 | 0.0001033 |
| Q3TWN8 | 56454 | ALDH18A1 | 0.8156173 | 0.0003448 |
| Q8BFY9 | 238799 | TNPO1 | 0.8148587 | 0.0023221 |
| P24288 | 12035 | BCAT1 | 0.8147384 | 0.0076868 |
| Q8BZA9 | 319801 | TIGAR | 0.8146918 | 0.0011171 |
| O08749 | 13382 | DLD | 0.8140508 | 0.0028634 |
| Q9D0I9 | 104458 | RARS | 0.812377 | 0.0014343 |
| Q80UK8 | 70422 | INTS2 | 0.8121943 | 0.0011002 |
| P18155 | 17768 | mthfd2 | 0.8120419 | 0.0016646 |
| P70388 | 19360 | RAD50 | 0.8113437 | 0.0001508 |
| Q64674 | 20810 | SRM | 0.8112751 | 0.001567 |
| Q9JJI8 | 67671 | RPL38 | 0.8111334 | 0.0057922 |
| Q3USP3 | 78244 | DNAJC21 | 0.8107436 | 0.006372 |
| Q5SWD9 | 104662 | TSR1 | 0.8104567 | 4.427E-05 |
| Q9R0X4 | 56360 | ACOT9 | 0.8104267 | 0.0021062 |
| Q6PHZ1 | 319195 | RPL17 | 0.8098152 | 0.0002164 |
| Q3TFF0 | 56445 | DNAJA2 | 0.8095475 | 0.0002951 |
| E9PYJ6 | 230393 | FOCAD | 0.8091995 | 0.0014463 |
| Q9CQB4 | 67530 | Uqcrb | 0.8089873 | 0.0024527 |
| Q9JMH6 | 50493 | TXNRD1 | 0.8084182 | 0.001857 |
| O70569 |  |  | 0.8080394 | 0.0007723 |
| Q8VC28 | 27384 | Akr1c13 | 0.8078058 | 0.0011134 |
| Q9CPY7 | 66988 | LAP3 | 0.8071546 | 0.0021093 |
| Q99N15 | 15108 | HSD17B10 | 0.8065799 | 0.0017588 |
| Q3ULZ3 | 107272 | PSAT1 | 0.8064448 | 0.0009488 |
| Q8BLN5 | 16987 | LSS | 0.8057466 | 0.0051075 |
| Q3TJA9 | 83945 | DNAJA3 | 0.802923 | 0.0026237 |
| Q9D8M4 | 66229 | RPL7L1 | 0.8029115 | 0.0024876 |
| P62900 | 114641 | RPL31 | 0.8028693 | 0.0009764 |
| P70188 | 16579 | KIFAP3 | 0.8023734 | 0.0039533 |
| Q8BPS5 | 20501 | SLC16A1 | 0.8020939 | 0.0042794 |
| P62889 | 19946 | RPL30 | 0.800963 | 0.0008106 |
| P62855 | 27370 | RPS26 | 0.8004541 | 0.0005838 |
| P62270 | 20084 | RPS18 | 0.8002884 | 0.001407 |
| P47740 | 11671 | ALDH3A2 | 0.8001557 | 0.0021879 |
| Q6P069 | 109552 | SRI | 0.7991156 | 0.0001031 |
| Q8BGS0 | 67920 | MAK16 | 0.7989123 | 0.000584 |
| A0A087WQE6 | 67923 | TCEB1 | 0.7982008 | 0.0019089 |
| Q8BWZ3 | 231713 | NAA25 | 0.797708 | 0.003631 |
| Q9D1M7 | 66120 | FKBP11 | 0.7974584 | 0.0019178 |
| A2A547 | 19921 | RPL19 | 0.7967284 | 0.0023716 |
| A2A4B3 | 64899 | LPIN3 | 0.7963554 | 0.0036938 |
| Q66L45 | 227695 | SPOUT1 | 0.7945038 | 0.0057192 |
| Q3UHX2 | 231887 | PDAP1 | 0.7940957 | 0.0011092 |
| Q9D7S7 | 68028 | RPL22L1 | 0.7935697 | 0.0071939 |
| Q9JI13 | 65961 | UTP3 | 0.7935383 | 0.0014819 |
| G3X8X7 | 80743 | VPS16 | 0.7924417 | 0.001557 |
| F6YQT7 | 320355 | LIPI | 0.7923839 | 0.0047624 |
| P81117 | 53322 | NUCB2 | 0.7913156 | 0.0012816 |
| Q62422 | 20409 | OSTF1 | 0.7912119 | 0.0041908 |
| A2BDX2 | 13480 | DPM1 | 0.7909339 | 0.0025082 |
| Q3U561 | 19896 | RPL10A | 0.7899614 | 0.0001219 |
| P63276 | 20068 | RPS17 | 0.789617 | 0.0012731 |
| A2AKI5 | 16410 | ITGAV | 0.7893637 | 0.0005996 |
| E9Q555 | 672511 | RNF213 | 0.789354 | 0.0010867 |
| Q8BI84 | 338366 | MIA3 | 0.7879788 | 0.0003713 |
| Q00612 | 14381 | G6pdx | 0.7874673 | 0.0003749 |
| Q8VDP6 | 52858 | CDIPT | 0.7858391 | 0.0054241 |
| P59325 | 217869 | EIF5 | 0.7852923 | 0.0005523 |
| Q99JX4 | 98221 | EIF3M | 0.7843016 | 0.0003409 |
| Q03145 | 13836 | EPHA2 | 0.7838254 | 0.0003077 |
| Q8BP47 | 70223 | NARS | 0.7831893 | 1.427E-05 |
| Q9CTI8 | 73834 | ATP6V1D | 0.7828394 | 0.0002089 |
| B2RY90 | 664994 | Isoc2a | 0.7826398 | 0.000626 |
| D3YXP6 | 68603 | pmvk | 0.7808269 | 0.0010591 |
| P35505 | 14085 | FAH | 0.7791207 | 0.0001632 |
| Q91W92 | 104445 | CDC42EP1 | 0.7789271 | 0.0029097 |
| Q91VC9 | 66092 | GHITM | 0.7786942 | 0.0072443 |
| Q5HZH2 | 68327 | TSR3 | 0.7780872 | 0.003376 |
| O88398 | 11567 | AVIL | 0.7775475 | 0.0002772 |
| E9QAZ2 | 100039748 | Gm10020 | 0.7736659 | 0.0025423 |
| Q78JE5 | 71999 | FBXO22 | 0.7734599 | 0.0038745 |
| Q9CX60 | 77889 | LBH | 0.7724477 | 0.0003269 |
| I4DCY6 | 18391 | SIGMAR1 | 0.7723286 | 0.0099526 |
| Q6ZWU9 | 57294 | RPS27 | 0.7717582 | 0.001792 |
| D3Z5I1 | 78781 | ZC3HAV1 | 0.7700368 | 0.0007572 |
| Q8BGP6 | 319653 | SLC25A40 | 0.768452 | 0.0095367 |
| P10852 | 17254 | SLC3A2 | 0.7683866 | 0.0028143 |
| B2RQQ5 | 17755 | MAP1B | 0.7608697 | 0.0007585 |
| P47955 | 56040 | RPLP1 | 0.7605179 | 0.0004997 |
| Q3TV20 | 27053 | ASNS | 0.7600306 | 5.215E-05 |
| Q3US29 | 110196 | FDPS | 0.7581841 | 0.000107 |
| P15864 | 50708 | HIST1H1C | 0.7579126 | 0.0009365 |
| Q9CR25 | 67728 | DPH2 | 0.7574314 | 0.0016625 |
| Q9QYR9 | 171210 | ACOT2 | 0.7512493 | 0.0002042 |
| Q62241 | 20630 | SNRPC | 0.7457986 | 0.0066712 |
| Q8CGP5 | 319173 | Hist1h2af | 0.7440419 | 0.0001874 |
| Q9D9V3 | 52665 | ECHDC1 | 0.7425405 | 0.0073226 |
| Q4VA28 | 19933 | RPL21 | 0.7417566 | 0.000939 |
| Q8BHC7 | 76867 | RHBDD1 | 0.7332828 | 0.004247 |
| Q8VED9 | 216551 | LGALSL | 0.7309756 | 0.0001862 |
| P07141 | 12977 | CSF1 | 0.7306649 | 0.0014546 |
| Q99LS3 | 100678 | PSPH | 0.7173395 | 0.0072886 |
| Q3U6U7 | 22375 | WARS | 0.7143688 | 0.0003756 |
| Q8BGZ7 | 109052 | KRT75 | 0.709469 | 0.0096156 |
| Q78RK2 | 170460 | STARD5 | 0.7063019 | 0.0018138 |
| Q1W5W7 |  |  | 0.7058068 | 0.0034912 |
| Q8VCN5 | 107869 | CTH | 0.6916369 | 0.0002814 |
| Q3THZ8 | 54342 | GNPNAT1 | 0.6487507 | 0.0003905 |
| P09528 | 14319 | FTH1 | 0.646619 | 2.351E-06 |
| Q3THE6 |  |  | 0.5792602 | 0.0068956 |
| Q64735 | 12946 | CR1L | 0.5580999 | 0.0078204 |
| Q5SSP3 | 55963 | SLC1A4 | 0.5490087 | 0.0001578 |

**Supplementary Tables S2:** The differentially expressed protein in L929 cells after treatment with 100 μM Ni^2+^ for 24h.

| UniProt Accession | Gene ID | Genesymbol | Ratio | P-Value |
| --- | --- | --- | --- | --- |
| Q3URT2 | 18187 | NRP2 | 1.877162171 | 0.0084072 |
| P18828 | 20969 | sdc1 | 1.392286339 | 0.0030969 |
| Q9D7B7 | 69590 | GPX8 | 0.825745755 | 0.0010678 |
| Q8C6B9 | 66538 | rps19bp1 | 0.814684996 | 0.0047921 |
| A2AWI9 | 227700 | SH3GLB2 | 0.810127055 | 0.0010311 |
| Q80W68 | 170643 | KIRREL | 0.781566691 | 0.0017955 |
| P17563 | 20341 | SELENBP1 | 0.779976698 | 0.0029097 |
| Q8CAC4 | 12572 | CDK7 | 0.779669001 | 0.0016265 |
| Q3TDE6 | 74551 | PCK2 | 0.775703934 | 0.0085369 |
| P52293 | 16647 | KPNA2 | 0.773773469 | 0.0005893 |
| Q8BZA9 | 319801 | TIGAR | 0.765118409 | 0.003883 |
| Q3UMQ8 | 234344 | NAF1 | 0.762876694 | 0.0008796 |
| D3YZC1 | 66904 | PCCB | 0.761383293 | 0.0013621 |
| Q5QNU0 | 19069 | NUP88 | 0.755330475 | 0.0021801 |
| Q8K039 | 66202 | 1110059G10Rik | 0.754534485 | 0.0079001 |
| Q3TVD9 | 72039 | MCCC1 | 0.748349567 | 0.0057814 |
| Q3TKT4 | 20586 | SMARCA4 | 0.746890945 | 6.606E-05 |
| Q9D0B6 | 67683 | PBDC1 | 0.74455614 | 0.0008056 |
| Q99KC3 | 110109 | NOP2 | 0.744395969 | 0.0055809 |
| Q8K0L1 | 104776 | ALDH6A1 | 0.739954569 | 0.0032953 |
| Q3TRX4 | 18483 | PALM | 0.738438715 | 0.0004692 |
| Q3TJH1 | 14679 | gnai3 | 0.738106175 | 0.0029904 |
| G5E8J3 | 207425 | WDR11 | 0.737896545 | 0.0054793 |
| Q61655 | 13680 | DDX19A | 0.736238681 | 0.0051935 |
| P16675 | 19025 | CTSA | 0.734686976 | 0.0004361 |
| Q3UT02 | 207214 | LARP4 | 0.732907833 | 0.0037288 |
| A7ISP9 | 78798 | EML4 | 0.731885068 | 0.0005182 |
| D3Z4U3 | 53323 | UBE2K | 0.729948268 | 0.0024121 |
| D3YYP5 | 54722 | DFNA5 | 0.728460333 | 0.0056319 |
| Q922H4 | 69080 | GMPPA | 0.728234998 | 0.0001245 |
| Q810A7 | 72047 | DDX42 | 0.727331629 | 0.0079179 |
| P84089 | 13877 | ERH | 0.726119 | 0.0007136 |
| D3Z0B9 | 69748 | ALDH16A1 | 0.725633042 | 0.0003005 |
| Q8BP71 | 93686 | RBFOX2 | 0.725124757 | 0.0059778 |
| G8JL40 | 99982 | KDM1A | 0.725009367 | 0.0056335 |
| E9QKG6 | 81702 | ANKRD17 | 0.724813279 | 0.0022614 |
| Q8CHP8 | 67078 | PGP | 0.724489424 | 0.0009596 |
| Q3UTW6 | 13591 | EBF1 | 0.722919974 | 0.0004931 |
| Q3U4F0 | 94280 | SFXN3 | 0.720766558 | 0.0079675 |
| G3UWZ0 | 217578 | BAZ1A | 0.720155764 | 0.0047019 |
| Q3UJS6 | 70568 | CPNE3 | 0.720002765 | 0.0008036 |
| Q8BTI8 | 75956 | SRRM2 | 0.717521336 | 0.0075662 |
| Q80Y81 | 68626 | ELAC2 | 0.716986036 | 0.0081175 |
| Q3U935 | 12785 | CNBP | 0.716839319 | 0.0005679 |
| Q3TD41 | 217127 | KAT7 | 0.716102142 | 0.0022522 |
| B7ZNJ0 | 11491 | ADAM17 | 0.715382339 | 7.514E-05 |
| Q8CGF7 | 56070 | TCERG1 | 0.714444375 | 0.0061493 |
| P11157 | 20135 | RRM2 | 0.714345343 | 0.0067732 |
| P70302 | 20866 | STIM1 | 0.71327301 | 0.0003335 |
| Q8VCF0 | 228607 | MAVS | 0.713231087 | 0.0005681 |
| Q5HZH2 | 68327 | TSR3 | 0.712931221 | 0.0091585 |
| Q1KYM2 |  |  | 0.711731424 | 0.0006093 |
| Q61033 | 21917 | TMPO | 0.711469047 | 3.925E-05 |
| E9Q4M4 | 66098 | CHCHD6 | 0.711179981 | 0.0025508 |
| Q3TJ76 | 320951 | PISD | 0.710134392 | 0.0024799 |
| Q6PIU9 | 102637099 | Gm38495 | 0.709709618 | 0.0083557 |
| Q99J09 | 70465 | WDR77 | 0.707700084 | 0.0001025 |
| D3YWS7 | 69064 | FUOM | 0.706909398 | 0.0004628 |
| Q6IWE2 | 11461 | ACTB | 0.706464627 | 0.0016708 |
| Q3TDD9 | 73825 | PPP1R21 | 0.706266787 | 0.0065004 |
| Q3THG9 | 69684 | AARSD1 | 0.706092544 | 0.0005499 |
| Q3V300 | 110033 | KIF22 | 0.706029544 | 0.0095352 |
| P10605 | 13030 | CTSB | 0.705125137 | 0.0012064 |
| Q3UJ44 | 12332 | CAPG | 0.703210878 | 7.556E-05 |
| Q4FJZ2 | 16650 | KPNA6 | 0.702963343 | 0.0085999 |
| O35218 | 51786 | CPSF2 | 0.702644634 | 0.0032916 |
| O08583 | 21681 | ALYREF | 0.70257864 | 0.0033588 |
| Q8C2E7 | 223593 | E430025E21Rik | 0.702375866 | 3.327E-05 |
| Q5NC05 | 74044 | TTF2 | 0.697936005 | 0.003243 |
| Q8K2M0 | 60441 | MRPL38 | 0.69792771 | 0.0044454 |
| O08848 | 20822 | TROVE2 | 0.697706854 | 0.0074168 |
| Q9D1I2 | 68480 | CARD19 | 0.696713832 | 0.001212 |
| Q3UID0 | 68094 | SMARCC2 | 0.696615637 | 0.0058852 |
| Q8BY71 | 107435 | HAT1 | 0.69623762 | 0.0002889 |
| B2RSW8 | 18536 | PCM1 | 0.696219033 | 0.000267 |
| Q6GQT9 | 211548 | nomo1 | 0.695843159 | 0.0002592 |
| D6RG99 | 14356 | TIMM10B | 0.694397024 | 0.0024484 |
| E9Q0W8 | 20643 | SNRPE | 0.694138852 | 0.0046679 |
| O54782 | 17160 | man2b2 | 0.693367732 | 0.0007162 |
| Q3U057 | 54451 | CPSF3 | 0.692593343 | 0.0001235 |
| Q3USX2 | 83602 | GTF2A1 | 0.691892824 | 0.0041323 |
| Q8BJW6 | 229317 | EIF2A | 0.691601395 | 0.0041675 |
| B9EKJ7 | 228140 | TNKS1BP1 | 0.691125448 | 0.0001046 |
| Q3U468 | 12505 | CD44 | 0.690249505 | 0.0014961 |
| Q9DC61 | 66865 | PMPCA | 0.690129164 | 0.0026049 |
| Q9R0B9 | 26432 | PLOD2 | 0.68981853 | 0.0014219 |
| Q8BGB7 | 67870 | ENOPH1 | 0.689502366 | 0.0014759 |
| Q7TQK1 | 77065 | INTS7 | 0.689296764 | 0.0050944 |
| Q9CPQ3 | 223696 | TOMM22 | 0.689113447 | 0.0043537 |
| Q07417 | 11409 | ACADS | 0.688713236 | 0.0070812 |
| A0A087WQE6 | 67923 | TCEB1 | 0.68845366 | 0.0043101 |
| Q8VBV3 | 227715 | EXOSC2 | 0.687976843 | 0.0040361 |
| Q3UDS4 | 59010 | SQRDL | 0.687901215 | 5.555E-05 |
| P40336 | 30930 | VPS26A | 0.687846312 | 0.001684 |
| P57784 | 68981 | SNRPA1 | 0.687434466 | 0.0010614 |
| Q3TQI7 | 227707 | BC005624 | 0.686875694 | 0.0031989 |
| E9QAT0 | 14265 | FMR1 | 0.684958604 | 0.0005841 |
| E9Q4Q2 | 22668 | SF1 | 0.684860449 | 0.0024453 |
| P27601 | 14674 | GNA13 | 0.684697235 | 0.0059059 |
| E9PWB1 | 102693 | PHLDB1 | 0.684600114 | 0.0035495 |
| O55201 | 20924 | Supt5 | 0.684595335 | 0.0006493 |
| Q8K3X4 | 238330 | IRF2BPL | 0.68434628 | 0.0053105 |
| Q8CHK3 | 77582 | MBOAT7 | 0.683845013 | 0.0025454 |
| Q3TZP3 | 23942 | MTA2 | 0.683811697 | 0.0019729 |
| Q8R3B1 | 18799 | PLCD1 | 0.683761655 | 0.0076557 |
| Q6ZWM4 | 76522 | LSM8 | 0.683389896 | 0.0001761 |
| Q9QXB9 | 13495 | DRG2 | 0.683227379 | 0.0036323 |
| Q9CX60 | 77889 | LBH | 0.682883333 | 0.0003492 |
| Q9CT36 | 72119 | TPX2 | 0.682020526 | 0.0015966 |
| P43274 | 50709 | HIST1H1E | 0.681533473 | 0.0033762 |
| A2A4Z1 | 68612 | UBE2C | 0.681036842 | 0.0077264 |
| Q9D1I6 | 68463 | MRPL14 | 0.680852309 | 0.0064307 |
| Q8R038 | 60315 | myg1 | 0.676257441 | 0.0003092 |
| Q9QXK3 | 54160 | COPG2 | 0.675994244 | 0.0024255 |
| Q99JI6 | 215449 | RAP1B | 0.675386732 | 0.0008602 |
| Q8VBT6 | 171504 | APOBR | 0.675318586 | 0.0004744 |
| Q62418 | 13169 | DBNL | 0.673902696 | 0.0002657 |
| Q7TQ39 |  |  | 0.673769181 | 0.0009261 |
| Q64310 | 20932 | SURF4 | 0.672763107 | 0.0003551 |
| Q61578 | 14149 | FDXR | 0.672716637 | 0.0006756 |
| Q99JR1 | 14057 | SFXN1 | 0.672539979 | 0.0025024 |
| Q8BU33 | 216136 | ILVBL | 0.67251603 | 0.0047536 |
| P28650 | 11565 | ADSSL1 | 0.671445127 | 0.0001174 |
| D3YYC2 | 66556 | DRAP1 | 0.671299217 | 0.0041375 |
| Q9JLI8 | 53890 | SART3 | 0.671241203 | 0.0024319 |
| Q3UMB9 | 319277 | A230046K03Rik | 0.67056028 | 0.0016375 |
| P11352 | 14775 | GPX1 | 0.670128469 | 0.0021525 |
| P70699 | 14387 | gaa | 0.66963933 | 0.0007069 |
| Q542W3 | 23881 | G3BP2 | 0.668683017 | 0.0096839 |
| P60487 | 57028 | PDXP | 0.668225611 | 0.0015951 |
| Q4TVN0 | 66687 | TBC1D15 | 0.667344253 | 0.0010932 |
| Q5SUR0 | 237823 | PFAS | 0.667272558 | 0.0025922 |
| A0A0A0MQ80 | 57815 | SPATA5 | 0.667106092 | 0.0001184 |
| Q80UK4 | 231830 | MICALL2 | 0.666671302 | 0.0022869 |
| Q9QZ82 | 13070 | CYP11A1 | 0.666098418 | 0.0016397 |
| Q9D967 | 67881 | MDP1 | 0.665197097 | 0.0004384 |
| Q8R105 | 107305 | VPS37C | 0.665032818 | 0.0052027 |
| P70441 | 26941 | SLC9A3R1 | 0.66466219 | 0.0021657 |
| G3UXW9 | 209318 | GPS1 | 0.664604214 | 0.0013183 |
| P70697 | 22275 | UROD | 0.664352654 | 0.0005811 |
| Q62383 | 20926 | Supt6 | 0.662590125 | 0.0057982 |
| P24668 | 17113 | M6PR | 0.662380251 | 0.0030254 |
| Q3TA75 | 23879 | FXR2 | 0.662264177 | 0.0019853 |
| Q80TU6 | 56215 | ACIN1 | 0.662170583 | 0.0067472 |
| Q3U643 | 276846 | PIGS | 0.661014553 | 0.0012877 |
| E9PWG6 | 54392 | NCAPG | 0.660836403 | 0.0028537 |
| Q9QXE7 | 21372 | TBL1X | 0.660808602 | 0.0010137 |
| Q8VD12 | 29813 | Zfp385a | 0.660550858 | 0.0080279 |
| P58742 | 223921 | AAAS | 0.659747657 | 0.0061325 |
| P62315 | 20641 | SNRPD1 | 0.659662414 | 0.0027745 |
| Q9DC50 | 74114 | CROT | 0.659553167 | 0.0025896 |
| Q8C9L6 | 11641 | AKAP2 | 0.659058339 | 0.0052972 |
| Q921M7 | 223601 | FAM49B | 0.658205672 | 0.0003722 |
| Q8BV40 | 74197 | GTF2E1 | 0.657793567 | 7.223E-05 |
| B7ZWM8 | 70144 | LRCH3 | 0.657217419 | 0.0069505 |
| B2RWW1 | 270058 | MAP1S | 0.657216048 | 0.0017163 |
| Q11136 | 18624 | PEPD | 0.657030631 | 0.0046416 |
| O08614 | 22288 | UTRN | 0.656122443 | 0.0001567 |
| Q8R059 | 74246 | GALE | 0.65480431 | 0.0052203 |
| B1AZ15 | 319876 | COBLL1 | 0.653655124 | 0.0033832 |
| Q3TKB7 | 66973 | MRPS18B | 0.653171708 | 0.0048547 |
| H7BWX9 | 170930 | SUMO2 | 0.652831896 | 0.0017354 |
| P25206 | 17215 | MCM3 | 0.650935732 | 0.0002667 |
| Q8BGP6 | 319653 | SLC25A40 | 0.65080377 | 0.0016802 |
| Q8C545 |  |  | 0.650710307 | 0.0020056 |
| Q8BTS4 | 269113 | NUP54 | 0.650227274 | 0.0055677 |
| P49718 | 17218 | MCM5 | 0.650066583 | 0.0001301 |
| Q8VE88 | 67726 | FAM114A2 | 0.65005078 | 0.0021749 |
| P20152 | 22352 | VIM | 0.649323406 | 8.653E-05 |
| Q8K4Q8 | 140792 | COLEC12 | 0.649316641 | 0.0005642 |
| Q3UU20 | 217310 | HID1 | 0.64922132 | 0.0013464 |
| Q8VBT9 | 68938 | ASPSCR1 | 0.648873064 | 0.0008205 |
| Q3TV93 | 56150 | MAD2L1 | 0.648724424 | 0.0002956 |
| Q64337 | 18412 | SQSTM1 | 0.648651615 | 0.0015975 |
| Q3U0V1 | 16549 | KHSRP | 0.648295455 | 0.000244 |
| Q9Z0H8 | 269713 | CLIP2 | 0.648109893 | 0.0026759 |
| P20108 | 11757 | PRDX3 | 0.647549761 | 0.0005588 |
| Q91YP0 | 217666 | L2HGDH | 0.647203515 | 7.857E-05 |
| Q9DCB8 | 74316 | ISCA2 | 0.64698413 | 0.0001814 |
| P31324 | 19088 | PRKAR2B | 0.646579723 | 0.0007014 |
| Q8C0I1 | 228061 | AGPS | 0.646528711 | 6.568E-05 |
| O88712 | 13016 | CTBP1 | 0.646479155 | 4.812E-05 |
| Q99N87 | 77721 | MRPS5 | 0.6462937 | 0.0004957 |
| D3Z0G0 | 65102 | NIF3L1 | 0.646189928 | 0.0010928 |
| Q8BTW3 | 72544 | EXOSC6 | 0.645877271 | 0.0001057 |
| Q3UHD6 | 76742 | SNX27 | 0.645844694 | 0.0062037 |
| Q8R5K4 | 230082 | NOL6 | 0.645311005 | 0.0022555 |
| A2AFG7 | 16728 | L1CAM | 0.64514447 | 0.0028545 |
| O88986 | 26912 | GCAT | 0.644722176 | 0.0002541 |
| P62307 | 69878 | SNRPF | 0.644683543 | 0.0064817 |
| Q3UM23 | 107702 | RNH1 | 0.644348836 | 0.0003665 |
| A2AL12 | 229279 | HNRNPA3 | 0.644201404 | 9.98E-06 |
| Q9R059 | 14201 | FHL3 | 0.644116571 | 0.0008295 |
| Q9D0G0 | 59054 | MRPS30 | 0.644078895 | 6.971E-05 |
| P53702 | 15159 | HCCS | 0.643872087 | 2.853E-05 |
| A2CG44 | 17127 | SMAD3 | 0.643391017 | 0.0034847 |
| Q3T992 | 56085 | UBQLN1 | 0.643131069 | 0.0094005 |
| Q02819 | 18220 | NUCB1 | 0.642839858 | 0.000568 |
| Q80YC2 | 15516 | HSP90AB1 | 0.642623686 | 0.0008229 |
| Q9QXT0 | 56530 | CNPY2 | 0.642610789 | 0.0051677 |
| Q9CQD1 | 271457 | RAB5A | 0.641833061 | 3.036E-05 |
| E9QLB8 | 76889 | COQ8B | 0.641726229 | 0.0001509 |
| G3X922 | 235567 | DNAJC13 | 0.641613811 | 0.0010549 |
| Q8K1N4 | 72572 | SPATS2 | 0.641531434 | 0.0002207 |
| Q9CYG7 | 67145 | TOMM34 | 0.641043987 | 0.0002471 |
| B1AZ46 | 108100 | BAIAP2 | 0.641032389 | 0.0075697 |
| P97384 | 11744 | ANXA11 | 0.640607607 | 0.0054205 |
| P62915 | 229906 | GTF2B | 0.640479243 | 0.0095493 |
| Q9CR60 | 66964 | GOLT1B | 0.640259211 | 0.001681 |
| P56389 | 72269 | CDA | 0.639652289 | 0.0025334 |
| P35278 | 19345 | RAB5C | 0.639463171 | 0.0090403 |
| H3BKN0 | 28114 | NSUN2 | 0.63938293 | 0.0001025 |
| D3YW48 | 12336 | CAPNS1 | 0.639336155 | 0.0019319 |
| Q8R3F5 | 223722 | MCAT | 0.639087588 | 0.0012776 |
| P62141 | 19046 | ppp1cb | 0.638442625 | 0.0047659 |
| D3Z795 | 56088 | PSMG1 | 0.638312425 | 0.0069774 |
| Q3U3L3 | 20719 | Serpinb6a | 0.638199447 | 3.569E-05 |
| Q80ZW2 | 223626 | THEM6 | 0.637976624 | 0.0086627 |
| P59999 | 68089 | ARPC4 | 0.637975279 | 0.0004481 |
| Q3V0L4 | 56878 | RBMS1 | 0.637916771 | 0.0025689 |
| Q91WM3 | 27966 | RRP9 | 0.637731258 | 0.0033494 |
| D3Z5P5 | 20589 | IGHMBP2 | 0.637351191 | 0.0085577 |
| P05064 | 11674 | ALDOA | 0.637348719 | 2.056E-06 |
| Q9Z247 | 27055 | FKBP9 | 0.637304648 | 0.0022407 |
| P12815 | 18570 | PDCD6 | 0.636677976 | 0.003277 |
| O35130 | 14791 | EMG1 | 0.635923071 | 1.186E-05 |
| Q9JJE7 | 60527 | FADS3 | 0.635815754 | 0.000292 |
| Q3TIJ4 | 66368 | RTCA | 0.635463049 | 0.0028131 |
| E9PYJ6 | 230393 | FOCAD | 0.635145148 | 0.0006517 |
| Q3UHJ0 | 269774 | AAK1 | 0.635083272 | 0.0012698 |
| Q3TW36 | 108077 | SKIV2L | 0.634749367 | 0.000201 |
| P08752 | 14678 | GNAI2 | 0.634186584 | 0.0007872 |
| P68181 | 18749 | PRKACB | 0.634074095 | 0.0013991 |
| Q8BMZ7 | 66914 | VPS28 | 0.633865792 | 0.0002913 |
| Q9WV03 | 108160 | FAM50A | 0.633573763 | 0.0034351 |
| O35566 | 12476 | CD151 | 0.633337042 | 0.0096953 |
| Q3UL43 | 170762 | NUP155 | 0.63324695 | 0.0007778 |
| Q8C550 | 78688 | NOL3 | 0.633226569 | 0.005004 |
| P12382 | 18641 | PFKL | 0.633028108 | 0.0009677 |
| Q8VHC3 | 114679 | selm | 0.632752643 | 0.0022603 |
| Q9QYS9 | 19317 | Qk | 0.632600044 | 0.0003683 |
| Q3TKY6 | 67285 | CWC27 | 0.632512444 | 0.0008503 |
| Q3UTE4 | 19357 | RAD21 | 0.631075058 | 0.0002301 |
| Q9CXV9 | 76863 | DCUN1D5 | 0.631014381 | 0.0058477 |
| Q9JJA4 | 57750 | WDR12 | 0.630666514 | 0.0057703 |
| Q61584 | 14359 | FXR1 | 0.630536862 | 0.0002187 |
| Q78IK4 | 68117 | APOOL | 0.630269972 | 0.0035869 |
| Q8BMG7 | 98732 | RAB3GAP2 | 0.629753049 | 0.0007292 |
| P34884 | 17319 | MIF | 0.62966765 | 5.208E-05 |
| Q9D7X3 | 72349 | DUSP3 | 0.629597776 | 0.0035021 |
| Q3UNJ3 | 14230 | FKBP10 | 0.6290816 | 8.227E-05 |
| Q5SWD9 | 104662 | TSR1 | 0.628911094 | 0.001163 |
| P50516 | 11964 | ATP6V1A | 0.62877466 | 7.368E-05 |
| Q8K4Z3 | 246703 | apoa1bp | 0.628772608 | 0.0060567 |
| Q3UF95 | 224727 | BAG6 | 0.628518248 | 0.0015133 |
| Q8R1G6 | 213019 | PDLIM2 | 0.628379869 | 0.0008503 |
| Q91WG4 | 58523 | ELP2 | 0.628327009 | 0.0012185 |
| Q8CFI7 | 231329 | POLR2B | 0.62797077 | 0.002779 |
| D3YUK4 | 68342 | NDUFB10 | 0.626147573 | 3.903E-05 |
| H3BK68 | 212528 | TRMT1 | 0.625986195 | 0.003681 |
| Q9QXS1 | 18810 | PLEC | 0.625726083 | 2.928E-06 |
| Q3TFP0 | 14105 | SRSF10 | 0.625644271 | 0.0028537 |
| A2A6U3 | 53860 | SEPT9 | 0.625626821 | 4.107E-05 |
| A0A087WSG5 | 17420 | MNAT1 | 0.625155482 | 0.0014033 |
| Q8BFW7 | 210126 | LPP | 0.625049224 | 0.0001593 |
| Q8BTE1 | 69674 | MIF4GD | 0.625039609 | 0.0011835 |
| E9Q5B5 | 15277 | HK2 | 0.624992538 | 4.073E-05 |
| Q08509 | 13860 | EPS8 | 0.624656092 | 0.0020449 |
| Q3TJN9 | 19224 | PTGS1 | 0.624545348 | 0.0005276 |
| P68373 | 22146 | TUBA1C | 0.624111487 | 2.704E-06 |
| P03958 | 11486 | ADA | 0.623899739 | 0.0099008 |
| Q3TF81 | 54364 | RPP30 | 0.623748491 | 0.0020301 |
| Q64442 | 20322 | SORD | 0.623317272 | 0.0029336 |
| Q8BIF7 | 101612 | GRWD1 | 0.623307538 | 0.0018828 |
| Q8VDM6 | 232989 | HNRNPUL1 | 0.622634091 | 0.0003711 |
| Q8BK66 | 52513 | DDX56 | 0.622553259 | 0.0013961 |
| Q8BWT1 | 52538 | ACAA2 | 0.622193632 | 9.694E-06 |
| Q91VE6 | 67949 | NIFK | 0.621868245 | 0.0002296 |
| Q91WD5 | 226646 | NDUFS2 | 0.62177103 | 0.0004906 |
| Q3U878 | 70248 | DAZAP1 | 0.621218927 | 0.0004255 |
| Q923G2 | 245841 | POLR2H | 0.620823603 | 0.0051934 |
| Q9CXJ1 | 67417 | EARS2 | 0.62048023 | 0.0089241 |
| P61961 | 67890 | UFM1 | 0.620246959 | 0.003048 |
| Q6PDM2 | 110809 | SRSF1 | 0.619678785 | 0.000253 |
| Q6NXX6 | 11975 | ATP6V0A1 | 0.619547042 | 0.0042103 |
| Q99LN9 | 102115 | DOHH | 0.619308595 | 0.0008779 |
| Q3TNH0 | 21917 | TMPO | 0.619196332 | 0.000193 |
| D3Z619 |  |  | 0.618833338 | 7.839E-05 |
| Q64521 | 14571 | GPD2 | 0.618637921 | 7.117E-05 |
| Q3V493 | 60409 | TRAPPC4 | 0.61854846 | 0.0095442 |
| Q3TLI6 | 75597 | ndufaf2 | 0.618043417 | 8.622E-05 |
| E9Q6Q8 | 210789 | TBC1D4 | 0.617953311 | 0.0004687 |
| P49962 | 27058 | SRP9 | 0.617922881 | 0.002192 |
| Q921M3 | 101943 | SF3B3 | 0.617813881 | 0.0001337 |
| Q8K3W0 | 107976 | BRE | 0.617433755 | 0.0043449 |
| Q91V41 | 68365 | RAB14 | 0.616439403 | 0.0006399 |
| O54734 | 13200 | DDOST | 0.616282494 | 0.0001099 |
| Q99K48 | 53610 | NONO | 0.616042605 | 0.0008174 |
| E9PXY1 | 72584 | CUL4B | 0.616012447 | 0.0020316 |
| Q3TQP7 | 110446 | ACAT1 | 0.615613382 | 0.0002572 |
| Q9CTI8 | 73834 | ATP6V1D | 0.615336436 | 0.0079604 |
| Q3TIX9 | 28035 | USP39 | 0.615020103 | 6.39E-05 |
| Q922S4 | 207728 | PDE2A | 0.615011754 | 0.0006138 |
| P62983 | 78294 | RPS27A | 0.614996312 | 3.247E-06 |
| F6ZFU0 | 66656 | EEF1D | 0.614592669 | 0.0029876 |
| Q61749 | 13667 | EIF2B4 | 0.614258954 | 0.0027218 |
| Q6PB44 | 104831 | PTPN23 | 0.614181444 | 0.001895 |
| Q9WTI7 | 17913 | MYO1C | 0.613754095 | 0.001024 |
| Q3TJ10 | 72141 | ADPGK | 0.613591275 | 0.0040528 |
| Q3UJP8 | 14450 | GART | 0.613423019 | 0.0001244 |
| Q6NWW1 | 63958 | UBE4B | 0.613392514 | 0.0046866 |
| G3X8Y7 | 26568 | SLC27A3 | 0.613118679 | 0.0008929 |
| G3X8R5 | 69232 | QRICH1 | 0.612938697 | 0.0063986 |
| E9QNQ4 | 76089 | RAPGEF2 | 0.612921448 | 0.009325 |
| Q9CYA0 | 76737 | CRELD2 | 0.612556441 | 7.535E-05 |
| Q9ER88 | 65111 | DAP3 | 0.612422158 | 0.0014918 |
| Q9R233 | 21356 | TAPBP | 0.612264308 | 0.0009035 |
| Q8R180 | 50527 | ERO1L | 0.611829272 | 0.0012648 |
| Q7TSV4 | 66681 | PGM1 | 0.611340129 | 0.0014453 |
| Q91VM9 | 74776 | PPA2 | 0.610953242 | 0.0006395 |
| Q8VDF2 | 18140 | UHRF1 | 0.610872077 | 0.0023696 |
| Q9JHI5 | 56357 | IVD | 0.610617743 | 0.000289 |
| Q99N93 | 94063 | MRPL16 | 0.610479287 | 5.89E-05 |
| E9QAJ9 | 70497 | ARHGAP17 | 0.610477758 | 0.0022492 |
| Q4KMV6 | 72238 | TBC1D5 | 0.610359442 | 0.004387 |
| Q8BJW5 | 68979 | NOL11 | 0.610155701 | 0.0003689 |
| Q9D706 | 71919 | RPAP3 | 0.610089202 | 0.0025502 |
| Q9R0E2 | 18822 | PLOD1 | 0.610016893 | 0.0004738 |
| O08915 | 11632 | AIP | 0.610011944 | 0.0012909 |
| Q3TJ21 | 69051 | PYCR2 | 0.609783678 | 0.0002456 |
| Q9D0M3 | 66445 | Cyc1 | 0.609642161 | 0.0036315 |
| Q3THA0 | 53356 | EIF3G | 0.609536284 | 0.0001495 |
| Q80YR5 | 224902 | SAFB2 | 0.60935373 | 0.0078764 |
| Q3ULG5 | 17219 | MCM6 | 0.60872054 | 0.0058966 |
| Q8R361 | 52055 | rab11fip5 | 0.608628887 | 0.0010273 |
| P70188 | 16579 | KIFAP3 | 0.608578766 | 0.0003958 |
| Q3U9Q8 | 227753 | GSN | 0.608386198 | 2.021E-05 |
| P52432 | 20016 | POLR1C | 0.608378582 | 0.0011953 |
| Q8C4B4 | 106840 | UNC119B | 0.608255499 | 0.0007603 |
| Q8R146 | 235606 | APEH | 0.608253971 | 0.0080263 |
| Q3U6X7 | 12566 | CDK2 | 0.608117367 | 0.0008489 |
| Q64674 | 20810 | SRM | 0.60810424 | 5.317E-05 |
| O54752 | 27419 | NAGLU | 0.607670487 | 0.0030619 |
| P27046 | 17158 | MAN2A1 | 0.607593898 | 0.0014198 |
| Q8CFX1 | 100198 | H6PD | 0.607224805 | 0.0008727 |
| Q3UJX2 | 66642 | CTNNBL1 | 0.606967672 | 4.418E-05 |
| P46664 | 11566 | ADSS | 0.606925059 | 0.0001456 |
| B1AT03 | 16882 | LIG3 | 0.606322151 | 0.0018725 |
| B1AXN9 | 110651 | RPS6KA3 | 0.60609817 | 0.0086995 |
| Q80UL3 | 14635 | GALK1 | 0.605803014 | 0.0003887 |
| Q8BP92 | 26611 | RCN2 | 0.605620741 | 2.673E-05 |
| Q3TUQ7 | 105787 | prkaa1 | 0.605406467 | 0.0003685 |
| Q3UK61 | 67041 | OXCT1 | 0.605290473 | 5.369E-07 |
| Q3USP3 | 78244 | DNAJC21 | 0.605203999 | 0.0009629 |
| Q9DCT2 | 68349 | NDUFS3 | 0.605176661 | 0.0051725 |
| P07356 | 12306 | ANXA2 | 0.604990268 | 1.391E-05 |
| A2AAN2 | 217337 | SRP68 | 0.6049199 | 0.0001043 |
| P46061 | 19387 | RANGAP1 | 0.604898744 | 0.0005711 |
| F8VPK0 | 218343 | TTC37 | 0.604749789 | 0.0098807 |
| Q99L13 | 58875 | HIBADH | 0.604641708 | 0.00247 |
| Q91VJ2 | 109042 | PRKCDBP | 0.604633348 | 0.0006007 |
| Q0P6B2 | 51886 | FUBP1 | 0.604558023 | 8.017E-05 |
| Q3UKP5 | 67439 | XAB2 | 0.604534077 | 0.0075565 |
| Q9ER72 | 27267 | CARS | 0.604361856 | 0.0007659 |
| Q9CQ60 | 66171 | PGLS | 0.604236614 | 0.0001167 |
| F8VPN4 | 77559 | AGL | 0.60394156 | 0.0045032 |
| G5E8R3 | 18563 | Pcx | 0.6037176 | 5.59E-06 |
| Q11011 | 19155 | NPEPPS | 0.603552984 | 0.0003132 |
| B1AZI6 | 331401 | THOC2 | 0.603426581 | 0.0003503 |
| P43247 | 17685 | MSH2 | 0.602900125 | 0.0001044 |
| Q9CZ44 | 386649 | NSFL1C | 0.60285399 | 7.344E-06 |
| Q5FWK3 | 228359 | ARHGAP1 | 0.602652022 | 0.0021868 |
| Q8BYA0 | 108903 | TBCD | 0.602561906 | 0.0003873 |
| Q91W50 | 229663 | CSDE1 | 0.602292808 | 7.662E-05 |
| Q8BXV2 | 76809 | BRI3BP | 0.602089177 | 0.007714 |
| O09005 | 13244 | DEGS1 | 0.602084461 | 0.0018617 |
| Q80U93 | 227720 | NUP214 | 0.601794196 | 0.0018307 |
| Q8BGC4 | 225791 | ZADH2 | 0.601520796 | 0.002802 |
| Q8BI84 | 338366 | MIA3 | 0.601416657 | 4.282E-05 |
| G5E850 | 109672 | CYB5A | 0.601412237 | 0.0049122 |
| Q3TSD4 | 107029 | ME2 | 0.601212818 | 0.0016554 |
| Q8BRF7 | 76983 | SCFD1 | 0.601074484 | 7.187E-05 |
| Q8BJ71 | 71805 | NUP93 | 0.600774216 | 0.0011282 |
| Q1W5W7 |  |  | 0.600688093 | 0.004165 |
| A2AN08 | 69116 | UBR4 | 0.600225305 | 0.0009205 |
| P28474 | 11532 | ADH5 | 0.600145843 | 0.0001513 |
| P68368 | 22145 | tuba4a | 0.600102244 | 3.578E-06 |
| Q7TSB1 | 234730 | FUK | 0.599849972 | 0.0023423 |
| Q80YR4 | 213753 | Zfp598 | 0.599689904 | 0.001367 |
| Q3UXD8 | 72508 | RPS6KB1 | 0.599399695 | 0.0040908 |
| Q9CQ75 | 17991 | NDUFA2 | 0.599331036 | 0.00187 |
| Q6ZPX7 |  |  | 0.599018782 | 1.667E-05 |
| Q61792 | 16796 | LASP1 | 0.598937038 | 0.0037193 |
| B2RQ83 | 216766 | GEMIN5 | 0.598554788 | 5.838E-06 |
| O35114 | 12492 | SCARB2 | 0.598067814 | 0.0023601 |
| B2RUG7 | 22763 | ZFR | 0.597518189 | 0.0014499 |
| G3X963 | 70472 | ATAD2 | 0.597430513 | 0.0014684 |
| I6L960 | 140630 | UBE4A | 0.597324604 | 0.0044553 |
| Q3ULJ0 | 333433 | GPD1L | 0.597010282 | 0.0005169 |
| Q8C5U8 |  |  | 0.596864811 | 0.0014212 |
| Q9CSZ8 | 67036 | MRPL45 | 0.596783872 | 0.0020391 |
| Q9JKY0 | 58184 | CNOT9 | 0.596274085 | 0.0011481 |
| Q8K3A8 | 57317 | SRSF4 | 0.59611063 | 0.0016544 |
| Q6P9T6 | 170760 | ACBD3 | 0.595620199 | 0.005877 |
| Q3UQM8 | 230027 | COQ3 | 0.595494823 | 0.0071107 |
| P61161 | 66713 | ACTR2 | 0.595361721 | 7.682E-05 |
| Q8BND3 | 74682 | WDR35 | 0.59526832 | 0.0003614 |
| P28352 | 11792 | APEX1 | 0.595246837 | 9.202E-06 |
| Q60931 | 22335 | VDAC3 | 0.595054971 | 0.0008238 |
| Q3UGS9 | 20480 | CLPB | 0.594932487 | 0.0001455 |
| Q9CRD2 | 66736 | EMC2 | 0.594898438 | 0.0041885 |
| P35293 | 19330 | RAB18 | 0.594690301 | 6.985E-06 |
| Q9CYL5 | 384009 | GLIPR2 | 0.594661686 | 4.271E-05 |
| E9QQ10 | 100986 | AKAP9 | 0.594590839 | 0.0082587 |
| Q9JKB1 | 50933 | UCHL3 | 0.594502854 | 0.0003343 |
| Q8R0F6 | 67444 | ILKAP | 0.594266187 | 0.0010593 |
| Q64261 | 12571 | CDK6 | 0.594160266 | 0.0061634 |
| Q8R3U8 | 17164 | MAPKAPK2 | 0.59402777 | 0.0088282 |
| Q8K366 | 50912 | EXOSC10 | 0.59393365 | 0.0088838 |
| E9Q5I9 | 23997 | PSMD13 | 0.593877034 | 0.0009059 |
| Q5SF07 | 319765 | IGF2BP2 | 0.593875152 | 0.0015752 |
| E9QKZ2 | 226432 | IPO9 | 0.593490235 | 1.862E-05 |
| F6TBV1 | 67075 | MAGT1 | 0.593459204 | 1.541E-05 |
| Q8R323 | 69263 | RFC3 | 0.593438492 | 0.0009565 |
| P17918 | 18538 | PCNA | 0.593321829 | 8.745E-05 |
| A2A547 | 19921 | RPL19 | 0.593063767 | 0.0005417 |
| Q5RL55 | 224742 | ABCF1 | 0.592980563 | 1.939E-05 |
| Q8K2Z4 | 68298 | NCAPD2 | 0.591738611 | 3.607E-05 |
| Q8JZM0 | 224481 | TFB1M | 0.591536767 | 0.0080106 |
| A0A087WNV1 | 15463 | AGFG1 | 0.591451311 | 0.0003451 |
| Q61206 | 18475 | PAFAH1B2 | 0.591425408 | 0.0031397 |
| Q8CB77 | 27224 | TCEB3 | 0.591374538 | 0.0051654 |
| Q9CQ18 | 68209 | rnaseh2c | 0.591198239 | 3.064E-05 |
| A0A0A6YVU8 | 100043022 | Gm9774 | 0.591016801 | 9.354E-05 |
| Q3THI5 | 19182 | PSMC3 | 0.590694535 | 0.0005051 |
| Q05816 | 16592 | FABP5 | 0.590496306 | 9.509E-05 |
| G3UVV4 | 15275 | HK1 | 0.590462126 | 0.0014471 |
| Q3UGL3 | 216705 | CLINT1 | 0.590445821 | 0.0004051 |
| Q9CYN2 | 66624 | SPCS2 | 0.590444269 | 0.0022456 |
| Q91Z49 | 69823 | FYTTD1 | 0.590347433 | 0.0039461 |
| Q3TJ52 | 19359 | RAD23B | 0.59001474 | 9.287E-05 |
| Q8BML9 | 97541 | QARS | 0.58998189 | 0.0001551 |
| O54984 | 56495 | ASNA1 | 0.589927431 | 0.0002895 |
| E9QN31 | 110109 | NOP2 | 0.589638407 | 0.0018341 |
| Q8BHC4 | 68087 | DCAKD | 0.589500917 | 0.0002284 |
| P24527 | 16993 | LTA4H | 0.589357656 | 0.0024383 |
| Q505F5 | 72946 | LRRC47 | 0.589170138 | 5.976E-05 |
| Q6R891 | 217124 | PPP1R9B | 0.589067751 | 0.0031523 |
| A2ARF6 | 11639 | AK4 | 0.588894649 | 0.0038406 |
| Q9CWJ9 | 108147 | ATIC | 0.588589685 | 0.0002714 |
| Q9ESY9 | 65972 | IFI30 | 0.588478466 | 0.0067808 |
| O08582 | 14904 | GTPBP1 | 0.588403335 | 0.0011663 |
| A0A068BGR9 | 66416 | NDUFA7 | 0.587849516 | 0.0025446 |
| Q9ERA0 | 21422 | tfcp2 | 0.58764841 | 0.0097185 |
| Q3UDE2 | 223723 | TTLL12 | 0.587542761 | 1.8E-06 |
| D3YVX4 | 98366 | SMAP1 | 0.58745892 | 0.0007499 |
| B7ZWI2 | 66118 | SARNP | 0.587118186 | 0.0093797 |
| Q9CQJ4 | 19821 | RNF2 | 0.58683254 | 0.0001478 |
| E9QL31 | 13132 | DAB2 | 0.586659447 | 0.0010403 |
| E9Q035 |  |  | 0.586495792 | 0.0002509 |
| E9QAI5 | 69719 | CAD | 0.5863684 | 9.57E-06 |
| E9Q6A3 | 20411 | sorbs1 | 0.58628595 | 0.0074541 |
| G5E896 | 234699 | EDC4 | 0.586269671 | 0.0020219 |
| Q8C586 | 19663 | RBPMS | 0.586201125 | 0.0009749 |
| Q8CDZ5 | 234865 | NUP133 | 0.586178382 | 0.0008428 |
| Q3UAS4 | 19156 | PSAP | 0.586131577 | 3.42E-05 |
| Q6P9Q4 | 234686 | FHOD1 | 0.586034804 | 0.0005537 |
| Q3TCE7 | 11867 | ARPC1B | 0.585652713 | 0.0005909 |
| Q8BNU0 | 76813 | ARMC6 | 0.585422332 | 0.0058829 |
| Q3TEZ2 | 83669 | wdr6 | 0.585103459 | 0.0004444 |
| Q8CCJ3 | 67490 | UFL1 | 0.58489027 | 0.0001575 |
| Q8QZY1 | 223691 | EIF3L | 0.584710817 | 0.0001044 |
| Q3TPD9 | 17161 | MAOA | 0.584203965 | 0.0011529 |
| Q3THK7 | 229363 | GMPS | 0.584044364 | 0.0003503 |
| Q921E2 | 106572 | RAB31 | 0.583966687 | 6.976E-05 |
| Q9DCL9 | 67054 | PAICS | 0.583812245 | 9.966E-05 |
| F8VQJ3 | 226519 | LAMC1 | 0.583689075 | 0.0001416 |
| Q8BK57 | 217715 | EIF2B2 | 0.583428474 | 0.0002828 |
| P61924 | 56447 | COPZ1 | 0.582759493 | 0.0005152 |
| Q9CX86 | 77134 | HNRNPA0 | 0.582586901 | 0.0014014 |
| G5E829 | 67972 | ATP2B1 | 0.582321114 | 0.0001575 |
| Q7TMB8 | 20430 | CYFIP1 | 0.582036894 | 2.188E-05 |
| Q9D8W6 | 56248 | AK3 | 0.581833263 | 0.0054259 |
| Q9DCT8 | 68337 | CRIP2 | 0.581806425 | 0.0045414 |
| A2AEB5 | 56397 | MORF4L2 | 0.581475768 | 0.0093932 |
| E9Q6R3 | 20333 | SEC22B | 0.581372155 | 0.0010228 |
| Q05DI7 | 17826 | FAM89B | 0.581355546 | 0.0057351 |
| G3X926 | 67239 | RPF2 | 0.581248842 | 7.926E-05 |
| Q07797 | 19039 | LGALS3BP | 0.581013942 | 0.0035756 |
| E9PUD2 | 74006 | DNM1L | 0.581003973 | 0.0013186 |
| Q5XG71 | 70683 | UTP20 | 0.580458625 | 0.0002506 |
| P60335 | 23983 | PCBP1 | 0.580151054 | 1.592E-06 |
| B9EKJ3 | 67074 | MON2 | 0.580125325 | 0.0001269 |
| K3W4Q8 | 12215 | BSG | 0.579991569 | 2.723E-06 |
| Q9D6Y7 | 110265 | MSRA | 0.57997583 | 5.577E-05 |
| Q8K2H2 | 72201 | OTUD6B | 0.579942271 | 3.752E-05 |
| Q7TPY3 | 18194 | NSDHL | 0.579470967 | 0.0015684 |
| Q3U6F1 | 19664 | RBPJ | 0.579348532 | 0.0014999 |
| A0A075DC90 | 17709 | COX2 | 0.579193479 | 0.0067275 |
| Q01730 | 20163 | RSU1 | 0.579145035 | 0.0002677 |
| M0QWY0 | 18117 | EMC8 | 0.579061832 | 0.0003116 |
| Q3URS9 | 66658 | CCDC51 | 0.578656912 | 0.0043804 |
| Q91ZX7 | 16971 | LRP1 | 0.578454655 | 0.000125 |
| Q3TSZ4 | 319625 | GALM | 0.578256443 | 0.0004423 |
| D3Z645 | 56433 | VPS29 | 0.578226159 | 0.0039381 |
| P57759 | 67397 | ERP29 | 0.57813708 | 0.0026239 |
| A2AW05 | 20833 | SSRP1 | 0.577864105 | 7.997E-05 |
| Q3U4W8 | 22225 | USP5 | 0.57733693 | 0.0001532 |
| Q3U026 | 57377 | MOGS | 0.577194189 | 0.0004032 |
| Q3U6Q1 | 60321 | WBP11 | 0.577190906 | 0.0051979 |
| P26039 | 21894 | TLN1 | 0.577185917 | 7.102E-05 |
| G3X9L6 | 100039281 | Gm10250 | 0.577121149 | 0.0015931 |
| Q6PB52 | 16976 | LRPAP1 | 0.576995951 | 0.0007097 |
| P49312 | 15382 | HNRNPA1 | 0.576850564 | 5.853E-05 |
| Q3UX26 | 68083 | PAK1IP1 | 0.576675857 | 1.111E-05 |
| Q62376 | 20637 | SNRNP70 | 0.576583141 | 0.0009819 |
| Q99LC5 | 110842 | ETFA | 0.576556603 | 4.566E-06 |
| P52480 | 18746 | PKM | 0.576527382 | 2.978E-06 |
| F8WIB1 | 104303 | ARL1 | 0.576312621 | 0.001199 |
| N0E4C0 | 13001 | CSNK2B | 0.576259916 | 0.0010631 |
| A1A596 | 110816 | PWP2 | 0.57623056 | 0.0002927 |
| Q3U3I6 |  |  | 0.576199459 | 0.0005833 |
| O88967 | 27377 | YME1L1 | 0.576047541 | 0.0001051 |
| P28660 | 50884 | NCKAP1 | 0.576017355 | 0.0002877 |
| Q9EPU0 | 19704 | UPF1 | 0.575981252 | 0.0008569 |
| H3BLJ9 | 13885 | ESD | 0.575545648 | 0.0002346 |
| Q8R436 |  |  | 0.57553589 | 4.338E-05 |
| P38060 | 15356 | HMGCL | 0.575531879 | 0.0031314 |
| P10833 | 20130 | RRAS | 0.575450564 | 9.432E-06 |
| Q8K297 | 234407 | COLGALT1 | 0.575347576 | 0.0009495 |
| Q9CW46 | 71766 | RAVER1 | 0.575319236 | 0.0036484 |
| Q9D1A2 | 66054 | CNDP2 | 0.575238724 | 5.249E-05 |
| A0PJ90 | 53312 | NUB1 | 0.575191345 | 0.0007288 |
| G5E8R1 | 22003 | TPM1 | 0.575168394 | 0.0001535 |
| Q6P5D8 | 74355 | SMCHD1 | 0.575018463 | 0.000355 |
| Q80SW1 | 229709 | AHCYL1 | 0.574988238 | 0.0005176 |
| P17182 | 13806 | ENO1 | 0.574810222 | 1.701E-06 |
| Q5F2E7 | 68564 | NUFIP2 | 0.574767872 | 0.0002213 |
| Q8BH64 | 259300 | EHD2 | 0.574723776 | 0.000781 |
| F6ZQA3 | 101706 | NUMA1 | 0.574479181 | 0.0098973 |
| D3YW87 |  |  | 0.574421506 | 6.481E-05 |
| Q61210 | 16801 | arhgef1 | 0.574390378 | 0.0001888 |
| P62880 | 14693 | GNB2 | 0.57431835 | 0.0030598 |
| Q8R509 | 19205 | PTBP1 | 0.574223316 | 3.207E-05 |
| E9QP46 | 319565 | SYNE2 | 0.574107301 | 0.0008014 |
| Q3U5L3 | 67804 | SNX2 | 0.574082771 | 0.0003193 |
| Q0VGB7 | 232314 | PPP4R2 | 0.573790766 | 0.0030567 |
| Q3TBQ3 | 73744 | MAN2C1 | 0.573764123 | 0.0090077 |
| Q8K0D5 | 28030 | GFM1 | 0.573665632 | 0.0002758 |
| Q9DD06 | 71660 | RARRES2 | 0.572981217 | 0.0060163 |
| Q3TZZ7 | 52635 | ESYT2 | 0.572386389 | 0.0001492 |
| B8X349 | 70834 | SPAG9 | 0.571986625 | 0.0005408 |
| E9Q586 | 13191 | DCTN1 | 0.571874103 | 1.798E-05 |
| P24547 | 23918 | IMPDH2 | 0.57167641 | 7.971E-06 |
| E9Q577 | 20184 | UIMC1 | 0.571660148 | 0.0062137 |
| Q80U72 | 105782 | SCRIB | 0.571428603 | 0.0011604 |
| Q3ULT8 | 18034 | NFKB2 | 0.571349614 | 0.0015479 |
| Q8R0W0 | 223650 | EPPK1 | 0.571243688 | 3.049E-05 |
| Q9WUM5 | 56451 | SUCLG1 | 0.571134826 | 0.0008402 |
| O35344 | 16648 | KPNA3 | 0.570794494 | 0.0004315 |
| G3UVU9 | 107817 | JMJD6 | 0.570596222 | 0.0007531 |
| P08228 | 20655 | SOD1 | 0.570568008 | 5.338E-05 |
| Q3TAD4 | 107652 | UAP1 | 0.5705093 | 0.0016192 |
| Q99NB8 | 94232 | UBQLN4 | 0.57045853 | 0.0024761 |
| Q9WVA4 | 21346 | TAGLN2 | 0.570225252 | 0.0005288 |
| Q8BU31 | 72065 | RAP2C | 0.569911 | 0.0010263 |
| Q3UYV9 | 433702 | NCBP1 | 0.569905789 | 0.0096166 |
| P99026 | 19172 | PSMB4 | 0.569846104 | 0.0019386 |
| Q9DBZ5 | 73830 | EIF3K | 0.5697301 | 0.0022777 |
| Q8BSX8 | 68018 | COL4A3BP | 0.569639145 | 0.0014168 |
| Q3THP1 | 75221 | DPP3 | 0.569585219 | 0.0001015 |
| D3YW20 | 71517 | 9030624J02Rik | 0.569536353 | 0.0043415 |
| O89054 | 11461 | ACTB | 0.569404438 | 0.0020976 |
| P16858 | 14433 | GAPDH | 0.569392553 | 5.106E-05 |
| D3YWT0 | 56529 | SEC11A | 0.569370693 | 0.0001222 |
| Q8C129 | 240028 | LNPEP | 0.56934534 | 0.0018618 |
| F6ZDS4 | 108989 | TPR | 0.568882416 | 0.0001312 |
| E9Q7G0 | 101706 | NUMA1 | 0.568648272 | 0.0001486 |
| E9QP59 | 380664 | lemd3 | 0.568571482 | 0.0013476 |
| P10518 | 17025 | ALAD | 0.568564564 | 0.0007396 |
| Q3TDN2 | 76577 | FAF2 | 0.568251808 | 5.643E-05 |
| P23591 | 22122 | TSTA3 | 0.568226512 | 0.0007406 |
| Q01320 | 21973 | TOP2A | 0.568163858 | 3.11E-05 |
| B2M1R7 | 18521 | PCBP2 | 0.568135993 | 0.0078082 |
| H3BJU7 | 16800 | ARHGEF2 | 0.568050875 | 0.0071837 |
| Q9R1P1 | 26446 | PSMB3 | 0.567588004 | 0.0001922 |
| P51125 | 12380 | CAST | 0.567526654 | 0.0001131 |
| A0A068BIT8 | 16913 | PSMB8 | 0.567412356 | 0.0010178 |
| Q9CRC8 | 67144 | LRRC40 | 0.567404782 | 0.000204 |
| Q60932 | 22333 | VDAC1 | 0.56697142 | 5.281E-05 |
| Q9CQ48 | 52653 | NUDCD2 | 0.566845943 | 0.0012657 |
| Q3UGC1 | 229707 | STRIP1 | 0.566771753 | 0.0002396 |
| Q3V3R1 | 270685 | MTHFD1L | 0.566637865 | 0.000613 |
| A0JLN6 | 213773 | TBL3 | 0.566336992 | 3.227E-05 |
| Q3UEB3 | 67959 | PUF60 | 0.566246975 | 0.0035082 |
| Q8BGS0 | 67920 | MAK16 | 0.566085133 | 0.0009871 |
| Z4YLI8 | 74148 | CLUH | 0.565955626 | 0.0008646 |
| Q9QUR6 | 19072 | PREP | 0.565929002 | 6.432E-06 |
| P26040 | 22350 | EZR | 0.565899708 | 0.0025277 |
| Q9QZB7 | 56444 | ACTR10 | 0.565755274 | 0.0080204 |
| Q9D1N9 | 353242 | MRPL21 | 0.565593494 | 0.0039751 |
| Q8R2Y2 | 84004 | MCAM | 0.565235172 | 0.0072186 |
| P48771 | 12866 | COX7A2 | 0.565187101 | 0.0032159 |
| Q9WUM3 | 23789 | CORO1B | 0.565144484 | 5.002E-05 |
| E9QAT4 | 227648 | SEC16A | 0.565133695 | 0.0042159 |
| P56480 | 11947 | ATP5B | 0.564935968 | 4.783E-06 |
| Q6PF96 | 66841 | ETFDH | 0.564816205 | 0.0034376 |
| D3YVV4 | 66848 | FUCA2 | 0.564685812 | 0.0013382 |
| Q3UPG1 | 15288 | HMBS | 0.56454976 | 0.0033755 |
| Q3TFD0 | 108037 | SHMT2 | 0.56447476 | 1.363E-05 |
| Q3TF87 | 226414 | DARS | 0.56418281 | 1.112E-05 |
| P97310 | 17216 | MCM2 | 0.564148412 | 0.000139 |
| Q9DB73 | 72017 | CYB5R1 | 0.564142848 | 0.0031211 |
| P19157 | 14870 | GSTP1 | 0.564078823 | 2.767E-05 |
| O88569 | 53379 | HNRNPA2B1 | 0.564044569 | 1.794E-06 |
| F8WHM5 | 20340 | GLG1 | 0.56388285 | 0.0084081 |
| Q8VED9 | 216551 | LGALSL | 0.563857448 | 1.266E-05 |
| Q9CSU0 | 70470 | RPRD1B | 0.563743963 | 0.0005375 |
| Q03265 | 11946 | ATP5A1 | 0.563708981 | 3.313E-05 |
| D3YWT1 | 432467 | HNRNPH3 | 0.563708128 | 0.0080917 |
| Q9D0T1 | 20826 | NHP2L1 | 0.563700825 | 0.0012907 |
| F8VPK5 | 19878 | ROCK2 | 0.563689061 | 0.0019115 |
| Q7TT04 | 69912 | NUP43 | 0.563349465 | 0.0037535 |
| O70503 | 56348 | HSD17B12 | 0.563261906 | 0.0004998 |
| P42208 | 18000 | SEPT2 | 0.562690811 | 4.836E-05 |
| Q91YZ2 | 13017 | CTBP2 | 0.562665807 | 0.0024556 |
| Q7TMF3 | 66414 | NDUFA12 | 0.562653321 | 0.0088418 |
| Q8BMJ2 | 107045 | LARS | 0.562543386 | 0.0003603 |
| G3UYV7 | 54127 | RPS28 | 0.562321954 | 0.000137 |
| Q8C166 | 266692 | CPNE1 | 0.562151143 | 0.0002581 |
| Q8CEG3 | 213895 | BMS1 | 0.562105955 | 0.00339 |
| Q9CY64 | 109778 | BLVRA | 0.561986042 | 0.0001066 |
| Q3UGR5 | 76987 | HDHD2 | 0.561829875 | 0.0005356 |
| Q922D8 | 108156 | MTHFD1 | 0.561558295 | 3.706E-06 |
| Q9Z2M7 | 54128 | PMM2 | 0.561206985 | 0.0013602 |
| Q3TKC5 | 231327 | PPAT | 0.560825035 | 0.0026762 |
| Q8R395 | 66398 | COMMD5 | 0.560668539 | 0.0033913 |
| A1A4T2 | 14376 | GANAB | 0.560603809 | 4.172E-05 |
| Q61191 | 15161 | HCFC1 | 0.560265508 | 0.0011039 |
| Q62318 | 21849 | TRIM28 | 0.560215738 | 5.981E-06 |
| Q9CPN8 | 140488 | IGF2BP3 | 0.560203765 | 0.0014122 |
| Q9CQH7 | 70533 | BTF3L4 | 0.560191505 | 4.636E-05 |
| F7ACR9 | 11426 | MACF1 | 0.56016896 | 0.0001188 |
| P14115 | 26451 | RPL27A | 0.559802524 | 0.0001776 |
| Q3TAV1 | 93695 | GPNMB | 0.559778456 | 0.0001561 |
| E9Q197 | 67201 | GLOD4 | 0.559751191 | 0.0004601 |
| E9Q1S3 | 20334 | SEC23A | 0.559737123 | 7.914E-05 |
| P47753 | 12340 | CAPZA1 | 0.559636464 | 3.598E-05 |
| Q80X85 | 50529 | MRPS7 | 0.559452244 | 0.0013767 |
| Q91XD6 | 70160 | VPS36 | 0.559391696 | 0.0054116 |
| Q60715 | 18451 | P4HA1 | 0.559234293 | 0.0029613 |
| P46471 | 19181 | PSMC2 | 0.5592271 | 5.274E-05 |
| Q61213 |  |  | 0.559157871 | 9.393E-05 |
| P97855 | 27041 | G3BP1 | 0.5591532 | 0.0002743 |
| Q3UZI6 | 57741 | NOC2L | 0.558995265 | 0.0004803 |
| Q8R464 | 260299 | CADM4 | 0.558734171 | 0.0083835 |
| Q922R9 | 68015 | TRAP1 | 0.558733484 | 4.726E-05 |
| Q9CRD0 | 68095 | OCIAD1 | 0.558577086 | 7.98E-05 |
| Q91Z53 | 76238 | GRHPR | 0.558568717 | 4.774E-06 |
| Q91VH6 | 76890 | MEMO1 | 0.558291592 | 0.0003649 |
| E9Q390 | 226101 | MYOF | 0.558228714 | 3.141E-07 |
| Q8C605 | 56421 | PFKP | 0.558223725 | 4.003E-06 |
| Q8VDN2 | 11928 | ATP1A1 | 0.557995683 | 8.495E-05 |
| F7BTZ2 | 18854 | PML | 0.557978454 | 0.0005131 |
| Q8R086 | 211389 | SUOX | 0.557842679 | 0.0010285 |
| O54941 | 57376 | SMARCE1 | 0.557767336 | 0.0034486 |
| Q9DBG5 | 66905 | PLIN3 | 0.557677671 | 0.0059785 |
| P84096 | 56212 | RHOG | 0.557654035 | 0.0011071 |
| Q80YG4 | 140740 | SEC63 | 0.557550174 | 0.0002822 |
| A2ADY9 | 68817 | DDI2 | 0.557401974 | 0.001759 |
| P35505 | 14085 | FAH | 0.557396203 | 4.119E-05 |
| E9Q852 | 17356 | AFDN | 0.557308453 | 0.0001662 |
| Q3U3S0 | 97387 | STRN4 | 0.557299247 | 0.00971 |
| Q9JMH6 | 50493 | TXNRD1 | 0.55717987 | 0.0001942 |
| Q9CPY7 | 66988 | LAP3 | 0.557172843 | 1.246E-05 |
| A2AWT6 | 21429 | UBTF | 0.557117331 | 0.0001284 |
| E9Q310 | 14815 | NR3C1 | 0.556928411 | 0.0069786 |
| Q8CI95 | 106326 | OSBPL11 | 0.556712814 | 0.0087748 |
| Q8CCG5 | 64143 | RALB | 0.556610284 | 0.0031646 |
| Q3TW01 | 67844 | RAB32 | 0.556521837 | 0.0011697 |
| Q8CH25 | 66660 | SLTM | 0.556415358 | 0.0039237 |
| Q9DC69 | 66108 | NDUFA9 | 0.556336646 | 0.0005318 |
| Q9JMD0 | 22680 | Zfp207 | 0.556204678 | 0.0002124 |
| P14152 | 17449 | MDH1 | 0.556168014 | 3.608E-07 |
| Q9EP83 | 27756 | LSM2 | 0.556069623 | 0.0011564 |
| Q3UIH7 | 56187 | RABGGTA | 0.556021264 | 0.0046603 |
| Q8BJY1 | 66998 | PSMD5 | 0.55585432 | 0.0005332 |
| Q91V89 | 21770 | PPP2R5D | 0.555814098 | 0.0016721 |
| Q3UJ70 | 208715 | HMGCS1 | 0.555801446 | 0.0034137 |
| Q61553 | 14086 | FSCN1 | 0.555776374 | 1.064E-05 |
| Q3TH64 | 66427 | CYB5B | 0.555639379 | 0.0029863 |
| Q5SUF2 | 67684 | LUC7L3 | 0.555577414 | 0.0001412 |
| O08759 | 22215 | UBE3A | 0.555571447 | 0.0024027 |
| Q91VW3 | 73723 | SH3BGRL3 | 0.555549197 | 0.0079784 |
| Q69ZJ6 | 19221 | PTGFRN | 0.555304861 | 0.0064283 |
| Q9QZL0 | 56532 | ripk3 | 0.555279394 | 0.0006832 |
| B1AT10 | 76626 | MSI2 | 0.555252592 | 0.0001658 |
| Q9DB77 | 67003 | Uqcrc2 | 0.554983486 | 1.23E-05 |
| Q99JB2 | 66592 | STOML2 | 0.554876291 | 0.0003216 |
| O08529 | 12334 | CAPN2 | 0.554837215 | 1.013E-06 |
| Q5BKS2 | 19043 | PPM1B | 0.55467745 | 0.0041357 |
| A0A068F126 |  |  | 0.554667154 | 0.0027574 |
| Q9Z2L6 | 17330 | MINPP1 | 0.554662102 | 0.0004378 |
| P35762 | 12520 | CD81 | 0.554580616 | 0.0012681 |
| Q80YW9 | 338355 | FKBP15 | 0.554502302 | 9.1E-06 |
| Q3UZ39 | 16978 | LRRFIP1 | 0.554354604 | 0.0001314 |
| Q8C4J7 | 213773 | TBL3 | 0.554177979 | 0.0075876 |
| Q3UD67 | 234734 | AARS | 0.554143123 | 3.327E-05 |
| P60710 | 11461 | ACTB | 0.553800158 | 2.676E-06 |
| Q3TWV4 | 11773 | AP2M1 | 0.553707125 | 0.0090595 |
| Q9D880 | 66525 | TIMM50 | 0.553387665 | 0.0002647 |
| Q9CSH3 | 72662 | dis3 | 0.552969006 | 0.0014773 |
| B9EHJ3 | 21872 | TJP1 | 0.552919369 | 0.0001516 |
| Q9QZE5 | 54161 | COPG1 | 0.552833652 | 1.218E-05 |
| P21981 | 21817 | TGM2 | 0.552723626 | 4.414E-06 |
| O70569 |  |  | 0.552600273 | 8.471E-05 |
| P30416 | 14228 | FKBP4 | 0.552580369 | 2.851E-06 |
| Q8BGH2 | 68653 | SAMM50 | 0.552539284 | 0.0008975 |
| O09061 | 19170 | PSMB1 | 0.55239647 | 0.0002764 |
| Q8BL36 | 72722 | FAM98A | 0.552108791 | 8.002E-05 |
| O88544 | 26891 | COPS4 | 0.552052005 | 2.804E-05 |
| Q99LI2 | 229725 | CLCC1 | 0.551795484 | 0.0005256 |
| Q3UDQ7 | 69597 | AFG3L2 | 0.551770489 | 0.0045663 |
| Q9EQQ9 | 76055 | MGEA5 | 0.551615854 | 0.006239 |
| P54103 | 22791 | DNAJC2 | 0.551504835 | 0.0009057 |
| Q3UM45 | 66385 | PPP1R7 | 0.551151682 | 0.0002659 |
| A0A068BGU5 | 21355 | tap2 | 0.550929234 | 4.004E-06 |
| Q8BHD7 | 230257 | PTBP3 | 0.55074141 | 0.0051714 |
| Q8BP60 |  |  | 0.550642129 | 0.0039361 |
| Q5SSI6 | 217109 | UTP18 | 0.550580148 | 0.0003089 |
| Q9QZ23 | 56748 | NFU1 | 0.550494955 | 0.001067 |
| P63168 | 56455 | DYNLL1 | 0.550487451 | 4.081E-05 |
| Q8BMF4 | 235339 | DLAT | 0.550186747 | 0.0001154 |
| P26041 | 17698 | MSN | 0.549969562 | 8.909E-05 |
| Q8K265 | 216156 | WDR18 | 0.549893571 | 0.0051929 |
| Q9Z0P5 | 23999 | TWF2 | 0.549868678 | 0.0031889 |
| Q9D1D4 | 68581 | TMED10 | 0.549640978 | 2.297E-05 |
| A4FUW1 | 74370 | RPTOR | 0.549377298 | 0.0011736 |
| Q7TPR4 | 109711 | ACTN1 | 0.549343921 | 2.067E-05 |
| P27612 | 18786 | PLAA | 0.549279073 | 2.332E-07 |
| Q80X90 | 286940 | FLNB | 0.549189055 | 2.347E-05 |
| Q8BLY2 | 272396 | TARSL2 | 0.549173676 | 0.0012569 |
| Q3UY05 | 225887 | NDUFS8 | 0.549167563 | 0.0038668 |
| Q6ZPE2 | 77980 | SBF1 | 0.549128923 | 9.167E-05 |
| Q3UUG1 | 216965 | TAOK1 | 0.548796961 | 0.0043792 |
| Q9D2G2 | 78920 | DLST | 0.548565317 | 7.822E-05 |
| Q9EPE9 | 170759 | ATP13A1 | 0.548353302 | 0.0004597 |
| P97429 | 11746 | ANXA4 | 0.548212167 | 0.000251 |
| A2A5R2 | 99371 | ARFGEF2 | 0.548055454 | 0.000142 |
| Q9Z1Z0 | 56041 | USO1 | 0.547981828 | 1.66E-06 |
| Q497Z1 | 217333 | TRIM47 | 0.547866928 | 1.05E-05 |
| Q9DCR2 | 11777 | AP3S1 | 0.54773771 | 0.0007192 |
| Q3UNN4 | 20588 | SMARCC1 | 0.547723393 | 0.0002989 |
| Q8C1X9 | 11745 | ANXA3 | 0.547685818 | 6.227E-05 |
| D3Z0F5 | 26893 | COPS6 | 0.547604597 | 0.0019331 |
| Q3UH60 | 239667 | DIP2B | 0.547567839 | 0.0029251 |
| Q8VEE4 | 68275 | RPA1 | 0.547502975 | 2.259E-05 |
| Q3TIC8 | 22273 | UQCRC1 | 0.54744109 | 0.0001561 |
| Q3UJN6 | 17535 | mre11a | 0.5472887 | 0.0007795 |
| Q91W09 | 14151 | FECH | 0.547201435 | 3.595E-05 |
| Q9Z0W3 | 59015 | NUP160 | 0.547088684 | 0.0001165 |
| Q8VHY0 | 121021 | CSPG4 | 0.546755935 | 8.361E-05 |
| Q91WC0 | 52690 | SETD3 | 0.546249811 | 0.0049257 |
| Q3U1J4 | 13194 | DDB1 | 0.546131116 | 9.008E-06 |
| P48036 | 11747 | ANXA5 | 0.546063903 | 7.318E-06 |
| Q3UW66 | 246221 | MPST | 0.545836295 | 0.0001689 |
| Q99J62 | 106344 | RFC4 | 0.545808155 | 0.0002403 |
| Q9DCM0 | 66071 | ETHE1 | 0.545780735 | 3.289E-05 |
| Q3TGI9 | 66409 | RSL1D1 | 0.545665677 | 0.0003472 |
| Q3UXP2 | 20174 | RUVBL2 | 0.54548599 | 1.367E-06 |
| Q8K183 | 216134 | PDXK | 0.545458612 | 0.0081297 |
| Q8R326 | 66645 | PSPC1 | 0.54545526 | 2.128E-05 |
| Q3UDI8 | 17220 | MCM7 | 0.54524079 | 0.0001764 |
| Q8BY87 | 74996 | USP47 | 0.545071826 | 4.083E-05 |
| Q8CIG8 | 27374 | PRMT5 | 0.544825148 | 0.0021035 |
| Q91V01 | 14792 | LPCAT3 | 0.54481399 | 0.0059621 |
| P97452 | 12181 | BOP1 | 0.544662571 | 0.0011318 |
| P54116 | 13830 | STOM | 0.54463862 | 0.0001639 |
| P63017 | 15481 | HSPA8 | 0.544536005 | 1.915E-07 |
| Q8BWM0 | 96979 | PTGES2 | 0.544374502 | 3.232E-06 |
| Q8K0C9 | 218138 | GMDS | 0.544364884 | 0.0002577 |
| Q5RKP0 | 26949 | VAT1 | 0.544227279 | 4.829E-05 |
| Q8CE96 | 66926 | TRMT6 | 0.544174243 | 0.0001294 |
| B9EHZ5 | 56524 | MPP6 | 0.543987581 | 3.889E-05 |
| O08847 | 20020 | POLR2A | 0.543638899 | 4.85E-05 |
| P62192 | 19179 | PSMC1 | 0.543330908 | 0.0005051 |
| B1AY13 | 329908 | USP24 | 0.543095501 | 0.0003906 |
| P17439 | 14466 | GBA | 0.543073022 | 0.0005281 |
| D6RHA2 | 19703 | RENBP | 0.54279546 | 0.0017751 |
| A0A0B4J1F2 |  |  | 0.54277223 | 0.0002285 |
| Q9EP82 | 57773 | WDR4 | 0.542352493 | 0.0001653 |
| Q61753 | 236539 | PHGDH | 0.542311933 | 2.522E-05 |
| Q9D338 | 56284 | MRPL19 | 0.542172501 | 0.0046897 |
| P70677 | 12367 | CASP3 | 0.542143222 | 5.268E-05 |
| P70444 | 12122 | BID | 0.542053217 | 0.005222 |
| Q3TV90 | 66978 | LUC7L | 0.541943503 | 0.0059344 |
| O55234 | 19173 | PSMB5 | 0.541340796 | 0.0002688 |
| Q7TMY8 | 59026 | HUWE1 | 0.541274275 | 0.000119 |
| Q3TFE8 | 16211 | KPNB1 | 0.54113861 | 3.365E-06 |
| P13439 | 22247 | UMPS | 0.541137799 | 0.0006886 |
| P60670 | 217365 | NPLOC4 | 0.541064767 | 0.0004047 |
| P57780 | 60595 | ACTN4 | 0.540852054 | 4.737E-05 |
| E0CXN5 | 14555 | GPD1 | 0.540513986 | 1.511E-05 |
| P70399 | 27223 | Trp53bp1 | 0.540063506 | 0.0013148 |
| Q06EZ3 | 20652 | SOAT1 | 0.539882503 | 0.0012935 |
| Q91VR8 | 101314 | BRK1 | 0.539847328 | 0.0002339 |
| P19783 | 12857 | Cox4I1 | 0.539775508 | 3.7E-05 |
| Q7TQE2 | 22793 | ZYX | 0.539769876 | 0.0032499 |
| Q99LF5 | 17436 | ME1 | 0.539624363 | 3.784E-05 |
| P70372 | 15568 | ELAVL1 | 0.539623451 | 0.0001356 |
| Q9CPW4 | 67771 | ARPC5 | 0.539552279 | 0.0094083 |
| Q6P4S8 | 68510 | INTS1 | 0.539498063 | 0.0003537 |
| E9PWY9 | 66590 | FARSA | 0.539283716 | 0.0001145 |
| Q6A022 |  |  | 0.539185813 | 0.0007712 |
| F6VG18 | 93730 | LZTFL1 | 0.539094421 | 0.0096587 |
| P51150 | 19349 | Rab7 | 0.539072291 | 6.007E-07 |
| O70194 | 55944 | EIF3D | 0.538959779 | 0.0001559 |
| Q3TWP9 | 98878 | EHD4 | 0.538771383 | 0.0002924 |
| Q8K2B3 | 66945 | SDHA | 0.538729306 | 5.684E-05 |
| B2RY56 | 67039 | RBM25 | 0.538709412 | 9.574E-05 |
| Q9EP69 | 83493 | SACM1L | 0.538530835 | 0.0011711 |
| O54825 | 53414 | BYSL | 0.538288652 | 0.0004644 |
| Q6PJ18 | 22004 | TPM2 | 0.538101453 | 0.0042555 |
| Q3TU36 | 229877 | RAP1GDS1 | 0.538030559 | 0.0011587 |
| O35887 | 12321 | CALU | 0.537997711 | 0.0003384 |
| Q922J3 | 56430 | CLIP1 | 0.537766617 | 0.0005831 |
| Q3UFY8 | 52575 | TRMT10C | 0.537727642 | 0.0003996 |
| E9Q933 | 216821 | TMEM11 | 0.537634796 | 0.0026652 |
| Q61166 | 13589 | MAPRE1 | 0.537592431 | 0.0001379 |
| P50544 | 11370 | ACADVL | 0.537554622 | 3.867E-05 |
| Q7TSC1 | 53761 | PRRC2A | 0.537447214 | 0.004128 |
| Q8K354 | 109857 | cbr3 | 0.537365465 | 6.415E-05 |
| A0A087WNP6 | 321022 | CDV3 | 0.537277811 | 0.0004532 |
| Q99LX0 | 57320 | PARK7 | 0.537265943 | 3.439E-05 |
| D3YTQ9 | 20054 | RPS15 | 0.537250237 | 0.0007867 |
| Q99N15 | 15108 | HSD17B10 | 0.537065425 | 2.349E-05 |
| P36552 | 12892 | CPOX | 0.536873987 | 0.0001415 |
| Q3U9R7 | 14782 | GSR | 0.536807584 | 0.0001067 |
| P54823 | 13209 | DDX6 | 0.536723504 | 1.48E-05 |
| Q6NS46 | 18572 | PDCD11 | 0.536649431 | 4.056E-05 |
| Q921T2 | 208263 | TOR1AIP1 | 0.536645528 | 0.001974 |
| Q9CXJ4 | 74610 | ABCB8 | 0.536383291 | 0.0054177 |
| Q3TTX0 | 17184 | MATR3 | 0.536152178 | 2.899E-05 |
| J3QQ30 | 18738 | pitpna | 0.535752837 | 0.0002994 |
| Q921X9 | 72599 | PDIA5 | 0.535579092 | 0.0032157 |
| E0CYE2 | 23917 | IMPDH1 | 0.53547553 | 6.507E-05 |
| B9EKP5 | 192176 | FLNA | 0.535288543 | 8.25E-07 |
| E9Q585 | 20534 | SLC4A1AP | 0.535113728 | 0.0030585 |
| P11499 | 15516 | HSP90AB1 | 0.53508932 | 2.046E-06 |
| P62806 | 69386 | Hist1h4h | 0.535006315 | 8.837E-07 |
| Q99KJ8 | 69654 | DCTN2 | 0.534937344 | 0.0007811 |
| U3RKD2 | 218756 | SLC4A7 | 0.534871796 | 0.0015052 |
| Q3UW53 | 63913 | FAM129A | 0.534836987 | 0.0002658 |
| O08795 | 19089 | PRKCSH | 0.534831522 | 6.151E-05 |
| Q921L3 | 68944 | TMCO1 | 0.53482101 | 0.0013909 |
| Q0KL02 | 223435 | TRIO | 0.534788696 | 0.0013268 |
| Q8R2Y8 | 217057 | PTRH2 | 0.53470977 | 0.0008881 |
| Q9CQL6 | 66223 | MRPL35 | 0.534673774 | 0.0006446 |
| P58389 | 110854 | PTPA | 0.534590937 | 0.0003289 |
| Q61136 | 19134 | PRPF4B | 0.534445723 | 0.0007471 |
| A0A0A6YW52 |  |  | 0.534421001 | 0.0076479 |
| Q9D0F3 | 70361 | LMAN1 | 0.534234731 | 0.0001458 |
| Q04899 | 18557 | CDK18 | 0.534112219 | 0.0034512 |
| B2RUJ7 | 22436 | XDH | 0.533932967 | 3.455E-05 |
| O08810 | 20624 | EFTUD2 | 0.533902834 | 5.452E-06 |
| Q3V471 | 16854 | LGALS3 | 0.53364125 | 5.597E-06 |
| Q3TKU6 | 65114 | VPS35 | 0.533576322 | 2.569E-05 |
| Q3TGW0 | 74117 | ACTR3 | 0.533351229 | 4.267E-05 |
| Q6ZWX6 | 13665 | EIF2S1 | 0.533160829 | 4.427E-05 |
| Q6PGL7 | 28006 | Fam21 | 0.533089794 | 0.001165 |
| G5E884 | 18479 | PAK1 | 0.533077887 | 0.0069037 |
| P61027 | 19325 | RAB10 | 0.532919781 | 0.0001355 |
| J3QK23 |  |  | 0.53283752 | 6.704E-06 |
| Q3TIU7 | 227197 | NDUFS1 | 0.532801404 | 1.081E-05 |
| P80318 | 12462 | CCT3 | 0.532771806 | 1.667E-06 |
| Q9WU62 | 16319 | INCENP | 0.532691096 | 0.0004872 |
| P05202 | 14719 | GOT2 | 0.53217338 | 3.166E-06 |
| E9PZK7 | 76688 | ARFRP1 | 0.531896371 | 0.0090236 |
| A0A087WSP0 | 13518 | DST | 0.531863895 | 0.006602 |
| Q9CPT5 | 28126 | NOP16 | 0.531838927 | 5.145E-05 |
| Q91V47 |  |  | 0.531730718 | 0.0002152 |
| F6SQH7 | 67811 | poldip2 | 0.531676584 | 0.000895 |
| B2RWW6 | 231659 | Gcn1l1 | 0.531621144 | 0.0007389 |
| Q9ERK4 | 110750 | CSE1L | 0.53161167 | 7.57E-06 |
| Q62348 | 22099 | TSN | 0.531404993 | 0.0007929 |
| Q9D0S9 | 68917 | HINT2 | 0.530786269 | 0.0001379 |
| A2AQE4 | 12848 | COPS2 | 0.530464387 | 3.016E-05 |
| Q3U281 | 66397 | SAR1B | 0.530256315 | 5.881E-05 |
| Q3UVK0 | 226090 | ERMP1 | 0.529869423 | 0.0005298 |
| Q3TDF8 | 225363 | ETF1 | 0.529772201 | 0.0002078 |
| P00375 | 13361 | DHFR | 0.529763171 | 0.0007588 |
| P51174 | 11363 | ACADL | 0.529709844 | 1.376E-05 |
| Q9CR61 | 66916 | NDUFB7 | 0.529488429 | 0.0028442 |
| Q9DBJ1 | 18648 | PGAM1 | 0.529364952 | 7.316E-06 |
| Q924T2 | 118451 | MRPS2 | 0.529129677 | 0.0020046 |
| Q3TWN8 | 56454 | ALDH18A1 | 0.529118853 | 0.0001148 |
| O08738 | 12368 | CASP6 | 0.528981636 | 0.0006281 |
| Q3V117 | 104112 | ACLY | 0.528772 | 7.357E-06 |
| Q9ESU6 | 57261 | BRD4 | 0.528734195 | 0.0059562 |
| O54784 | 13144 | DAPK3 | 0.528654387 | 0.0002128 |
| B2RY51 | 109181 | TRIP11 | 0.528653779 | 6.158E-05 |
| Q3ULB1 | 21753 | TES | 0.528566393 | 0.002373 |
| P47754 | 12343 | CAPZA2 | 0.528488854 | 0.0001589 |
| Q7TMQ7 | 101240 | WDR91 | 0.528193041 | 0.0019794 |
| Q3UA06 | 69716 | TRIP13 | 0.528188858 | 0.0001596 |
| Q9DCU6 | 66163 | MRPL4 | 0.528120094 | 9.819E-05 |
| A0A0A6YWP9 | 98258 | TXNDC9 | 0.528111785 | 0.0028778 |
| P97390 | 22365 | VPS45 | 0.528079627 | 0.0011903 |
| P51863 | 11972 | ATP6V0D1 | 0.528042489 | 0.0024858 |
| Q9ERS2 | 67184 | NDUFA13 | 0.52766487 | 0.0011313 |
| P48678 | 16905 | LMNA | 0.527589578 | 7.563E-06 |
| Q925I1 | 108888 | ATAD3A | 0.527502419 | 0.0004319 |
| Q9D2R8 | 14548 | MRPS33 | 0.527469799 | 0.0009616 |
| Q9Z1Z2 | 20901 | STRAP | 0.52746494 | 0.0001161 |
| Q3UPH1 | 73137 | PRRC1 | 0.527258651 | 0.0018195 |
| P27048 | 20638 | SNRPB | 0.527167726 | 3.644E-05 |
| Q14AX9 | 17534 | mrc2 | 0.527093849 | 0.0034475 |
| Q02053 | 22201 | UBA1 | 0.527085189 | 1.056E-06 |
| D3Z0A2 |  |  | 0.52696501 | 2.248E-05 |
| G3X934 | 545085 | WDR70 | 0.526929182 | 0.0031967 |
| E9PWQ3 | 12835 | COL6A3 | 0.526773412 | 8.477E-05 |
| Q8BHL5 | 140579 | elmo2 | 0.526701615 | 0.000374 |
| Q63844 | 26417 | MAPK3 | 0.526566134 | 0.0022059 |
| Q80VI1 | 384309 | TRIM56 | 0.52647047 | 0.0007593 |
| Q3UN02 | 225010 | LCLAT1 | 0.526442895 | 0.0007032 |
| G3UXL2 | 328099 | Prps1l3 | 0.526392278 | 0.0003423 |
| Q9D8X2 | 234388 | CCDC124 | 0.52626236 | 0.00051 |
| P11438 | 16783 | LAMP1 | 0.526250241 | 0.0004931 |
| Q3TG58 | 22384 | EIF4H | 0.526213675 | 0.002739 |
| Q9CR25 | 67728 | DPH2 | 0.525947236 | 0.0001379 |
| Q3TA68 | 225348 | WDR36 | 0.525655398 | 0.0005856 |
| Q8VCQ8 | 109624 | CALD1 | 0.525442568 | 2.934E-05 |
| Q04750 | 21969 | TOP1 | 0.525250588 | 0.0001964 |
| P54276 | 17688 | MSH6 | 0.524967727 | 0.0001647 |
| D3Z5I1 | 78781 | ZC3HAV1 | 0.524737986 | 3.851E-05 |
| D3Z7C0 | 18100 | MRPL40 | 0.524615144 | 0.0001106 |
| Q3UAZ7 | 97165 | HMGB2 | 0.524441808 | 0.0067922 |
| Q8VBV7 | 108679 | COPS8 | 0.524409373 | 0.0046188 |
| P45376 | 11677 | Akr1b3 | 0.524402079 | 3.193E-06 |
| A7M7Q8 | 64656 | MRPS23 | 0.524385531 | 0.0036463 |
| Q91V12 | 70025 | ACOT7 | 0.524322406 | 3.945E-05 |
| Q6P6I4 | 103140 | GstT3 | 0.524294552 | 0.0013036 |
| O35075 | 13185 | DSCR3 | 0.524233063 | 0.0013032 |
| P60122 | 56505 | RUVBL1 | 0.524129767 | 3.17E-06 |
| P62918 | 26961 | RPL8 | 0.524111524 | 0.0010708 |
| P17427 | 11772 | AP2A2 | 0.524095282 | 0.0002251 |
| Q8VDP6 | 52858 | CDIPT | 0.524007569 | 0.0002448 |
| Q8BKC5 | 70572 | IPO5 | 0.523959242 | 4.411E-05 |
| A2AJ72 | 320267 | FUBP3 | 0.523792156 | 0.0002732 |
| E9PW15 | 52615 | SUZ12 | 0.523674619 | 0.0009889 |
| P99028 | 66576 | Uqcrh | 0.523581518 | 0.000223 |
| Q9D883 | 108121 | U2AF1 | 0.523573824 | 0.000253 |
| Q9R1Q6 | 65963 | TMEM176B | 0.523521999 | 0.0044414 |
| Q3TB65 | 75805 | NLN | 0.523446765 | 0.0008161 |
| Q5SSP3 | 55963 | SLC1A4 | 0.523407964 | 0.0005911 |
| Q99KR3 | 212442 | LACTB2 | 0.5233987 | 0.0002169 |
| A0A087WRU0 | 21961 | TNS1 | 0.523323641 | 0.0001234 |
| Q9DCV4 | 66302 | RMDN1 | 0.523301221 | 0.004474 |
| Q6ZPJ3 | 217342 | UBE2O | 0.522923119 | 9.835E-05 |
| D3Z061 | 231380 | UBA6 | 0.522915745 | 0.0002581 |
| Q99JW7 | 12534 | CDK1 | 0.5228538 | 0.0031767 |
| Q9Z1N5 | 53817 | DDX39B | 0.522828882 | 0.0003404 |
| Q6A0A9 | 218236 | FAM120A | 0.52267537 | 0.0002059 |
| Q91WS0 | 52637 | CISD1 | 0.52257507 | 0.0001182 |
| A7VJ98 | 63985 | GMFB | 0.522530501 | 0.0063213 |
| P80313 | 12468 | CCT7 | 0.522405845 | 2.229E-06 |
| P53994 | 59021 | RAB2A | 0.522394862 | 0.0002197 |
| Q3TXS7 | 70247 | PSMD1 | 0.521845752 | 1.983E-05 |
| G5E866 | 81898 | SF3B1 | 0.521659274 | 4.725E-06 |
| Q9DCN2 | 109754 | cyb5r3 | 0.521506991 | 0.000219 |
| P62317 | 107686 | SNRPD2 | 0.521453991 | 0.0001285 |
| Q9Z277 | 22385 | BAZ1B | 0.521124537 | 7.002E-05 |
| Q64435 | 94284 | Ugt1a6a | 0.521094375 | 0.0019649 |
| Q9D820 | 67939 | Prorsd1 | 0.520987832 | 0.0017852 |
| P62962 | 18643 | PFN1 | 0.520929926 | 3.328E-08 |
| Q9JJ94 | 68475 | SSNA1 | 0.520713993 | 0.0020509 |
| Q3TC93 | 58240 | HS1BP3 | 0.520692453 | 0.0029727 |
| A6H663 | 29810 | BAG3 | 0.520687372 | 0.0001224 |
| P17751 | 21991 | TPI1 | 0.520467137 | 6.684E-06 |
| Q80WJ7 | 67154 | MTDH | 0.520345279 | 0.0004775 |
| Q9WUP7 | 56207 | UCHL5 | 0.520141677 | 0.0010228 |
| Q64105 | 20751 | SPR | 0.520130919 | 1.127E-05 |
| Q78ZA7 | 17955 | NAP1L4 | 0.520075162 | 0.0002366 |
| O54774 | 11776 | AP3D1 | 0.51998618 | 1.293E-05 |
| Q3V3N5 | 230737 | GNL2 | 0.519795122 | 0.0014935 |
| P70296 | 23980 | PEBP1 | 0.519639471 | 0.0003254 |
| O35841 | 11800 | API5 | 0.519560502 | 0.0004604 |
| E9Q6J5 | 665775 | Bod1l | 0.519513608 | 0.000464 |
| Q8CIV8 | 70430 | TBCE | 0.51946573 | 0.0008309 |
| Q9WVS5 |  |  | 0.519414302 | 4.591E-05 |
| P97351 | 20091 | Rps3a1 | 0.519315571 | 3.077E-05 |
| Q3ULZ3 | 107272 | PSAT1 | 0.518976535 | 3.315E-06 |
| Q3U2G2 | 15525 | HSPA4 | 0.518960015 | 9.407E-06 |
| Q80UE5 | 13822 | EPB41L2 | 0.518813313 | 3.756E-06 |
| Q6NVF9 | 432508 | CPSF6 | 0.518809685 | 0.0019181 |
| A2AP32 | 230075 | NDUFB6 | 0.51880795 | 0.0004756 |
| Q6A099 | 107338 | GBF1 | 0.518718217 | 0.0005479 |
| Q8CJ53 | 106628 | TRIP10 | 0.518529913 | 0.0025698 |
| P14576 | 24067 | Srp54a | 0.518503593 | 2.63E-06 |
| O55029 | 50797 | COPB2 | 0.518423854 | 0.0006373 |
| G3UX26 | 22334 | VDAC2 | 0.518406495 | 9.15E-07 |
| B2RQQ5 | 17755 | MAP1B | 0.518404816 | 3.408E-06 |
| Q9WVA3 | 12237 | BUB3 | 0.518395393 | 0.0002586 |
| A2BGI8 | 66101 | PPIH | 0.518243432 | 0.0014048 |
| Q9DBR0 | 56399 | AKAP8 | 0.518181682 | 5.957E-05 |
| Q3ULG4 | 19185 | PSMD4 | 0.518103721 | 6.943E-05 |
| P97450 | 11957 | ATP5J | 0.51791354 | 0.0046836 |
| Q9Z2A7 | 13350 | DGAT1 | 0.517879447 | 0.0021193 |
| P63276 | 20068 | RPS17 | 0.51778612 | 6.516E-06 |
| E9QP99 | 269682 | GOLGA3 | 0.517705104 | 6.635E-06 |
| P23492 | 18950 | PNP | 0.517569702 | 0.0080656 |
| P61222 | 24015 | ABCE1 | 0.517538413 | 2.267E-05 |
| D3YVN7 | 100041265 | Gm9755 | 0.517439755 | 0.0001579 |
| Q3V1L7 | 80294 | POFUT2 | 0.517300042 | 0.0002897 |
| P12787 | 12858 | Cox5a | 0.517213605 | 0.0019757 |
| P63037 | 15502 | DNAJA1 | 0.51698731 | 0.0003725 |
| Q9WTP6 | 11637 | AK2 | 0.516900503 | 0.000449 |
| Q569Z5 | 212880 | DDX46 | 0.516808774 | 0.000517 |
| O35593 | 59029 | PSMD14 | 0.516805559 | 0.0007433 |
| Q8BN64 | 65967 | EEFSEC | 0.516569432 | 0.002464 |
| Q99P72 | 68585 | RTN4 | 0.516499152 | 1.072E-05 |
| Q61699 | 15505 | HSPH1 | 0.516445704 | 0.0002507 |
| Q921H8 | 113868 | Acaa1a | 0.516404517 | 1.428E-05 |
| Q3URN5 | 56321 | AATF | 0.516227008 | 0.0004756 |
| Q5SS83 | 14252 | FLOT2 | 0.516190465 | 0.0004614 |
| Q9QUM9 | 26443 | PSMA6 | 0.515699065 | 2.775E-05 |
| Q921C5 | 76895 | BICD2 | 0.515675496 | 0.0006804 |
| P70362 | 22230 | UFD1L | 0.515607139 | 3.637E-06 |
| Q3UHX2 | 231887 | PDAP1 | 0.515559095 | 0.0006638 |
| Q8BFZ9 | 244373 | ERLIN2 | 0.515533799 | 0.0008086 |
| Q9DAW9 | 71994 | CNN3 | 0.514881021 | 0.000632 |
| Q9CQF0 | 66419 | MRPL11 | 0.514824671 | 0.0002623 |
| P06745 | 14751 | Gpi1 | 0.514807195 | 1.328E-06 |
| Q921S7 | 56280 | MRPL37 | 0.514789245 | 0.0004113 |
| F8WGB3 | 66052 | SDHC | 0.514671691 | 5.364E-05 |
| Q05D44 | 226982 | EIF5B | 0.5145719 | 2.236E-05 |
| Q3UW32 | 67628 | ANP32B | 0.514505516 | 0.0002198 |
| Q3TM89 | 76302 | PCNP | 0.514220253 | 0.0002742 |
| E3SRG7 | 17128 | SMAD4 | 0.514181836 | 0.0019709 |
| Q923D2 | 233016 | BLVRB | 0.514165286 | 7.328E-05 |
| Q9DAW6 | 70052 | PRPF4 | 0.514094339 | 0.0001653 |
| P80314 | 12461 | CCT2 | 0.514035708 | 1.126E-05 |
| Q6PDG0 | 70699 | NUP205 | 0.513789102 | 0.0003138 |
| Q9DBE9 | 56095 | FTSJ3 | 0.51330246 | 0.0001203 |
| P53569 | 12607 | CEBPZ | 0.513077078 | 0.000417 |
| P17426 | 11771 | AP2A1 | 0.512911908 | 1.47E-05 |
| Q8CHW4 | 224045 | EIF2B5 | 0.512796768 | 0.0001089 |
| P61965 | 140858 | WDR5 | 0.512747842 | 0.003734 |
| Q6P4T2 | 320632 | SNRNP200 | 0.512714365 | 5.945E-05 |
| P08074 | 12409 | Cbr2 | 0.512602462 | 2.623E-05 |
| E9QLZ0 | 216148 | SHC2 | 0.512493057 | 0.001218 |
| A0A087WNZ7 | 14897 | TRIP12 | 0.512393562 | 0.0008644 |
| P35279 | 19346 | RAB6A | 0.512367899 | 0.0006175 |
| Q921N6 | 228889 | DDX27 | 0.512336653 | 3.817E-05 |
| Q8VI75 | 75751 | IPO4 | 0.512280458 | 3.677E-05 |
| Q9CW03 | 13006 | SMC3 | 0.512113601 | 0.000101 |
| Q9CPQ8 | 27425 | ATP5L | 0.512026021 | 9.803E-05 |
| Q9CX34 | 67955 | SUGT1 | 0.511975168 | 0.0007903 |
| Q14DR9 | 67475 | ERO1LB | 0.51196632 | 0.0059377 |
| O88492 | 57435 | PLIN4 | 0.511925085 | 0.0003576 |
| Q3TDD8 | 75705 | EIF4B | 0.511710334 | 0.0003492 |
| S4R1L5 | 12211 | BIRC6 | 0.511660909 | 0.0007412 |
| Q3UA17 | 56428 | MTCH2 | 0.511614443 | 4.893E-05 |
| O08553 | 12934 | DPYSL2 | 0.511541252 | 1.002E-05 |
| Q8CC03 | 11765 | AP1G1 | 0.51139698 | 1.214E-06 |
| P10649 | 14862 | GSTM1 | 0.511394629 | 0.0022342 |
| O70475 | 22235 | UGDH | 0.511365734 | 9.971E-05 |
| Q3U868 | 11545 | PARP1 | 0.511308364 | 2.782E-06 |
| B2RXS4 | 140570 | PLXNB2 | 0.511284406 | 2.945E-05 |
| Q8C1A5 | 50492 | THOP1 | 0.511244156 | 9.535E-06 |
| Q03958 | 14976 | PFDN6 | 0.511196088 | 0.0019201 |
| A2RRK3 | 20874 | SLK | 0.511175819 | 0.0008082 |
| Q3UE92 | 170750 | XPNPEP1 | 0.510888609 | 2.143E-05 |
| Q9Z1F9 | 50995 | UBA2 | 0.510838702 | 0.0004766 |
| D3YTP0 | 68428 | STEAP3 | 0.510792974 | 0.0008129 |
| P00493 | 15452 | hprT | 0.510562207 | 3.964E-05 |
| Q3TVZ1 | 12359 | CAT | 0.510474689 | 1.791E-05 |
| Q7TSS2 | 70093 | UBE2Q1 | 0.510460374 | 0.0037569 |
| P32067 | 20823 | SSB | 0.510150756 | 9.271E-06 |
| P19324 | 12406 | SERPINH1 | 0.509915025 | 6.014E-06 |
| Q5SVG5 | 11764 | AP1B1 | 0.509881959 | 0.0004227 |
| O35134 | 20019 | POLR1A | 0.509791217 | 0.0007801 |
| O35343 | 16649 | KPNA4 | 0.509620401 | 0.006503 |
| Q8C878 | 22200 | UBA3 | 0.509140584 | 0.0001957 |
| O09172 | 14630 | GCLM | 0.509074588 | 3.931E-05 |
| Q99J36 | 233802 | THUMPD1 | 0.509045966 | 0.0025133 |
| Q3TQY2 | 67665 | DCTN4 | 0.509020373 | 0.0017808 |
| Q60676 | 19060 | PPP5C | 0.508993732 | 1.364E-05 |
| Q8BGS2 | 66162 | bola2 | 0.508787358 | 0.0001055 |
| Q9JHU4 | 13424 | DYNC1H1 | 0.508751558 | 1.485E-05 |
| Q6A068 | 71702 | CDC5L | 0.508508343 | 5.261E-05 |
| Q9JLI6 | 50880 | SCLY | 0.508471072 | 0.0003893 |
| F8WH41 | 268980 | STRN | 0.508404963 | 0.0024964 |
| P28271 | 11428 | ACO1 | 0.508348856 | 0.000106 |
| P08249 | 17448 | MDH2 | 0.508183376 | 1.417E-05 |
| Q5JC28 | 13858 | EPS15 | 0.5081476 | 0.0010271 |
| Q3TJZ7 | 52009 | HN1L | 0.508091393 | 0.0003172 |
| F6YMR0 | 228836 | DLGAP4 | 0.507979246 | 0.0024217 |
| Q9D6E8 | 100126824 | SCO2 | 0.507973256 | 0.0007692 |
| E9Q7G1 | 66676 | TMED7 | 0.507953908 | 0.0010063 |
| Q3UPL0 | 69162 | SEC31A | 0.507922468 | 5.114E-05 |
| Q9CZX0 | 74195 | ELP3 | 0.507880949 | 0.0030754 |
| Q9D6K5 | 24071 | SYNJ2BP | 0.507827027 | 0.0003095 |
| Q3TI79 | 245474 | DKC1 | 0.50752509 | 0.0004999 |
| Q8C2A3 | 235072 | SEPT7 | 0.50730201 | 0.0009254 |
| Q9CQV8 | 54401 | YWHAB | 0.50716085 | 5.101E-05 |
| Q60597 | 18293 | OGDH | 0.506981221 | 3.371E-05 |
| Q3THS6 | 232087 | MAT2A | 0.506901279 | 0.0067831 |
| P05132 | 18747 | PRKACA | 0.506747997 | 0.0003811 |
| P62814 | 11966 | ATP6V1B2 | 0.506677811 | 0.0003628 |
| E9Q555 | 672511 | RNF213 | 0.506616869 | 0.0001357 |
| Q8VDG8 | 330260 | PON2 | 0.50648243 | 2.633E-06 |
| O55222 | 16202 | ILK | 0.506281236 | 0.0013144 |
| P63328 | 19055 | PPP3CA | 0.506279489 | 9.66E-05 |
| F6RPJ9 | 15925 | IDE | 0.506238372 | 0.0001959 |
| Q61733 | 57312 | MRPS31 | 0.506237081 | 0.0079709 |
| P62754 | 20104 | RPS6 | 0.506052116 | 2.454E-05 |
| Q00612 | 14381 | G6pdx | 0.506035714 | 8.589E-06 |
| Q3TML7 |  |  | 0.50593057 | 0.0006626 |
| Q8R3C6 | 74111 | RBM19 | 0.505751733 | 0.0002158 |
| Q8VDD5 | 17886 | MYH9 | 0.505397427 | 3.263E-07 |
| Q9D5T0 | 67979 | ATAD1 | 0.505387338 | 0.0004114 |
| Q9JKF1 | 29875 | iqgap1 | 0.505293515 | 1.091E-06 |
| Q922F4 | 67951 | TUBB6 | 0.505237661 | 0.0002009 |
| Q3U417 | 67533 | PPFIBP1 | 0.50518277 | 0.0003744 |
| B9EKT6 | 404710 | IQGAP3 | 0.505166739 | 0.0002698 |
| Q4FZL1 | 13681 | EIF4A1 | 0.505157492 | 5.837E-05 |
| P99027 | 67186 | RPLP2 | 0.505090407 | 1.901E-05 |
| P52196 | 22117 | TST | 0.504852817 | 0.0001953 |
| Q68FD5 | 67300 | CLTC | 0.50481465 | 3.235E-06 |
| Q0QEW9 | 19899 | RPL18 | 0.504375761 | 8.01E-06 |
| G3X9V2 | 12388 | CTNND1 | 0.504326862 | 2.857E-05 |
| Q6P1B9 | 30948 | BIN1 | 0.504154336 | 0.0004367 |
| P37913 | 16881 | LIG1 | 0.504135725 | 0.0004117 |
| E9PXB7 | 83814 | NEDD4L | 0.504122229 | 0.0052292 |
| Q5U438 | 18148 | NPM1 | 0.503956308 | 3.874E-07 |
| A2AKI5 | 16410 | ITGAV | 0.503915559 | 8.625E-05 |
| P47911 | 19988 | RPL6 | 0.503823546 | 3.585E-06 |
| Q3TL58 | 21402 | Skp1a | 0.503800722 | 0.000139 |
| P47968 | 19895 | RPIA | 0.503661453 | 0.0098257 |
| Q3U8R9 | 53382 | TXNL1 | 0.503499104 | 0.0002865 |
| Q6ZQ58 | 73158 | LARP1 | 0.503441082 | 0.0002646 |
| D3YXK2 | 224903 | SAFB | 0.503267578 | 0.0001244 |
| O88477 | 140486 | IGF2BP1 | 0.503095654 | 0.0001241 |
| Q3U3C4 | 20405 | SH3GL1 | 0.502997924 | 0.0097725 |
| O35129 | 12034 | PHB2 | 0.502953356 | 6.539E-06 |
| Q3TE06 | 22388 | WDR1 | 0.502621346 | 3.655E-06 |
| E0CYI7 | 78581 | UTP23 | 0.502522413 | 0.0003798 |
| Q99LS3 | 100678 | PSPH | 0.502473285 | 0.0004911 |
| Q80VJ3 | 381101 | DNPH1 | 0.50244781 | 0.0015642 |
| E9QAH1 | 224139 | GOLGB1 | 0.50232816 | 4.047E-05 |
| Q3T9L0 | 68278 | ddx39 | 0.502320974 | 0.0005241 |
| Q8VI84 | 57753 | NOC3L | 0.502196583 | 2.185E-05 |
| P35282 | 216344 | RAB21 | 0.502058095 | 4.656E-06 |
| P49586 | 13026 | PCYT1A | 0.501904009 | 0.0003352 |
| Q3UAY3 | 269252 | GTF3C4 | 0.501900085 | 0.0027673 |
| Q80UJ7 | 226407 | RAB3GAP1 | 0.501745877 | 0.0024724 |
| Q7TMI0 | 69077 | PSMD11 | 0.501714651 | 2.101E-07 |
| P52825 | 12896 | cpt2 | 0.501640586 | 4.945E-05 |
| P14069 | 20200 | S100A6 | 0.501578549 | 0.002574 |
| Q8K1R7 | 217718 | NEK9 | 0.501573741 | 1.217E-07 |
| Q3TW51 | 56463 | SND1 | 0.501534634 | 7.014E-06 |
| Q8BTB8 | 18120 | MRPL49 | 0.501486963 | 5.97E-06 |
| Q3U7R1 | 23943 | ESYT1 | 0.50124735 | 2.268E-05 |
| Q8K009 | 216188 | ALDH1L2 | 0.501223515 | 4.254E-05 |
| P52431 | 18971 | POLD1 | 0.501196854 | 0.0008044 |
| P07901 | 15519 | HSP90AA1 | 0.501053377 | 1.932E-06 |
| Q3UIJ2 | 26905 | Eif2s3x | 0.500966847 | 1.717E-05 |
| Q8BMS1 | 97212 | HADHA | 0.500851052 | 5.317E-05 |
| P62334 | 67089 | PSMC6 | 0.5007752 | 1.827E-06 |
| Q05BN2 | 18813 | PA2G4 | 0.500731354 | 2.209E-06 |
| Q3URM4 | 76299 | ERP44 | 0.500726397 | 0.0003797 |
| Q1KYM0 |  |  | 0.500069783 | 4.445E-05 |
| Q5SWU9 | 107476 | ACACA | 0.499824696 | 0.000138 |
| Q6P8N8 | 270166 | CLPX | 0.499819507 | 0.0001272 |
| Q9CY49 | 27368 | TBL2 | 0.499725312 | 0.000343 |
| Q7TPT7 | 22321 | VARS | 0.499624284 | 4.949E-05 |
| Q6NV83 | 67958 | U2SURP | 0.499533868 | 0.0001123 |
| Q3UUX9 | 14569 | GDI2 | 0.499453626 | 1.271E-07 |
| Q45VK5 | 16201 | ILF3 | 0.499423068 | 0.0002878 |
| E9PXX7 | 105245 | TXNDC5 | 0.499360085 | 1.931E-06 |
| Q8BXZ1 | 67988 | TMX3 | 0.499206717 | 0.002878 |
| Q9DAR7 | 69305 | DCPS | 0.499071499 | 1.004E-05 |
| P20029 | 14828 | HSPA5 | 0.498941845 | 6.654E-07 |
| P57776 | 66656 | EEF1D | 0.498903055 | 0.0098587 |
| Q8VE10 | 70999 | NAA40 | 0.498822969 | 0.0005281 |
| V9GXJ1 |  |  | 0.498743746 | 0.0019041 |
| P17742 | 268373 | PPIA | 0.498625243 | 3.53E-06 |
| P62075 | 30055 | TIMM13 | 0.498614997 | 0.000228 |
| Q9JIF7 | 70349 | COPB1 | 0.498606332 | 3.323E-05 |
| Q99K30 | 98845 | EPS8L2 | 0.498509843 | 0.0076243 |
| A2AA71 | 77371 | sec24a | 0.498509763 | 0.0032951 |
| A0A0A6YX73 | 19087 | PRKAR2A | 0.498476927 | 0.001303 |
| Q3TG21 | 66335 | ATP6V1C1 | 0.498371093 | 1.242E-05 |
| Q62084 | 18938 | PPP1R14B | 0.498364847 | 0.0006429 |
| Q8CGP5 | 319173 | Hist1h2af | 0.498325239 | 8E-06 |
| P56391 | 110323 | Cox6b1 | 0.498293163 | 0.0006253 |
| Q6NXL1 | 69608 | SEC24D | 0.498273597 | 0.0002093 |
| F8WJI3 | 381045 | CCDC58 | 0.498263118 | 0.005564 |
| Q9CPP6 | 68202 | NDUFA5 | 0.498232316 | 0.0038132 |
| P31230 | 13722 | AIMP1 | 0.498145432 | 1.341E-05 |
| P31786 | 13167 | DBI | 0.497995118 | 0.0001633 |
| Q9D1M7 | 66120 | FKBP11 | 0.497789615 | 0.0059443 |
| P62960 | 22608 | YBX1 | 0.49742685 | 0.000689 |
| Q3TFQ8 | 110078 | PYGB | 0.497071023 | 3.938E-07 |
| Q61171 | 21672 | PRDX2 | 0.497025752 | 4.858E-07 |
| E9Q9E1 | 208643 | EIF4G1 | 0.496871052 | 6.388E-06 |
| Q9JI13 | 65961 | UTP3 | 0.496826339 | 4.092E-05 |
| Q6IRT4 | 66085 | EIF3F | 0.49672917 | 0.0003254 |
| Q6DI95 | 320938 | TNPO3 | 0.496662516 | 0.0045743 |
| P54775 | 23996 | PSMC4 | 0.496658385 | 3.109E-05 |
| Q9R1T2 | 56459 | SAE1 | 0.496513428 | 0.0051508 |
| Q3U0J1 | 68816 | PPIL1 | 0.496443854 | 0.0042041 |
| Q8R1B4 | 56347 | EIF3C | 0.496398877 | 0.0001103 |
| Q9WU28 | 56612 | PFDN5 | 0.496116419 | 0.0002057 |
| Q9JHW2 | 52633 | NIT2 | 0.496084467 | 5.251E-05 |
| G3UWE1 | 106529 | TECR | 0.496081301 | 4.138E-06 |
| Q8BH86 | 217830 | 9030617O03Rik | 0.496074942 | 0.0003595 |
| Q8C660 | 108707 | FAM207A | 0.495752081 | 0.0012925 |
| Q8JZQ9 | 27979 | EIF3B | 0.495686102 | 7.287E-05 |
| P40142 | 21881 | TKT | 0.495505813 | 4.907E-08 |
| Q9QYB1 | 29876 | CLIC4 | 0.495102562 | 0.0001666 |
| Q62145 | 14470 | RABAC1 | 0.495085718 | 0.0001329 |
| Q99JY0 | 231086 | HADHB | 0.495059785 | 0.0004071 |
| Q91W96 | 52206 | ANAPC4 | 0.495020815 | 0.0002108 |
| O70579 | 20524 | SLC25A17 | 0.494941374 | 0.0089972 |
| Q69ZY3 | 74737 | PCF11 | 0.494617498 | 0.0021275 |
| Q3UKA1 | 100037258 | DNAJC3 | 0.494460778 | 0.0001403 |
| Q3TDE4 | 59287 | NCSTN | 0.494407358 | 0.0001201 |
| P05622 | 18596 | PDGFRB | 0.494360378 | 0.0001235 |
| Q9CWZ5 | 56200 | DDX21 | 0.494143385 | 1.086E-05 |
| Q6ZQ38 | 71902 | CAND1 | 0.494060583 | 1.408E-05 |
| Q8K363 | 66942 | DDX18 | 0.494028344 | 9.209E-06 |
| Q7TNV0 | 110052 | DEK | 0.493759645 | 0.0002316 |
| P61804 | 13135 | DAD1 | 0.493648272 | 0.001026 |
| P97494 | 14629 | GCLC | 0.493605184 | 0.0017871 |
| Q3UMT7 | 15388 | HNRNPL | 0.493304382 | 0.0002476 |
| Q9CQM9 | 30926 | GLRX3 | 0.49326662 | 9.06E-05 |
| Q3TIV5 | 69082 | ZC3H15 | 0.493080901 | 0.000171 |
| Q8BTF0 | 12847 | COPA | 0.492984508 | 4.818E-07 |
| Q9R257 | 15199 | HEBP1 | 0.492663918 | 2.837E-05 |
| A0A087WPL5 | 13211 | DHX9 | 0.492648524 | 6.502E-05 |
| J3QPZ8 | 20918 | EIF1 | 0.492615035 | 0.0017631 |
| Q3U9A8 | 56726 | SH3BGRL | 0.492586178 | 0.0033839 |
| P81117 | 53322 | NUCB2 | 0.492524569 | 3.756E-06 |
| P47963 | 270106 | RPL13 | 0.492200061 | 0.000192 |
| E9PX48 | 67299 | DOCK7 | 0.492101225 | 0.0001838 |
| Q5D0F3 | 21354 | TAP1 | 0.492100908 | 0.0018283 |
| Q3U2B5 | 68188 | SYMPK | 0.492063719 | 0.0010569 |
| P47955 | 56040 | RPLP1 | 0.492021801 | 3.323E-05 |
| Q8BYY4 | 69863 | TTC39B | 0.492018124 | 0.0012579 |
| Q3V2N5 | 20650 | SNTB2 | 0.491960362 | 0.0007326 |
| P49717 | 17217 | MCM4 | 0.491939244 | 0.0007177 |
| P35486 | 18597 | PDHA1 | 0.491833023 | 0.0002412 |
| Q9Z2X1 | 98758 | HNRNPF | 0.491579903 | 8.935E-08 |
| P62196 | 19184 | PSMC5 | 0.491352247 | 1.809E-05 |
| Q3V1M8 | 110611 | HDLBP | 0.491272828 | 2.193E-05 |
| Q9CS42 | 110639 | PRPS2 | 0.491224373 | 0.0005549 |
| Q3TZU7 | 66616 | SNX9 | 0.491206183 | 2.676E-05 |
| Q3TW28 | 22019 | TPP2 | 0.491155223 | 2.218E-05 |
| P42125 | 13177 | ECI1 | 0.491154397 | 0.0002141 |
| Q91YS7 | 26396 | MAP2K2 | 0.491146156 | 0.0022881 |
| Q3UJZ7 | 67134 | NOP56 | 0.491100827 | 2.706E-05 |
| Q3TUI9 | 26442 | PSMA5 | 0.490902681 | 5.984E-05 |
| P54227 | 16765 | STMN1 | 0.490647236 | 0.0001176 |
| Q60520 | 20466 | SIN3A | 0.490616515 | 0.0078874 |
| Q3TVM1 | 67465 | SF3A1 | 0.490490369 | 8.178E-05 |
| P47856 | 14583 | GFPT1 | 0.490416259 | 0.0001385 |
| Q9CTR1 | 404634 | H2AFY2 | 0.49033565 | 0.0005488 |
| Q8BLN5 | 16987 | LSS | 0.490181675 | 0.0004486 |
| Q9WVQ5 | 56369 | APIP | 0.489932017 | 0.0006309 |
| Q8BH80 | 56491 | VAPB | 0.48988222 | 0.0006504 |
| H7BX88 | 12908 | CRAT | 0.489864465 | 0.0007608 |
| Q60605 | 17904 | MYL6 | 0.489654004 | 0.0001769 |
| P97370 | 11933 | ATP1B3 | 0.489532409 | 1.777E-05 |
| Q9CR57 | 67115 | RPL14 | 0.489459529 | 9.088E-06 |
| Q01853 | 269523 | VCP | 0.489247145 | 1.325E-06 |
| Q68FL6 | 216443 | MARS | 0.489238754 | 4.002E-05 |
| Q3U9G9 | 98386 | LBR | 0.489105395 | 0.0014356 |
| Q08288 | 17089 | LYAR | 0.488992558 | 0.0032402 |
| Q8CD92 | 74196 | TTC27 | 0.488610495 | 0.0036293 |
| Q99LJ6 | 67305 | GPX7 | 0.488604199 | 0.000394 |
| Q3TXN6 | 228410 | CSTF3 | 0.488496918 | 0.0001168 |
| Q3UT19 | 70767 | PRPF3 | 0.488311777 | 0.0007109 |
| Q91WP9 | 110095 | PYGL | 0.488311609 | 0.0015232 |
| O88398 | 11567 | AVIL | 0.488216626 | 0.0001549 |
| Q8R3X4 | 21856 | TIMM44 | 0.487928433 | 5.948E-05 |
| Q9Z0F7 | 20618 | SNCG | 0.487595436 | 2.256E-05 |
| Q8BH59 | 78830 | SLC25A12 | 0.487570675 | 7.102E-06 |
| Q8C1V4 | 85305 | KARS | 0.487269521 | 6.3E-05 |
| Q3TAW4 | 27054 | SEC23B | 0.487228439 | 0.0006229 |
| Q3TT85 | 56361 | PUS1 | 0.487030159 | 0.0002125 |
| Q06185 | 11958 | Atp5k | 0.486782819 | 2.329E-05 |
| Q99PT1 | 192662 | ARHGDIA | 0.486754569 | 9.43E-05 |
| P46737 | 210766 | BRCC3 | 0.486665016 | 0.0004658 |
| Q8R1Q8 | 235661 | DYNC1LI1 | 0.486556988 | 3.613E-05 |
| Q6ZWS7 | 12325 | CAMK2G | 0.4863782 | 8.991E-06 |
| D6RGL6 | 77219 | PTGR2 | 0.486344321 | 0.0036069 |
| O70310 | 18107 | NMT1 | 0.486307991 | 0.0001271 |
| E9Q715 | 192196 | LUC7L2 | 0.486186796 | 0.0005825 |
| Q08093 | 12798 | CNN2 | 0.485846331 | 9.328E-05 |
| Q64511 | 21974 | TOP2B | 0.485455708 | 4.323E-06 |
| P83887 | 103733 | TUBG1 | 0.485165373 | 5.46E-05 |
| Q3TJF2 | 67059 | OLA1 | 0.484983476 | 6.072E-05 |
| Q9EPL8 | 233726 | IPO7 | 0.484935934 | 9.458E-06 |
| Q99KQ4 | 59027 | NAMPT | 0.484687381 | 3.793E-05 |
| Q3U0I9 | 231834 | SNX8 | 0.484548614 | 0.0004026 |
| D3Z0M9 | 74351 | DDX23 | 0.484519709 | 0.0002637 |
| Q9CQ54 | 68197 | NDUFC2 | 0.484505965 | 0.0019177 |
| Q80TX7 |  |  | 0.484413924 | 0.0010385 |
| F6ZFT1 | 70316 | NDUFAB1 | 0.484358072 | 1.662E-05 |
| Q8C1B7 | 52398 | SEPT11 | 0.484343376 | 0.0015872 |
| E0CX20 | 231889 | BUD31 | 0.484337006 | 0.000948 |
| Q3U561 | 19896 | RPL10A | 0.48429714 | 5.062E-05 |
| P70388 | 19360 | RAD50 | 0.484129518 | 0.0006689 |
| Q91VC3 | 192170 | EIF4A3 | 0.484005601 | 0.0008987 |
| Q3UQU5 | 319322 | SF3B2 | 0.483810072 | 0.0001771 |
| Q99MR6 | 83701 | SRRT | 0.483708774 | 0.0004 |
| P45952 | 11364 | ACADM | 0.483695399 | 4.5E-05 |
| Q9Z1T1 | 11774 | AP3B1 | 0.483573075 | 0.0004896 |
| P52927 | 15364 | hmga2 | 0.4834955 | 0.0035926 |
| Q3UDX4 | 13204 | DHX15 | 0.483363945 | 0.0001816 |
| P50543 | 20195 | S100A11 | 0.483351204 | 6.606E-06 |
| Q91YR7 | 68879 | PRPF6 | 0.483178099 | 0.0002435 |
| Q8BSL7 | 11841 | ARF2 | 0.483099092 | 0.0001336 |
| Q8R2M2 | 99480 | DNTTIP2 | 0.483082485 | 0.0003296 |
| B2CY77 | 16785 | RPSA | 0.483018852 | 3.408E-05 |
| Q99JI4 | 66413 | PSMD6 | 0.482918014 | 2.863E-06 |
| B2RQS1 | 94186 | STRN3 | 0.482586489 | 0.0029554 |
| P56376 | 66204 | ACYP1 | 0.482570183 | 0.000491 |
| Q61941 | 18115 | NNT | 0.482506165 | 1.682E-05 |
| A2CG35 | 19328 | RAB12 | 0.482484843 | 5.505E-05 |
| Q99PV0 | 192159 | PRPF8 | 0.482274232 | 0.0026741 |
| P70698 | 51797 | Ctps | 0.482190877 | 0.000476 |
| Q3UFR4 | 20514 | slc1a5 | 0.482147598 | 0.0024764 |
| Q8R502 | 100604 | LRRC8C | 0.482117176 | 0.0029629 |
| P47740 | 11671 | ALDH3A2 | 0.481967349 | 5.469E-05 |
| Q9JI10 | 56274 | stk3 | 0.481930941 | 0.0039412 |
| Q8BL66 | 216238 | EEA1 | 0.481659721 | 5.837E-05 |
| Q5RKP4 | 103963 | RPN1 | 0.48164662 | 1.117E-05 |
| P46935 | 17999 | NEDD4 | 0.48164277 | 1.829E-05 |
| Q920A5 | 74617 | SCPEP1 | 0.481427327 | 0.0014451 |
| Q8R1F1 | 227737 | FAM129B | 0.481175191 | 0.0031382 |
| Q8CGK3 | 74142 | LONP1 | 0.481172923 | 2.016E-05 |
| Q9R0P5 | 56431 | DSTN | 0.480989654 | 1.798E-05 |
| P16460 | 11898 | ASS1 | 0.480971887 | 2.534E-05 |
| D3YWF6 | 107260 | OTUB1 | 0.480686786 | 1.838E-05 |
| A2AE27 | 109674 | AMPD2 | 0.480585056 | 0.0011223 |
| P50247 | 11615 | Gm4737 | 0.480507706 | 3.844E-05 |
| Q5SYD0 | 338367 | MYO1D | 0.480453614 | 0.0002156 |
| P10852 | 17254 | SLC3A2 | 0.480410021 | 0.000132 |
| Q3ULP8 | 78697 | PUS7 | 0.480354078 | 0.0001414 |
| Q9JJ28 | 14248 | FLII | 0.480296181 | 7.28E-06 |
| Q924A9 | 114663 | IMPA2 | 0.480149997 | 0.0007848 |
| Q9DC23 | 66861 | DNAJC10 | 0.480014166 | 0.0002019 |
| Q922R8 | 71853 | PDIA6 | 0.479940516 | 1.84E-06 |
| A2A4B3 | 64899 | LPIN3 | 0.479900854 | 0.0018035 |
| Q9CQ71 | 68240 | RPA3 | 0.479897118 | 0.0030428 |
| E9QQ99 | 74126 | SYVN1 | 0.479769821 | 0.0019596 |
| I7HJS1 | 13726 | EMD | 0.479737674 | 0.0010034 |
| Q3TJ01 | 28088 | RTCB | 0.479478244 | 1.019E-06 |
| O35685 | 18221 | NUDC | 0.479327081 | 0.0001884 |
| Q71RI9 | 229905 | KYAT3 | 0.479175019 | 0.0005911 |
| Q8R0M2 | 216558 | UGP2 | 0.47891895 | 5.944E-05 |
| Q91V64 | 66307 | ISOC1 | 0.478831471 | 0.0016493 |
| Q9Z2I8 | 20917 | SUCLG2 | 0.478820554 | 3.429E-05 |
| Q9Z1Q5 | 114584 | CLIC1 | 0.478674984 | 1.168E-07 |
| Q9DCA4 | 20088 | RPS24 | 0.478549956 | 0.0002981 |
| A0A087WQQ5 | 16709 | KTN1 | 0.478527862 | 6.481E-08 |
| Q9DCC4 | 66194 | PYCRL | 0.478078618 | 0.0004637 |
| P18760 | 12631 | CFL1 | 0.477701232 | 4.638E-05 |
| E9Q6U4 | 109054 | PFDN4 | 0.477655027 | 0.0001891 |
| A2ALV7 | 230249 | AI314180 | 0.477510337 | 0.0002278 |
| P35979 | 269261 | RPL12 | 0.477462647 | 7.417E-05 |
| Q9CPU0 | 109801 | GLO1 | 0.477236494 | 0.0002455 |
| Q8C788 | 170625 | SNX18 | 0.477229803 | 0.0001956 |
| P80316 | 12465 | CCT5 | 0.477196943 | 1.436E-05 |
| Q9JKR6 | 12282 | HYOU1 | 0.477193277 | 6.445E-08 |
| P61600 | 67877 | NAA20 | 0.477180857 | 0.0025367 |
| Q8BIZ9 | 68058 | CHD1L | 0.477074203 | 0.0001143 |
| Q0VGU9 | 170791 | RBM39 | 0.476832458 | 6.179E-05 |
| Q8BP48 | 75624 | METAP1 | 0.47682788 | 0.0001359 |
| Q9CTT4 | 17993 | NDUFS4 | 0.476805962 | 0.0021496 |
| Q8BVA5 | 68832 | LDAH | 0.476798498 | 0.0011782 |
| Q5M9L1 | 54217 | RPL36 | 0.476711311 | 2.815E-05 |
| Q9DBS1 | 74122 | TMEM43 | 0.476529701 | 0.0008124 |
| Q9DB20 | 28080 | ATP5O | 0.476305726 | 0.0049531 |
| Q2L4X1 | 66912 | BZW2 | 0.476006798 | 5.751E-05 |
| P09411 | 18655 | PGK1 | 0.475895832 | 4.006E-05 |
| P60469 | 76787 | PPFIA3 | 0.475763406 | 0.0024046 |
| E9QAS4 | 107932 | CHD4 | 0.475713997 | 2.852E-05 |
| Q9ERR7 | 93684 | sep15 | 0.47559399 | 5.96E-05 |
| Q3TFF0 | 56445 | DNAJA2 | 0.475582748 | 1.055E-05 |
| Q6GU23 | 20848 | STAT3 | 0.475455025 | 0.0014819 |
| Q6UIL4 | 66395 | AHNAK | 0.475382802 | 0.0051108 |
| Q3TV20 | 27053 | ASNS | 0.475343003 | 1.726E-05 |
| P26638 | 20226 | SARS | 0.475341549 | 1.835E-06 |
| P63001 | 19353 | RAC1 | 0.474909078 | 0.0001864 |
| Q3UT23 | 114893 | DCUN1D1 | 0.474787803 | 0.0045555 |
| Q9WU78 | 18571 | PDCD6IP | 0.474620605 | 9.265E-06 |
| Q3UXI9 | 67781 | ILF2 | 0.474509615 | 9.657E-05 |
| Q8BKZ9 | 27402 | PDHX | 0.474286068 | 0.0003388 |
| Q80UK8 | 70422 | INTS2 | 0.474266673 | 5.651E-05 |
| P23506 | 18537 | PCMT1 | 0.473885489 | 0.0005581 |
| Q8VC85 | 67207 | LSM1 | 0.473838062 | 0.0002217 |
| D3Z074 | 13367 | DIAPH1 | 0.473444972 | 3.34E-05 |
| Z4YJU8 | 99412 | GOLGA2 | 0.473419816 | 0.0051395 |
| Q9R118 | 56213 | HTRA1 | 0.473114533 | 2.893E-05 |
| Q8CAQ8 | 76614 | IMMT | 0.472857238 | 1.418E-05 |
| Q8C1M0 | 71974 | PRMT3 | 0.472758815 | 0.001375 |
| Q9JKB3 | 56449 | YBX3 | 0.472595745 | 0.0006561 |
| Q61927 |  |  | 0.472347681 | 0.000613 |
| F6XC25 | 319965 | CC2D1B | 0.472252202 | 0.0010053 |
| Q3THW7 | 68135 | EIF3H | 0.471944372 | 9.915E-05 |
| P62908 | 27050 | RPS3 | 0.471738003 | 6.633E-06 |
| Q3TBW2 | 107732 | MRPL10 | 0.471679587 | 0.000757 |
| P61620 | 53421 | SEC61A1 | 0.471600544 | 0.0024426 |
| Q61081 | 12539 | CDC37 | 0.471406595 | 0.0001155 |
| Q9R1P4 | 26440 | PSMA1 | 0.471238212 | 3.091E-05 |
| P97742 | 12894 | CPT1A | 0.471230725 | 5.009E-05 |
| E9PYA3 | 14651 | HAGH | 0.471167315 | 0.0001157 |
| Q8BPB0 | 68473 | MOB1B | 0.471068902 | 0.0065754 |
| G3UYD0 | 14886 | GTF2I | 0.470985023 | 9.37E-05 |
| Q8BWZ3 | 231713 | NAA25 | 0.470720756 | 0.0003164 |
| O35295 | 19291 | PURB | 0.470698148 | 0.0056958 |
| I4DCY6 | 18391 | SIGMAR1 | 0.470519577 | 0.000322 |
| Q8BX02 | 235041 | KANK2 | 0.470447475 | 0.0011237 |
| Q8BTW7 | 227619 | MAN1B1 | 0.470298908 | 0.0074212 |
| A1L2Z3 | 230866 | EMC1 | 0.470189544 | 0.005341 |
| Q3U646 | 11886 | ASAH1 | 0.469910752 | 0.004911 |
| P55096 | 19299 | ABCD3 | 0.469678803 | 0.001465 |
| Q8R016 | 104184 | BLMH | 0.469663911 | 6.037E-05 |
| Q3TXN0 | 64704 | HTRA2 | 0.469589023 | 0.0018434 |
| P47962 | 100503670 | RPL5 | 0.469526479 | 6.856E-05 |
| Q99LP6 | 17713 | GRPEL1 | 0.469429829 | 4.693E-06 |
| P49710 | 15163 | HCLS1 | 0.469428827 | 0.0044722 |
| P27659 | 27367 | RPL3 | 0.469367408 | 0.000751 |
| P08003 | 12304 | PDIA4 | 0.469220654 | 1.982E-07 |
| Q3UDH4 | 99683 | SEC24B | 0.469163301 | 0.0030012 |
| P63038 | 15510 | HSPD1 | 0.469148856 | 6.372E-08 |
| Q8R0R0 | 338467 | MORC3 | 0.469001432 | 0.0014795 |
| Q8VCW8 | 264895 | ACSF2 | 0.46896144 | 9.721E-06 |
| Q9ESP1 | 64136 | SDF2L1 | 0.468858921 | 8.214E-05 |
| Q811N1 |  |  | 0.468446641 | 0.0036649 |
| Q3TW96 | 227620 | UAP1L1 | 0.468378208 | 0.007582 |
| O35326 | 20384 | SRSF5 | 0.468349538 | 4.712E-05 |
| A2A9X5 | 50773 | NT5C | 0.468301463 | 0.0002778 |
| Q8BFY9 | 238799 | TNPO1 | 0.468114198 | 2.947E-06 |
| Q3U6D2 | 100088 | RCC1 | 0.468114 | 0.0003006 |
| Q9CRT8 | 73192 | XPOT | 0.468100278 | 6.526E-05 |
| B2RXT3 | 239017 | OGDHL | 0.4680054 | 0.009941 |
| E9Q616 | 66395 | AHNAK | 0.467872334 | 1.002E-06 |
| P68254 | 22630 | YWHAQ | 0.467828754 | 5.776E-06 |
| Q6PB51 | 104479 | CCDC117 | 0.467661667 | 0.0017971 |
| Q8CG48 | 14211 | SMC2 | 0.467644275 | 2.002E-06 |
| Q3UF75 | 57342 | PARVA | 0.467483154 | 0.0001623 |
| P83917 | 12412 | CBX1 | 0.46745742 | 0.0033189 |
| Q4VA28 | 19933 | RPL21 | 0.467444937 | 0.0013487 |
| P70349 | 15254 | HINT1 | 0.467339737 | 0.0003868 |
| P30412 | 19038 | PPIC | 0.467269082 | 0.0008911 |
| O55143 | 11938 | ATP2A2 | 0.467081523 | 1.867E-06 |
| P19096 | 14104 | FASN | 0.466972867 | 6.313E-07 |
| P16332 | 17850 | MUT | 0.466914954 | 0.0002326 |
| Q8CIN4 | 224105 | PAK2 | 0.466855736 | 0.0023308 |
| Q3TUW9 | 13660 | EHD1 | 0.466838388 | 3.669E-05 |
| Q2VPC9 | 19082 | PRKAG1 | 0.466790876 | 7.111E-05 |
| O08663 | 56307 | METAP2 | 0.46669141 | 4.695E-05 |
| Q3TF41 | 53605 | NAP1L1 | 0.466691155 | 5.03E-05 |
| F8WH20 | 71720 | OSBPL3 | 0.46667743 | 1.822E-05 |
| P49722 | 19166 | PSMA2 | 0.466665132 | 0.0001923 |
| P58021 | 68059 | TM9SF2 | 0.466626691 | 0.0033439 |
| Q3TXS9 | 16898 | RPS2 | 0.466581589 | 3.022E-05 |
| P62748 | 53602 | HPCAL1 | 0.466501957 | 1.022E-05 |
| Q3UK68 | 17938 | NACA | 0.466479529 | 0.0001021 |
| Q9D051 | 68263 | PDHB | 0.466425408 | 0.0001163 |
| Q5RJV4 | 72157 | PGM2 | 0.466144672 | 0.0093879 |
| A0AUN0 | 207352 | SEC23IP | 0.465993993 | 0.0001858 |
| Q4FJY5 | 67103 | PTGR1 | 0.465968445 | 0.0003113 |
| Q3UYD0 | 72554 | UTP14A | 0.465948819 | 0.0025364 |
| Q3USG5 | 100952 | EMILIN1 | 0.465877438 | 0.0011888 |
| B7ZWF1 | 13205 | DDX3X | 0.465790675 | 1.532E-05 |
| Q3TMP1 | 98488 | GTF3C3 | 0.465733365 | 0.0028687 |
| Q62426 | 13014 | CSTB | 0.465570986 | 0.0023045 |
| Q6P5F9 | 103573 | XPO1 | 0.465506739 | 3.933E-07 |
| Q9D6J6 | 72900 | NDUFV2 | 0.465316811 | 0.0007655 |
| P04117 | 11770 | FABP4 | 0.465257079 | 1.337E-06 |
| A0A0A6YW28 | 22258 | USP4 | 0.465241985 | 0.0004091 |
| Q3UGN9 | 20844 | STAM | 0.465003482 | 0.0020671 |
| Q9CT23 | 16341 | EIF3E | 0.464880768 | 5.206E-05 |
| P31938 | 26395 | MAP2K1 | 0.464528685 | 2.058E-05 |
| D3Z3F8 | 229285 | SPG20 | 0.464417362 | 5.695E-05 |
| Q9CUB4 | 68646 | NADK2 | 0.464340553 | 8.899E-05 |
| Q9D6K8 | 67391 | FUNDC2 | 0.464294917 | 0.0016673 |
| Q9CZT6 | 66497 | CMSS1 | 0.464267012 | 3.95E-07 |
| P15626 | 14863 | GSTM2 | 0.464194607 | 4.74E-05 |
| Q8K2F8 | 67070 | LSM14A | 0.464059428 | 0.0084644 |
| Q6ZWU9 | 57294 | RPS27 | 0.464043499 | 9.688E-05 |
| Q9D1H7 | 67604 | GET4 | 0.464019799 | 1.978E-05 |
| E9Q2S9 | 52715 | CCDC43 | 0.463767632 | 0.0018883 |
| P32233 | 13494 | DRG1 | 0.463685604 | 0.0002055 |
| Q8K2V6 | 76582 | IPO11 | 0.463663938 | 0.0078181 |
| B7ZWL1 | 234594 | CNOT1 | 0.463240467 | 0.0006486 |
| J3QMC5 | 100019 | Mdn1 | 0.462908939 | 3.51E-05 |
| Q8VCE7 | 73130 | TMED5 | 0.462903381 | 1.053E-05 |
| P62259 | 22627 | YWHAE | 0.46278988 | 1.004E-06 |
| P15864 | 50708 | HIST1H1C | 0.462655997 | 5.362E-05 |
| Q9CU62 | 24061 | SMC1A | 0.462567681 | 2.721E-05 |
| P48758 | 12408 | CBR1 | 0.462471926 | 0.0003186 |
| Q922A3 | 71807 | TARS2 | 0.462380701 | 0.0003035 |
| Q8BU30 | 105148 | IARS | 0.462296963 | 1.361E-06 |
| D3Z2J6 | 66958 | TMX2 | 0.462110165 | 0.001874 |
| Q9CQU0 | 66073 | TXNDC12 | 0.461796977 | 8.063E-07 |
| Q3TXJ4 | 20224 | SAR1A | 0.46166269 | 9.555E-05 |
| Q8R3C0 | 210711 | MCMBP | 0.461589102 | 0.0014681 |
| D3Z637 | 21985 | TPD52 | 0.461480569 | 1.373E-05 |
| Q9EQQ2 | 67180 | YIPF5 | 0.461293988 | 0.0002469 |
| Q9D8B3 | 75608 | CHMP4B | 0.461010252 | 0.0065341 |
| Q3UL78 | 12540 | CDC42 | 0.460872301 | 2.871E-05 |
| Q3UQD0 | 53333 | TOMM40 | 0.460654071 | 0.0014993 |
| D3YUM1 | 17995 | NDUFV1 | 0.4606432 | 3.947E-05 |
| P23116 | 13669 | EIF3A | 0.460530732 | 3.19E-05 |
| Q7TPV4 | 18432 | MYBBP1A | 0.460411172 | 3.846E-07 |
| E9QMV2 | 73112 | ABRACL | 0.460327138 | 0.0003002 |
| Q32P00 | 12417 | CBX3 | 0.460230388 | 0.0005853 |
| Q9CRY7 | 66569 | GDPD1 | 0.45995922 | 0.0001244 |
| P35550 | 14113 | FBL | 0.459810909 | 8.045E-06 |
| Q9D6R2 | 67834 | IDH3A | 0.459559495 | 1.719E-05 |
| A2BDX2 | 13480 | DPM1 | 0.458969123 | 0.0004395 |
| Q62186 | 20832 | SSR4 | 0.458831593 | 0.0001661 |
| P52624 | 22271 | UPP1 | 0.458823535 | 0.0001337 |
| Q6PFD9 | 269966 | NUP98 | 0.458609327 | 8.621E-05 |
| Q8BH04 | 74551 | PCK2 | 0.458593411 | 0.00349 |
| Q9D0I8 | 69902 | MRTO4 | 0.458554283 | 0.0018434 |
| Q3TG12 | 23874 | FARSB | 0.45844648 | 6.432E-06 |
| P60867 | 67427 | RPS20 | 0.45796233 | 0.0001825 |
| Q8BH79 | 102566 | ANO10 | 0.457953031 | 0.0005418 |
| F8WHU9 | 22687 | ZPR1 | 0.457760373 | 7.459E-05 |
| S4R1E5 | 625249 | GPX4 | 0.457673175 | 4.608E-05 |
| Q8C5B5 |  |  | 0.457583186 | 0.0005393 |
| B9EIU1 | 107508 | EPRS | 0.456962338 | 1.498E-06 |
| Q7TT37 | 230233 | IKBKAP | 0.456669307 | 3.655E-05 |
| Q3UXM3 | 94275 | MAGED1 | 0.456566522 | 0.0010032 |
| Q922Q8 | 98238 | LRRC59 | 0.45645493 | 8.195E-07 |
| P62855 | 27370 | RPS26 | 0.456452552 | 1.747E-05 |
| Q8BHA3 | 328092 | DTD2 | 0.456444605 | 0.005252 |
| F6RJV6 | 71835 | LANCL2 | 0.456196369 | 0.0009929 |
| Q8BTV2 | 269061 | CPSF7 | 0.456062912 | 0.0045665 |
| O55057 | 18582 | PDE6D | 0.456060462 | 2.922E-06 |
| P14824 | 11749 | ANXA6 | 0.45601521 | 0.0001198 |
| P97807 | 14194 | Fh1 | 0.455872893 | 8.777E-05 |
| Q99K51 | 102866 | PLS3 | 0.455789246 | 1.586E-07 |
| Q78XF5 | 66357 | OSTC | 0.455769615 | 0.0016249 |
| Q8BKS9 | 52874 | PUM3 | 0.455633773 | 2.467E-05 |
| Q9D517 | 28169 | AGPAT3 | 0.455626892 | 3.589E-06 |
| Q3UGJ7 | 18226 | NUP62 | 0.455191542 | 3.632E-05 |
| P26443 | 14661 | GLUD1 | 0.455181458 | 0.0004449 |
| Q9DBR1 | 24128 | XRN2 | 0.45509497 | 4.145E-07 |
| Q61833 | 20014 | RPN2 | 0.454961403 | 5.448E-07 |
| Q9EQK5 | 78388 | MVP | 0.454900866 | 6.267E-06 |
| Q9Z2I0 | 56384 | LETM1 | 0.454893494 | 0.0006552 |
| P42669 | 19290 | PURA | 0.454870865 | 3.653E-05 |
| B2RY90 | 664994 | Isoc2a | 0.454745564 | 0.0012807 |
| Q3UJK2 | 66870 | SERBP1 | 0.454694174 | 1.684E-05 |
| Q3TN42 | 105372 | UTP15 | 0.454686741 | 0.0004359 |
| Q8VCT3 | 215615 | RNPEP | 0.454623202 | 0.0086882 |
| Q64433 | 15528 | hspe1 | 0.454111075 | 7.723E-05 |
| P70279 | 20935 | SURF6 | 0.453460954 | 1.908E-05 |
| P67778 | 18673 | PHB | 0.453318129 | 4.688E-06 |
| Q9Z130 | 50926 | HNRNPDL | 0.453230923 | 2.91E-05 |
| A2AMW0 | 12345 | CAPZB | 0.453195156 | 1.708E-05 |
| Q7TQH0 | 233871 | ATXN2L | 0.453075274 | 0.0004757 |
| P02469 | 16777 | LAMB1 | 0.453033907 | 9.868E-07 |
| P29391 | 14325 | Ftl1 | 0.452891845 | 0.0043811 |
| Q8C845 | 27984 | EFHD2 | 0.452709452 | 0.0011145 |
| Q8VC94 | 67025 | RPL11 | 0.452592492 | 6.251E-05 |
| P62267 | 66475 | rps23 | 0.452523237 | 1.254E-05 |
| Q8CE21 | 70769 | NOLC1 | 0.452201427 | 0.0012477 |
| Q60598 | 13043 | CTTN | 0.452154568 | 0.0028249 |
| Q9CRF5 | 73674 | WDR75 | 0.45212069 | 0.0002366 |
| Q6ZQI3 | 109154 | MLEC | 0.451968141 | 2.095E-05 |
| Q9JLV5 | 26554 | CUL3 | 0.451820167 | 3.372E-05 |
| P08113 | 22027 | HSP90B1 | 0.451745364 | 2.871E-08 |
| P38647 | 15526 | HSPA9 | 0.451717302 | 2.526E-06 |
| P11983 | 21454 | TCP1 | 0.451671742 | 2.369E-05 |
| Q5XJY5 | 213827 | ARCN1 | 0.451617919 | 7.184E-06 |
| S4R294 | 226562 | PRRC2C | 0.451529274 | 0.0049026 |
| Q3TMB5 | 22051 | TRIP6 | 0.451390715 | 0.0004565 |
| Q91VJ4 | 106504 | STK38 | 0.451340932 | 0.0005701 |
| P61290 | 19192 | PSME3 | 0.451260326 | 1.791E-07 |
| P68040 | 14694 | RACK1 | 0.451171918 | 3.307E-05 |
| Q80XR5 | 22185 | U2AF2 | 0.451102001 | 0.0023389 |
| Q6PAR5 | 66691 | GAPVD1 | 0.450996403 | 0.0002011 |
| Q4VA53 | 100710 | PDS5B | 0.450868837 | 1.11E-06 |
| Q9DB05 | 108124 | NAPA | 0.450770397 | 4.002E-05 |
| Q6DFW4 | 55989 | NOP58 | 0.450711795 | 2.68E-05 |
| Q62351 | 22042 | TFRC | 0.450569179 | 2.053E-05 |
| Q9D8E6 | 67891 | RPL4 | 0.450522835 | 1.443E-05 |
| Q62422 | 20409 | OSTF1 | 0.45038405 | 0.0006716 |
| Q921F4 | 72692 | HNRNPLL | 0.450272158 | 0.0025404 |
| Q8R010 | 231872 | AIMP2 | 0.450229834 | 0.0053051 |
| P63028 | 22070 | TPT1 | 0.450153399 | 1.3E-05 |
| P62717 | 76808 | RPL18A | 0.449344931 | 3.403E-05 |
| P24288 | 12035 | BCAT1 | 0.449182041 | 5.28E-05 |
| P14733 | 16906 | LMNB1 | 0.449095097 | 0.0001416 |
| P11031 | 20024 | SUB1 | 0.448963122 | 0.0001844 |
| E9PUE7 | 109934 | ABR | 0.448913607 | 0.0035784 |
| P16045 | 16852 | LGALS1 | 0.44883083 | 1.08E-07 |
| Q3TX72 | 14229 | FKBP5 | 0.448599027 | 2.437E-06 |
| P61021 | 19344 | RAB5B | 0.447825857 | 0.0002905 |
| R4H4V1 | 78891 | SCYL1 | 0.447810548 | 0.0011285 |
| P14869 | 11837 | RPLP0 | 0.447722325 | 2.138E-06 |
| Q9EP72 | 73024 | EMC7 | 0.447484209 | 0.0022638 |
| P29341 | 18458 | PABPC1 | 0.447444728 | 0.0001879 |
| Q9DBG3 | 71770 | AP2B1 | 0.44729153 | 8.367E-05 |
| Q9ERU9 | 19386 | RANBP2 | 0.447272707 | 4.207E-05 |
| Q9D8N0 | 67160 | EEF1G | 0.447060856 | 7.563E-07 |
| P80315 | 12464 | CCT4 | 0.446844539 | 1.554E-05 |
| Q91XI1 | 224907 | DUS3L | 0.446818382 | 0.0004003 |
| O54833 | 13000 | CSNK2A2 | 0.44666744 | 0.000373 |
| Q3TCW5 | 192292 | NRBP1 | 0.44655404 | 0.0004655 |
| Q9D0L7 | 67211 | ARMC10 | 0.44616746 | 0.0007995 |
| O70251 | 55949 | EEF1B2 | 0.446045852 | 0.0001675 |
| E9QLA5 | 70435 | INF2 | 0.445778641 | 0.0001296 |
| Q3TCH7 | 99375 | CUL4A | 0.445775355 | 0.0001401 |
| P10107 | 16952 | ANXA1 | 0.445564311 | 8.01E-07 |
| G3X9U9 | 66437 | FIS1 | 0.44548029 | 4.271E-05 |
| G3UZY2 | 56551 | TXN2 | 0.445431282 | 0.002294 |
| Q9D0R2 | 110960 | TARS | 0.445258775 | 8.886E-07 |
| Q9JJ89 | 108673 | CCDC86 | 0.445248104 | 7.473E-05 |
| B7ZP20 | 11736 | ANKFY1 | 0.445098848 | 1.892E-05 |
| Q8R3R9 | 67199 | PFDN1 | 0.445074211 | 0.0055259 |
| P47738 | 11669 | ALDH2 | 0.444918449 | 4.74E-07 |
| Q9D7V6 | 67973 | MPHOSPH10 | 0.44482895 | 0.0002656 |
| Q9D903 | 69072 | EBNA1BP2 | 0.444570588 | 0.0015852 |
| Q91WN1 | 108671 | DNAJC9 | 0.44451529 | 7.199E-05 |
| A0A0A6YW80 | 74838 | NAA15 | 0.444477127 | 0.0003331 |
| P53811 | 56305 | PITPNB | 0.444445606 | 4.084E-06 |
| Q8BFQ8 | 213350 | PDDC1 | 0.444253612 | 0.0001382 |
| Q3UAX2 | 16956 | LPL | 0.444218125 | 0.0001307 |
| O35855 | 12036 | BCAT2 | 0.444197708 | 0.0002502 |
| B1AT36 | 66997 | PSMD12 | 0.443781174 | 0.0029403 |
| F8VPX1 | 252870 | USP7 | 0.443719552 | 0.0008024 |
| Q02844 | 100503895 | Tpsab1 | 0.443515858 | 0.0079459 |
| F6VQ81 | 66314 | TPD52L2 | 0.443194678 | 0.0002932 |
| Q3THB3 | 76936 | HNRNPM | 0.442668757 | 1.17E-05 |
| A0A068BIU7 | 57315 | WDR46 | 0.442656349 | 0.0003962 |
| Q9WV60 | 56637 | GSK3B | 0.442554001 | 0.001112 |
| Q8K1J6 | 70047 | TRNT1 | 0.442518582 | 8.287E-05 |
| B1AU25 | 26926 | AIFM1 | 0.442400354 | 0.0001168 |
| Q9D7S7 | 68028 | RPL22L1 | 0.442233176 | 1.218E-05 |
| Q8K411 | 69617 | PITRM1 | 0.442206405 | 9.802E-05 |
| P21550 | 13808 | ENO3 | 0.441982591 | 6.483E-06 |
| Q8BU20 | 66046 | NDUFB5 | 0.441919665 | 1.323E-05 |
| P51660 | 15488 | HSD17B4 | 0.441830012 | 3.911E-06 |
| Z4YKM2 | 217149 | CISD3 | 0.441609384 | 9.338E-05 |
| P62242 | 20116 | RPS8 | 0.441487152 | 1.292E-05 |
| Q3TXV1 | 21762 | PSMD2 | 0.441351504 | 2.219E-05 |
| Q60737 | 12995 | CSNK2A1 | 0.440861668 | 0.0001006 |
| Q8K298 | 68743 | ANLN | 0.440566355 | 0.001505 |
| P43275 | 80838 | Hist1h1a | 0.440137068 | 0.0001234 |
| Q8C1E7 | 215210 | tmem120a | 0.440099888 | 7.067E-05 |
| D3YXW1 | 66225 | llph | 0.440071594 | 0.0064809 |
| Q8C570 | 66679 | RAE1 | 0.439563318 | 0.006133 |
| Q6PIP5 | 67429 | NUDCD1 | 0.439234162 | 0.0004834 |
| P62204 | 12313 | CALM1 | 0.43911959 | 4.181E-05 |
| Q9D0R8 | 268490 | LSM12 | 0.439069773 | 0.0096967 |
| Q8C7C4 |  |  | 0.439051998 | 1.811E-05 |
| Q99KG1 | 74326 | HNRNPR | 0.438999603 | 0.0004013 |
| O35215 | 13202 | DDT | 0.438715584 | 0.0007871 |
| Q99JX4 | 98221 | EIF3M | 0.438706457 | 8.398E-06 |
| P16125 | 16832 | LDHB | 0.438637812 | 5.668E-05 |
| Q6ZWZ6 | 20042 | RPS12 | 0.438285537 | 1.736E-05 |
| Q60692 | 19175 | PSMB6 | 0.438266874 | 0.0085245 |
| Z4YL78 | 75786 | CKAP5 | 0.438021207 | 0.0004493 |
| A7VL18 | 56752 | ALDH9A1 | 0.437813568 | 7.121E-06 |
| Q3UK41 | 24116 | NELFA | 0.437811396 | 0.0054259 |
| F6SPK0 | 56228 | UBE2J1 | 0.437767472 | 0.0016686 |
| A2BE93 | 56086 | SET | 0.437598007 | 2.581E-06 |
| Q3UDR2 | 18453 | P4HB | 0.437292712 | 1.268E-07 |
| Q9CQR4 | 66834 | ACOT13 | 0.437208722 | 0.0005157 |
| Q91WQ3 | 107271 | YARS | 0.43707376 | 3.985E-06 |
| Q9D1G1 | 76308 | RAB1B | 0.437066215 | 7.161E-06 |
| Q6P5E4 | 320011 | UGGT1 | 0.436550716 | 2.875E-06 |
| Q8BP47 | 70223 | NARS | 0.436412681 | 1.371E-05 |
| Q99KP6 | 28000 | PRPF19 | 0.436326849 | 7.611E-05 |
| Q9DCW4 | 110826 | ETFB | 0.436232659 | 9.277E-05 |
| P14211 | 12317 | CALR | 0.435909926 | 3.403E-05 |
| Q91V55 | 20103 | RPS5 | 0.434959103 | 3.885E-06 |
| Q99J77 | 94181 | NANS | 0.434939332 | 0.0005915 |
| Q91VC9 | 66092 | GHITM | 0.43464835 | 0.004367 |
| Q9DBH5 | 66890 | LMAN2 | 0.434628254 | 0.0005355 |
| P47199 | 12972 | CRYZ | 0.434615207 | 0.0005968 |
| Q99PL5 | 81910 | RRBP1 | 0.434292593 | 7.974E-06 |
| Q9R1C7 | 56194 | PRPF40A | 0.434190505 | 0.0061824 |
| F8VQC1 | 66661 | SRP72 | 0.434101073 | 1.51E-07 |
| Q3U5I9 | 19079 | PRKAB1 | 0.433889545 | 0.0042538 |
| Q922D4 | 52036 | PPP6R3 | 0.433852218 | 3.787E-06 |
| Q7TMK9 | 56403 | SYNCRIP | 0.433677421 | 2.593E-07 |
| Q5M9N6 | 19981 | RPL37A | 0.433211196 | 0.0058957 |
| Q6PHZ1 | 319195 | RPL17 | 0.433142795 | 2.209E-06 |
| P19182 | 15982 | IFRD1 | 0.432915875 | 0.0001218 |
| P26043 | 19684 | RDX | 0.432814954 | 0.0009359 |
| Q7TT42 |  |  | 0.432735922 | 1.927E-06 |
| Q3UVI9 | 27407 | ABCF2 | 0.432506587 | 5.34E-05 |
| Q91VR5 | 104721 | DDX1 | 0.432505722 | 8.323E-06 |
| E9Q634 | 71602 | MYO1E | 0.432254284 | 0.0008385 |
| Q3U0B0 | 16498 | KCNAB2 | 0.432221898 | 0.0006763 |
| Q9QWZ1 | 19355 | RAD1 | 0.432210589 | 0.0008654 |
| P40124 | 12331 | CAP1 | 0.432196781 | 0.0003543 |
| Q8CCX9 | 27393 | MRPL39 | 0.432101084 | 0.0019001 |
| K7Q751 | 14083 | PTK2 | 0.431863383 | 0.0001326 |
| P54071 | 269951 | IDH2 | 0.431469818 | 4.165E-07 |
| Q6NZM8 | 23971 | PAPSS1 | 0.431393128 | 2.308E-06 |
| O89079 | 59042 | COPE | 0.431340595 | 0.0010648 |
| P51881 | 11740 | SLC25A5 | 0.430727364 | 4.942E-05 |
| Q9CQC7 | 68194 | NDUFB4 | 0.430397742 | 0.0003133 |
| Q8R5L1 | 12261 | C1QBP | 0.430026056 | 1.512E-06 |
| E9QME5 | 94093 | TRIM33 | 0.429856966 | 0.0053715 |
| E0CY49 | 109900 | ASL | 0.429385556 | 8.844E-05 |
| F6RDM4 | 14660 | GLS | 0.428812948 | 0.0034825 |
| Q8BTY3 | 67895 | PPA1 | 0.428684179 | 9.524E-06 |
| B7ZNW0 | 72542 | PGAM5 | 0.428642792 | 0.001587 |
| E9PYI8 | 59025 | USP14 | 0.428609504 | 0.0057653 |
| Q99ME9 | 69237 | GTPBP4 | 0.42847968 | 6.226E-05 |
| P70122 | 66711 | SBDS | 0.428424085 | 0.001312 |
| P18155 | 17768 | mthfd2 | 0.428416385 | 5.81E-05 |
| Q9JJI8 | 67671 | RPL38 | 0.428393364 | 0.0003842 |
| Q64727 | 22330 | VCL | 0.428303284 | 0.0014731 |
| B7ZWC4 | 16004 | IGF2R | 0.428001938 | 0.0011025 |
| Q8C2D1 | 15115 | HARS | 0.427411225 | 0.0003676 |
| Q9CWT6 | 71986 | DDX28 | 0.427261145 | 0.0002117 |
| Q3UIS6 | 21761 | MORF4L1 | 0.426806705 | 0.0004776 |
| Q3TEU8 | 23790 | CORO1C | 0.426170156 | 9.229E-05 |
| Q99KV1 | 67838 | DNAJB11 | 0.426107345 | 2.627E-06 |
| Q9WVG6 | 59035 | CARM1 | 0.426027214 | 0.0001616 |
| Q99K70 | 54170 | RRAGC | 0.425969236 | 0.0001118 |
| P27773 | 14827 | PDIA3 | 0.425625983 | 2.224E-07 |
| P19536 | 12859 | Cox5b | 0.425573983 | 0.0018326 |
| A6PWC3 | 230598 | NRD1 | 0.425534907 | 0.0001981 |
| Q8JZN5 | 229211 | ACAD9 | 0.425195307 | 0.0024763 |
| Q9Z1E4 | 14936 | GYS1 | 0.424814476 | 0.0057032 |
| P56812 | 56330 | PDCD5 | 0.424668242 | 4.417E-05 |
| Q00PI9 | 68693 | HNRNPUL2 | 0.42466674 | 3.728E-06 |
| O35737 | 59013 | HNRNPH1 | 0.42454632 | 1.06E-05 |
| Q8BK67 | 108911 | RCC2 | 0.42447228 | 0.0002044 |
| Q3THU8 | 18674 | SLC25A3 | 0.42443828 | 0.000448 |
| Q3TI63 | 56440 | SNX1 | 0.423979448 | 4.709E-05 |
| Q8BIP0 | 226539 | DARS2 | 0.423906988 | 0.0007063 |
| P99029 | 54683 | PRDX5 | 0.423369159 | 1.348E-05 |
| Q8BK72 | 218506 | MRPS27 | 0.423031041 | 4.141E-05 |
| Q3U6U7 | 22375 | WARS | 0.422734393 | 9.1E-05 |
| Q9R0X4 | 56360 | ACOT9 | 0.42269008 | 2.04E-05 |
| Q8C140 | 50799 | SLC25A13 | 0.422548264 | 0.0019299 |
| Q9CR41 | 67693 | HYPK | 0.422453961 | 0.0080665 |
| Q8C1Y3 | 14958 | H1F0 | 0.42244038 | 7.197E-07 |
| P58252 | 13629 | EEF2 | 0.421274367 | 4.799E-06 |
| B2RXU2 | 66580 | ESF1 | 0.421120979 | 3.363E-05 |
| Q03145 | 13836 | EPHA2 | 0.421108853 | 1.334E-05 |
| P10630 | 13682 | EIF4A2 | 0.420968802 | 0.0002484 |
| Q91WK1 | 66701 | spryd4 | 0.420755061 | 0.0005266 |
| A2ALF0 | 68598 | DNAJC8 | 0.420717429 | 4.433E-06 |
| Q9D0I9 | 104458 | RARS | 0.420705544 | 2.6E-07 |
| Q99KI0 | 11429 | ACO2 | 0.420235516 | 4.958E-07 |
| Q1WWK3 | 56702 | HIST1H1B | 0.420046813 | 1.131E-05 |
| Q9D1P4 | 66917 | CHORDC1 | 0.420002237 | 2.025E-05 |
| O70591 | 18637 | PFDN2 | 0.41995427 | 0.0006808 |
| Q8CH72 | 69807 | TRIM32 | 0.419850565 | 0.0009922 |
| Q8CI86 | 18797 | plcb3 | 0.419594758 | 4.493E-05 |
| P54822 | 11564 | ADSL | 0.419551153 | 0.0007352 |
| Q9DCA5 | 67832 | BRIX1 | 0.418722181 | 1.867E-05 |
| A3KMJ8 | 22327 | VBP1 | 0.418711714 | 4.925E-05 |
| P52503 | 407785 | NDUFS6 | 0.418635766 | 0.0004974 |
| E9QAZ2 | 100039748 | Gm10020 | 0.418385296 | 0.0003714 |
| Q62165 | 13138 | DAG1 | 0.418270635 | 0.0013783 |
| Q8BUM1 | 230908 | TARDBP | 0.41820148 | 3.592E-07 |
| G3X956 | 114741 | Supt16 | 0.418098985 | 1.164E-05 |
| Q8BH95 | 93747 | ECHS1 | 0.418049848 | 6.805E-05 |
| P59325 | 217869 | EIF5 | 0.417872559 | 1.079E-05 |
| Q66L45 | 227695 | SPOUT1 | 0.417627411 | 0.0001265 |
| Q3UAG2 | 110208 | PGD | 0.417333495 | 1.961E-06 |
| P51410 | 20005 | RPL9 | 0.417324895 | 0.0004125 |
| Q9QYR9 | 171210 | ACOT2 | 0.417279773 | 1.129E-05 |
| Q3THQ5 | 20867 | STIP1 | 0.417168467 | 0.0001627 |
| P63005 | 18472 | PAFAH1B1 | 0.417093816 | 0.0041229 |
| O88811 | 56324 | STAM2 | 0.417041464 | 0.0016792 |
| O35387 | 23897 | HAX1 | 0.416607303 | 0.0001028 |
| Q9D554 | 75062 | SF3A3 | 0.416471861 | 0.0011441 |
| Q05186 | 19672 | RCN1 | 0.41636909 | 1.773E-05 |
| Q99KD6 | 20463 | COX7A2L | 0.415934258 | 0.0010219 |
| P21619 | 16907 | LMNB2 | 0.415933629 | 8.594E-05 |
| P50518 | 11973 | ATP6V1E1 | 0.41579819 | 5.182E-05 |
| Q8K2C6 | 68346 | SIRT5 | 0.414627573 | 0.002486 |
| Q8VIJ6 | 71514 | SFPQ | 0.414398544 | 2.999E-05 |
| P62137 | 19045 | PPP1CA | 0.414343968 | 0.0005932 |
| Q9CXW2 | 64655 | MRPS22 | 0.41431075 | 6.243E-05 |
| P61358 | 19942 | RPL27 | 0.414296141 | 0.0001242 |
| O09131 | 14873 | GSTO1 | 0.413835742 | 0.0002083 |
| Q3THZ8 | 54342 | GNPNAT1 | 0.413350263 | 8.566E-06 |
| E9QKV6 | 17925 | MYO9B | 0.413278127 | 0.0024045 |
| P58044 | 319554 | IDI1 | 0.413213409 | 0.0005252 |
| Q3UJW9 | 58810 | AKR1A1 | 0.412891842 | 9.093E-05 |
| Q3UGS4 | 192173 | fam195b | 0.412869407 | 0.0004736 |
| P09671 | 20656 | SOD2 | 0.412165375 | 0.0007587 |
| P61089 | 93765 | UBE2N | 0.410758054 | 0.0013605 |
| Q3TE45 | 67680 | SDHB | 0.410699757 | 0.00038 |
| Q3U6Y9 | 12491 | CD36 | 0.410438701 | 0.0004273 |
| Q68FC6 | 72515 | WDR43 | 0.410361111 | 0.001737 |
| Q61656 | 13207 | DDX5 | 0.409923295 | 0.0011278 |
| Q9D0J2 | 66043 | ATP5D | 0.409620371 | 4.56E-05 |
| A2A513 | 16661 | KRT10 | 0.409008439 | 0.0004563 |
| G3UXZ5 | 19186 | PSME1 | 0.408755766 | 1.482E-05 |
| Q3TEN9 | 14933 | gk | 0.408456504 | 0.0029955 |
| Q99JF8 | 101739 | PSIP1 | 0.407555705 | 3.012E-05 |
| Q76MZ3 | 51792 | PPP2R1A | 0.407496016 | 0.0042945 |
| P63325 | 67097 | RPS10 | 0.407438551 | 0.0001339 |
| Q9CQJ2 | 68845 | PIH1D1 | 0.407326231 | 0.0033879 |
| Q3TD51 | 233489 | PICALM | 0.407210673 | 0.0038538 |
| P62900 | 114641 | RPL31 | 0.406586483 | 7.48E-06 |
| Q3UF82 | 26413 | MAPK1 | 0.406162468 | 0.0015994 |
| P62889 | 19946 | RPL30 | 0.405926318 | 0.0001718 |
| P63101 | 22631 | YWHAZ | 0.40586356 | 8.248E-07 |
| P97770 | 14911 | THUMPD3 | 0.405623739 | 0.0006582 |
| Q9Z2Y8 | 114863 | PROSC | 0.405585377 | 0.0002103 |
| Q8R5H1 | 14479 | USP15 | 0.4055753 | 0.0016086 |
| Q7TMH5 | 68537 | MRPL13 | 0.404937733 | 0.003107 |
| G3X9T8 | 12870 | CP | 0.404931585 | 0.0007181 |
| Q80UZ2 | 231452 | SDAD1 | 0.404725378 | 0.0029884 |
| Q99M31 | 50497 | HSPA14 | 0.404710572 | 0.0001298 |
| D3Z4V1 | 53317 | plrg1 | 0.404024746 | 0.0057538 |
| O08709 | 11758 | PRDX6 | 0.403358885 | 5.214E-06 |
| Q3TN29 | 71701 | PNPT1 | 0.40303664 | 0.0009335 |
| P46460 | 18195 | NSF | 0.402728004 | 0.0001976 |
| P97352 | 20196 | S100A13 | 0.402587613 | 0.0010032 |
| Q9WUD1 | 56424 | STUB1 | 0.40136286 | 0.0062257 |
| P45377 | 14187 | Akr1b8 | 0.400830751 | 0.0014819 |
| Q80X50 | 74383 | UBAP2L | 0.400326366 | 0.0001444 |
| P35564 | 12330 | CANX | 0.400218443 | 3.333E-09 |
| Q9D020 | 107569 | nt5c3 | 0.400031305 | 0.0011249 |
| Q9CQN7 | 107733 | MRPL41 | 0.399985534 | 0.00051 |
| O08749 | 13382 | DLD | 0.399490896 | 7.058E-05 |
| Q99020 | 15384 | HNRNPAB | 0.399212049 | 2.254E-06 |
| Q9WVE8 | 23970 | PACSIN2 | 0.399011016 | 0.0030711 |
| Q9R1P0 | 26441 | PSMA4 | 0.398865792 | 2.548E-05 |
| Q99KF1 | 67511 | TMED9 | 0.398420128 | 0.0010766 |
| O88653 | 56692 | LAMTOR3 | 0.398201524 | 1.718E-05 |
| Q6P8X1 | 72183 | SNX6 | 0.39815197 | 2.432E-05 |
| Q00422 | 14390 | GABPA | 0.398129416 | 0.0033514 |
| Q9CWU9 | 69736 | NUP37 | 0.398092704 | 0.0005933 |
| Q8CHC7 | 26936 | MPRIP | 0.397341801 | 0.0008338 |
| Q9DBU8 | 55946 | AP3M1 | 0.396608194 | 0.0002213 |
| Q99L47 | 70356 | ST13 | 0.396165863 | 0.0021248 |
| Q80VD1 | 68215 | FAM98B | 0.396070889 | 0.0034725 |
| Q06138 | 12283 | CAB39 | 0.396061235 | 0.0016797 |
| G3X9B1 | 217995 | HEATR1 | 0.395942331 | 0.0002176 |
| P62830 | 65019 | RPL23 | 0.395412622 | 0.0002225 |
| P97825 | 15374 | HN1 | 0.395385239 | 0.001106 |
| Q9D2R0 | 78894 | AACS | 0.395316087 | 6.14E-05 |
| E9PYC6 | 23986 | ECI2 | 0.395290168 | 0.0062959 |
| Q05BH6 | 21453 | TCOF1 | 0.395219933 | 0.0060925 |
| Q78JE5 | 71999 | FBXO22 | 0.395066148 | 0.00019 |
| Q9WUL7 | 56350 | ARL3 | 0.394920281 | 8.616E-05 |
| Z4YKV1 | 14683 | GNAS | 0.39481121 | 0.0004522 |
| Q04207 | 19697 | RELA | 0.394709768 | 5.705E-05 |
| Q60865 | 53872 | CAPRIN1 | 0.394704742 | 1.613E-05 |
| A2ATT5 | 53893 | NUDT5 | 0.394116207 | 0.0003624 |
| Q8C838 | 237858 | TUSC5 | 0.393732678 | 0.0013326 |
| Q9D172 | 28295 | D10Jhu81e | 0.393721765 | 0.0012427 |
| Q8VCN5 | 107869 | CTH | 0.393614438 | 9.21E-05 |
| P68510 | 22629 | YWHAH | 0.393391296 | 5.314E-05 |
| Q3TN31 | 26444 | PSMA7 | 0.392953345 | 8.48E-05 |
| P05201 | 14718 | GOT1 | 0.392885973 | 0.0002779 |
| Q9CQZ5 | 67130 | NDUFA6 | 0.392701347 | 1.793E-05 |
| Q3V212 | 76457 | CCDC134 | 0.392523213 | 0.0079648 |
| Q9ERE7 | 67943 | MESDC2 | 0.392395272 | 0.00049 |
| Q9CQE8 | 68045 | 2700060E02Rik | 0.392246859 | 0.0024829 |
| Q6P069 | 109552 | SRI | 0.39192085 | 0.000214 |
| P26350 | 19231 | PTMA | 0.391522656 | 7.224E-05 |
| Q9R1P3 | 26445 | PSMB2 | 0.389884244 | 7.627E-05 |
| Q62159 | 11853 | RHOC | 0.389263924 | 2.316E-05 |
| Q8BG51 | 59040 | RHOT1 | 0.389226213 | 1.356E-05 |
| Q501J6 | 67040 | DDX17 | 0.389018297 | 0.0017965 |
| Q99JX6 | 11749 | ANXA6 | 0.388902152 | 0.0038623 |
| Q6PGB6 | 72117 | NAA50 | 0.388507321 | 0.0007909 |
| Q684I8 | 15929 | IDH3G | 0.388384383 | 0.0040188 |
| O35655 | 237178 | PPEF1 | 0.387935923 | 0.0024274 |
| H3BKK2 | 51944 | KNSTRN | 0.387720014 | 7.746E-05 |
| Q8WTY4 | 109006 | CIAPIN1 | 0.387236779 | 0.001068 |
| D3YVW2 | 73124 | GOLIM4 | 0.386654165 | 0.0001116 |
| Q9DBL7 | 71743 | COASY | 0.38597919 | 0.0005749 |
| P14131 | 20055 | RPS16 | 0.385471746 | 7.611E-06 |
| P99024 | 22154 | TUBB5 | 0.384537502 | 2.272E-06 |
| Q5DU34 | 105522 | ANKRD28 | 0.384432005 | 0.0071186 |
| Q9D2Y4 | 74568 | MLKL | 0.384427445 | 0.001632 |
| Q91VM5 | 19656 | RBMXL1 | 0.384305332 | 7.929E-06 |
| Q8R050 | 14852 | GSPT1 | 0.383781406 | 0.0002347 |
| O08997 | 11927 | ATOX1 | 0.383511579 | 0.0003039 |
| P62911 | 19951 | RPL32 | 0.382416682 | 2.923E-05 |
| A0A068BFR3 | 19326 | RAB11B | 0.382196338 | 2.118E-05 |
| P26516 | 17463 | PSMD7 | 0.382084609 | 0.0003939 |
| Q60973 | 245688 | RBBP7 | 0.381921924 | 0.000429 |
| O35381 | 11737 | ANP32A | 0.381717934 | 3.783E-06 |
| P62331 | 11845 | ARF6 | 0.381665055 | 0.0001545 |
| Q9CQB4 | 67530 | Uqcrb | 0.38159065 | 7.385E-05 |
| O08599 | 20910 | STXBP1 | 0.380519754 | 0.0008585 |
| O08917 | 14251 | FLOT1 | 0.380172566 | 0.009839 |
| A0A023T672 | 60365 | RBM8A | 0.37970804 | 0.0010767 |
| P62320 | 67332 | SNRPD3 | 0.379396412 | 4.38E-06 |
| Q9D1M4 | 66143 | EEF1E1 | 0.378592948 | 5.265E-05 |
| Q8K0C4 | 13121 | CYP51 | 0.378318273 | 3.074E-05 |
| Q9JIX0 | 223527 | ENY2 | 0.377113302 | 0.0017879 |
| Q9D024 | 67163 | CCDC47 | 0.377002031 | 0.008849 |
| Q9DB15 | 56282 | MRPL12 | 0.376910357 | 6.397E-05 |
| Q2YDW1 |  |  | 0.375833537 | 0.000675 |
| Q9D8V0 | 14950 | H13 | 0.375271339 | 0.0009734 |
| P51859 | 15191 | HDGF | 0.3751443 | 2.584E-05 |
| G3UYI5 |  |  | 0.375012441 | 0.0001626 |
| P62270 | 20084 | RPS18 | 0.374595134 | 1.252E-05 |
| Q6IRU2 | 326618 | TPM4 | 0.374146703 | 1.927E-06 |
| P28658 | 54138 | ATXN10 | 0.37404396 | 7.739E-05 |
| Q9CZD3 | 353172 | GARS | 0.37390261 | 5.282E-05 |
| Q3TB79 | 12333 | CAPN1 | 0.372779862 | 8.663E-05 |
| Q3US29 | 110196 | FDPS | 0.372138412 | 1.482E-05 |
| Q9CVT6 | 67105 | TIMM21 | 0.371941835 | 0.0004608 |
| Q3U7I9 | 13033 | CTSD | 0.371690347 | 3.808E-05 |
| P10711 | 21399 | TCEA1 | 0.371597827 | 7.425E-05 |
| Q9R062 | 27357 | Gyg | 0.370661035 | 0.0002336 |
| F6V084 | 72736 | TMX1 | 0.36995407 | 0.0084688 |
| Q3U7A6 | 20853 | STAU1 | 0.369952437 | 0.0033572 |
| Q3U5R8 | 55980 | IMPA1 | 0.369253689 | 0.000176 |
| Q3TK27 | 30877 | GNL3 | 0.368985486 | 0.0002124 |
| Q921J2 | 19744 | RHEB | 0.368819812 | 0.0072867 |
| Q8JZX4 | 76938 | RBM17 | 0.368751035 | 0.0001855 |
| E9PYT3 | 109168 | ATL3 | 0.368675579 | 0.0007307 |
| O54692 | 26951 | ZW10 | 0.368595983 | 0.0009956 |
| D3Z2F7 | 56378 | ARPC3 | 0.366499039 | 5.473E-05 |
| Q9D5V5 | 75717 | CUL5 | 0.366310057 | 0.0010175 |
| Q9CYH6 | 59014 | RRS1 | 0.365457882 | 0.0004246 |
| Q99L45 | 67204 | EIF2S2 | 0.364376292 | 7.056E-05 |
| Q05C51 | 69724 | RNASEH2A | 0.364173963 | 0.0042864 |
| P61982 | 22628 | YWHAG | 0.363965074 | 0.0004649 |
| Q9CZU6 | 12974 | CS | 0.362652993 | 0.0004713 |
| A2AU61 | 19383 | RALY | 0.362453369 | 0.0014468 |
| Q8C2Q8 | 11949 | ATP5C1 | 0.36182422 | 0.0018503 |
| E9PXV3 | 108058 | CAMK2D | 0.360999253 | 0.0023714 |
| O55135 | 16418 | EIF6 | 0.36034958 | 4.473E-07 |
| B1ATZ0 | 15239 | HGS | 0.360166583 | 0.0006724 |
| Q3TMX5 | 74840 | MANF | 0.359773698 | 1.037E-05 |
| Q99LC3 | 67273 | NDUFA10 | 0.358949555 | 0.0003451 |
| A2ATP5 | 17876 | MYEF2 | 0.358639599 | 0.0012056 |
| Q8C0Z3 | 69207 | SRSF11 | 0.358598547 | 0.003906 |
| A2AP78 | 15354 | HMGB3 | 0.358234813 | 0.0002214 |
| D3YWK1 | 74781 | WIPI2 | 0.357150996 | 3.663E-05 |
| Q91V76 | 70984 | 4931406C07Rik | 0.357004719 | 0.0015127 |
| O88844 | 15926 | idh1 | 0.356601111 | 0.0003654 |
| P61164 | 54130 | ACTR1A | 0.356486299 | 0.0038652 |
| Q5SW88 | 19324 | RAB1A | 0.356460722 | 7.9E-05 |
| Q3UK38 | 66254 | DIMT1 | 0.356119862 | 0.0001402 |
| Q99LC2 | 67337 | CSTF1 | 0.355471294 | 0.0001296 |
| Q9DBP5 | 66588 | CMPK1 | 0.353734734 | 1.582E-05 |
| Q62446 | 30795 | FKBP3 | 0.35314499 | 0.0003409 |
| A2AFI3 | 19655 | RBMX | 0.351037103 | 0.0097366 |
| Q9WV98 | 30056 | TIMM9 | 0.3493354 | 0.0023131 |
| Q5EBG5 | 27176 | RPL7A | 0.349086913 | 3.931E-05 |
| Q61768 | 16573 | KIF5B | 0.347305897 | 0.0001971 |
| Q9CQF3 | 68219 | NUDT21 | 0.347169332 | 0.0002325 |
| P34022 | 19385 | RANBP1 | 0.346556624 | 0.0001757 |
| Q4V9X9 | 268449 | RPL23A | 0.346051079 | 0.0001897 |
| G3UZ26 | 20425 | SHMT1 | 0.346025224 | 0.0001087 |
| Q8CH18 | 67500 | CCAR1 | 0.344583914 | 0.0006353 |
| Q8CGZ0 | 27967 | CHERP | 0.343838947 | 0.0003598 |
| P45878 | 14227 | FKBP2 | 0.343076306 | 0.0002051 |
| Q8R1V4 | 103694 | TMED4 | 0.342851138 | 0.0002972 |
| Q9WV55 | 30960 | VAPA | 0.34049951 | 4.609E-05 |
| O70435 | 19167 | PSMA3 | 0.339274662 | 0.0007744 |
| Q61686 | 12419 | CBX5 | 0.338705028 | 0.0009053 |
| A2BFF8 | 13427 | DYNC1I2 | 0.337643746 | 1.11E-05 |
| Q3UBB0 | 75612 | GNS | 0.335400262 | 0.0001712 |
| P14148 | 19989 | RPL7 | 0.335391526 | 0.0002534 |
| P09528 | 14319 | FTH1 | 0.335218452 | 1.587E-08 |
| Q921I9 | 109075 | EXOSC4 | 0.334505477 | 0.0006578 |
| B2RXR6 | 329154 | ANKRD44 | 0.332543011 | 0.001309 |
| Q9Z204 | 15381 | HNRNPC | 0.332228477 | 1.51E-05 |
| Q6NZN9 | 109079 | SEPHS1 | 0.331973387 | 0.0004916 |
| P09405 | 17975 | NCL | 0.331546604 | 5.021E-05 |
| O35864 | 26754 | COPS5 | 0.330575425 | 0.0034584 |
| Q4FE56 | 22284 | USP9X | 0.329919647 | 0.0024155 |
| Q9Z2G9 | 53415 | HTATIP2 | 0.329240356 | 0.0002357 |
| Q99P31 | 66245 | HSPBP1 | 0.329221127 | 0.0001927 |
| O70252 | 15369 | HMOX2 | 0.328572465 | 0.0001439 |
| Q3U617 | 57296 | PSMD8 | 0.326770336 | 0.0006453 |
| E9Q1M6 | 108857 | ANKHD1 | 0.326744574 | 0.008905 |
| P06151 | 16828 | LDHA | 0.325358783 | 9.682E-05 |
| Q3TDQ1 | 68292 | STT3B | 0.324319671 | 1.177E-05 |
| I7HLV2 | 110954 | RPL10 | 0.32300353 | 1.631E-05 |
| P63330 | 19052 | PPP2CA | 0.32246454 | 2.827E-05 |
| P47915 | 19944 | RPL29 | 0.322406653 | 0.0023764 |
| Q3U9H4 | 16430 | STT3A | 0.321343538 | 1.2E-05 |
| Q9D828 | 21787 | TFG | 0.320905736 | 0.0001846 |
| Q9DAU1 | 72029 | CNPY3 | 0.320789595 | 7.7E-05 |
| Q61074 | 14208 | PPM1G | 0.320196509 | 0.0040795 |
| P12265 | 110006 | GUSB | 0.319661897 | 0.0001052 |
| Q61425 | 15107 | HADH | 0.318851913 | 0.0002808 |
| Q8VCB1 | 72787 | NDC1 | 0.31867869 | 0.0046322 |
| A3KGQ6 |  |  | 0.317166442 | 1.39E-05 |
| Q9CZ04 | 26894 | COPS7A | 0.316584561 | 0.0001992 |
| Q9CQS8 | 66212 | SEC61B | 0.315821129 | 0.0001517 |
| Q3TFA9 | 50875 | TMOD3 | 0.31580962 | 0.0001012 |
| Q8BGZ7 | 109052 | KRT75 | 0.315663612 | 2.458E-05 |
| Q80W54 | 230709 | ZMPSTE24 | 0.315521501 | 0.002095 |
| Q9CQU3 | 67830 | RER1 | 0.313397211 | 9.059E-05 |
| Q78RK2 | 170460 | STARD5 | 0.312854271 | 0.0001158 |
| Q3TDR0 | 70750 | KDSR | 0.311891292 | 0.0009399 |
| A2RRJ4 | 72322 | XPO5 | 0.311866516 | 0.0011279 |
| P17047 | 16784 | LAMP2 | 0.311409419 | 0.0001334 |
| P61079 | 66105 | UBE2D3 | 0.311407517 | 0.0009828 |
| P33174 | 16571 | kif4 | 0.310563922 | 1.03E-05 |
| Q3TEL0 | 19063 | PPT1 | 0.310480443 | 0.0006267 |
| A1L0U3 | 319151 | HIST1H3E | 0.309743848 | 0.0030802 |
| D3Z041 | 14081 | ACSL1 | 0.309646585 | 0.0080735 |
| Q62425 | 17992 | NDUFA4 | 0.309421826 | 0.0001676 |
| P19253 | 22121 | RPL13A | 0.30825404 | 7.34E-06 |
| Q9WUU7 | 64138 | CTSZ | 0.307094812 | 0.0002648 |
| Q9CR59 | 102060 | GADD45GIP1 | 0.306659995 | 0.0001508 |
| Q3TL79 | 217737 | AHSA1 | 0.30631005 | 3.013E-06 |
| Q9QZD9 | 54709 | EIF3I | 0.305833513 | 0.0001562 |
| Q8VE47 | 66663 | UBA5 | 0.305580833 | 0.0005104 |
| P48962 | 11739 | SLC25A4 | 0.305244469 | 0.000111 |
| P29595 | 18002 | NEDD8 | 0.30521189 | 6.508E-05 |
| P14685 | 22123 | PSMD3 | 0.304960462 | 0.0001117 |
| P35700 | 18477 | PRDX1 | 0.304656205 | 9.941E-06 |
| Q8BIW1 | 229589 | PRUNE1 | 0.303483532 | 0.0001413 |
| P62862 | 14109 | FAU | 0.303017403 | 3.456E-05 |
| P49615 | 12568 | CDK5 | 0.3024006 | 0.0081197 |
| Q6PB66 | 72416 | LRPPRC | 0.301559756 | 1.68E-05 |
| Q6P5B0 | 107094 | RRP12 | 0.301229493 | 0.001428 |
| Q9DC16 | 67458 | ERGIC1 | 0.300764041 | 0.004279 |
| Q3U3C2 | 67963 | NPC2 | 0.300748098 | 0.0044415 |
| P62869 | 67673 | TCEB2 | 0.300746434 | 3.755E-05 |
| P10126 | 13627 | EEF1A1 | 0.300137963 | 7.026E-07 |
| P62827 | 19384 | RAN | 0.299846992 | 5.846E-06 |
| G3UVU2 | 20222 | SF3A2 | 0.299680221 | 5.036E-06 |
| Q6ZWV7 | 66489 | RPL35 | 0.299604063 | 0.0008152 |
| P24472 | 14860 | GSTA4 | 0.297822884 | 0.0035699 |
| A0JNY7 | 13690 | EIF4G2 | 0.29496043 | 4.451E-05 |
| P62702 | 20102 | RPS4X | 0.29476563 | 6.505E-05 |
| Q7TMM9 | 22151 | TUBB2A | 0.294733806 | 0.0002832 |
| D3YZ09 | 68147 | GAR1 | 0.293684865 | 0.0001905 |
| P45591 | 12632 | CFL2 | 0.292300384 | 0.002795 |
| E9PWK1 | 13849 | EPHX1 | 0.292263315 | 0.0004794 |
| Q05DE0 | 228998 | ARFGAP1 | 0.291920313 | 0.0032226 |
| Q3TJN6 | 69178 | SNX5 | 0.291540958 | 0.0003925 |
| F8WHV1 | 68523 | FAM96B | 0.290659485 | 1.863E-05 |
| Q8BX70 | 320528 | VPS13C | 0.29015442 | 0.007528 |
| Q5D098 | 19177 | PSMB7 | 0.289767418 | 0.0002829 |
| Q9CWW6 | 69713 | PIN4 | 0.28884553 | 0.0002312 |
| Q3ULI5 | 52040 | PPP1R10 | 0.288466675 | 0.0002147 |
| Q3UMR5 | 215999 | MCU | 0.285682888 | 0.0064556 |
| P84084 | 11844 | ARF5 | 0.284162566 | 0.0001413 |
| B1AWZ5 | 66536 | NIPSNAP3B | 0.281628918 | 0.0011857 |
| B1ARA5 | 19941 | RPL26 | 0.281377892 | 0.0014774 |
| G3UWG1 |  |  | 0.280421007 | 2.854E-05 |
| Q8CFZ0 | 22196 | UBE2I | 0.279877116 | 0.0003287 |
| Q60749 | 20218 | KHDRBS1 | 0.278500543 | 0.0004886 |
| O54962 | 23825 | BANF1 | 0.275094221 | 4.522E-06 |
| P41105 | 19943 | RPL28 | 0.275086748 | 8.169E-05 |
| Q6P7V9 | 70099 | SMC4 | 0.274483847 | 3.919E-05 |
| Q3TLJ5 | 80280 | CDK5RAP3 | 0.273681749 | 0.0011583 |
| Q8BP67 | 68193 | RPL24 | 0.273570009 | 0.0002981 |
| O88848 | 56297 | ARL6 | 0.270708313 | 6.329E-05 |
| Q3UDM8 | 66646 | RPE | 0.268209655 | 5.39E-05 |
| Q9JLZ3 | 11992 | AUH | 0.267030604 | 0.0045547 |
| B8JJI4 | 224630 | BNIP1 | 0.265997913 | 0.0005467 |
| Q6NVC2 | 21843 | TIAL1 | 0.264626481 | 0.0004835 |
| Q921H9 | 69893 | COA7 | 0.263970211 | 0.0009426 |
| Q9CZX7 | 72519 | TMEM55A | 0.262619103 | 0.0023549 |
| Q4FZG9 | 407790 | NDUFA4L2 | 0.262548922 | 0.0006559 |
| Q9QZQ8 | 26914 | h2afy | 0.262528176 | 4.864E-05 |
| Q9D8M4 | 66229 | RPL7L1 | 0.26108967 | 0.000136 |
| Q9Z2I9 | 20916 | SUCLA2 | 0.259894039 | 0.000133 |
| E9Q4B9 | 59046 | ARPP19 | 0.258410332 | 0.0014491 |
| P41241 | 12988 | CSK | 0.254640292 | 0.0007937 |
| P16254 | 20813 | SRP14 | 0.253974672 | 0.0016412 |
| Q6ZWQ7 | 76687 | SPCS3 | 0.253807576 | 0.0001121 |
| P07091 | 20198 | S100A4 | 0.252391832 | 0.0003107 |
| Q99MV1 | 83561 | TDRD1 | 0.251454619 | 0.0001533 |
| Q8R5J9 | 65106 | ARL6IP5 | 0.25089494 | 0.0001094 |
| Q9CR16 | 67738 | PPID | 0.250212525 | 2.549E-06 |
| P20060 | 15212 | HEXB | 0.25019192 | 0.0016089 |
| Q9CPN9 | 67373 | 2210010C04Rik | 0.249925771 | 0.0001326 |
| Q8C2T9 | 24010 | IK | 0.247996147 | 9.182E-05 |
| Q8BPS5 | 20501 | SLC16A1 | 0.247038845 | 0.0003523 |
| Q91YN9 | 213539 | BAG2 | 0.246602778 | 3.458E-05 |
| P48428 | 21371 | TBCA | 0.24456965 | 0.0003998 |
| Q9CXW3 | 12301 | CACYBP | 0.243891578 | 0.0001031 |
| Q3THE6 |  |  | 0.243566626 | 0.0007784 |
| Q8BMJ3 | 66235 | EIF1AX | 0.243096022 | 0.0007998 |
| D3Z6S1 | 68796 | TMEM214 | 0.242979722 | 0.0001711 |
| Q3TWW8 | 67996 | SRSF6 | 0.242485408 | 1.953E-05 |
| Q9CQI6 | 72042 | COTL1 | 0.242476976 | 0.000609 |
| Q9WUV0 | 26429 | ORC5 | 0.241975505 | 0.0003655 |
| P04104 | 16678 | KRT1 | 0.241596347 | 0.0002903 |
| P08207 | 20194 | S100A10 | 0.240903301 | 1.499E-05 |
| Q3U9U5 | 27061 | BCAP31 | 0.240445779 | 0.0014163 |
| P24369 | 19035 | PPIB | 0.240168422 | 1.815E-05 |
| Q9CQC6 | 66882 | BZW1 | 0.239962724 | 2.542E-06 |
| Q3THB4 | 16828 | LDHA | 0.239057756 | 1.444E-06 |
| P27661 | 15270 | H2AFX | 0.233486516 | 4.841E-05 |
| Q9D6N1 | 71934 | Car13 | 0.233112245 | 4.797E-05 |
| A2AQ17 | 69702 | NDUFAF1 | 0.23164498 | 3.456E-08 |
| P07141 | 12977 | CSF1 | 0.230413902 | 0.0002592 |
| Q9CVB6 | 76709 | ARPC2 | 0.229911502 | 6.772E-06 |
| B7ZCU2 | 11308 | ABI1 | 0.229343425 | 6.87E-05 |
| Q9CXR1 | 66375 | DHRS7 | 0.226549616 | 0.0001455 |
| Q8BMD8 | 229731 | SLC25A24 | 0.223696005 | 0.0001924 |
| H3BKE1 | 269437 | PLCH1 | 0.220004156 | 4.169E-06 |
| F6SVV1 | 670565 | Gm9493 | 0.218523283 | 1.873E-05 |
| P41731 | 12512 | CD63 | 0.218423975 | 4.24E-05 |
| Q91VA7 | 170718 | IDH3B | 0.217178793 | 4.701E-07 |
| Q8BKE6 | 77951 | CYP20A1 | 0.216791298 | 0.0033492 |
| Q8CD09 | 65970 | LIMA1 | 0.216527731 | 5.339E-05 |
| Q80UU6 | 72075 | OGFR | 0.211813237 | 0.0003725 |
| P61750 | 11843 | ARF4 | 0.211497458 | 0.00102 |
| P54923 | 11544 | ADPRH | 0.211408684 | 0.0036096 |
| Q3UM18 | 224092 | LSG1 | 0.209901443 | 5.113E-07 |
| Q3THA6 | 225027 | SRSF7 | 0.209324283 | 0.0005082 |
| Q8CGC6 | 68272 | RBM28 | 0.208900267 | 0.0001353 |
| Q922Q1 | 67247 | MARC2 | 0.207241993 | 0.0003696 |
| D3YXP6 | 68603 | pmvk | 0.206934364 | 1.204E-06 |
| Q9CRS5 | 14030 | EWSR1 | 0.206543356 | 0.0005092 |
| P62281 | 27207 | RPS11 | 0.205662314 | 1.44E-05 |
| Q8BKT8 | 73738 | HAUS7 | 0.205636695 | 0.0002842 |
| Q3TEA8 | 15441 | HP1BP3 | 0.203650161 | 8.034E-05 |
| Q3B7Z2 | 76303 | OSBP | 0.203603311 | 3.136E-06 |
| P62852 | 75617 | RPS25 | 0.20158991 | 4.605E-08 |
| F8WHU7 | 20842 | STAG1 | 0.200045709 | 2.054E-05 |
| Q9CPT4 | 28106 | MYDGF | 0.197878173 | 5.214E-05 |
| H3BLI9 | 21807 | TSC22D1 | 0.195371019 | 1.233E-06 |
| P97315 | 13007 | CSRP1 | 0.194851612 | 0.000207 |
| P10639 | 22166 | Txn1 | 0.194048183 | 1.962E-05 |
| Q3TJD4 | 11950 | ATP5F1 | 0.191100776 | 8.266E-07 |
| D3YVV7 | 384569 | NOVA2 | 0.188795699 | 9.389E-05 |
| P61082 | 22192 | UBE2M | 0.187102287 | 3.296E-05 |
| F6YQT7 | 320355 | LIPI | 0.18648961 | 2.316E-05 |
| G5E8G0 | 11991 | HNRNPD | 0.18534791 | 2.606E-07 |
| Q9D1H8 | 68499 | MRPL53 | 0.185014998 | 0.0001347 |
| Q3U781 | 20383 | SRSF3 | 0.183258966 | 3.757E-06 |
| Q9D2M8 | 70620 | UBE2V2 | 0.183205529 | 0.0002028 |
| E9QKL6 | 15951 | Ifi204 | 0.179230301 | 3.566E-05 |
| Q9CZX8 | 20085 | RPS19 | 0.177652977 | 3.466E-07 |
| D3Z0F3 | 21780 | TFAM | 0.177278839 | 3.628E-06 |
| P62245 | 267019 | rps15a | 0.176232544 | 1.208E-06 |
| F6SFF5 | 67914 | COQ9 | 0.174668402 | 3.548E-05 |
| Q569Z6 | 230753 | THRAP3 | 0.174340494 | 0.0007311 |
| Q3TL33 | 12321 | CALU | 0.171026942 | 0.0006029 |
| Q3UJR8 | 218490 | BTF3 | 0.168054458 | 0.0001356 |
| Q3U449 | 23827 | BPNT1 | 0.167930399 | 3.393E-06 |
| P55264 | 11534 | ADK | 0.167648292 | 9.292E-05 |
| Q9CPQ1 | 12864 | COX6C | 0.166958751 | 0.0001647 |
| P50637 | 12257 | TSPO | 0.163743979 | 3.419E-05 |
| Q9D1R9 | 68436 | RPL34 | 0.160696 | 8.366E-06 |
| A0A087WQD1 | 17973 | NCK1 | 0.157231335 | 2.414E-05 |
| Q91VG6 | 66075 | CHCHD3 | 0.157177502 | 0.0002638 |
| Q91ZW3 | 93762 | SMARCA5 | 0.156077114 | 1.295E-06 |
| O55142 | 57808 | RPL35A | 0.155797175 | 3.104E-05 |
| Q921E4 | 13476 | REEP5 | 0.154244501 | 2.681E-05 |
| P62774 | 14489 | MTPN | 0.154117399 | 7.104E-05 |
| D3Z5M2 |  |  | 0.153363229 | 3.601E-07 |
| P32020 | 20280 | SCP2 | 0.151211663 | 8.611E-06 |
| Q9D6J9 | 66925 | SDHD | 0.151115571 | 3.536E-05 |
| A2AUE1 | 13002 | DNAJC5 | 0.149333647 | 6.191E-05 |
| Q3TJG6 | 56351 | PTGES3 | 0.148611129 | 3.898E-06 |
| Q9QYJ3 | 81489 | DNAJB1 | 0.147505501 | 4.633E-06 |
| Q6ZWN5 | 76846 | RPS9 | 0.147467542 | 2.419E-07 |
| Q6PHN9 | 77407 | RAB35 | 0.14606533 | 1.016E-06 |
| Q3TSX8 | 28185 | tomm70a | 0.146053401 | 2.997E-06 |
| Q3TVV6 | 51810 | HNRNPU | 0.145199581 | 3.399E-08 |
| B2RTP7 | 16681 | KRT2 | 0.138786714 | 1.973E-06 |
| Q93092 | 21351 | TALDO1 | 0.138229858 | 1.674E-06 |
| Q3TZK4 | 76614 | IMMT | 0.138083536 | 1.4E-05 |
| P19001 | 16669 | KRT19 | 0.136952944 | 0.0014447 |
| Q9DCJ5 | 68375 | NDUFA8 | 0.134539586 | 0.0006098 |
| Q3TSX5 | 75416 | NOP14 | 0.130475481 | 2.863E-05 |
| P17665 | 12867 | COX7C | 0.129192369 | 0.0001835 |
| Q66JR8 | 69202 | PTMS | 0.126265675 | 8.154E-06 |
| Q3TWN5 | 51810 | HNRNPU | 0.12400455 | 8.586E-06 |
| E9PUH0 | 97159 | A430005L14Rik | 0.123714287 | 4.343E-06 |
| A0A087WRY3 | 98415 | NUCKS1 | 0.123227501 | 1.587E-05 |
| Q3UYV7 | 27225 | DDX24 | 0.121545518 | 8.841E-05 |
| Q9JL35 | 50887 | HMGN5 | 0.116538323 | 1.903E-07 |
| G8DXR6 |  |  | 0.112509162 | 0.000108 |
| Q6PJ91 | 68312 | Gstm7 | 0.108986487 | 0.0001956 |

**Supplementary Tables S3:** The differentially expressed protein in L929 cells after treatment with 100 μM Ni^2+^ for 48h.

| UniProt Accession | Gene ID | Genesymbol | Ratio | P-Value |
| --- | --- | --- | --- | --- |
| P07724 | 11657 | ALB | 2.058121374 | 0.001986569 |
| Q4FZG9 | 407790 | NDUFA4L2 | 1.583834654 | 0.006808105 |
| E9QPX1 | 12822 | COL18A1 | 1.558144018 | 0.003414462 |
| B7ZWC0 | 17988 | NDRG1 | 1.516118548 | 0.000877746 |
| P11087 | 12842 | COL1A1 | 1.450483405 | 0.000616591 |
| Q8BG60 | 56338 | TXNIP | 1.443624917 | 0.001368573 |
| P02463 | 12826 | COL4A1 | 1.411090572 | 0.000808994 |
| G5E898 | 19041 | PPL | 1.3894989 | 0.006871288 |
| O88207 | 12831 | COL5A1 | 1.367118935 | 0.002010482 |
| P17563 | 20341 | SELENBP1 | 1.333642614 | 0.000338161 |
| Q8R180 | 50527 | ERO1L | 1.22180243 | 0.008682215 |
| P07356 | 12306 | ANXA2 | 0.830460106 | 0.00018654 |
| B1AWZ5 | 66536 | NIPSNAP3B | 0.82885274 | 0.005351566 |
| P16858 | 14433 | GAPDH | 0.827659094 | 6.8961E-05 |
| Q04899 | 18557 | CDK18 | 0.827563173 | 0.001879289 |
| Q3TVZ1 | 12359 | CAT | 0.827373046 | 0.000931853 |
| E0CY49 | 109900 | ASL | 0.82589112 | 0.008182963 |
| Q3UX10 | 238463 | TUBAL3 | 0.82577924 | 0.000957769 |
| Q60972 | 19646 | RBBP4 | 0.825679579 | 0.003213476 |
| D3Z2J6 | 66958 | TMX2 | 0.825270666 | 0.002886713 |
| O88967 | 27377 | YME1L1 | 0.824430015 | 0.003133706 |
| E9QAT4 | 227648 | SEC16A | 0.824167129 | 0.001752585 |
| Q3TW36 | 108077 | SKIV2L | 0.823492945 | 5.53322E-07 |
| Q9Z1N5 | 53817 | DDX39B | 0.821898613 | 0.000200885 |
| Q3TW01 | 67844 | RAB32 | 0.819693524 | 0.002051295 |
| F8WIH0 | 83997 | SLMAP | 0.818599585 | 0.004849166 |
| Q6IWE2 | 11461 | ACTB | 0.817912936 | 0.006226296 |
| Q99N87 | 77721 | MRPS5 | 0.816585593 | 0.003710409 |
| Q91WP9 | 110095 | PYGL | 0.816509219 | 0.000792096 |
| Q4FJY5 | 67103 | PTGR1 | 0.816363895 | 0.004602788 |
| Q8BLL4 | 70478 | mipep | 0.814566524 | 0.00097234 |
| Z4YJU8 | 99412 | GOLGA2 | 0.811710629 | 0.001930414 |
| P97429 | 11746 | ANXA4 | 0.811372306 | 0.003309029 |
| Q1KYM0 |  |  | 0.811337134 | 0.00079855 |
| Q91VC9 | 66092 | GHITM | 0.809218701 | 0.009437261 |
| Q9CUB4 | 68646 | NADK2 | 0.808793945 | 0.006821165 |
| Q7TPR4 | 109711 | ACTN1 | 0.808433728 | 8.78876E-05 |
| Q8R361 | 52055 | rab11fip5 | 0.808419498 | 0.003844968 |
| Q9CQR4 | 66834 | ACOT13 | 0.808106452 | 0.007027081 |
| Q9JHW2 | 52633 | NIT2 | 0.80804983 | 0.000555211 |
| P10649 | 14862 | GSTM1 | 0.807760787 | 0.00011171 |
| O88712 | 13016 | CTBP1 | 0.807133746 | 0.000504244 |
| P52825 | 12896 | cpt2 | 0.806816298 | 0.00613845 |
| P24668 | 17113 | M6PR | 0.805833985 | 0.00712136 |
| Q8K0L1 | 104776 | ALDH6A1 | 0.805678124 | 0.001446453 |
| Q3UAS4 | 19156 | PSAP | 0.802605604 | 0.000808025 |
| P17047 | 16784 | LAMP2 | 0.801699788 | 0.004018314 |
| Q8R0M2 | 216558 | UGP2 | 0.801662087 | 0.00134289 |
| A0A068F126 |  |  | 0.801589581 | 0.008175847 |
| Q9JJE7 | 60527 | FADS3 | 0.801521889 | 0.007855649 |
| Q8C586 | 19663 | RBPMS | 0.80135307 | 0.00446232 |
| Q8VBT6 | 171504 | APOBR | 0.799696624 | 0.002264472 |
| Q8K2F8 | 67070 | LSM14A | 0.799391554 | 0.002966536 |
| O09172 | 14630 | GCLM | 0.798786416 | 0.00058032 |
| P32020 | 20280 | SCP2 | 0.798469662 | 0.003316531 |
| Q91YN9 | 213539 | BAG2 | 0.796921708 | 7.43966E-05 |
| Q5MJ56 | 17921 | MYO7A | 0.79657621 | 5.4923E-05 |
| Q3UDR2 | 18453 | P4HB | 0.796360632 | 1.55276E-05 |
| E9Q634 | 71602 | MYO1E | 0.795809541 | 0.005745456 |
| Q3V3R1 | 270685 | MTHFD1L | 0.795410823 | 0.000171684 |
| Q9D136 | 66179 | OGFOD3 | 0.794406229 | 0.001147712 |
| E9PUE7 | 109934 | ABR | 0.79423456 | 0.001190624 |
| Q9R118 | 56213 | HTRA1 | 0.7936273 | 0.001857808 |
| E9PWK1 | 13849 | EPHX1 | 0.793229882 | 0.003077595 |
| F6SPK0 | 56228 | UBE2J1 | 0.792973849 | 0.000434773 |
| Q80YW9 | 338355 | FKBP15 | 0.791960285 | 0.000779911 |
| Q8BIJ6 | 381314 | iars2 | 0.791679608 | 0.001279045 |
| E9QLA5 | 70435 | INF2 | 0.791262954 | 0.009567169 |
| O70475 | 22235 | UGDH | 0.791191629 | 0.000254327 |
| Q8R1F1 | 227737 | FAM129B | 0.791145372 | 0.001428127 |
| Q3U9Q8 | 227753 | GSN | 0.791045563 | 5.05503E-05 |
| A2AUE1 | 13002 | DNAJC5 | 0.79048935 | 0.000439076 |
| Q5F258 | 216963 | GIT1 | 0.790005652 | 0.0058628 |
| Q9D1I2 | 68480 | CARD19 | 0.789922477 | 0.002072075 |
| Q8C129 | 240028 | LNPEP | 0.789376821 | 0.000383435 |
| Q3UDQ7 | 69597 | AFG3L2 | 0.789344596 | 4.07121E-05 |
| G3X8R5 | 69232 | QRICH1 | 0.789266661 | 0.003869739 |
| O54752 | 27419 | NAGLU | 0.789130456 | 0.000526092 |
| Q3TCH7 | 99375 | CUL4A | 0.788539058 | 0.006746182 |
| P62983 | 78294 | RPS27A | 0.788491056 | 3.74365E-06 |
| Q571H0 | 207932 | URB1 | 0.788153417 | 0.007972212 |
| P62880 | 14693 | GNB2 | 0.786749723 | 0.001911528 |
| P08228 | 20655 | SOD1 | 0.78666742 | 0.000468958 |
| Q3TF87 | 226414 | DARS | 0.786131532 | 0.000203978 |
| Q9R0Y5 | 11636 | AK1 | 0.786069774 | 4.53924E-05 |
| Q8BNF0 | 233833 | TNRC6A | 0.785908559 | 0.009276497 |
| Q8BIW1 | 229589 | PRUNE1 | 0.78562453 | 0.00448039 |
| P97825 | 15374 | HN1 | 0.785545907 | 0.006429192 |
| Q9QXS1 | 18810 | PLEC | 0.785369182 | 5.19254E-06 |
| E9PYT3 | 109168 | ATL3 | 0.78455979 | 0.004015303 |
| Q9CRD0 | 68095 | OCIAD1 | 0.784085432 | 0.007002016 |
| Q99KQ4 | 59027 | NAMPT | 0.784052838 | 0.002329679 |
| P97822 | 66471 | ANP32E | 0.78371773 | 0.0023206 |
| Q95457 | 14964 | H2-D1 | 0.783280876 | 0.004441801 |
| E9PUD2 | 74006 | DNM1L | 0.78283609 | 0.00096655 |
| Q9Z2L6 | 17330 | MINPP1 | 0.782579025 | 0.002237002 |
| Q3TG21 | 66335 | ATP6V1C1 | 0.782167398 | 0.000931883 |
| Q64337 | 18412 | SQSTM1 | 0.782137858 | 0.006211367 |
| Q66JR8 | 69202 | PTMS | 0.781609841 | 0.001189228 |
| Q80UK4 | 231830 | MICALL2 | 0.78110201 | 0.008057677 |
| Q9CQI6 | 72042 | COTL1 | 0.780950023 | 0.009649659 |
| Q62446 | 30795 | FKBP3 | 0.779820887 | 0.003320921 |
| Q80VJ3 | 381101 | DNPH1 | 0.77969235 | 0.003494916 |
| A0A087WRU0 | 21961 | TNS1 | 0.779578296 | 0.001166731 |
| B2RY90 | 664994 | Isoc2a | 0.779526325 | 0.000667595 |
| Q3TJH1 | 14679 | gnai3 | 0.779380174 | 0.000846051 |
| Q11011 | 19155 | NPEPPS | 0.779081754 | 0.001706123 |
| Q9D517 | 28169 | AGPAT3 | 0.779037921 | 1.09665E-05 |
| P48678 | 16905 | LMNA | 0.778816068 | 6.78547E-05 |
| Q3UE92 | 170750 | XPNPEP1 | 0.778799058 | 0.000629261 |
| B1AZ46 | 108100 | BAIAP2 | 0.778487481 | 0.002100449 |
| Q920A5 | 74617 | SCPEP1 | 0.778203766 | 0.005817091 |
| Q5NC05 | 74044 | TTF2 | 0.777945126 | 0.002309838 |
| O54941 | 57376 | SMARCE1 | 0.777386927 | 0.006581174 |
| P61804 | 13135 | DAD1 | 0.777041674 | 0.001873126 |
| I7HJS1 | 13726 | EMD | 0.776186939 | 0.006224637 |
| Q91VR8 | 101314 | BRK1 | 0.775894443 | 0.001418998 |
| Q3TB65 | 75805 | NLN | 0.775858203 | 8.58257E-05 |
| F6SQH7 | 67811 | poldip2 | 0.77584564 | 0.004961749 |
| G3X9U9 | 66437 | FIS1 | 0.775547169 | 0.004572297 |
| Q3UXJ3 | 72692 | HNRNPLL | 0.775002083 | 0.009652258 |
| Q3U7I9 | 13033 | CTSD | 0.774757977 | 0.00029547 |
| G5E8R3 | 18563 | Pcx | 0.774453184 | 0.000300382 |
| Q3UBB0 | 75612 | GNS | 0.774343121 | 0.00049141 |
| G5E884 | 18479 | PAK1 | 0.7741327 | 0.006954447 |
| Q9D7J9 | 67856 | ECHDC3 | 0.774125194 | 0.008045108 |
| P48036 | 11747 | ANXA5 | 0.774071543 | 1.03249E-06 |
| Q60598 | 13043 | CTTN | 0.77375903 | 0.000117138 |
| D3Z0F3 | 21780 | TFAM | 0.773372401 | 0.00202722 |
| Q9D8W6 | 56248 | AK3 | 0.773178607 | 0.002322619 |
| Q6ZQH4 | 72462 | RRP1B | 0.772871808 | 0.006087151 |
| Q3TCZ2 | 63959 | SLC29A1 | 0.772599156 | 0.000762554 |
| Q60710 | 56045 | SAMHD1 | 0.772367918 | 0.002514778 |
| Q3UAG2 | 110208 | PGD | 0.772341753 | 2.4935E-05 |
| Q62418 | 13169 | DBNL | 0.772328117 | 0.000235437 |
| Q922S4 | 207728 | PDE2A | 0.77199851 | 0.002809987 |
| Q9Z247 | 27055 | FKBP9 | 0.77196077 | 5.49138E-05 |
| Q5RKP0 | 26949 | VAT1 | 0.771918459 | 0.000291569 |
| Q99K30 | 98845 | EPS8L2 | 0.770538898 | 0.008092711 |
| E9Q7G0 | 101706 | NUMA1 | 0.770510231 | 0.000699679 |
| Q3UAX2 | 16956 | LPL | 0.770306311 | 0.001098069 |
| Q05C51 | 69724 | RNASEH2A | 0.769668148 | 0.000849842 |
| P47199 | 12972 | CRYZ | 0.769661217 | 0.00105281 |
| Q63844 | 26417 | MAPK3 | 0.769478964 | 0.004562644 |
| D3Z074 | 13367 | DIAPH1 | 0.769299157 | 0.00371065 |
| A2AP32 | 230075 | NDUFB6 | 0.768889015 | 0.000778418 |
| Q6ZPE2 | 77980 | SBF1 | 0.76883859 | 0.000380397 |
| Q3UDS4 | 59010 | SQRDL | 0.768549852 | 0.000212961 |
| Q62426 | 13014 | CSTB | 0.768043684 | 0.002969634 |
| O35344 | 16648 | KPNA3 | 0.767613818 | 0.001437496 |
| Q8BHC4 | 68087 | DCAKD | 0.767228696 | 0.002029887 |
| E9QLB8 | 76889 | COQ8B | 0.767063567 | 0.00025694 |
| B9EKJ3 | 67074 | MON2 | 0.766835754 | 0.000660204 |
| O88447 | 16593 | KLC1 | 0.766738136 | 0.006813732 |
| B1AT10 | 76626 | MSI2 | 0.766531485 | 0.004007455 |
| Q8BWT1 | 52538 | ACAA2 | 0.76628346 | 8.27455E-06 |
| P11438 | 16783 | LAMP1 | 0.766234397 | 0.004571781 |
| Q6NXX6 | 11975 | ATP6V0A1 | 0.766172397 | 0.005942271 |
| Q91Z53 | 76238 | GRHPR | 0.766103931 | 0.000294215 |
| G3X8Y7 | 26568 | SLC27A3 | 0.766086673 | 0.003242863 |
| Q6PB52 | 16976 | LRPAP1 | 0.765256776 | 0.001343449 |
| Q80UK8 | 70422 | INTS2 | 0.765184759 | 0.006916682 |
| A2BFF8 | 13427 | DYNC1I2 | 0.765164659 | 0.001828108 |
| Q91WC0 | 52690 | SETD3 | 0.765148638 | 0.005577097 |
| Q02819 | 18220 | NUCB1 | 0.764947733 | 0.002134585 |
| Q8BP92 | 26611 | RCN2 | 0.764917107 | 0.000947445 |
| Q3UIS6 | 21761 | MORF4L1 | 0.76464599 | 0.00626155 |
| P08752 | 14678 | GNAI2 | 0.764639186 | 0.000678078 |
| Q640N1 | 11568 | AEBP1 | 0.764553407 | 0.003622427 |
| P50544 | 11370 | ACADVL | 0.764315188 | 0.000688075 |
| B7ZWC4 | 16004 | IGF2R | 0.764243543 | 0.000194347 |
| Q9WV03 | 108160 | FAM50A | 0.76402109 | 0.007982631 |
| Q8R3B1 | 18799 | PLCD1 | 0.7639934 | 0.009451978 |
| P70697 | 22275 | UROD | 0.763950653 | 0.001817293 |
| Q99JX6 | 11749 | ANXA6 | 0.76365045 | 0.001723366 |
| E9Q197 | 67201 | GLOD4 | 0.763528659 | 0.000463984 |
| Q3UDD3 | 73826 | POLDIP3 | 0.763390056 | 0.00345399 |
| Q9QYS9 | 19317 | Qk | 0.763068264 | 0.004692726 |
| Q91V41 | 68365 | RAB14 | 0.763048561 | 0.00794177 |
| Q8C845 | 27984 | EFHD2 | 0.762972619 | 0.000818112 |
| B1AZ15 | 319876 | COBLL1 | 0.762849128 | 0.00055983 |
| Q9DCN2 | 109754 | cyb5r3 | 0.762671691 | 0.003821565 |
| Q9D8V0 | 14950 | H13 | 0.762412404 | 0.009649408 |
| A1L3S7 | 229542 | GATAD2B | 0.762200921 | 9.66539E-05 |
| Q3U3C2 | 67963 | NPC2 | 0.761957998 | 0.000105603 |
| Q9CPN9 | 67373 | 2210010C04Rik | 0.761921295 | 0.006762609 |
| Q9D0F3 | 70361 | LMAN1 | 0.761811287 | 0.002833142 |
| O35295 | 19291 | PURB | 0.761375925 | 0.001466692 |
| Q8CFX1 | 100198 | H6PD | 0.761332438 | 0.007914423 |
| P42125 | 13177 | ECI1 | 0.761163412 | 0.003345435 |
| Q3U5I9 | 19079 | PRKAB1 | 0.760959631 | 0.004693248 |
| Q3TQP7 | 110446 | ACAT1 | 0.760762737 | 0.000150312 |
| Q9R257 | 15199 | HEBP1 | 0.760717403 | 0.001493843 |
| F8WIB1 | 104303 | ARL1 | 0.76068696 | 0.009975264 |
| Q8BMD8 | 229731 | SLC25A24 | 0.760211449 | 0.00196557 |
| F8VPK5 | 19878 | ROCK2 | 0.760149132 | 6.76701E-05 |
| P70699 | 14387 | gaa | 0.759944329 | 0.004018309 |
| Q6P5D8 | 74355 | SMCHD1 | 0.7599028 | 0.000119822 |
| Q8R2Y2 | 84004 | MCAM | 0.759628169 | 7.79209E-06 |
| Q9CQJ4 | 19821 | RNF2 | 0.75959694 | 0.003243825 |
| P62075 | 30055 | TIMM13 | 0.759549572 | 0.000426801 |
| U3RKD2 | 218756 | SLC4A7 | 0.75951525 | 5.80273E-05 |
| P50516 | 11964 | ATP6V1A | 0.75931137 | 0.000421833 |
| Q9JKB1 | 50933 | UCHL3 | 0.759239036 | 0.005096527 |
| Q6P1B9 | 30948 | BIN1 | 0.758771233 | 0.000295616 |
| Q9D4H1 | 66482 | exoc2 | 0.758234858 | 0.000801816 |
| P68368 | 22145 | tuba4a | 0.758232085 | 0.000182812 |
| B7ZWL1 | 234594 | CNOT1 | 0.758171801 | 0.000640812 |
| Q62048 | 18611 | Pea15a | 0.757880987 | 0.002684343 |
| Q80U93 | 227720 | NUP214 | 0.757688385 | 0.003623273 |
| P84089 | 13877 | ERH | 0.757579581 | 0.001585606 |
| P62874 | 14688 | GNB1 | 0.757409377 | 0.007146762 |
| Q9R0E1 | 26433 | PLOD3 | 0.757195342 | 0.001020612 |
| F8VPN4 | 77559 | AGL | 0.757052193 | 0.009402521 |
| B9EKT6 | 404710 | IQGAP3 | 0.757043781 | 0.003733879 |
| Q3U9H4 | 16430 | STT3A | 0.756801327 | 6.61252E-05 |
| Q3V1L7 | 80294 | POFUT2 | 0.756684565 | 0.006160968 |
| P50518 | 11973 | ATP6V1E1 | 0.756604551 | 0.001611525 |
| Q91YU8 | 235036 | PPAN | 0.756458386 | 0.001953268 |
| Q3TW96 | 227620 | UAP1L1 | 0.75620611 | 2.15465E-05 |
| Q91V12 | 70025 | ACOT7 | 0.756178459 | 0.000927867 |
| Q3UZI6 | 57741 | NOC2L | 0.756011439 | 0.004647691 |
| Q6A0A9 | 218236 | FAM120A | 0.755980884 | 0.000122742 |
| P50543 | 20195 | S100A11 | 0.755853775 | 0.000101398 |
| Q921H8 | 113868 | Acaa1a | 0.755718256 | 0.000239433 |
| Q8BK57 | 217715 | EIF2B2 | 0.755634998 | 0.00055113 |
| Q91VM9 | 74776 | PPA2 | 0.755559681 | 0.001246807 |
| Q6ZWM4 | 76522 | LSM8 | 0.755347521 | 0.005017872 |
| P40124 | 12331 | CAP1 | 0.755338311 | 0.000595261 |
| B8JJI4 | 224630 | BNIP1 | 0.755220074 | 0.006303119 |
| Q8CCJ3 | 67490 | UFL1 | 0.754977721 | 0.000532681 |
| S4R294 | 226562 | PRRC2C | 0.754875085 | 0.002095087 |
| Q8BFZ9 | 244373 | ERLIN2 | 0.754576055 | 0.000782736 |
| Q8R0W0 | 223650 | EPPK1 | 0.754575086 | 0.000350791 |
| Q8C2Q8 | 11949 | ATP5C1 | 0.754517793 | 0.001681206 |
| Q3U2B5 | 68188 | SYMPK | 0.754110914 | 0.005647092 |
| P62748 | 53602 | HPCAL1 | 0.753682166 | 1.21104E-05 |
| Q3TFA9 | 50875 | TMOD3 | 0.753630172 | 0.000244699 |
| P54823 | 13209 | DDX6 | 0.753590401 | 0.000230209 |
| Q6NXL1 | 69608 | SEC24D | 0.75355336 | 0.002790211 |
| Q9CQU3 | 67830 | RER1 | 0.753411453 | 0.007795668 |
| D3YYC2 | 66556 | DRAP1 | 0.753321615 | 0.007571707 |
| D3Z645 | 56433 | VPS29 | 0.753123308 | 0.003605676 |
| Q5SW88 | 19324 | RAB1A | 0.752756644 | 0.00087698 |
| P43275 | 80838 | Hist1h1a | 0.752477281 | 0.003548626 |
| Q8BH64 | 259300 | EHD2 | 0.752224683 | 0.001536234 |
| Q3U643 | 276846 | PIGS | 0.752154893 | 0.00804649 |
| P56391 | 110323 | Cox6b1 | 0.752152331 | 0.000117607 |
| D3YYP5 | 54722 | DFNA5 | 0.752111797 | 0.006753671 |
| B9EKJ7 | 228140 | TNKS1BP1 | 0.752079948 | 6.9013E-05 |
| Q8K1A6 | 212139 | cc2d1a | 0.751762704 | 0.005924983 |
| P84084 | 11844 | ARF5 | 0.751589613 | 0.002330155 |
| Q60932 | 22333 | VDAC1 | 0.7515104 | 0.00015417 |
| Q99LC2 | 67337 | CSTF1 | 0.75121455 | 0.003572559 |
| Q7TT42 |  |  | 0.751053493 | 0.000469653 |
| Q9D5V5 | 75717 | CUL5 | 0.75091341 | 0.000951669 |
| Q3U9U5 | 27061 | BCAP31 | 0.750744733 | 5.66938E-05 |
| Q5U438 | 18148 | NPM1 | 0.750686233 | 5.43828E-06 |
| Z4YKV1 | 14683 | GNAS | 0.750569846 | 0.000106514 |
| Q8BJL1 | 71865 | FBXO30 | 0.750502279 | 0.002958543 |
| B7ZNJ0 | 11491 | ADAM17 | 0.750307284 | 0.003002346 |
| Q9CPU0 | 109801 | GLO1 | 0.75012765 | 0.000110536 |
| A2A4B3 | 64899 | LPIN3 | 0.750101445 | 0.003478783 |
| Q80UL3 | 14635 | GALK1 | 0.749977742 | 0.000130856 |
| A2AMW0 | 12345 | CAPZB | 0.74996561 | 0.000360154 |
| Q7TQH0 | 233871 | ATXN2L | 0.749926645 | 0.002097492 |
| Q9D1A2 | 66054 | CNDP2 | 0.749682716 | 8.82464E-05 |
| Q8R5J9 | 65106 | ARL6IP5 | 0.749519061 | 0.000299087 |
| O55143 | 11938 | ATP2A2 | 0.74921563 | 2.66244E-05 |
| Q6NXI6 | 75137 | RPRD2 | 0.749188497 | 0.000789958 |
| Q9WUM3 | 23789 | CORO1B | 0.748848613 | 0.000634813 |
| Q9CQC7 | 68194 | NDUFB4 | 0.748822173 | 0.005779634 |
| E9QAT0 | 14265 | FMR1 | 0.748722346 | 0.003421084 |
| Q8CEE7 | 108841 | RDH13 | 0.748462265 | 0.003325081 |
| P26041 | 17698 | MSN | 0.748219053 | 9.5596E-06 |
| Q8R1V4 | 103694 | TMED4 | 0.748147333 | 0.000648724 |
| A6H663 | 29810 | BAG3 | 0.747963903 | 0.001826891 |
| P03958 | 11486 | ADA | 0.747949282 | 0.005549981 |
| Q8K039 | 66202 | 1110059G10Rik | 0.747789812 | 0.006213646 |
| Q8C2A3 | 235072 | SEPT7 | 0.747682034 | 0.000151835 |
| Q99LI2 | 229725 | CLCC1 | 0.747537548 | 0.003111897 |
| Q8CFQ9 | 233908 | FUS | 0.747536142 | 0.004449433 |
| Z4YKC4 | 230861 | EIF4G3 | 0.747300133 | 0.000135433 |
| Q8K1N4 | 72572 | SPATS2 | 0.747297203 | 0.001437148 |
| Q3TEZ2 | 83669 | wdr6 | 0.747280183 | 0.001886718 |
| P08113 | 22027 | HSP90B1 | 0.747079855 | 1.84957E-06 |
| Q3U7R1 | 23943 | ESYT1 | 0.747041538 | 0.000272643 |
| D3YWF6 | 107260 | OTUB1 | 0.746818222 | 0.000553991 |
| Q68FD5 | 67300 | CLTC | 0.746806823 | 2.65753E-06 |
| Q8C550 | 78688 | NOL3 | 0.74680239 | 0.000316127 |
| P62141 | 19046 | ppp1cb | 0.746482441 | 0.004068914 |
| Q7TQ39 |  |  | 0.746264921 | 0.000601205 |
| A2CG44 | 17127 | SMAD3 | 0.746186895 | 0.000945962 |
| E9Q368 | 212728 | TARBP1 | 0.746064568 | 0.006726182 |
| Q9DBS1 | 74122 | TMEM43 | 0.746001998 | 2.8329E-05 |
| Q9CSZ8 | 67036 | MRPL45 | 0.745772984 | 0.006028676 |
| Q9CQ18 | 68209 | rnaseh2c | 0.745747972 | 0.003357943 |
| Q8C0I1 | 228061 | AGPS | 0.745646591 | 0.000231717 |
| E9QAH1 | 224139 | GOLGB1 | 0.745598615 | 0.000468738 |
| Q99JF8 | 101739 | PSIP1 | 0.745427364 | 5.66376E-05 |
| P40336 | 30930 | VPS26A | 0.745385874 | 0.000355031 |
| Q62422 | 20409 | OSTF1 | 0.74534064 | 0.002622933 |
| A2CG35 | 19328 | RAB12 | 0.745231039 | 0.008045658 |
| Q62318 | 21849 | TRIM28 | 0.745200131 | 8.7186E-06 |
| D3Z7P3 | 14660 | GLS | 0.745110882 | 0.000395437 |
| D3Z6S1 | 68796 | TMEM214 | 0.745110823 | 0.000840372 |
| P24527 | 16993 | LTA4H | 0.745087588 | 0.002092019 |
| P83887 | 103733 | TUBG1 | 0.745027802 | 0.000754243 |
| Q8BTB8 | 18120 | MRPL49 | 0.744977898 | 0.001812363 |
| P35762 | 12520 | CD81 | 0.744977595 | 0.001004281 |
| Q8BYA0 | 108903 | TBCD | 0.744939373 | 6.6797E-05 |
| Q8BY87 | 74996 | USP47 | 0.744775553 | 2.64382E-05 |
| O55135 | 16418 | EIF6 | 0.744767585 | 0.000832392 |
| E9Q586 | 13191 | DCTN1 | 0.744738156 | 1.41325E-05 |
| A2AWI9 | 227700 | SH3GLB2 | 0.744658809 | 0.001307724 |
| P62192 | 19179 | PSMC1 | 0.744612005 | 0.002616129 |
| O08614 | 22288 | UTRN | 0.74444899 | 0.000499347 |
| Q8R086 | 211389 | SUOX | 0.744440178 | 0.007217107 |
| D3YWT0 | 56529 | SEC11A | 0.744296931 | 4.62557E-06 |
| F6VQ81 | 66314 | TPD52L2 | 0.744291768 | 0.001034133 |
| E9Q4Q2 | 22668 | SF1 | 0.744266524 | 0.004867453 |
| P97370 | 11933 | ATP1B3 | 0.74422305 | 0.00011608 |
| Q8C1B7 | 52398 | SEPT11 | 0.744129614 | 0.001292407 |
| Q9D0S9 | 68917 | HINT2 | 0.743922059 | 0.005923216 |
| Q9D6J9 | 66925 | SDHD | 0.743768738 | 0.004706814 |
| Q8BU33 | 216136 | ILVBL | 0.743759875 | 0.001930353 |
| Q3UM23 | 107702 | RNH1 | 0.743734688 | 0.000520868 |
| O08529 | 12334 | CAPN2 | 0.743487252 | 7.86015E-05 |
| Q3UJ44 | 12332 | CAPG | 0.743411421 | 4.78908E-05 |
| Q3TJN9 | 19224 | PTGS1 | 0.743289593 | 0.000256225 |
| Q8CIB5 | 218952 | FERMT2 | 0.743265764 | 0.000371671 |
| P70677 | 12367 | CASP3 | 0.743161486 | 0.000890349 |
| Q9EQQ2 | 67180 | YIPF5 | 0.743078582 | 0.000486611 |
| Q3V0L4 | 56878 | RBMS1 | 0.742960124 | 0.004102631 |
| Q8BFQ8 | 213350 | PDDC1 | 0.742746155 | 0.008816283 |
| Q3TXN0 | 64704 | HTRA2 | 0.742658132 | 0.009561204 |
| E9Q1S3 | 20334 | SEC23A | 0.742418653 | 0.001433904 |
| G5E8R1 | 22003 | TPM1 | 0.742162267 | 5.32781E-05 |
| P10639 | 22166 | Txn1 | 0.742105477 | 0.00132271 |
| Q9ERU9 | 19386 | RANBP2 | 0.742090522 | 8.80735E-05 |
| P52624 | 22271 | UPP1 | 0.742020407 | 0.001734515 |
| P31938 | 26395 | MAP2K1 | 0.741998586 | 0.000290623 |
| Q8C1A5 | 50492 | THOP1 | 0.741979757 | 9.99819E-05 |
| O55234 | 19173 | PSMB5 | 0.741538685 | 0.002130388 |
| D3YTP0 | 68428 | STEAP3 | 0.74139751 | 0.000269491 |
| G3UWZ0 | 217578 | BAZ1A | 0.741392173 | 0.000235181 |
| Q9CYG7 | 67145 | TOMM34 | 0.741224151 | 0.000102925 |
| Q3TC93 | 58240 | HS1BP3 | 0.740951958 | 0.005445716 |
| Q99L47 | 70356 | ST13 | 0.740676052 | 2.19456E-05 |
| Q9EQK5 | 78388 | MVP | 0.74066764 | 6.18251E-05 |
| Q3TZZ7 | 52635 | ESYT2 | 0.740462155 | 0.000180776 |
| Q99JW7 | 12534 | CDK1 | 0.740414562 | 0.000899041 |
| Q3U417 | 67533 | PPFIBP1 | 0.740279966 | 0.003231637 |
| Q3TSZ4 | 319625 | GALM | 0.74024146 | 0.005012987 |
| Q91W09 | 14151 | FECH | 0.740057358 | 0.007075893 |
| E9PXX7 | 105245 | TXNDC5 | 0.740048144 | 7.44648E-06 |
| P97315 | 13007 | CSRP1 | 0.739938178 | 0.00673699 |
| P21981 | 21817 | TGM2 | 0.739825178 | 0.000954973 |
| N0E4C0 | 13001 | CSNK2B | 0.739720763 | 0.000294972 |
| G8JL40 | 99982 | KDM1A | 0.739633738 | 0.002864761 |
| Q921C5 | 76895 | BICD2 | 0.739283013 | 0.008490652 |
| Q7TMB8 | 20430 | CYFIP1 | 0.739269278 | 0.000270286 |
| E9Q616 | 66395 | AHNAK | 0.7392508 | 5.3555E-06 |
| Q64727 | 22330 | VCL | 0.739152043 | 0.000419078 |
| P99029 | 54683 | PRDX5 | 0.739112697 | 0.001877477 |
| B2RY51 | 109181 | TRIP11 | 0.739075294 | 0.000329751 |
| Q9CQH7 | 70533 | BTF3L4 | 0.738848512 | 0.002959002 |
| Q9D6Y7 | 110265 | MSRA | 0.738813993 | 0.000388674 |
| Q3TUW9 | 13660 | EHD1 | 0.738691744 | 0.000458011 |
| Q71FD7 | 74202 | FBLIM1 | 0.738657508 | 0.009395849 |
| Q03958 | 14976 | PFDN6 | 0.738613502 | 0.001275484 |
| Q8BIZ9 | 68058 | CHD1L | 0.738584544 | 0.001108778 |
| P56376 | 66204 | ACYP1 | 0.738570856 | 0.004153062 |
| O35114 | 12492 | SCARB2 | 0.738565109 | 0.000210477 |
| Q3UTE4 | 19357 | RAD21 | 0.738467674 | 0.000391604 |
| P48428 | 21371 | TBCA | 0.738412387 | 0.000173704 |
| Q3TYR7 | 54673 | SH3GLB1 | 0.738347728 | 0.009749874 |
| Q6ZQI3 | 109154 | MLEC | 0.738335919 | 0.000850515 |
| P07901 | 15519 | HSP90AA1 | 0.738296704 | 0.00022238 |
| Q3UG53 | 20868 | STK10 | 0.738224819 | 0.006449227 |
| Q3U449 | 23827 | BPNT1 | 0.73805901 | 0.002143308 |
| G5E8J3 | 207425 | WDR11 | 0.737882658 | 0.000425908 |
| F8WHU7 | 20842 | STAG1 | 0.73787006 | 0.003968886 |
| O55091 | 16210 | IMPACT | 0.737822483 | 0.001541663 |
| Q99K51 | 102866 | PLS3 | 0.737714857 | 1.14179E-05 |
| Q8R146 | 235606 | APEH | 0.737623395 | 0.009200563 |
| Q8K2B0 | 66180 | P3H4 | 0.737311954 | 0.002360829 |
| G3X963 | 70472 | ATAD2 | 0.737197565 | 0.003479695 |
| Q9D828 | 21787 | TFG | 0.737166968 | 0.005414378 |
| Q9Z2M7 | 54128 | PMM2 | 0.736974839 | 0.006478311 |
| P56389 | 72269 | CDA | 0.736920167 | 9.87515E-06 |
| P26883 | 14225 | FKBP1A | 0.73654553 | 0.00198477 |
| P10833 | 20130 | RRAS | 0.736539496 | 0.001149916 |
| Q8K239 | 66399 | TSFM | 0.736409932 | 0.000690322 |
| P37913 | 16881 | LIG1 | 0.736290885 | 0.002407831 |
| Q3TKT4 | 20586 | SMARCA4 | 0.736276914 | 0.001745858 |
| Q3TDQ1 | 68292 | STT3B | 0.736213245 | 0.000689127 |
| P19783 | 12857 | Cox4I1 | 0.736184123 | 0.002738255 |
| Q3TXJ4 | 20224 | SAR1A | 0.736163087 | 0.001013822 |
| Q8VDD5 | 17886 | MYH9 | 0.736157614 | 1.47814E-06 |
| Q9DBG3 | 71770 | AP2B1 | 0.736089502 | 4.84753E-05 |
| Q9DB05 | 108124 | NAPA | 0.735970292 | 0.001013019 |
| Q8C4J7 | 213773 | TBL3 | 0.735958936 | 0.002892892 |
| Q9D0J2 | 66043 | ATP5D | 0.735873538 | 0.000452432 |
| D3Z7C0 | 18100 | MRPL40 | 0.735869428 | 0.00024604 |
| Q3V493 | 60409 | TRAPPC4 | 0.735847903 | 0.004328421 |
| O35655 | 237178 | PPEF1 | 0.735803541 | 0.008957549 |
| E9PUB7 | 229524 | MSTO1 | 0.735735912 | 0.00270889 |
| Q9CQF0 | 66419 | MRPL11 | 0.735610793 | 0.002830693 |
| Q922R9 | 68015 | TRAP1 | 0.735594772 | 6.5817E-05 |
| Q3TLJ5 | 80280 | CDK5RAP3 | 0.735501555 | 0.001007337 |
| Q3U0V1 | 16549 | KHSRP | 0.735423566 | 0.000407699 |
| Q6ZPJ3 | 217342 | UBE2O | 0.735301967 | 0.00045013 |
| Q8CA04 | 78523 | MRPL9 | 0.735288213 | 0.000551024 |
| Q8K1R7 | 217718 | NEK9 | 0.735151711 | 7.30935E-06 |
| B7ZWI2 | 66118 | SARNP | 0.735061483 | 0.002531524 |
| Q922H4 | 69080 | GMPPA | 0.734768919 | 0.000597687 |
| Q8BP48 | 75624 | METAP1 | 0.734756052 | 0.001509889 |
| E9Q390 | 226101 | MYOF | 0.734685015 | 1.94686E-05 |
| P51863 | 11972 | ATP6V0D1 | 0.734639957 | 0.001158648 |
| Q8CAQ8 | 76614 | IMMT | 0.734630525 | 0.000723531 |
| A0A087WNV1 | 15463 | AGFG1 | 0.734623969 | 0.003216223 |
| B7ZCU2 | 11308 | ABI1 | 0.734566035 | 0.003550071 |
| P70296 | 23980 | PEBP1 | 0.734557799 | 0.000337217 |
| D6REF7 | 20397 | SGPL1 | 0.734467728 | 0.001356773 |
| Q3THS6 | 232087 | MAT2A | 0.73445111 | 0.002938658 |
| Q99JR1 | 14057 | SFXN1 | 0.734282885 | 0.001137219 |
| Q9Z0H8 | 269713 | CLIP2 | 0.734145497 | 0.005968191 |
| Q7TNV0 | 110052 | DEK | 0.734054925 | 0.000189158 |
| P10630 | 13682 | EIF4A2 | 0.734045159 | 0.008639507 |
| Q9JI10 | 56274 | stk3 | 0.733981914 | 4.38108E-05 |
| D3YW48 | 12336 | CAPNS1 | 0.733937921 | 0.00090528 |
| F7BTZ2 | 18854 | PML | 0.733790916 | 0.002258471 |
| Q542W3 | 23881 | G3BP2 | 0.733735769 | 0.000856054 |
| Q80ZS3 | 99045 | MRPS26 | 0.733651375 | 0.004768039 |
| P27601 | 14674 | GNA13 | 0.733646303 | 0.00332527 |
| Q68FL6 | 216443 | MARS | 0.733629407 | 0.00047021 |
| P70441 | 26941 | SLC9A3R1 | 0.7336137 | 0.000121985 |
| Q8VHY0 | 121021 | CSPG4 | 0.733469984 | 4.39859E-05 |
| Q9JJ78 | 52033 | PBK | 0.733462639 | 0.000137633 |
| D3YW87 |  |  | 0.733271625 | 4.52619E-06 |
| Q6IRU2 | 326618 | TPM4 | 0.733171894 | 3.53069E-06 |
| Q3UW66 | 246221 | MPST | 0.733163629 | 0.00057243 |
| Q9CQC6 | 66882 | BZW1 | 0.733098427 | 0.000454838 |
| Q8BG51 | 59040 | RHOT1 | 0.733010253 | 0.000820603 |
| P97393 | 11855 | ARHGAP5 | 0.732899922 | 0.005877435 |
| Q91WU5 | 57344 | AS3MT | 0.732894544 | 0.006670905 |
| Q3UYV7 | 27225 | DDX24 | 0.732871803 | 0.009414399 |
| Q5RKP4 | 103963 | RPN1 | 0.732762421 | 6.52953E-08 |
| Q8BGC4 | 225791 | ZADH2 | 0.732573484 | 0.004132663 |
| Q3TYS2 | 217370 | BC017643 | 0.732536586 | 0.004188428 |
| P23506 | 18537 | PCMT1 | 0.732486161 | 0.009119521 |
| Q3TFD9 | 22352 | VIM | 0.732326153 | 0.007574143 |
| P99024 | 22154 | TUBB5 | 0.732202895 | 7.39091E-05 |
| Q3UGS4 | 192173 | fam195b | 0.732151762 | 0.00075095 |
| Q3UKP5 | 67439 | XAB2 | 0.732135179 | 3.1607E-05 |
| E9PXV3 | 108058 | CAMK2D | 0.732129046 | 0.000811709 |
| Q80W68 | 170643 | KIRREL | 0.732101286 | 0.003352358 |
| P16460 | 11898 | ASS1 | 0.731735869 | 9.29463E-05 |
| Q14AX9 | 17534 | mrc2 | 0.73172155 | 0.002464453 |
| Q69ZY3 | 74737 | PCF11 | 0.731708637 | 0.002957317 |
| Q8BXZ1 | 67988 | TMX3 | 0.731657228 | 0.003790224 |
| Q9Z277 | 22385 | BAZ1B | 0.731629766 | 0.006831365 |
| P15532 | 18102 | NME1 | 0.731367184 | 0.002654835 |
| Q3TB79 | 12333 | CAPN1 | 0.731364084 | 0.000356755 |
| Q9Z2I8 | 20917 | SUCLG2 | 0.731155992 | 0.001418532 |
| Q62186 | 20832 | SSR4 | 0.731111158 | 0.002149133 |
| Q9Z1Q5 | 114584 | CLIC1 | 0.731080742 | 0.000162085 |
| Q923D2 | 233016 | BLVRB | 0.731063649 | 0.000210355 |
| Q99KR7 | 105675 | PPIF | 0.731058376 | 0.001184199 |
| Q9JLI8 | 53890 | SART3 | 0.731045885 | 0.002424736 |
| P28474 | 11532 | ADH5 | 0.731037822 | 0.001024303 |
| A2ALV7 | 230249 | AI314180 | 0.731022904 | 0.009682713 |
| Q9WVA4 | 21346 | TAGLN2 | 0.731003198 | 0.000520794 |
| Q4TVN0 | 66687 | TBC1D15 | 0.730935633 | 0.00067702 |
| Q8VCB1 | 72787 | NDC1 | 0.730918394 | 0.002292312 |
| P70302 | 20866 | STIM1 | 0.730908287 | 0.000648216 |
| Q60605 | 17904 | MYL6 | 0.73079782 | 0.002077854 |
| O88811 | 56324 | STAM2 | 0.730739314 | 0.005093753 |
| D3Z2F7 | 56378 | ARPC3 | 0.73073502 | 0.000876685 |
| O08599 | 20910 | STXBP1 | 0.730694712 | 0.005448612 |
| Q8R5K4 | 230082 | NOL6 | 0.730676247 | 0.005903842 |
| P20152 | 22352 | VIM | 0.730594346 | 0.000443555 |
| Q9D1H8 | 68499 | MRPL53 | 0.730569632 | 0.007386304 |
| Q00612 | 14381 | G6pdx | 0.730548061 | 0.000155447 |
| E9QKL6 | 15951 | Ifi204 | 0.730364868 | 0.004197207 |
| D3Z061 | 231380 | UBA6 | 0.730327221 | 0.001466467 |
| P35831 | 19248 | PTPN12 | 0.730232571 | 0.001130292 |
| Q3UGN9 | 20844 | STAM | 0.73019353 | 0.000381551 |
| Q3U781 | 20383 | SRSF3 | 0.73009351 | 0.001118495 |
| Q9QZL0 | 56532 | ripk3 | 0.730069809 | 0.001387846 |
| P59016 | 233405 | VPS33B | 0.729999273 | 0.008550459 |
| B1AY13 | 329908 | USP24 | 0.729897703 | 0.007606532 |
| O70435 | 19167 | PSMA3 | 0.72987842 | 0.000529971 |
| Q07417 | 11409 | ACADS | 0.729752188 | 0.001321434 |
| Q3TM89 | 76302 | PCNP | 0.729695909 | 0.003171803 |
| Q14DR9 | 67475 | ERO1LB | 0.729562672 | 0.009343037 |
| Q9R1P1 | 26446 | PSMB3 | 0.729500065 | 0.000732011 |
| P29595 | 18002 | NEDD8 | 0.729495191 | 0.00109792 |
| Q8VE88 | 67726 | FAM114A2 | 0.729049417 | 0.001403504 |
| Q8VDN2 | 11928 | ATP1A1 | 0.72891641 | 0.000303677 |
| Q8C9B9 | 23856 | DIDO1 | 0.728908315 | 0.006936069 |
| P35564 | 12330 | CANX | 0.728880644 | 2.88873E-06 |
| H3BKN0 | 28114 | NSUN2 | 0.728768932 | 0.00071514 |
| Q99PL5 | 81910 | RRBP1 | 0.728750762 | 5.3217E-05 |
| P62204 | 12313 | CALM1 | 0.728682529 | 0.000136615 |
| P63328 | 19055 | PPP3CA | 0.72862297 | 0.002942069 |
| Q91ZX7 | 16971 | LRP1 | 0.728617993 | 0.000494664 |
| P35585 | 11767 | AP1M1 | 0.728434813 | 0.008813423 |
| Q78IK4 | 68117 | APOOL | 0.728413612 | 0.000299947 |
| P57776 | 66656 | EEF1D | 0.728413122 | 0.002680109 |
| Q3TW51 | 56463 | SND1 | 0.728391223 | 0.000101213 |
| P63017 | 15481 | HSPA8 | 0.728362195 | 3.39911E-06 |
| Q8BTS4 | 269113 | NUP54 | 0.728354151 | 0.00124259 |
| Q3UKA1 | 100037258 | DNAJC3 | 0.7282405 | 0.00026401 |
| B9EHZ5 | 56524 | MPP6 | 0.728182316 | 0.000159799 |
| G3X9V2 | 12388 | CTNND1 | 0.728082807 | 0.000337724 |
| Q3UIJ2 | 26905 | Eif2s3x | 0.728033272 | 0.00017429 |
| Q8R105 | 107305 | VPS37C | 0.728006714 | 0.003494222 |
| Q922F4 | 67951 | TUBB6 | 0.727974819 | 0.000374432 |
| Q3TFD0 | 108037 | SHMT2 | 0.727948299 | 3.37088E-05 |
| Q8CB77 | 27224 | TCEB3 | 0.727881927 | 0.000889526 |
| Q921X9 | 72599 | PDIA5 | 0.727779955 | 0.001231349 |
| Q3UW53 | 63913 | FAM129A | 0.727735144 | 0.001364154 |
| A4FUV9 | 338364 | TRIM65 | 0.727560363 | 5.97062E-05 |
| P19536 | 12859 | Cox5b | 0.727491929 | 0.005348125 |
| Q921T2 | 208263 | TOR1AIP1 | 0.727408027 | 0.002638368 |
| P28660 | 50884 | NCKAP1 | 0.72740647 | 0.002119372 |
| O35593 | 59029 | PSMD14 | 0.727359199 | 0.001755139 |
| Q8BH80 | 56491 | VAPB | 0.727317044 | 0.004471996 |
| Q99KV1 | 67838 | DNAJB11 | 0.727252316 | 7.82293E-06 |
| Q921I9 | 109075 | EXOSC4 | 0.727114058 | 0.003419067 |
| Q99JI4 | 66413 | PSMD6 | 0.727069414 | 0.000365654 |
| P12815 | 18570 | PDCD6 | 0.727052472 | 0.000939847 |
| O08997 | 11927 | ATOX1 | 0.727022566 | 0.003740472 |
| G3X934 | 545085 | WDR70 | 0.726997325 | 0.003605373 |
| Q8BMS1 | 97212 | HADHA | 0.726946389 | 1.38514E-05 |
| P97855 | 27041 | G3BP1 | 0.726913293 | 0.000146105 |
| P21279 | 14682 | GNAQ | 0.726886309 | 0.000988917 |
| Q61941 | 18115 | NNT | 0.726840123 | 0.000195066 |
| O88792 | 16456 | F11R | 0.726795803 | 0.002302008 |
| O09061 | 19170 | PSMB1 | 0.726751488 | 0.002351072 |
| Q6SLK2 | 232341 | WNK1 | 0.72641556 | 0.0014733 |
| E9Q6Q8 | 210789 | TBC1D4 | 0.726317031 | 1.7765E-05 |
| A2AKI5 | 16410 | ITGAV | 0.726294712 | 0.000222256 |
| K3W4Q8 | 12215 | BSG | 0.72618586 | 4.12318E-05 |
| Q505F5 | 72946 | LRRC47 | 0.726182497 | 0.000319289 |
| Q9JJ28 | 14248 | FLII | 0.726168731 | 0.000406543 |
| Q61213 |  |  | 0.72614803 | 0.000370905 |
| P70349 | 15254 | HINT1 | 0.726058308 | 0.00093527 |
| B2RXS4 | 140570 | PLXNB2 | 0.725988622 | 8.60183E-05 |
| Q9QUM9 | 26443 | PSMA6 | 0.725949206 | 0.000427096 |
| Q9CYN2 | 66624 | SPCS2 | 0.725880419 | 0.003927522 |
| Q8K009 | 216188 | ALDH1L2 | 0.725879353 | 0.000687269 |
| Q8VDM6 | 232989 | HNRNPUL1 | 0.725808891 | 0.000110735 |
| Q6P5F9 | 103573 | XPO1 | 0.725773675 | 2.49569E-06 |
| Q9CPY7 | 66988 | LAP3 | 0.725727127 | 1.72863E-05 |
| Q3TZP3 | 23942 | MTA2 | 0.725713798 | 0.001560886 |
| Q64511 | 21974 | TOP2B | 0.725562426 | 6.9271E-06 |
| F7ACR9 | 11426 | MACF1 | 0.72546061 | 0.00026278 |
| Q3V2N5 | 20650 | SNTB2 | 0.725452012 | 0.004605429 |
| F6ZFT1 | 70316 | NDUFAB1 | 0.72539342 | 5.08256E-05 |
| Q9CS42 | 110639 | PRPS2 | 0.725303872 | 0.002204112 |
| Q3UDE2 | 223723 | TTLL12 | 0.725179448 | 1.12467E-05 |
| E9PWG6 | 54392 | NCAPG | 0.725077692 | 0.000869548 |
| Q8BL66 | 216238 | EEA1 | 0.725022623 | 5.78383E-05 |
| Q64521 | 14571 | GPD2 | 0.725016186 | 0.000186495 |
| Q3TU36 | 229877 | RAP1GDS1 | 0.724977088 | 0.00410118 |
| Q91XI1 | 224907 | DUS3L | 0.724949581 | 0.002833104 |
| Q8VBZ3 | 56457 | CLPTM1 | 0.724880563 | 0.008673248 |
| Q3TI63 | 56440 | SNX1 | 0.724759582 | 0.000237105 |
| Q8CHK3 | 77582 | MBOAT7 | 0.724747492 | 0.004985271 |
| Q9CU62 | 24061 | SMC1A | 0.724618415 | 0.000328998 |
| Q9EQQ9 | 76055 | MGEA5 | 0.724604765 | 0.000143199 |
| P45878 | 14227 | FKBP2 | 0.724588271 | 0.000629874 |
| Q3TBQ3 | 73744 | MAN2C1 | 0.724519445 | 0.00382881 |
| D3Z7E5 | 606496 | GSK3A | 0.724405461 | 0.005116249 |
| Q9D2M8 | 70620 | UBE2V2 | 0.72438573 | 0.000283979 |
| Q9CQ71 | 68240 | RPA3 | 0.724278854 | 0.000366745 |
| Q8K3W0 | 107976 | BRE | 0.724242884 | 0.003475796 |
| P42669 | 19290 | PURA | 0.72420855 | 0.000561161 |
| Q8BIP0 | 226539 | DARS2 | 0.724165094 | 0.000891792 |
| Q6ZPX7 |  |  | 0.724095386 | 0.000214337 |
| Q9DCL9 | 67054 | PAICS | 0.724069605 | 4.37376E-05 |
| Q9CXR1 | 66375 | DHRS7 | 0.724027395 | 0.00023039 |
| Q3URM4 | 76299 | ERP44 | 0.72402321 | 1.50385E-05 |
| Q501J6 | 67040 | DDX17 | 0.724012387 | 6.10305E-05 |
| O35864 | 26754 | COPS5 | 0.723978233 | 0.000158 |
| Q5SVG5 | 11764 | AP1B1 | 0.723955974 | 0.001268625 |
| Q6P5E4 | 320011 | UGGT1 | 0.72389136 | 2.66709E-05 |
| B8ZXI1 | 106248 | QTRTD1 | 0.723869941 | 0.000501658 |
| Q8R5C0 | 17827 | MTX1 | 0.723817629 | 0.00025208 |
| O35887 | 12321 | CALU | 0.723800029 | 0.000870451 |
| G5E866 | 81898 | SF3B1 | 0.723779351 | 0.000378548 |
| P46460 | 18195 | NSF | 0.723648225 | 0.001023037 |
| Q9DC29 | 74104 | ABCB6 | 0.723589241 | 0.005004673 |
| Q925I1 | 108888 | ATAD3A | 0.72356522 | 0.000946353 |
| Q99PT1 | 192662 | ARHGDIA | 0.723544415 | 4.12635E-06 |
| P55096 | 19299 | ABCD3 | 0.723429366 | 0.001278495 |
| Q99KC3 | 110109 | NOP2 | 0.723422213 | 0.003862507 |
| Q3U9G9 | 98386 | LBR | 0.723374209 | 6.45382E-05 |
| O55057 | 18582 | PDE6D | 0.723371756 | 0.000297389 |
| Q9CPN8 | 140488 | IGF2BP3 | 0.723334719 | 0.00609028 |
| P62331 | 11845 | ARF6 | 0.723305158 | 0.00039877 |
| Q9ER88 | 65111 | DAP3 | 0.723296221 | 0.000822666 |
| Q3UQM5 | 105014 | rdh14 | 0.723248569 | 0.001270718 |
| Q8BP71 | 93686 | RBFOX2 | 0.723226574 | 0.005769734 |
| Q9DCW4 | 110826 | ETFB | 0.723225699 | 0.00140901 |
| P16045 | 16852 | LGALS1 | 0.723222945 | 3.04798E-05 |
| B2RSW8 | 18536 | PCM1 | 0.72318029 | 0.000558461 |
| Q8CD09 | 65970 | LIMA1 | 0.723131206 | 0.000107821 |
| Q6NWW1 | 63958 | UBE4B | 0.723066121 | 0.006645943 |
| Q8C1V4 | 85305 | KARS | 0.723062399 | 0.000503009 |
| Q5F2E7 | 68564 | NUFIP2 | 0.722899831 | 0.000611878 |
| Q9CVB6 | 76709 | ARPC2 | 0.722885676 | 0.000355364 |
| Q9WU78 | 18571 | PDCD6IP | 0.72285368 | 0.000858854 |
| Q5SF07 | 319765 | IGF2BP2 | 0.722837052 | 0.00010454 |
| O70591 | 18637 | PFDN2 | 0.722787768 | 0.000948022 |
| Q684I8 | 15929 | IDH3G | 0.722739473 | 0.005378903 |
| Q8C0D5 | 101592 | EFL1 | 0.722609714 | 0.000824049 |
| Q06185 | 11958 | Atp5k | 0.722559964 | 0.000152368 |
| Q9JHI5 | 56357 | IVD | 0.722533359 | 0.00080891 |
| Q922R8 | 71853 | PDIA6 | 0.722525073 | 1.89109E-06 |
| Q5RL55 | 224742 | ABCF1 | 0.722520305 | 1.23177E-05 |
| Q9DC61 | 66865 | PMPCA | 0.72250606 | 0.000573557 |
| P35486 | 18597 | PDHA1 | 0.722505016 | 0.00013489 |
| P54227 | 16765 | STMN1 | 0.722230267 | 0.000412159 |
| Q99L13 | 58875 | HIBADH | 0.722153642 | 0.000281699 |
| P35282 | 216344 | RAB21 | 0.722120239 | 8.29281E-05 |
| A0A087WS18 | 76707 | CLASP1 | 0.722029741 | 0.000579444 |
| D3Z637 | 21985 | TPD52 | 0.721982612 | 7.08177E-05 |
| Q3TFQ8 | 110078 | PYGB | 0.721976295 | 5.95203E-05 |
| Q8C166 | 266692 | CPNE1 | 0.721949837 | 3.57317E-05 |
| A0A0A6YX73 | 19087 | PRKAR2A | 0.721891056 | 0.000708976 |
| Q61578 | 14149 | FDXR | 0.721883999 | 0.003915534 |
| Q80X85 | 50529 | MRPS7 | 0.721692804 | 0.003677155 |
| A0A0A6YXX3 | 80877 | LRBA | 0.721649956 | 0.00354884 |
| P05202 | 14719 | GOT2 | 0.721638738 | 0.000120276 |
| Q9CW46 | 71766 | RAVER1 | 0.72163565 | 0.006275774 |
| Q91VE6 | 67949 | NIFK | 0.721609381 | 0.001281987 |
| Q6PF96 | 66841 | ETFDH | 0.721578362 | 0.000219276 |
| Q9JKY0 | 58184 | CNOT9 | 0.721561392 | 0.002326593 |
| P40142 | 21881 | TKT | 0.721456341 | 8.55635E-07 |
| F6YQT7 | 320355 | LIPI | 0.721319566 | 0.001866893 |
| Q78ZA7 | 17955 | NAP1L4 | 0.721243047 | 0.000598867 |
| A2ALA4 | 20933 | MED22 | 0.721224589 | 0.00895845 |
| P61967 | 11769 | AP1S1 | 0.72111724 | 0.001059184 |
| Q60597 | 18293 | OGDH | 0.721025628 | 0.00051283 |
| O88544 | 26891 | COPS4 | 0.720983288 | 1.08957E-05 |
| Q60749 | 20218 | KHDRBS1 | 0.720963955 | 5.20575E-05 |
| Q99LX0 | 57320 | PARK7 | 0.720902749 | 2.30633E-05 |
| O54734 | 13200 | DDOST | 0.720842894 | 0.000178788 |
| Q1WWK3 | 56702 | HIST1H1B | 0.720823704 | 1.07256E-05 |
| Q3UZ39 | 16978 | LRRFIP1 | 0.720821914 | 0.000200669 |
| Q3TWV4 | 11773 | AP2M1 | 0.720802236 | 0.000576259 |
| Q91WN1 | 108671 | DNAJC9 | 0.720364923 | 0.002107461 |
| P62814 | 11966 | ATP6V1B2 | 0.720327405 | 0.002144691 |
| Q6PB44 | 104831 | PTPN23 | 0.720263834 | 0.002192165 |
| B2RWW6 | 231659 | Gcn1l1 | 0.720173517 | 0.0001252 |
| Q3V1L4 | 76952 | NT5C2 | 0.720169864 | 0.002878642 |
| Q71RI9 | 229905 | KYAT3 | 0.720167853 | 0.001579707 |
| G3UXL2 | 328099 | Prps1l3 | 0.720161979 | 0.004435999 |
| Q3TEA8 | 15441 | HP1BP3 | 0.720139526 | 0.000351211 |
| P14733 | 16906 | LMNB1 | 0.719970534 | 0.000169016 |
| Q9CQJ2 | 68845 | PIH1D1 | 0.71992315 | 0.000625594 |
| Q3UXP2 | 20174 | RUVBL2 | 0.719898638 | 2.33034E-06 |
| Q2YDW1 |  |  | 0.719893182 | 1.33915E-07 |
| O54962 | 23825 | BANF1 | 0.719830271 | 0.0005337 |
| P47753 | 12340 | CAPZA1 | 0.719819952 | 6.04815E-06 |
| A2ADY9 | 68817 | DDI2 | 0.719785063 | 0.00870247 |
| P11031 | 20024 | SUB1 | 0.719783973 | 0.002260644 |
| Q3TLI6 | 75597 | ndufaf2 | 0.719777337 | 0.004642341 |
| O08709 | 11758 | PRDX6 | 0.719710084 | 9.00591E-06 |
| P62869 | 67673 | TCEB2 | 0.719699555 | 0.002624636 |
| P17427 | 11772 | AP2A2 | 0.719575376 | 0.000512193 |
| Q9DBG7 | 67398 | SRPR | 0.719555081 | 0.009585754 |
| P60122 | 56505 | RUVBL1 | 0.719519121 | 8.54319E-05 |
| Q64435 | 94284 | Ugt1a6a | 0.719450176 | 0.000985998 |
| Q62145 | 14470 | RABAC1 | 0.719405182 | 0.004198455 |
| Q497Z1 | 217333 | TRIM47 | 0.719382333 | 0.003465766 |
| Q91W96 | 52206 | ANAPC4 | 0.719297405 | 0.000351961 |
| Q7TMI0 | 69077 | PSMD11 | 0.719238024 | 6.38661E-08 |
| Q50HX0 | 68365 | RAB14 | 0.719142995 | 0.009190284 |
| A0A087WNP6 | 321022 | CDV3 | 0.719095406 | 0.000210249 |
| A7VJ98 | 63985 | GMFB | 0.719063144 | 0.001236526 |
| P42208 | 18000 | SEPT2 | 0.719044967 | 7.15539E-05 |
| Q9D0T1 | 20826 | NHP2L1 | 0.718989608 | 0.006834625 |
| Q8K4Z3 | 246703 | apoa1bp | 0.718882343 | 0.000160068 |
| Q8K297 | 234407 | COLGALT1 | 0.718875653 | 0.003962445 |
| G5E8G0 | 11991 | HNRNPD | 0.718843125 | 0.000383082 |
| E9Q6J5 | 665775 | Bod1l | 0.718643096 | 0.004651396 |
| Q80TU6 | 56215 | ACIN1 | 0.718603522 | 0.003737317 |
| O08807 | 53381 | PRDX4 | 0.718578094 | 0.009177364 |
| P27773 | 14827 | PDIA3 | 0.718540591 | 2.66957E-07 |
| P57780 | 60595 | ACTN4 | 0.718479077 | 0.000109797 |
| P47738 | 11669 | ALDH2 | 0.718440292 | 1.09499E-05 |
| E9Q0W8 | 20643 | SNRPE | 0.718414797 | 0.004161741 |
| Q9R059 | 14201 | FHL3 | 0.71835393 | 0.004537417 |
| P26040 | 22350 | EZR | 0.718252099 | 0.000198242 |
| P21550 | 13808 | ENO3 | 0.718228142 | 0.001743654 |
| Q6ZQH8 | 227699 | NUP188 | 0.718100038 | 0.006438582 |
| Q80X50 | 74383 | UBAP2L | 0.718076437 | 0.0015526 |
| Q80UE5 | 13822 | EPB41L2 | 0.718073438 | 2.59657E-05 |
| Q3TV94 | 107513 | SSR1 | 0.718057532 | 0.002237773 |
| Q61191 | 15161 | HCFC1 | 0.718049321 | 0.001430749 |
| Q3TEL0 | 19063 | PPT1 | 0.71785208 | 0.005894268 |
| Q9DCC4 | 66194 | PYCRL | 0.717787384 | 0.001211873 |
| M0QWY0 | 18117 | EMC8 | 0.717712267 | 0.00183067 |
| P14824 | 11749 | ANXA6 | 0.717577035 | 0.006126194 |
| P56480 | 11947 | ATP5B | 0.717365996 | 1.87209E-06 |
| P02469 | 16777 | LAMB1 | 0.717327153 | 6.74029E-05 |
| P62862 | 14109 | FAU | 0.717275516 | 0.00141787 |
| Q6PGH6 | 71169 | NBAS | 0.717210963 | 0.007969021 |
| P54276 | 17688 | MSH6 | 0.717115258 | 0.000395512 |
| Q8R323 | 69263 | RFC3 | 0.716901955 | 0.000362452 |
| Q2L4X1 | 66912 | BZW2 | 0.716873479 | 0.000249836 |
| Q64310 | 20932 | SURF4 | 0.716800586 | 0.00012825 |
| Q9QZE5 | 54161 | COPG1 | 0.716648082 | 0.000142649 |
| B1AT36 | 66997 | PSMD12 | 0.716502745 | 0.000504223 |
| Q8R326 | 66645 | PSPC1 | 0.716483363 | 0.000103983 |
| Q7TMY8 | 59026 | HUWE1 | 0.716438587 | 0.000584126 |
| G3X926 | 67239 | RPF2 | 0.716341757 | 0.000854266 |
| Q3TJD4 | 11950 | ATP5F1 | 0.716302626 | 0.000156097 |
| P61089 | 93765 | UBE2N | 0.716242419 | 0.000852814 |
| Q3U878 | 70248 | DAZAP1 | 0.716018594 | 0.000521651 |
| O09131 | 14873 | GSTO1 | 0.715998168 | 2.20707E-05 |
| E9QLZ0 | 216148 | SHC2 | 0.715993856 | 7.60131E-05 |
| P97352 | 20196 | S100A13 | 0.715952946 | 0.005904916 |
| Q9JJ89 | 108673 | CCDC86 | 0.715952064 | 0.000776258 |
| P14115 | 26451 | RPL27A | 0.715812283 | 0.000174994 |
| Q9JKR6 | 12282 | HYOU1 | 0.715796107 | 3.15989E-07 |
| P51859 | 15191 | HDGF | 0.715680843 | 0.000321057 |
| P52196 | 22117 | TST | 0.715655313 | 0.001108008 |
| Q9QZB7 | 56444 | ACTR10 | 0.715637358 | 0.001194675 |
| P19324 | 12406 | SERPINH1 | 0.715555586 | 5.97251E-07 |
| Q8BMJ2 | 107045 | LARS | 0.715502197 | 8.5872E-05 |
| P60487 | 57028 | PDXP | 0.715486526 | 0.003759081 |
| J3QPZ8 | 20918 | EIF1 | 0.715461118 | 0.000775115 |
| Q3U3L3 | 20719 | Serpinb6a | 0.715423958 | 2.50587E-05 |
| P31324 | 19088 | PRKAR2B | 0.715388112 | 0.004027186 |
| P68373 | 22146 | TUBA1C | 0.715352629 | 6.51965E-07 |
| Q3ULT8 | 18034 | NFKB2 | 0.715317943 | 0.002680758 |
| Q5D098 | 19177 | PSMB7 | 0.715278733 | 0.003392192 |
| Q01730 | 20163 | RSU1 | 0.715172956 | 0.001531318 |
| F6RPJ9 | 15925 | IDE | 0.715115783 | 2.51045E-05 |
| Q8CGK3 | 74142 | LONP1 | 0.715059707 | 3.65353E-05 |
| F8WH20 | 71720 | OSBPL3 | 0.715002221 | 0.000173314 |
| Q8R436 |  |  | 0.714972894 | 7.55455E-05 |
| P62827 | 19384 | RAN | 0.714875157 | 7.79947E-05 |
| Q61833 | 20014 | RPN2 | 0.714798964 | 8.50058E-06 |
| Q3USC0 | 234549 | HEATR3 | 0.714750477 | 0.002068022 |
| Q9QUR6 | 19072 | PREP | 0.714666887 | 1.83029E-05 |
| Q8K2Z4 | 68298 | NCAPD2 | 0.714607267 | 1.01887E-05 |
| D3Z0F5 | 26893 | COPS6 | 0.714549919 | 0.001593803 |
| Q9D5T0 | 67979 | ATAD1 | 0.714434255 | 0.003304568 |
| A2ATT5 | 53893 | NUDT5 | 0.714379774 | 0.002035523 |
| Q9WVE8 | 23970 | PACSIN2 | 0.714329958 | 0.0048843 |
| A2AP78 | 15354 | HMGB3 | 0.714296939 | 0.000373451 |
| P59017 | 94044 | BCL2L13 | 0.714262693 | 8.65225E-05 |
| Q3UUX9 | 14569 | GDI2 | 0.714233179 | 0.000185896 |
| Q9D7S9 | 76959 | CHMP5 | 0.71420682 | 0.00359847 |
| B2RUJ7 | 22436 | XDH | 0.714199131 | 0.000157977 |
| Q9CQ60 | 66171 | PGLS | 0.714151608 | 0.001641974 |
| Q05BH6 | 21453 | TCOF1 | 0.714025958 | 0.001939026 |
| Q3TKU6 | 65114 | VPS35 | 0.71389771 | 0.000183754 |
| Q6ZWS7 | 12325 | CAMK2G | 0.713890139 | 0.001122665 |
| Q8VCH8 | 67812 | UBXN4 | 0.713795525 | 0.001665039 |
| P61358 | 19942 | RPL27 | 0.71366665 | 0.003496408 |
| Q9QZ82 | 13070 | CYP11A1 | 0.713657709 | 0.000860953 |
| A6PWC3 | 230598 | NRD1 | 0.7136526 | 0.000179464 |
| Q3U4W8 | 22225 | USP5 | 0.713555077 | 0.000137794 |
| P53811 | 56305 | PITPNB | 0.713485444 | 0.001222863 |
| Q6P5B0 | 107094 | RRP12 | 0.713470535 | 0.000445621 |
| Q3TGI9 | 66409 | RSL1D1 | 0.713447935 | 0.005441938 |
| Q61210 | 16801 | arhgef1 | 0.713432193 | 0.002397613 |
| P62911 | 19951 | RPL32 | 0.7133892 | 0.000511524 |
| P70279 | 20935 | SURF6 | 0.713387219 | 0.001504587 |
| E9PUH0 | 97159 | A430005L14Rik | 0.713386476 | 0.000326878 |
| Q9D6R2 | 67834 | IDH3A | 0.713350278 | 4.76498E-05 |
| F8VQJ3 | 226519 | LAMC1 | 0.713346289 | 0.000305753 |
| Q99LJ0 | 80281 | CTTNBP2NL | 0.713311101 | 0.004469704 |
| Q3TA75 | 23879 | FXR2 | 0.713287763 | 0.003449413 |
| Q9WVM1 | 26934 | RACGAP1 | 0.713284896 | 0.00264231 |
| Q9D0B6 | 67683 | PBDC1 | 0.7132696 | 0.000392678 |
| Q8BMF4 | 235339 | DLAT | 0.713266179 | 4.58013E-06 |
| Q3THH0 | 102545 | CMTM7 | 0.71324851 | 0.004620914 |
| Q3TIV5 | 69082 | ZC3H15 | 0.713145529 | 0.000953182 |
| Q8BTE1 | 69674 | MIF4GD | 0.713114106 | 0.004639669 |
| P37040 | 18984 | POR | 0.713043748 | 7.76958E-06 |
| Q8BTY3 | 67895 | PPA1 | 0.713026155 | 9.22807E-05 |
| A2ALF0 | 68598 | DNAJC8 | 0.712953839 | 4.34056E-05 |
| Q921M7 | 223601 | FAM49B | 0.712899287 | 0.000371072 |
| Q9R0X4 | 56360 | ACOT9 | 0.712885362 | 0.004212097 |
| Q3TCW5 | 192292 | NRBP1 | 0.712805345 | 0.000350454 |
| Q3UL43 | 170762 | NUP155 | 0.712714317 | 0.000209139 |
| Q9D1D4 | 68581 | TMED10 | 0.712709835 | 0.000177288 |
| Q9JHU4 | 13424 | DYNC1H1 | 0.712650569 | 8.20481E-06 |
| P05132 | 18747 | PRKACA | 0.712631035 | 0.00234699 |
| Q8C5B5 |  |  | 0.712628217 | 0.000261742 |
| Q80U72 | 105782 | SCRIB | 0.712616783 | 0.001742493 |
| E3SRG7 | 17128 | SMAD4 | 0.712386489 | 0.00076138 |
| P62334 | 67089 | PSMC6 | 0.712334108 | 2.24757E-06 |
| P46664 | 11566 | ADSS | 0.712315152 | 0.000126101 |
| Q8C5Q4 | 231413 | GRSF1 | 0.712302906 | 0.000206284 |
| Q9JKF1 | 29875 | iqgap1 | 0.712187421 | 1.45955E-05 |
| O08583 | 21681 | ALYREF | 0.712178912 | 0.004737557 |
| Q6P8N8 | 270166 | CLPX | 0.712108525 | 0.000653085 |
| A7VL18 | 56752 | ALDH9A1 | 0.712073373 | 0.000790874 |
| Q3TN31 | 26444 | PSMA7 | 0.7120723 | 3.57252E-05 |
| B7ZNW0 | 72542 | PGAM5 | 0.712045958 | 0.000415306 |
| Q9D172 | 28295 | D10Jhu81e | 0.711964689 | 0.00085912 |
| Q91VG6 | 66075 | CHCHD3 | 0.711845242 | 0.004546147 |
| Q8BZA9 | 319801 | TIGAR | 0.711840881 | 0.000309485 |
| P18760 | 12631 | CFL1 | 0.711594668 | 4.87566E-05 |
| Q3U5R8 | 55980 | IMPA1 | 0.711536242 | 0.000114958 |
| Q3TDN2 | 76577 | FAF2 | 0.711430463 | 0.000366097 |
| Q8K2H2 | 72201 | OTUD6B | 0.711333169 | 0.000235976 |
| Q8R010 | 231872 | AIMP2 | 0.711320822 | 0.003699591 |
| Q3US65 | 20815 | SRPK1 | 0.71132057 | 0.000219987 |
| P84096 | 56212 | RHOG | 0.711273554 | 0.001137343 |
| P11983 | 21454 | TCP1 | 0.711126825 | 2.74808E-05 |
| Q99KR3 | 212442 | LACTB2 | 0.711015146 | 0.000165423 |
| E9QP59 | 380664 | lemd3 | 0.710909657 | 0.006647263 |
| Q9CXJ4 | 74610 | ABCB8 | 0.710876743 | 0.00393263 |
| Q8C1E7 | 215210 | tmem120a | 0.710839247 | 0.001072471 |
| Q60737 | 12995 | CSNK2A1 | 0.710760467 | 0.000238696 |
| P52431 | 18971 | POLD1 | 0.710755196 | 8.45535E-05 |
| Q6P7V9 | 70099 | SMC4 | 0.710691347 | 0.000179316 |
| Q8R038 | 60315 | myg1 | 0.710654352 | 0.00014812 |
| Q9EP69 | 83493 | SACM1L | 0.710646997 | 0.005869177 |
| Q9D2R8 | 14548 | MRPS33 | 0.710625318 | 0.00874325 |
| Q3U9R7 | 14782 | GSR | 0.710574186 | 0.002162316 |
| G3X9B1 | 217995 | HEATR1 | 0.710430975 | 0.000124902 |
| O54984 | 56495 | ASNA1 | 0.710409345 | 0.001113324 |
| Q8CG48 | 14211 | SMC2 | 0.710343921 | 8.48138E-06 |
| Q91VH6 | 76890 | MEMO1 | 0.710239639 | 0.000409763 |
| P62754 | 20104 | RPS6 | 0.710037138 | 0.00027433 |
| Q9CZ44 | 386649 | NSFL1C | 0.709985183 | 7.74173E-05 |
| Q3TWN8 | 56454 | ALDH18A1 | 0.709838022 | 7.09997E-05 |
| D3YU17 | 103425 | NCLN | 0.709820129 | 0.008679061 |
| P45952 | 11364 | ACADM | 0.709730182 | 0.000607938 |
| Q6ZQ38 | 71902 | CAND1 | 0.709705029 | 1.60297E-05 |
| Q3TIC8 | 22273 | UQCRC1 | 0.709636201 | 0.001654821 |
| P24369 | 19035 | PPIB | 0.709577297 | 1.12175E-06 |
| Q3UPL0 | 69162 | SEC31A | 0.709406146 | 6.94249E-05 |
| Q3UF82 | 26413 | MAPK1 | 0.709380089 | 0.000151367 |
| Q6P4S8 | 68510 | INTS1 | 0.709347674 | 0.000941211 |
| Q6PB66 | 72416 | LRPPRC | 0.709329394 | 8.22913E-07 |
| Q3USP3 | 78244 | DNAJC21 | 0.709328833 | 0.004876734 |
| P15864 | 50708 | HIST1H1C | 0.709325028 | 0.000443146 |
| Q9Z1F9 | 50995 | UBA2 | 0.709320364 | 0.003353142 |
| Q9D8B3 | 75608 | CHMP4B | 0.709273725 | 0.001762713 |
| P35278 | 19345 | RAB5C | 0.709252089 | 0.006482023 |
| A0A087WQQ5 | 16709 | KTN1 | 0.709148114 | 0.000127893 |
| Q9Z2G9 | 53415 | HTATIP2 | 0.709117134 | 0.005629614 |
| Q99LP6 | 17713 | GRPEL1 | 0.709032924 | 0.000199774 |
| Q91YZ2 | 13017 | CTBP2 | 0.709026955 | 0.005605359 |
| Q80X90 | 286940 | FLNB | 0.708920759 | 0.000115768 |
| P80318 | 12462 | CCT3 | 0.70890239 | 4.33526E-06 |
| P17742 | 268373 | PPIA | 0.708846307 | 2.21115E-06 |
| Q3TFE8 | 16211 | KPNB1 | 0.708810547 | 8.60415E-06 |
| E9Q5I9 | 23997 | PSMD13 | 0.708774579 | 8.33166E-05 |
| Q80TX4 |  |  | 0.708750551 | 0.003730889 |
| Q9CQU0 | 66073 | TXNDC12 | 0.708742535 | 0.000195986 |
| A0PJ57 | 70333 | CD3EAP | 0.708695189 | 0.000578578 |
| Q3THP1 | 75221 | DPP3 | 0.708690815 | 1.07017E-05 |
| Q62351 | 22042 | TFRC | 0.708657447 | 0.000205591 |
| B1ATZ0 | 15239 | HGS | 0.708643701 | 5.63176E-05 |
| Q9CZT6 | 66497 | CMSS1 | 0.708600955 | 0.000244909 |
| Q7TMH5 | 68537 | MRPL13 | 0.708600641 | 0.00573315 |
| O08795 | 19089 | PRKCSH | 0.708525773 | 3.12773E-05 |
| Q9CQ48 | 52653 | NUDCD2 | 0.708449283 | 0.00150592 |
| E9PZF0 |  |  | 0.708306569 | 0.004460058 |
| Q3TJF2 | 67059 | OLA1 | 0.708274454 | 0.000454401 |
| Q8CHP8 | 67078 | PGP | 0.708262291 | 0.000363007 |
| Q61553 | 14086 | FSCN1 | 0.708249376 | 5.89782E-05 |
| Q3UID0 | 68094 | SMARCC2 | 0.70819175 | 0.000622202 |
| Q99LC5 | 110842 | ETFA | 0.707989005 | 3.79289E-05 |
| Q3U4F0 | 94280 | SFXN3 | 0.707946726 | 0.003743694 |
| Q9R1P4 | 26440 | PSMA1 | 0.707888512 | 0.000114941 |
| P08249 | 17448 | MDH2 | 0.707878007 | 6.98372E-06 |
| P53569 | 12607 | CEBPZ | 0.707847502 | 4.0293E-05 |
| Q8BX02 | 235041 | KANK2 | 0.707774171 | 0.001262467 |
| Q8CH18 | 67500 | CCAR1 | 0.707633132 | 0.000301681 |
| Q8VCQ8 | 109624 | CALD1 | 0.707617434 | 0.000229529 |
| O35129 | 12034 | PHB2 | 0.70754219 | 8.55174E-06 |
| Q3T9L0 | 68278 | ddx39 | 0.707535011 | 0.003725746 |
| Q8BK72 | 218506 | MRPS27 | 0.707498927 | 0.000303494 |
| Q8BH86 | 217830 | 9030617O03Rik | 0.707358717 | 0.00184633 |
| Q8VCF0 | 228607 | MAVS | 0.707225345 | 0.000373147 |
| E9QKZ2 | 226432 | IPO9 | 0.70715908 | 5.0723E-05 |
| P99028 | 66576 | Uqcrh | 0.707150923 | 0.000828041 |
| P49312 | 15382 | HNRNPA1 | 0.707132497 | 2.66509E-05 |
| G3UX26 | 22334 | VDAC2 | 0.707040976 | 2.31868E-06 |
| B2RQS1 | 94186 | STRN3 | 0.706962002 | 0.003307549 |
| Q6PFD9 | 269966 | NUP98 | 0.706812724 | 0.000625928 |
| F8VQC1 | 66661 | SRP72 | 0.70657287 | 0.000513111 |
| Q00422 | 14390 | GABPA | 0.70646567 | 0.002884327 |
| Q9WV55 | 30960 | VAPA | 0.70644532 | 0.000116012 |
| P14131 | 20055 | RPS16 | 0.706391905 | 0.000235604 |
| P60335 | 23983 | PCBP1 | 0.706370563 | 4.51609E-06 |
| Q91YR7 | 68879 | PRPF6 | 0.706224014 | 0.000848127 |
| Q3TJ52 | 19359 | RAD23B | 0.706151208 | 0.000157232 |
| Q3UT23 | 114893 | DCUN1D1 | 0.706147972 | 0.001368674 |
| E9Q6R3 | 20333 | SEC22B | 0.70613701 | 0.009245941 |
| P30416 | 14228 | FKBP4 | 0.706124966 | 2.39855E-05 |
| D3YXK2 | 224903 | SAFB | 0.706013686 | 0.001977143 |
| D3YZ09 | 68147 | GAR1 | 0.705800391 | 0.000258377 |
| P49722 | 19166 | PSMA2 | 0.705792003 | 0.005212979 |
| Q3TJ76 | 320951 | PISD | 0.705585391 | 0.000481015 |
| Q8BTF0 | 12847 | COPA | 0.705552644 | 1.30361E-05 |
| A2AJ72 | 320267 | FUBP3 | 0.705549788 | 0.001111421 |
| Q9JLI6 | 50880 | SCLY | 0.70546674 | 0.001939523 |
| Q5XJY5 | 213827 | ARCN1 | 0.705426341 | 5.74556E-05 |
| Q8K2M0 | 60441 | MRPL38 | 0.705414616 | 0.001896382 |
| Q3UGS9 | 20480 | CLPB | 0.705335704 | 0.00114695 |
| D3YUK4 | 68342 | NDUFB10 | 0.705313584 | 7.03611E-05 |
| B2RQ68 | 269593 | LUZP1 | 0.705227749 | 0.003821228 |
| P61082 | 22192 | UBE2M | 0.705151577 | 0.000442911 |
| P62962 | 18643 | PFN1 | 0.70508023 | 6.55344E-07 |
| Q9DBP5 | 66588 | CMPK1 | 0.704995486 | 2.48454E-05 |
| Q6ZQ58 | 73158 | LARP1 | 0.704915803 | 0.000169954 |
| Q3U281 | 66397 | SAR1B | 0.704892919 | 0.000183498 |
| Q9DAW9 | 71994 | CNN3 | 0.704880512 | 0.000470542 |
| Q62084 | 18938 | PPP1R14B | 0.704866484 | 0.00468954 |
| Q921F4 | 72692 | HNRNPLL | 0.704820767 | 0.000989681 |
| Q9DCR2 | 11777 | AP3S1 | 0.704781769 | 0.0001317 |
| P09671 | 20656 | SOD2 | 0.704766094 | 0.000183216 |
| Q3UDI8 | 17220 | MCM7 | 0.704724091 | 0.000430559 |
| P60670 | 217365 | NPLOC4 | 0.704631765 | 5.43872E-06 |
| Q8CJ26 | 67169 | Nradd | 0.704621836 | 0.000152905 |
| A7ISP9 | 78798 | EML4 | 0.704541528 | 5.97033E-05 |
| Q3THB3 | 76936 | HNRNPM | 0.704433216 | 9.92724E-05 |
| P14211 | 12317 | CALR | 0.704418254 | 3.85695E-06 |
| P97452 | 12181 | BOP1 | 0.704318961 | 0.007954588 |
| P80313 | 12468 | CCT7 | 0.704298313 | 1.16472E-05 |
| Q9CQV4 | 67998 | FAM134C | 0.704160348 | 0.004784493 |
| Q3V1M8 | 110611 | HDLBP | 0.704126333 | 8.97669E-06 |
| Q9D1G1 | 76308 | RAB1B | 0.704113535 | 3.65606E-05 |
| Q91WM3 | 27966 | RRP9 | 0.704051338 | 0.007067061 |
| Q9CRD2 | 66736 | EMC2 | 0.703947364 | 0.000386058 |
| P35700 | 18477 | PRDX1 | 0.703911195 | 1.06422E-05 |
| Q8K3X4 | 238330 | IRF2BPL | 0.703772556 | 0.000241902 |
| H3BKE1 | 269437 | PLCH1 | 0.703766727 | 0.000761261 |
| Q91VJ2 | 109042 | PRKCDBP | 0.703678998 | 0.000225816 |
| A0A068BFR6 | 100502766 | KIFC1 | 0.70363642 | 0.001183909 |
| E9PXY1 | 72584 | CUL4B | 0.703600751 | 0.004429729 |
| Q3TAD4 | 107652 | UAP1 | 0.703600349 | 0.000687996 |
| Q9DCU6 | 66163 | MRPL4 | 0.70355371 | 0.001149515 |
| D3YVN7 | 100041265 | Gm9755 | 0.70354173 | 0.00092918 |
| Q9WU62 | 16319 | INCENP | 0.703524561 | 0.000730153 |
| Q921M3 | 101943 | SF3B3 | 0.7033965 | 0.000418817 |
| Q80YC2 | 15516 | HSP90AB1 | 0.703382972 | 0.003745068 |
| Q9Z0W3 | 59015 | NUP160 | 0.7033507 | 0.002001342 |
| Q3TNH0 | 21917 | TMPO | 0.703341583 | 0.001743367 |
| Q3TKB7 | 66973 | MRPS18B | 0.703273825 | 0.001547932 |
| P26043 | 19684 | RDX | 0.703270362 | 5.60304E-05 |
| P05622 | 18596 | PDGFRB | 0.70326142 | 0.000661623 |
| Q3TAV1 | 93695 | GPNMB | 0.703216313 | 0.001196241 |
| F8WGB3 | 66052 | SDHC | 0.70321077 | 0.002461609 |
| Q3TEU8 | 23790 | CORO1C | 0.703183842 | 0.00065481 |
| Q8C570 | 66679 | RAE1 | 0.703110189 | 0.002647285 |
| Q9JLN9 | 56717 | MTOR | 0.703075813 | 0.006485188 |
| Q3ULJ0 | 333433 | GPD1L | 0.703032892 | 0.0003846 |
| Q8VCE7 | 73130 | TMED5 | 0.703017421 | 3.85795E-05 |
| Q8C788 | 170625 | SNX18 | 0.703009933 | 0.003461375 |
| G3UXZ5 | 19186 | PSME1 | 0.703007545 | 0.00029686 |
| Q921E2 | 106572 | RAB31 | 0.702932427 | 0.000125979 |
| Q6PGB6 | 72117 | NAA50 | 0.702862899 | 0.004977451 |
| Q3UJP8 | 14450 | GART | 0.702831737 | 2.77934E-05 |
| Q6ZWV7 | 66489 | RPL35 | 0.702729259 | 0.001960548 |
| Q3UQD0 | 53333 | TOMM40 | 0.702494583 | 0.000724157 |
| Q91W50 | 229663 | CSDE1 | 0.702420928 | 0.000136181 |
| Q9CX34 | 67955 | SUGT1 | 0.702269472 | 0.000536474 |
| Q3UA06 | 69716 | TRIP13 | 0.702262605 | 0.000334574 |
| Q3TE06 | 22388 | WDR1 | 0.702223338 | 2.50775E-05 |
| Q9CY64 | 109778 | BLVRA | 0.702164292 | 0.000627989 |
| Q99P72 | 68585 | RTN4 | 0.702000729 | 2.01727E-05 |
| A2BE93 | 56086 | SET | 0.701777074 | 6.54892E-05 |
| F8VPK0 | 218343 | TTC37 | 0.701760541 | 0.000521322 |
| P53702 | 15159 | HCCS | 0.70171256 | 0.00188721 |
| P54071 | 269951 | IDH2 | 0.701599632 | 5.4704E-06 |
| Q9QZ08 | 56174 | NAGK | 0.701571434 | 0.00375932 |
| Q3TIX9 | 28035 | USP39 | 0.701523264 | 0.003392716 |
| P99026 | 19172 | PSMB4 | 0.701494996 | 0.004820879 |
| Q9DBR1 | 24128 | XRN2 | 0.701476715 | 3.87448E-06 |
| P33174 | 16571 | kif4 | 0.701475805 | 0.000210915 |
| P97450 | 11957 | ATP5J | 0.701457563 | 0.000625116 |
| P51174 | 11363 | ACADL | 0.701438084 | 2.17655E-05 |
| Q3TG12 | 23874 | FARSB | 0.701364656 | 3.41328E-05 |
| Q9D8N0 | 67160 | EEF1G | 0.701350121 | 1.32962E-05 |
| P32067 | 20823 | SSB | 0.701343792 | 6.55128E-05 |
| Q9WTP6 | 11637 | AK2 | 0.7013107 | 0.004786382 |
| P97770 | 14911 | THUMPD3 | 0.701281475 | 0.000215954 |
| O35381 | 11737 | ANP32A | 0.701268909 | 3.75331E-05 |
| A2AHX9 | 12048 | BCL2L1 | 0.701240192 | 0.00362108 |
| F6TBV1 | 67075 | MAGT1 | 0.701217886 | 0.00011794 |
| Q8BU20 | 66046 | NDUFB5 | 0.70116428 | 0.000245322 |
| Q8C622 | 319188 | Hist1h2bp | 0.701158483 | 0.003261414 |
| Q8K411 | 69617 | PITRM1 | 0.701117397 | 0.001646218 |
| Q9CXW3 | 12301 | CACYBP | 0.701116841 | 0.000321245 |
| Q3U0B0 | 16498 | KCNAB2 | 0.701094155 | 0.000179429 |
| Q99N93 | 94063 | MRPL16 | 0.700992322 | 0.002431395 |
| P11499 | 15516 | HSP90AB1 | 0.700875212 | 7.9644E-05 |
| P62259 | 22627 | YWHAE | 0.700691502 | 6.12997E-06 |
| P10518 | 17025 | ALAD | 0.700663767 | 0.000243759 |
| Q8C2E7 | 223593 | E430025E21Rik | 0.700659561 | 7.10405E-05 |
| Q01320 | 21973 | TOP2A | 0.700639665 | 0.000326379 |
| P57759 | 67397 | ERP29 | 0.700594416 | 0.006406913 |
| E9Q6U4 | 109054 | PFDN4 | 0.700536864 | 0.004093565 |
| Q01853 | 269523 | VCP | 0.700532626 | 7.86465E-06 |
| Q3UJW9 | 58810 | AKR1A1 | 0.700528508 | 0.001474729 |
| Q9CW03 | 13006 | SMC3 | 0.70049496 | 5.09565E-05 |
| Q9ESP1 | 64136 | SDF2L1 | 0.700414631 | 0.000274578 |
| Q4FZL1 | 13681 | EIF4A1 | 0.700405702 | 6.68754E-05 |
| Q5QNU0 | 19069 | NUP88 | 0.7003523 | 0.0018651 |
| P62830 | 65019 | RPL23 | 0.700327251 | 0.00025924 |
| Q32P00 | 12417 | CBX3 | 0.700224155 | 0.000103608 |
| Q00PI9 | 68693 | HNRNPUL2 | 0.700168433 | 0.00012897 |
| Q3TXS7 | 70247 | PSMD1 | 0.700144748 | 0.000104424 |
| Q3UQU5 | 319322 | SF3B2 | 0.700142293 | 0.000460884 |
| G3X922 | 235567 | DNAJC13 | 0.700135765 | 0.006203798 |
| Q8CI86 | 18797 | plcb3 | 0.700124239 | 0.000988102 |
| E9QP99 | 269682 | GOLGA3 | 0.700038533 | 2.82259E-05 |
| P47962 | 100503670 | RPL5 | 0.699949638 | 1.82719E-05 |
| Q11136 | 18624 | PEPD | 0.699813656 | 0.001831059 |
| Q9R1P0 | 26441 | PSMA4 | 0.699811399 | 2.27553E-05 |
| Q5SUR0 | 237823 | PFAS | 0.699787656 | 0.001157563 |
| P62196 | 19184 | PSMC5 | 0.699782655 | 6.97085E-05 |
| Q6GQV8 | 74392 | SPECC1L | 0.699735295 | 0.00081921 |
| Q91V01 | 14792 | LPCAT3 | 0.699729103 | 0.00556432 |
| Q3UNJ3 | 14230 | FKBP10 | 0.699578187 | 0.000291209 |
| P17918 | 18538 | PCNA | 0.699550519 | 0.000108107 |
| A2AQE4 | 12848 | COPS2 | 0.699527335 | 0.001078903 |
| E9QMV2 | 73112 | ABRACL | 0.699523429 | 0.001107021 |
| Q61792 | 16796 | LASP1 | 0.699460123 | 0.001264559 |
| Q3UAZ7 | 97165 | HMGB2 | 0.69935618 | 0.000649753 |
| Q9CQD1 | 271457 | RAB5A | 0.699347775 | 0.000447437 |
| P35550 | 14113 | FBL | 0.6993038 | 3.25459E-05 |
| P97807 | 14194 | Fh1 | 0.699269495 | 0.000189381 |
| B7ZP20 | 11736 | ANKFY1 | 0.699229084 | 0.000264653 |
| P14576 | 24067 | Srp54a | 0.699143308 | 7.79339E-05 |
| P70444 | 12122 | BID | 0.699109182 | 0.000308289 |
| Q99PV0 | 192159 | PRPF8 | 0.699011718 | 0.001140815 |
| O35841 | 11800 | API5 | 0.69897619 | 5.88787E-05 |
| Q3TMX5 | 74840 | MANF | 0.698952142 | 7.65588E-05 |
| Q0QEW9 | 19899 | RPL18 | 0.698951341 | 3.08242E-05 |
| Q9CWJ9 | 108147 | ATIC | 0.698918767 | 9.84056E-05 |
| Q9D020 | 107569 | nt5c3 | 0.698903094 | 9.34148E-05 |
| Q99JY0 | 231086 | HADHB | 0.698894467 | 9.10326E-06 |
| P60710 | 11461 | ACTB | 0.698874306 | 1.695E-05 |
| Q03265 | 11946 | ATP5A1 | 0.698831761 | 1.0803E-05 |
| A0A068BFR3 | 19326 | RAB11B | 0.698822804 | 1.63129E-05 |
| B1AT03 | 16882 | LIG3 | 0.698739114 | 0.003296095 |
| Q9CR57 | 67115 | RPL14 | 0.698704637 | 6.06659E-05 |
| Q99KG1 | 74326 | HNRNPR | 0.698694889 | 0.000834889 |
| Q91V89 | 21770 | PPP2R5D | 0.698667769 | 0.003084087 |
| Q9QYJ3 | 81489 | DNAJB1 | 0.6986423 | 0.000781548 |
| P25206 | 17215 | MCM3 | 0.698604041 | 0.000428811 |
| B9EKP5 | 192176 | FLNA | 0.698568237 | 5.37526E-07 |
| Q8VCW8 | 264895 | ACSF2 | 0.698522757 | 0.000113 |
| Q3UJR8 | 218490 | BTF3 | 0.698520444 | 0.00185769 |
| Q8VC85 | 67207 | LSM1 | 0.698520286 | 5.93354E-05 |
| P60867 | 67427 | RPS20 | 0.698316527 | 0.00020457 |
| Q99JI6 | 215449 | RAP1B | 0.698282792 | 0.00054756 |
| Q99K48 | 53610 | NONO | 0.698259744 | 0.00038342 |
| P09405 | 17975 | NCL | 0.698186573 | 2.05589E-06 |
| Q9QWZ1 | 19355 | RAD1 | 0.698061477 | 0.008226595 |
| Q99KP6 | 28000 | PRPF19 | 0.698017106 | 0.001773657 |
| P08003 | 12304 | PDIA4 | 0.69789393 | 1.86193E-06 |
| Q99JB2 | 66592 | STOML2 | 0.697845454 | 0.000937699 |
| J3QQ30 | 18738 | pitpna | 0.697809338 | 0.001388499 |
| P68372 | 227613 | TUBB4B | 0.697797346 | 0.000780106 |
| Q8C7C4 |  |  | 0.6977549 | 8.19718E-05 |
| Q9D2G2 | 78920 | DLST | 0.697737644 | 0.000354156 |
| Q9D1M4 | 66143 | EEF1E1 | 0.697731876 | 0.000250927 |
| P63101 | 22631 | YWHAZ | 0.697709075 | 6.85984E-06 |
| Q3TBU6 | 13430 | DNM2 | 0.697702515 | 0.000777238 |
| Q8VE47 | 66663 | UBA5 | 0.697656714 | 0.00656609 |
| Z4YL78 | 75786 | CKAP5 | 0.697648504 | 0.000537997 |
| P51125 | 12380 | CAST | 0.697643139 | 0.000578373 |
| Q9CXJ1 | 67417 | EARS2 | 0.697607578 | 0.002744766 |
| Q3TFP0 | 14105 | SRSF10 | 0.697551817 | 0.003158533 |
| P62806 | 69386 | Hist1h4h | 0.697520847 | 2.70881E-06 |
| Q05816 | 16592 | FABP5 | 0.6974794 | 0.000482614 |
| P43247 | 17685 | MSH2 | 0.697458019 | 0.00012225 |
| Q3THK7 | 229363 | GMPS | 0.697338266 | 1.38272E-05 |
| P70372 | 15568 | ELAVL1 | 0.697235766 | 4.46078E-06 |
| Q8BJY1 | 66998 | PSMD5 | 0.697222473 | 0.000172854 |
| P54116 | 13830 | STOM | 0.697107611 | 0.0003913 |
| Q08509 | 13860 | EPS8 | 0.69704602 | 5.61085E-05 |
| Q3ULI5 | 52040 | PPP1R10 | 0.697041404 | 0.005712354 |
| Q6NVC2 | 21843 | TIAL1 | 0.696904652 | 0.00074818 |
| Q9WUL7 | 56350 | ARL3 | 0.696869874 | 0.001676148 |
| Q8K2B3 | 66945 | SDHA | 0.696709661 | 6.25858E-06 |
| Q9EQ06 | 114664 | HSD17B11 | 0.696619177 | 0.004606391 |
| Q8CIF2 | 107747 | ALDH1L1 | 0.696607395 | 0.00159282 |
| Q8JZN5 | 229211 | ACAD9 | 0.696545404 | 0.001023858 |
| Q8C1Y3 | 14958 | H1F0 | 0.69654446 | 2.53999E-06 |
| A1A4T2 | 14376 | GANAB | 0.696529809 | 1.74801E-05 |
| Q64674 | 20810 | SRM | 0.69647304 | 0.000100346 |
| D3YVV4 | 66848 | FUCA2 | 0.69646742 | 0.002317013 |
| B2CY77 | 16785 | RPSA | 0.696454957 | 6.76167E-05 |
| Q9CPQ8 | 27425 | ATP5L | 0.696441007 | 0.000808617 |
| Q76MZ3 | 51792 | PPP2R1A | 0.696415217 | 3.70104E-06 |
| A3KMJ8 | 22327 | VBP1 | 0.696413502 | 0.00141497 |
| P38647 | 15526 | HSPA9 | 0.696250214 | 8.15982E-07 |
| Q9D8X2 | 234388 | CCDC124 | 0.696156406 | 0.004835455 |
| Q8C1X9 | 11745 | ANXA3 | 0.696128599 | 0.000174895 |
| Q4V9X9 | 268449 | RPL23A | 0.695973808 | 0.000256785 |
| Q3U026 | 57377 | MOGS | 0.695925509 | 0.00013557 |
| Q8K366 | 50912 | EXOSC10 | 0.695912557 | 0.009838884 |
| O35855 | 12036 | BCAT2 | 0.695855984 | 0.000481493 |
| O35326 | 20384 | SRSF5 | 0.695828744 | 0.000857792 |
| Q61206 | 18475 | PAFAH1B2 | 0.695827453 | 0.001851584 |
| Q8K0C9 | 218138 | GMDS | 0.695812686 | 0.001377819 |
| Q9Z2I9 | 20916 | SUCLA2 | 0.695774995 | 0.000364444 |
| P20108 | 11757 | PRDX3 | 0.695726711 | 0.000294842 |
| Q3UMB9 | 319277 | A230046K03Rik | 0.695616606 | 0.000310716 |
| Q6PDG0 | 70699 | NUP205 | 0.695541539 | 0.006457413 |
| Q9CSC2 | 54198 | SNX3 | 0.695533658 | 0.004156294 |
| Q8BMG7 | 98732 | RAB3GAP2 | 0.695446745 | 0.00227396 |
| I6L960 | 140630 | UBE4A | 0.695439017 | 0.007030833 |
| Q3U868 | 11545 | PARP1 | 0.69542927 | 4.05169E-05 |
| Q921L3 | 68944 | TMCO1 | 0.695419428 | 0.00859177 |
| P27046 | 17158 | MAN2A1 | 0.695401836 | 1.41038E-05 |
| Q8C5U8 |  |  | 0.695360523 | 0.002456167 |
| Q9CQM9 | 30926 | GLRX3 | 0.695352419 | 0.00019074 |
| Q9D1P4 | 66917 | CHORDC1 | 0.695298162 | 0.000348022 |
| P31230 | 13722 | AIMP1 | 0.69522877 | 3.05069E-06 |
| Q80YG4 | 140740 | SEC63 | 0.695212565 | 7.63136E-05 |
| Q5SYD0 | 338367 | MYO1D | 0.695203437 | 6.45334E-05 |
| Q9CRY7 | 66569 | GDPD1 | 0.695138109 | 0.001573206 |
| Q922J3 | 56430 | CLIP1 | 0.695136139 | 0.004913851 |
| Q6P069 | 109552 | SRI | 0.695050473 | 4.16988E-05 |
| P53994 | 59021 | RAB2A | 0.695031026 | 0.000881013 |
| Q3THI5 | 19182 | PSMC3 | 0.695004974 | 7.24825E-05 |
| Q9DCM0 | 66071 | ETHE1 | 0.694955732 | 0.000134041 |
| P63168 | 56455 | DYNLL1 | 0.694953387 | 0.000664171 |
| Q8BK29 | 67763 | PRPSAP1 | 0.694877336 | 0.001935658 |
| P36552 | 12892 | CPOX | 0.69471156 | 0.000755655 |
| Q6R891 | 217124 | PPP1R9B | 0.694672028 | 0.002668712 |
| P15626 | 14863 | GSTM2 | 0.694650744 | 3.55116E-05 |
| O88848 | 56297 | ARL6 | 0.694567354 | 2.17081E-05 |
| Q91VJ4 | 106504 | STK38 | 0.694475722 | 0.004226799 |
| Q9DAR7 | 69305 | DCPS | 0.694449281 | 8.48698E-06 |
| A0A068BIU7 | 57315 | WDR46 | 0.694417394 | 0.000645732 |
| Q8BGS2 | 66162 | bola2 | 0.694267907 | 0.000534728 |
| Q80YR5 | 224902 | SAFB2 | 0.694234011 | 0.006422753 |
| Q3UW32 | 67628 | ANP32B | 0.694225513 | 0.000180054 |
| Q8CDZ5 | 234865 | NUP133 | 0.694177336 | 0.000305239 |
| Q8BH59 | 78830 | SLC25A12 | 0.694065923 | 4.26786E-05 |
| E9QNY8 | 50720 | SACS | 0.694018535 | 0.001265596 |
| D3Z041 | 14081 | ACSL1 | 0.69395243 | 0.000223471 |
| Q9QXK3 | 54160 | COPG2 | 0.6939325 | 0.000160084 |
| Q9DBR0 | 56399 | AKAP8 | 0.693842319 | 0.000759098 |
| Q8VDP6 | 52858 | CDIPT | 0.69375821 | 0.001465709 |
| Q6ZWQ9 | 67268 | MYL12A | 0.693718978 | 4.25915E-05 |
| Q9CPQ3 | 223696 | TOMM22 | 0.693708914 | 0.00284447 |
| Q8BI84 | 338366 | MIA3 | 0.693692261 | 1.40727E-05 |
| Q3UJS6 | 70568 | CPNE3 | 0.693673725 | 0.001016995 |
| Q9CT36 | 72119 | TPX2 | 0.693658883 | 0.005357884 |
| A0ZVB6 | 107182 | BTAF1 | 0.693544783 | 0.000322438 |
| Q93092 | 21351 | TALDO1 | 0.693527012 | 0.000376629 |
| A2AL12 | 229279 | HNRNPA3 | 0.693344428 | 3.92004E-05 |
| G5E896 | 234699 | EDC4 | 0.693245995 | 0.002250198 |
| Q8K3A8 | 57317 | SRSF4 | 0.693233456 | 0.00290377 |
| D6RHA2 | 19703 | RENBP | 0.693222678 | 0.005075278 |
| Q3THK3 | 98053 | GTF2F1 | 0.693151078 | 0.000184785 |
| P14069 | 20200 | S100A6 | 0.693134606 | 0.00431189 |
| Q3V222 | 218811 | SEC24C | 0.693035117 | 0.005417754 |
| P21619 | 16907 | LMNB2 | 0.693032289 | 0.000269765 |
| H3BIX4 | 20320 | NPTN | 0.693028928 | 0.002594444 |
| Q921S7 | 56280 | MRPL37 | 0.692949514 | 1.07024E-05 |
| D3Z3F8 | 229285 | SPG20 | 0.692919012 | 8.22738E-05 |
| P36916 | 14670 | GNL1 | 0.692896323 | 0.000376615 |
| Q9DAW6 | 70052 | PRPF4 | 0.692893098 | 0.001571894 |
| E9Q035 |  |  | 0.692880156 | 0.000119975 |
| Q8K3H0 | 72993 | APPL1 | 0.69284887 | 0.002177814 |
| Q3U6D2 | 100088 | RCC1 | 0.692842092 | 0.00219291 |
| Q8K1J6 | 70047 | TRNT1 | 0.69277568 | 0.000511253 |
| P50247 | 11615 | Gm4737 | 0.692753086 | 6.74899E-05 |
| P61161 | 66713 | ACTR2 | 0.692740772 | 0.000240307 |
| Q8CC03 | 11765 | AP1G1 | 0.692708138 | 0.000364271 |
| F6RJV6 | 71835 | LANCL2 | 0.692675073 | 0.000298083 |
| Q61166 | 13589 | MAPRE1 | 0.692616532 | 3.04418E-05 |
| Q8BHL5 | 140579 | elmo2 | 0.692594207 | 0.003900856 |
| A2RRK3 | 20874 | SLK | 0.692516054 | 0.000446955 |
| O89054 | 11461 | ACTB | 0.692419611 | 0.002216337 |
| Q8BU30 | 105148 | IARS | 0.692417043 | 2.71214E-05 |
| P35279 | 19346 | RAB6A | 0.692388397 | 0.001024772 |
| P62320 | 67332 | SNRPD3 | 0.692342953 | 6.10506E-06 |
| P14685 | 22123 | PSMD3 | 0.692342782 | 7.10089E-05 |
| Q3TJG6 | 56351 | PTGES3 | 0.692270768 | 0.000260199 |
| O35685 | 18221 | NUDC | 0.69223067 | 0.000205299 |
| Q8BKC5 | 70572 | IPO5 | 0.692143226 | 3.79271E-05 |
| Q60902 | 13859 | EPS15L1 | 0.692126281 | 0.002289882 |
| Q9Z0F7 | 20618 | SNCG | 0.692102264 | 0.000211745 |
| Q3TKY6 | 67285 | CWC27 | 0.692091037 | 0.001701318 |
| A2A5R2 | 99371 | ARFGEF2 | 0.692072869 | 0.001248545 |
| Q3TGW0 | 74117 | ACTR3 | 0.692056116 | 0.000363096 |
| Q8BPB0 | 68473 | MOB1B | 0.691987363 | 0.005287927 |
| P46935 | 17999 | NEDD4 | 0.691930075 | 0.000123336 |
| B9EIU1 | 107508 | EPRS | 0.691929566 | 2.69876E-06 |
| O88587 | 12846 | COMT | 0.691926319 | 0.000349791 |
| Q9WVG6 | 59035 | CARM1 | 0.691909787 | 0.001334569 |
| Q9CQ54 | 68197 | NDUFC2 | 0.691909703 | 0.003590376 |
| E9Q7G1 | 66676 | TMED7 | 0.691908736 | 0.000719332 |
| P46471 | 19181 | PSMC2 | 0.691867045 | 1.25562E-05 |
| P35505 | 14085 | FAH | 0.691829033 | 4.3741E-05 |
| E9PWY9 | 66590 | FARSA | 0.691737411 | 0.000530351 |
| P27048 | 20638 | SNRPB | 0.691728281 | 2.35889E-05 |
| Q61768 | 16573 | KIF5B | 0.691718773 | 0.001067411 |
| P50396 | 14567 | GDI1 | 0.691555299 | 0.009280829 |
| Q8BU88 | 216767 | MRPL22 | 0.691530939 | 0.003766157 |
| P62242 | 20116 | RPS8 | 0.691511819 | 4.69257E-06 |
| A2BDX2 | 13480 | DPM1 | 0.691498004 | 3.61082E-05 |
| Q3UA17 | 56428 | MTCH2 | 0.69148107 | 8.30698E-06 |
| Q8K0D5 | 28030 | GFM1 | 0.691468883 | 7.82691E-05 |
| P45376 | 11677 | Akr1b3 | 0.691354885 | 7.13864E-06 |
| Q3ULP8 | 78697 | PUS7 | 0.691316517 | 0.000601313 |
| Q9WUM5 | 56451 | SUCLG1 | 0.691249783 | 0.001076826 |
| P47754 | 12343 | CAPZA2 | 0.691232708 | 0.000417185 |
| Q9ER72 | 27267 | CARS | 0.691134417 | 3.34699E-05 |
| B2RWW1 | 270058 | MAP1S | 0.691111877 | 0.001115892 |
| B2RQ83 | 216766 | GEMIN5 | 0.691100363 | 6.29091E-05 |
| P47911 | 19988 | RPL6 | 0.691093247 | 5.75379E-05 |
| Q3UM18 | 224092 | LSG1 | 0.690906687 | 0.000169782 |
| G3UYV7 | 54127 | RPS28 | 0.690840683 | 1.50549E-05 |
| Q3TUI9 | 26442 | PSMA5 | 0.690821864 | 0.00033958 |
| P68254 | 22630 | YWHAQ | 0.69075196 | 1.42464E-06 |
| P28658 | 54138 | ATXN10 | 0.690744257 | 9.84598E-06 |
| P58389 | 110854 | PTPA | 0.690694387 | 0.000644045 |
| Q9JIF7 | 70349 | COPB1 | 0.690689807 | 1.74153E-05 |
| Q05186 | 19672 | RCN1 | 0.690677982 | 0.000175502 |
| P68040 | 14694 | RACK1 | 0.690653012 | 1.29523E-05 |
| Q91VA7 | 170718 | IDH3B | 0.690612484 | 5.27045E-05 |
| Q8BN64 | 65967 | EEFSEC | 0.690567822 | 0.005842642 |
| Q8R1B4 | 56347 | EIF3C | 0.690560091 | 0.000129774 |
| D3Z5M2 |  |  | 0.690524935 | 3.00351E-05 |
| Q8R016 | 104184 | BLMH | 0.690372194 | 0.000687567 |
| P26443 | 14661 | GLUD1 | 0.690344153 | 1.45365E-05 |
| Q99LB6 | 108645 | MAT2B | 0.690251782 | 0.008156027 |
| Q9CZH7 | 67622 | MXRA7 | 0.690216141 | 0.002335033 |
| Q3TSX8 | 28185 | tomm70a | 0.690119371 | 0.000152614 |
| Q99J77 | 94181 | NANS | 0.690117523 | 8.8239E-05 |
| Q3UNF6 | 66615 | ATG4B | 0.690100544 | 0.002423071 |
| Q60676 | 19060 | PPP5C | 0.690096534 | 5.33137E-05 |
| Q3UKJ7 | 74255 | SMU1 | 0.690065577 | 0.002347781 |
| Q9QXB9 | 13495 | DRG2 | 0.690044744 | 0.000226732 |
| Q9QXA5 | 50783 | LSM4 | 0.689812832 | 0.009499439 |
| O35134 | 20019 | POLR1A | 0.6897547 | 0.004602052 |
| Q921E4 | 13476 | REEP5 | 0.689482806 | 0.001476729 |
| Q3TF41 | 53605 | NAP1L1 | 0.689478049 | 9.69169E-05 |
| Q99KJ8 | 69654 | DCTN2 | 0.689476107 | 0.000492943 |
| Q9D903 | 69072 | EBNA1BP2 | 0.689458099 | 1.69726E-05 |
| Q3UL78 | 12540 | CDC42 | 0.68944035 | 3.24866E-05 |
| Q9D706 | 71919 | RPAP3 | 0.689427098 | 0.002347244 |
| Q91WS0 | 52637 | CISD1 | 0.689426892 | 0.000258431 |
| O54774 | 11776 | AP3D1 | 0.689365393 | 4.13781E-06 |
| D3YVW2 | 73124 | GOLIM4 | 0.689362481 | 0.001091289 |
| Q80VI1 | 384309 | TRIM56 | 0.689285665 | 0.005837635 |
| B9EHJ3 | 21872 | TJP1 | 0.68924796 | 1.02874E-05 |
| Q3TW28 | 22019 | TPP2 | 0.68921033 | 0.000103431 |
| Q9D1I6 | 68463 | MRPL14 | 0.689146009 | 0.000537063 |
| Q91YP0 | 217666 | L2HGDH | 0.689144978 | 0.000208171 |
| Q3TGI0 | 22594 | XRCC1 | 0.689131727 | 0.004532504 |
| P34022 | 19385 | RANBP1 | 0.688982765 | 1.28035E-05 |
| Q3U2G2 | 15525 | HSPA4 | 0.688852026 | 3.1454E-05 |
| P97742 | 12894 | CPT1A | 0.688762436 | 0.000793208 |
| H3BKK2 | 51944 | KNSTRN | 0.688700987 | 0.001422758 |
| Q62348 | 22099 | TSN | 0.688697683 | 0.000101365 |
| B1AU25 | 26926 | AIFM1 | 0.688689698 | 0.00090952 |
| E9PUC2 | 74205 | ACSL3 | 0.688389812 | 0.001196381 |
| Q5U5I3 | 68565 | MRPS18A | 0.688350391 | 0.006767713 |
| Q6PAR5 | 66691 | GAPVD1 | 0.688334927 | 0.003414907 |
| Q3TDE4 | 59287 | NCSTN | 0.688289706 | 0.003106779 |
| Q9DB20 | 28080 | ATP5O | 0.688276934 | 0.000324865 |
| P51150 | 19349 | Rab7 | 0.688242607 | 1.16219E-06 |
| Q08093 | 12798 | CNN2 | 0.688076757 | 0.001666525 |
| Q3UM45 | 66385 | PPP1R7 | 0.688041279 | 0.000240684 |
| O35459 | 51798 | ECH1 | 0.688003443 | 0.00772291 |
| Q2VPC9 | 19082 | PRKAG1 | 0.687987366 | 0.000814427 |
| Q3U3B9 | 67043 | SYAP1 | 0.6878488 | 0.005863864 |
| Q9D883 | 108121 | U2AF1 | 0.687829645 | 0.001137307 |
| A2AWT6 | 21429 | UBTF | 0.687703941 | 0.000246684 |
| Q8CHW4 | 224045 | EIF2B5 | 0.687703562 | 0.000360719 |
| Q8BTW3 | 72544 | EXOSC6 | 0.687595479 | 0.000933994 |
| Q6PAM1 | 109658 | TXLNA | 0.687582143 | 0.001224359 |
| P63001 | 19353 | RAC1 | 0.687535436 | 4.75563E-05 |
| Q3ULG5 | 17219 | MCM6 | 0.687479187 | 0.000484896 |
| Q9CRC8 | 67144 | LRRC40 | 0.68747278 | 0.000378986 |
| Q9ERE7 | 67943 | MESDC2 | 0.687437073 | 0.002880542 |
| O08553 | 12934 | DPYSL2 | 0.687407524 | 5.04653E-06 |
| Q3U9A8 | 56726 | SH3BGRL | 0.687379967 | 0.000657659 |
| A0A023T672 | 60365 | RBM8A | 0.687322175 | 0.005931027 |
| Q05D44 | 226982 | EIF5B | 0.687314335 | 8.49281E-05 |
| Q91VR5 | 104721 | DDX1 | 0.687275639 | 0.000201148 |
| Q9D2Y4 | 74568 | MLKL | 0.687061343 | 0.00027345 |
| Q8JZQ9 | 27979 | EIF3B | 0.686757133 | 0.000207199 |
| Q91V64 | 66307 | ISOC1 | 0.686743762 | 0.007105726 |
| A2AAN2 | 217337 | SRP68 | 0.68669857 | 6.70715E-05 |
| G3UWE1 | 106529 | TECR | 0.686596092 | 0.000441951 |
| Q91YR1 | 19230 | TWF1 | 0.68654618 | 0.002375359 |
| Q5SS83 | 14252 | FLOT2 | 0.686545401 | 0.003410911 |
| Q7TSB1 | 234730 | FUK | 0.686515099 | 0.006681375 |
| O08810 | 20624 | EFTUD2 | 0.686499794 | 3.5673E-05 |
| Q8BVQ0 | 66317 | WDR61 | 0.686487702 | 0.000333285 |
| P61961 | 67890 | UFM1 | 0.686386834 | 0.006166826 |
| Q7TT04 | 69912 | NUP43 | 0.68636422 | 0.003209427 |
| P63038 | 15510 | HSPD1 | 0.68635342 | 9.61136E-07 |
| P59708 | 66055 | SF3B6 | 0.686238138 | 0.005595573 |
| P70122 | 66711 | SBDS | 0.686136518 | 0.000597218 |
| Q60865 | 53872 | CAPRIN1 | 0.686113038 | 0.000122131 |
| Q3UK61 | 67041 | OXCT1 | 0.686110747 | 5.57822E-06 |
| Q99J09 | 70465 | WDR77 | 0.685995474 | 0.000213372 |
| Q3TJ21 | 69051 | PYCR2 | 0.685913186 | 0.001026616 |
| P48771 | 12866 | COX7A2 | 0.685729733 | 0.001747703 |
| Q3UJN6 | 17535 | mre11a | 0.685711954 | 0.002296466 |
| Q9JMH6 | 50493 | TXNRD1 | 0.685697602 | 0.000263568 |
| Q3TXH6 | 83796 | SMARCD2 | 0.68569146 | 0.005048292 |
| A2AMY5 | 68926 | UBAP2 | 0.68563246 | 0.000149934 |
| Q6P8X1 | 72183 | SNX6 | 0.685582877 | 7.0305E-05 |
| Q8CE96 | 66926 | TRMT6 | 0.685530156 | 0.002082603 |
| Q3TAW4 | 27054 | SEC23B | 0.685512535 | 0.002238364 |
| Q6IRT4 | 66085 | EIF3F | 0.685384946 | 0.000773476 |
| P80315 | 12464 | CCT4 | 0.685355324 | 0.000107168 |
| Q99M31 | 50497 | HSPA14 | 0.685313505 | 0.000204261 |
| Q3TVM1 | 67465 | SF3A1 | 0.685294011 | 7.89721E-05 |
| Q3UJZ7 | 67134 | NOP56 | 0.685195925 | 0.000148601 |
| Q8BH95 | 93747 | ECHS1 | 0.685184887 | 2.52277E-05 |
| P51660 | 15488 | HSD17B4 | 0.685072723 | 4.65938E-06 |
| P63325 | 67097 | RPS10 | 0.685043467 | 7.67857E-05 |
| Q7TPV4 | 18432 | MYBBP1A | 0.684748508 | 2.14922E-05 |
| Q8BWM0 | 96979 | PTGES2 | 0.684744038 | 4.73781E-05 |
| B7ZWF1 | 13205 | DDX3X | 0.684676381 | 5.48027E-05 |
| H7BWX9 | 170930 | SUMO2 | 0.684663114 | 0.001077994 |
| P26516 | 17463 | PSMD7 | 0.684623148 | 0.000151052 |
| Q5U5W6 |  |  | 0.684621229 | 0.004156804 |
| Q3UGL3 | 216705 | CLINT1 | 0.684617273 | 9.53291E-05 |
| Q9CT23 | 16341 | EIF3E | 0.684602164 | 0.000323699 |
| P80314 | 12461 | CCT2 | 0.684470495 | 9.23064E-05 |
| Q9CQF3 | 68219 | NUDT21 | 0.68446866 | 0.000399928 |
| P26350 | 19231 | PTMA | 0.684339167 | 8.60181E-05 |
| Q6P289 | 16396 | ITCH | 0.684329842 | 0.002543398 |
| Q3UT02 | 207214 | LARP4 | 0.684279309 | 0.001461648 |
| G3UZ26 | 20425 | SHMT1 | 0.68411083 | 0.000843297 |
| P20029 | 14828 | HSPA5 | 0.684030106 | 3.01451E-06 |
| Q04207 | 19697 | RELA | 0.683972024 | 0.003008807 |
| Q3UHZ3 | 13433 | DNMT1 | 0.683951167 | 0.007984273 |
| G3XA25 | 110460 | ACAT2 | 0.683949895 | 0.004107776 |
| Q14CH7 | 224805 | AARS2 | 0.683776901 | 0.000214441 |
| Q7TMK9 | 56403 | SYNCRIP | 0.683749879 | 3.40521E-06 |
| Q5M9L1 | 54217 | RPL36 | 0.683721262 | 0.000116014 |
| E9QME5 | 94093 | TRIM33 | 0.683695456 | 0.009190698 |
| O55029 | 50797 | COPB2 | 0.683687302 | 5.633E-06 |
| Q61753 | 236539 | PHGDH | 0.683641458 | 3.67311E-06 |
| Q3USX2 | 83602 | GTF2A1 | 0.683603203 | 0.000340413 |
| O70194 | 55944 | EIF3D | 0.683575803 | 0.0004377 |
| Q9Z2B9 | 56613 | RPS6KA4 | 0.683411293 | 0.001324727 |
| P80316 | 12465 | CCT5 | 0.683405312 | 3.68003E-06 |
| Q9JL35 | 50887 | HMGN5 | 0.683386685 | 0.000399558 |
| Q8BMZ7 | 66914 | VPS28 | 0.683343195 | 0.000611818 |
| Q5D0F3 | 21354 | TAP1 | 0.683304651 | 0.000106509 |
| Q922A3 | 71807 | TARS2 | 0.683265312 | 0.000599402 |
| Q8CGZ0 | 27967 | CHERP | 0.683248936 | 0.002402988 |
| E9QKA4 | 51796 | SRRM1 | 0.68318031 | 0.002395979 |
| Q8C5G6 | 54473 | TOLLIP | 0.683144696 | 0.00276551 |
| P97384 | 11744 | ANXA11 | 0.683142506 | 0.00054716 |
| Q9CYL5 | 384009 | GLIPR2 | 0.683131561 | 0.000477307 |
| O88844 | 15926 | idh1 | 0.683130794 | 0.000254695 |
| Q8C4B4 | 106840 | UNC119B | 0.683093364 | 0.00171954 |
| Q3TJ01 | 28088 | RTCB | 0.683026622 | 2.22256E-05 |
| F6ZDS4 | 108989 | TPR | 0.682998687 | 4.91234E-06 |
| Q6P9T6 | 170760 | ACBD3 | 0.682868775 | 0.000231836 |
| Q9Z2I0 | 56384 | LETM1 | 0.682868624 | 0.000228991 |
| Q6ZWX6 | 13665 | EIF2S1 | 0.682859493 | 6.80214E-05 |
| Q9DC23 | 66861 | DNAJC10 | 0.682797252 | 0.001075414 |
| Q99N15 | 15108 | HSD17B10 | 0.6827836 | 0.000190944 |
| P46061 | 19387 | RANGAP1 | 0.682770689 | 0.001055252 |
| Q3UTW6 | 13591 | EBF1 | 0.682770186 | 0.001194221 |
| P58044 | 319554 | IDI1 | 0.682470836 | 0.000964807 |
| P19253 | 22121 | RPL13A | 0.682394497 | 1.75848E-05 |
| Q9DC69 | 66108 | NDUFA9 | 0.682349631 | 0.001845558 |
| Q9Z204 | 15381 | HNRNPC | 0.682316994 | 1.71655E-05 |
| Q9DCA5 | 67832 | BRIX1 | 0.682311158 | 0.000569927 |
| Q3TJA9 | 83945 | DNAJA3 | 0.682302408 | 0.000421784 |
| A7M7Q8 | 64656 | MRPS23 | 0.682285466 | 0.002654075 |
| Q99KI0 | 11429 | ACO2 | 0.682210927 | 1.22502E-07 |
| P31786 | 13167 | DBI | 0.682109867 | 0.000836569 |
| Q99L45 | 67204 | EIF2S2 | 0.681991627 | 1.46799E-05 |
| Q02053 | 22201 | UBA1 | 0.681953339 | 6.13354E-06 |
| Q922D8 | 108156 | MTHFD1 | 0.681936201 | 4.79168E-06 |
| Q569Z5 | 212880 | DDX46 | 0.681873636 | 0.001441928 |
| P68510 | 22629 | YWHAH | 0.681854294 | 4.34111E-05 |
| Q3THG9 | 69684 | AARSD1 | 0.68178391 | 0.000241874 |
| Q9CZD3 | 353172 | GARS | 0.68174751 | 0.000177369 |
| A0A0A6YW80 | 74838 | NAA15 | 0.68167736 | 9.74344E-05 |
| A2RRJ4 | 72322 | XPO5 | 0.681663897 | 0.00036965 |
| Q6NS46 | 18572 | PDCD11 | 0.681636318 | 8.74035E-05 |
| D3Z619 |  |  | 0.681585807 | 8.8929E-05 |
| O54724 | 19285 | PTRF | 0.681553181 | 0.002836395 |
| P48962 | 11739 | SLC25A4 | 0.681550374 | 2.05517E-05 |
| Q3UHD6 | 76742 | SNX27 | 0.681477836 | 0.001309985 |
| Q9Z1Z2 | 20901 | STRAP | 0.681415097 | 0.000304816 |
| P97310 | 17216 | MCM2 | 0.68136427 | 7.16499E-05 |
| Q9CZ04 | 26894 | COPS7A | 0.681350558 | 0.000376681 |
| O35387 | 23897 | HAX1 | 0.681286594 | 0.001927723 |
| A0A0B4J1F2 |  |  | 0.681272815 | 0.000755272 |
| P35979 | 269261 | RPL12 | 0.681249499 | 0.001086589 |
| P27612 | 18786 | PLAA | 0.681065698 | 2.00978E-05 |
| Q8R0F6 | 67444 | ILKAP | 0.681047367 | 0.002696168 |
| Q9CWU9 | 69736 | NUP37 | 0.681018241 | 0.005003773 |
| Q3TJZ7 | 52009 | HN1L | 0.680981502 | 0.000356113 |
| P27659 | 27367 | RPL3 | 0.680948085 | 1.87417E-05 |
| Q9DCA4 | 20088 | RPS24 | 0.680930003 | 4.76067E-06 |
| Q3TTX0 | 17184 | MATR3 | 0.680881345 | 4.65411E-05 |
| Q8VBT9 | 68938 | ASPSCR1 | 0.680769355 | 0.000309509 |
| Q8VIJ6 | 71514 | SFPQ | 0.680716824 | 0.00013163 |
| Q61584 | 14359 | FXR1 | 0.68070812 | 0.000268531 |
| P47963 | 270106 | RPL13 | 0.680615031 | 0.000232883 |
| Q8BSX8 | 68018 | COL4A3BP | 0.680608456 | 0.000997238 |
| Q8C140 | 50799 | SLC25A13 | 0.680603628 | 0.001913945 |
| P49718 | 17218 | MCM5 | 0.680508751 | 2.29599E-05 |
| Q8R395 | 66398 | COMMD5 | 0.680342502 | 0.005911403 |
| P61924 | 56447 | COPZ1 | 0.680322189 | 7.04142E-05 |
| Q9WTI7 | 17913 | MYO1C | 0.680296522 | 3.05452E-05 |
| Q91W92 | 104445 | CDC42EP1 | 0.680282811 | 0.004955012 |
| O35737 | 59013 | HNRNPH1 | 0.680269108 | 5.36701E-05 |
| P59999 | 68089 | ARPC4 | 0.680264787 | 0.000525887 |
| Q8C878 | 22200 | UBA3 | 0.680235014 | 0.000182925 |
| Q9R1P3 | 26445 | PSMB2 | 0.680185994 | 5.21488E-05 |
| Q8BML9 | 97541 | QARS | 0.680141375 | 0.00059971 |
| Q9ERK4 | 110750 | CSE1L | 0.680018233 | 7.20022E-05 |
| Z4YLI8 | 74148 | CLUH | 0.67998619 | 0.003318203 |
| Q8BTI8 | 75956 | SRRM2 | 0.679963621 | 0.004970308 |
| Q91ZW3 | 93762 | SMARCA5 | 0.679826646 | 3.16645E-06 |
| Q8C2D1 | 15115 | HARS | 0.679766894 | 0.001675848 |
| Q3THQ5 | 20867 | STIP1 | 0.679731415 | 5.99281E-05 |
| Q3TXV1 | 21762 | PSMD2 | 0.679730117 | 7.12214E-05 |
| Q99LC3 | 67273 | NDUFA10 | 0.679672701 | 4.27167E-05 |
| Q8C2Q3 | 56275 | RBM14 | 0.67963005 | 0.009294568 |
| P61982 | 22628 | YWHAG | 0.679475006 | 0.000947465 |
| Q3TL58 | 21402 | Skp1a | 0.679434958 | 0.000667323 |
| Q9CR16 | 67738 | PPID | 0.679407628 | 6.20003E-05 |
| Q6PGL7 | 28006 | Fam21 | 0.679378205 | 0.001104901 |
| Q8BJW5 | 68979 | NOL11 | 0.679240069 | 0.000859664 |
| Q3TSX5 | 75416 | NOP14 | 0.679175151 | 7.30954E-05 |
| A2ABY3 | 68671 | PCYT2 | 0.679155489 | 0.001920858 |
| Q8BPS5 | 20501 | SLC16A1 | 0.678991792 | 0.000192 |
| P16332 | 17850 | MUT | 0.678962943 | 0.000828191 |
| Q9CSU0 | 70470 | RPRD1B | 0.678867648 | 0.00021324 |
| P55264 | 11534 | ADK | 0.67868681 | 0.001596827 |
| Q45VK5 | 16201 | ILF3 | 0.678595736 | 0.000132397 |
| O35218 | 51786 | CPSF2 | 0.678546812 | 0.000842612 |
| Q9Z1T1 | 11774 | AP3B1 | 0.678490429 | 0.000128759 |
| Q3TIU7 | 227197 | NDUFS1 | 0.678421463 | 2.84729E-05 |
| Q9D1H7 | 67604 | GET4 | 0.678360161 | 9.64393E-05 |
| P99027 | 67186 | RPLP2 | 0.678205101 | 5.08681E-06 |
| E9QAJ9 | 70497 | ARHGAP17 | 0.678167223 | 0.002630625 |
| P62315 | 20641 | SNRPD1 | 0.678100984 | 0.00041442 |
| Q9CR59 | 102060 | GADD45GIP1 | 0.678077394 | 0.000468978 |
| Q9CR68 | 66694 | Uqcrfs1 | 0.678022333 | 0.000294085 |
| P81117 | 53322 | NUCB2 | 0.677857621 | 2.32008E-05 |
| Q8CH25 | 66660 | SLTM | 0.67779893 | 0.004511929 |
| G3X9L6 | 100039281 | Gm10250 | 0.677775252 | 4.57938E-05 |
| Q9D0M3 | 66445 | Cyc1 | 0.67776353 | 0.001660988 |
| Q3UZJ4 | 26932 | PPP2R5E | 0.67772341 | 0.005519876 |
| Q9DCT8 | 68337 | CRIP2 | 0.677714497 | 0.000372142 |
| O89079 | 59042 | COPE | 0.677642476 | 0.003072312 |
| Q9DBC3 | 74157 | CMTR1 | 0.677521021 | 0.009022149 |
| Q9D104 | 66384 | SRP19 | 0.677504324 | 0.003179325 |
| Q9QZQ8 | 26914 | h2afy | 0.677489157 | 0.000726633 |
| Q6A068 | 71702 | CDC5L | 0.677457683 | 7.11642E-06 |
| Q80WJ7 | 67154 | MTDH | 0.677423998 | 2.3777E-05 |
| P61027 | 19325 | RAB10 | 0.677396204 | 2.56787E-05 |
| Q99J62 | 106344 | RFC4 | 0.677373947 | 0.000493378 |
| Q64010 | 12928 | CRK | 0.677265915 | 0.007814636 |
| P49717 | 17217 | MCM4 | 0.677256535 | 0.000495009 |
| P47740 | 11671 | ALDH3A2 | 0.677182511 | 0.000323078 |
| Q9D7X3 | 72349 | DUSP3 | 0.677156132 | 0.003024266 |
| Q8R050 | 14852 | GSPT1 | 0.677136356 | 0.002606693 |
| Q99KF1 | 67511 | TMED9 | 0.677132433 | 0.000415158 |
| Q3U3I6 |  |  | 0.67708936 | 0.000179467 |
| Q8VI84 | 57753 | NOC3L | 0.677082891 | 0.000250766 |
| Q8BRF7 | 76983 | SCFD1 | 0.6770719 | 0.000116413 |
| E9Q9A5 | 59047 | PNKP | 0.677070488 | 0.004075635 |
| Q3TWW8 | 67996 | SRSF6 | 0.677070278 | 0.000277929 |
| Q9WUD1 | 56424 | STUB1 | 0.677052689 | 0.001192157 |
| D3Z7P2 | 68539 | TMEM109 | 0.677019632 | 0.00023693 |
| Q7TPT7 | 22321 | VARS | 0.677018399 | 1.076E-05 |
| F8WHU9 | 22687 | ZPR1 | 0.676944544 | 8.37598E-05 |
| B2M1R7 | 18521 | PCBP2 | 0.676897952 | 0.000107485 |
| Q8CEG3 | 213895 | BMS1 | 0.676844617 | 0.00713772 |
| E9PX48 | 67299 | DOCK7 | 0.676839558 | 0.000115041 |
| Q60931 | 22335 | VDAC3 | 0.676823043 | 0.000551055 |
| A0A087WPL5 | 13211 | DHX9 | 0.67682169 | 0.000130583 |
| Q8K354 | 109857 | cbr3 | 0.676802112 | 0.000461329 |
| Q3TD41 | 217127 | KAT7 | 0.676739446 | 0.000901698 |
| Q3UK68 | 17938 | NACA | 0.676704762 | 0.001667671 |
| Q80Y81 | 68626 | ELAC2 | 0.676656598 | 0.000898547 |
| Q8K0Z7 | 70207 | taco1 | 0.676613542 | 0.000369456 |
| P45591 | 12632 | CFL2 | 0.676457837 | 0.001194129 |
| Q06138 | 12283 | CAB39 | 0.676443335 | 7.3724E-05 |
| Q3UJX2 | 66642 | CTNNBL1 | 0.67636919 | 0.000258542 |
| Q3TF81 | 54364 | RPP30 | 0.676307557 | 0.000553534 |
| Q810A7 | 72047 | DDX42 | 0.676204031 | 0.002238718 |
| Q8R5C5 | 226977 | ACTR1B | 0.676141538 | 0.001213917 |
| Q9QYB1 | 29876 | CLIC4 | 0.676040141 | 0.001138235 |
| P62137 | 19045 | PPP1CA | 0.675968144 | 0.001615304 |
| Q3UY05 | 225887 | NDUFS8 | 0.675840654 | 0.003152371 |
| Q8C1M0 | 71974 | PRMT3 | 0.675836779 | 0.001453646 |
| D3YWK1 | 74781 | WIPI2 | 0.67575135 | 0.004387247 |
| Q8C6B9 | 66538 | rps19bp1 | 0.675597174 | 0.001337865 |
| Q8CGP5 | 319173 | Hist1h2af | 0.675592588 | 4.7856E-05 |
| Q8K363 | 66942 | DDX18 | 0.675505767 | 2.66692E-05 |
| Q3ULZ3 | 107272 | PSAT1 | 0.675460573 | 0.000107349 |
| Q6PHZ1 | 319195 | RPL17 | 0.675455383 | 7.90996E-07 |
| Q9CRF5 | 73674 | WDR75 | 0.675440558 | 0.001644737 |
| Q3V3N5 | 230737 | GNL2 | 0.675379002 | 0.000468183 |
| Q3UVK0 | 226090 | ERMP1 | 0.675335373 | 0.001210297 |
| O55201 | 20924 | Supt5 | 0.675231429 | 6.12946E-05 |
| Q3THW7 | 68135 | EIF3H | 0.675164555 | 0.000637024 |
| Q9CQI7 | 20639 | SNRPB2 | 0.675074022 | 0.00358961 |
| Q99MR6 | 83701 | SRRT | 0.675031721 | 3.55406E-05 |
| O35130 | 14791 | EMG1 | 0.67488816 | 5.14499E-05 |
| Q3THU8 | 18674 | SLC25A3 | 0.674851017 | 1.59038E-05 |
| P26039 | 21894 | TLN1 | 0.674756153 | 1.5288E-05 |
| Q9CQS8 | 66212 | SEC61B | 0.674731408 | 0.001600652 |
| Q64105 | 20751 | SPR | 0.674729201 | 5.82641E-05 |
| A0A087WNZ7 | 14897 | TRIP12 | 0.674717504 | 0.001064483 |
| E9Q1M6 | 108857 | ANKHD1 | 0.674702841 | 0.004443193 |
| Q6P9Q4 | 234686 | FHOD1 | 0.674693777 | 4.30324E-06 |
| Q3TVV6 | 51810 | HNRNPU | 0.674591754 | 2.90718E-06 |
| E9QAI5 | 69719 | CAD | 0.674578599 | 8.76911E-07 |
| Q3UGC1 | 229707 | STRIP1 | 0.674519246 | 0.000970706 |
| Q9D0I8 | 69902 | MRTO4 | 0.674501058 | 0.000411288 |
| Q3UD67 | 234734 | AARS | 0.674323506 | 4.87981E-06 |
| Q3UFY8 | 52575 | TRMT10C | 0.674314913 | 0.001235684 |
| Q8BTV2 | 269061 | CPSF7 | 0.674301127 | 2.06622E-05 |
| Q8R1Q8 | 235661 | DYNC1LI1 | 0.674252858 | 3.55417E-06 |
| Q3UNN4 | 20588 | SMARCC1 | 0.674201325 | 0.002944543 |
| A2AW05 | 20833 | SSRP1 | 0.674080822 | 1.46068E-06 |
| P54775 | 23996 | PSMC4 | 0.674047077 | 0.000134898 |
| D3Z0A2 |  |  | 0.67393863 | 2.69772E-05 |
| Q9CYA0 | 76737 | CRELD2 | 0.673902026 | 0.000248071 |
| Q5FWK3 | 228359 | ARHGAP1 | 0.673838205 | 0.002056277 |
| P62281 | 27207 | RPS11 | 0.673754707 | 0.0001574 |
| A2AFI3 | 19655 | RBMX | 0.673752037 | 0.003100536 |
| P43274 | 50709 | HIST1H1E | 0.673728845 | 0.00049936 |
| Q8BP67 | 68193 | RPL24 | 0.673693266 | 4.41818E-05 |
| P24288 | 12035 | BCAT1 | 0.673671972 | 0.000263253 |
| P23591 | 22122 | TSTA3 | 0.673541996 | 0.000377309 |
| P62267 | 66475 | rps23 | 0.673470261 | 0.000372644 |
| A2AQ17 | 69702 | NDUFAF1 | 0.673445422 | 3.53029E-06 |
| E9Q9E1 | 208643 | EIF4G1 | 0.673416295 | 3.64013E-06 |
| B1AZI6 | 331401 | THOC2 | 0.673349814 | 2.06931E-05 |
| Q3UU20 | 217310 | HID1 | 0.67333763 | 0.000382973 |
| Q924T2 | 118451 | MRPS2 | 0.673330071 | 0.006519926 |
| P19157 | 14870 | GSTP1 | 0.673307521 | 7.04116E-05 |
| P54103 | 22791 | DNAJC2 | 0.673177526 | 0.000247545 |
| Q6DFW4 | 55989 | NOP58 | 0.673035239 | 2.84028E-06 |
| P67778 | 18673 | PHB | 0.67290913 | 8.39641E-05 |
| P62855 | 27370 | RPS26 | 0.672858358 | 4.3548E-05 |
| P47856 | 14583 | GFPT1 | 0.672596206 | 2.04482E-05 |
| Q8CFI7 | 231329 | POLR2B | 0.672593409 | 0.001866858 |
| Q9DB15 | 56282 | MRPL12 | 0.672564326 | 0.001213534 |
| Q61599 | 11857 | ARHGDIB | 0.6725571 | 0.000172214 |
| P23116 | 13669 | EIF3A | 0.672332103 | 2.46001E-05 |
| Q8BMJ3 | 66235 | EIF1AX | 0.67232437 | 0.009367135 |
| Q64442 | 20322 | SORD | 0.672280909 | 0.00409257 |
| A1A596 | 110816 | PWP2 | 0.672260528 | 0.006476195 |
| Q9D554 | 75062 | SF3A3 | 0.672205004 | 0.000180543 |
| Q6NVF9 | 432508 | CPSF6 | 0.672177619 | 0.0036606 |
| Z4YKT6 | 216820 | DHRS7B | 0.672034117 | 0.005550201 |
| P14152 | 17449 | MDH1 | 0.672026942 | 9.26081E-07 |
| Q9WU28 | 56612 | PFDN5 | 0.672013279 | 4.45778E-05 |
| Q64433 | 15528 | hspe1 | 0.671988084 | 3.58266E-05 |
| Q9D1M7 | 66120 | FKBP11 | 0.671933413 | 0.003963766 |
| Q91VC3 | 192170 | EIF4A3 | 0.671906174 | 0.000345035 |
| Q9CXW2 | 64655 | MRPS22 | 0.671851984 | 0.000479113 |
| A0A0A6YWP9 | 98258 | TXNDC9 | 0.671834259 | 0.003847624 |
| O70310 | 18107 | NMT1 | 0.671772815 | 0.001137152 |
| G3X956 | 114741 | Supt16 | 0.671760155 | 0.000108622 |
| Q3TT85 | 56361 | PUS1 | 0.671733281 | 0.002531123 |
| Q9R0P5 | 56431 | DSTN | 0.671718357 | 5.54722E-07 |
| Q9JHS9 | 66070 | CWC15 | 0.671648833 | 0.00444013 |
| Q8VDG8 | 330260 | PON2 | 0.671613626 | 0.000286107 |
| Q3TWP9 | 98878 | EHD4 | 0.671582441 | 0.000180073 |
| Q8R5L1 | 12261 | C1QBP | 0.671360241 | 6.23986E-06 |
| P47941 | 12929 | CRKL | 0.671314471 | 0.002231743 |
| A0A0A0MQ80 | 57815 | SPATA5 | 0.671245035 | 0.000517258 |
| Q8C0Z3 | 69207 | SRSF11 | 0.671244097 | 0.000234592 |
| Q9CPT4 | 28106 | MYDGF | 0.671239173 | 0.000381617 |
| Q68FC6 | 72515 | WDR43 | 0.671155199 | 0.00241747 |
| Q9CSH3 | 72662 | dis3 | 0.671125167 | 0.000706543 |
| Q3TQI7 | 227707 | BC005624 | 0.671040031 | 0.002240812 |
| E9QND8 | 56298 | ATL2 | 0.670969036 | 0.006433728 |
| Q3THA0 | 53356 | EIF3G | 0.670808047 | 0.000309224 |
| Q9D8S3 | 66251 | ARFGAP3 | 0.670784146 | 0.002383246 |
| Q9Z1Z0 | 56041 | USO1 | 0.670783777 | 5.11994E-06 |
| P26638 | 20226 | SARS | 0.670698229 | 1.18572E-05 |
| Q9D6S7 | 67871 | MRRF | 0.670688717 | 0.000219847 |
| A2ALB2 | 227656 | REXO4 | 0.670685569 | 0.000562151 |
| Q3U6X7 | 12566 | CDK2 | 0.670677913 | 0.003441067 |
| D3Z113 | 245945 | RBM47 | 0.670462028 | 0.00843282 |
| P47955 | 56040 | RPLP1 | 0.670411806 | 0.000341453 |
| Q9D051 | 68263 | PDHB | 0.67032484 | 8.1415E-05 |
| Q9EP82 | 57773 | WDR4 | 0.670319513 | 0.000692503 |
| Q8QZY1 | 223691 | EIF3L | 0.670278116 | 0.000186367 |
| Q3URN5 | 56321 | AATF | 0.670195965 | 0.000198711 |
| P63028 | 22070 | TPT1 | 0.670190951 | 0.000122608 |
| F6SVV1 | 670565 | Gm9493 | 0.67004501 | 0.000384285 |
| P51881 | 11740 | SLC25A5 | 0.670024996 | 6.29638E-06 |
| Q6P4T2 | 320632 | SNRNP200 | 0.6699973 | 0.000184532 |
| P63037 | 15502 | DNAJA1 | 0.669943671 | 0.000556265 |
| Q9Z2Y8 | 114863 | PROSC | 0.669884466 | 0.000417033 |
| Q9CRT8 | 73192 | XPOT | 0.669843104 | 0.000662549 |
| Q3UJK2 | 66870 | SERBP1 | 0.669785833 | 8.08705E-05 |
| Q3TUQ7 | 105787 | prkaa1 | 0.669772582 | 7.02684E-05 |
| Q3URX8 | 276919 | GEMIN4 | 0.669595714 | 0.003566324 |
| G3UYD0 | 14886 | GTF2I | 0.669567123 | 0.000903989 |
| P14869 | 11837 | RPLP0 | 0.669559433 | 2.62332E-05 |
| Q80XR5 | 22185 | U2AF2 | 0.669437337 | 0.000322577 |
| Q9D0G0 | 59054 | MRPS30 | 0.66942118 | 0.000100215 |
| P62908 | 27050 | RPS3 | 0.669381846 | 3.39826E-06 |
| Q9JLV5 | 26554 | CUL3 | 0.669357514 | 0.001014809 |
| D3Z4U3 | 53323 | UBE2K | 0.669234318 | 0.001836043 |
| P68181 | 18749 | PRKACB | 0.669232934 | 0.002853056 |
| P32233 | 13494 | DRG1 | 0.669229894 | 0.000348033 |
| Q8BKZ9 | 27402 | PDHX | 0.669185098 | 3.91943E-05 |
| Q61171 | 21672 | PRDX2 | 0.669128219 | 7.37431E-05 |
| P33610 | 19076 | PRIM2 | 0.669103262 | 0.003989606 |
| B2RY56 | 67039 | RBM25 | 0.669066889 | 1.84316E-05 |
| P10126 | 13627 | EEF1A1 | 0.669053582 | 6.44707E-06 |
| Q3TXS9 | 16898 | RPS2 | 0.669015822 | 3.02918E-05 |
| Q3TXN6 | 228410 | CSTF3 | 0.668914181 | 8.60769E-05 |
| Q8R3X4 | 21856 | TIMM44 | 0.668741027 | 0.000457047 |
| Q8JZX4 | 76938 | RBM17 | 0.668639082 | 0.001109768 |
| I7HLV2 | 110954 | RPL10 | 0.668568601 | 0.000314594 |
| Q61699 | 15505 | HSPH1 | 0.668403279 | 0.000224833 |
| Q9EPU0 | 19704 | UPF1 | 0.668401348 | 0.000353923 |
| Q3TGH6 | 14156 | FEN1 | 0.668398582 | 0.001824179 |
| A0AUN0 | 207352 | SEC23IP | 0.66818791 | 0.000771033 |
| G5E829 | 67972 | ATP2B1 | 0.668159817 | 7.78418E-05 |
| P28352 | 11792 | APEX1 | 0.668149045 | 4.63603E-05 |
| Q9CX86 | 77134 | HNRNPA0 | 0.668144263 | 0.002494278 |
| Q9ES97 | 20168 | RTN3 | 0.668106266 | 5.26431E-05 |
| Q8BJ71 | 71805 | NUP93 | 0.668073284 | 0.000368968 |
| D6RG99 | 14356 | TIMM10B | 0.668066495 | 0.001370599 |
| P63005 | 18472 | PAFAH1B1 | 0.66787092 | 0.000116996 |
| P58252 | 13629 | EEF2 | 0.667839279 | 1.4572E-05 |
| Q9CQV8 | 54401 | YWHAB | 0.667803774 | 1.5985E-05 |
| Q05BN2 | 18813 | PA2G4 | 0.6676105 | 2.11533E-05 |
| Q3TA68 | 225348 | WDR36 | 0.667514046 | 0.000757927 |
| Q8BFY6 | 67898 | PEF1 | 0.667117136 | 0.001012982 |
| Q3U3C4 | 20405 | SH3GL1 | 0.667076852 | 0.000330616 |
| Q91V47 |  |  | 0.667030496 | 0.002910015 |
| Q3T992 | 56085 | UBQLN1 | 0.667023166 | 0.002826951 |
| Q3V117 | 104112 | ACLY | 0.667000111 | 2.70048E-05 |
| O70251 | 55949 | EEF1B2 | 0.66682531 | 0.001342152 |
| Q61081 | 12539 | CDC37 | 0.666599909 | 1.25586E-06 |
| Q8BSL7 | 11841 | ARF2 | 0.66649793 | 5.6266E-05 |
| Q99K70 | 54170 | RRAGC | 0.666120676 | 0.000791009 |
| Q3UF95 | 224727 | BAG6 | 0.665862156 | 0.000176817 |
| Q3UMT7 | 15388 | HNRNPL | 0.665842874 | 0.000102109 |
| P58021 | 68059 | TM9SF2 | 0.665701808 | 0.008360105 |
| G3UVU2 | 20222 | SF3A2 | 0.665669432 | 0.000124008 |
| P70698 | 51797 | Ctps | 0.665551671 | 0.00030311 |
| G3UXW9 | 209318 | GPS1 | 0.66549866 | 0.001758244 |
| Q61074 | 14208 | PPM1G | 0.665478427 | 7.96894E-05 |
| E9Q852 | 17356 | AFDN | 0.66547552 | 0.00074532 |
| P24547 | 23918 | IMPDH2 | 0.665310818 | 2.67949E-05 |
| Q9Z2X1 | 98758 | HNRNPF | 0.665292378 | 6.33521E-08 |
| P51410 | 20005 | RPL9 | 0.66526805 | 2.55183E-05 |
| P04117 | 11770 | FABP4 | 0.665156327 | 7.99519E-06 |
| Q61249 | 18518 | IGBP1 | 0.665027844 | 0.001978803 |
| Q8BGH2 | 68653 | SAMM50 | 0.664969274 | 0.001022614 |
| O88477 | 140486 | IGF2BP1 | 0.664884503 | 6.65034E-05 |
| A2AU61 | 19383 | RALY | 0.664807309 | 5.87329E-05 |
| P10107 | 16952 | ANXA1 | 0.664796389 | 1.79575E-05 |
| D3YUM1 | 17995 | NDUFV1 | 0.664780165 | 2.12237E-06 |
| Q8CIG8 | 27374 | PRMT5 | 0.664778688 | 0.006623402 |
| Q3TL79 | 217737 | AHSA1 | 0.664740983 | 1.06059E-05 |
| Q3TDF8 | 225363 | ETF1 | 0.664703392 | 2.28215E-05 |
| Q8BH58 | 226591 | TIPRL | 0.664684343 | 0.001145937 |
| P57784 | 68981 | SNRPA1 | 0.664677959 | 0.001002203 |
| Q9R1T2 | 56459 | SAE1 | 0.664456646 | 0.001580494 |
| Q9JJA4 | 57750 | WDR12 | 0.664409829 | 0.002646166 |
| Q9DBU8 | 55946 | AP3M1 | 0.664357126 | 0.00242421 |
| E9PYI8 | 59025 | USP14 | 0.664110868 | 4.76333E-05 |
| Q60973 | 245688 | RBBP7 | 0.66391845 | 0.000800975 |
| Q8K2C6 | 68346 | SIRT5 | 0.663914116 | 0.004163956 |
| B2RUG7 | 22763 | ZFR | 0.663811678 | 0.00056335 |
| Q91WD5 | 226646 | NDUFS2 | 0.663688138 | 0.000495108 |
| Q3UYD0 | 72554 | UTP14A | 0.663568466 | 9.377E-06 |
| D3YVX4 | 98366 | SMAP1 | 0.663247934 | 0.00140794 |
| P06795 | 18669 | Abcb1b | 0.663242251 | 0.00022893 |
| Q3U617 | 57296 | PSMD8 | 0.663227661 | 0.000262057 |
| Q8BU31 | 72065 | RAP2C | 0.663196241 | 0.001625582 |
| Q8K265 | 216156 | WDR18 | 0.663129173 | 0.003837109 |
| Q922Q8 | 98238 | LRRC59 | 0.663108882 | 3.92537E-05 |
| Q8BYY4 | 69863 | TTC39B | 0.663090495 | 0.000976958 |
| Q8R4R6 | 69482 | NUP35 | 0.662912006 | 0.003161551 |
| P45377 | 14187 | Akr1b8 | 0.662621789 | 0.000631533 |
| Q61033 | 21917 | TMPO | 0.66262115 | 4.13539E-05 |
| Q9CQE8 | 68045 | 2700060E02Rik | 0.662498485 | 0.00019503 |
| Q9JIQ4 | 21335 | TACC3 | 0.662353224 | 0.000936997 |
| B2RQQ5 | 17755 | MAP1B | 0.662279697 | 4.7837E-06 |
| P61222 | 24015 | ABCE1 | 0.662271135 | 7.84125E-05 |
| Q9CZU6 | 12974 | CS | 0.662254917 | 0.000126785 |
| P63330 | 19052 | PPP2CA | 0.661865817 | 0.000314851 |
| Q9DB77 | 67003 | Uqcrc2 | 0.661829198 | 6.39569E-05 |
| Q9D338 | 56284 | MRPL19 | 0.661514391 | 0.000337688 |
| E9QAS4 | 107932 | CHD4 | 0.661272166 | 2.99622E-05 |
| Q9CQZ5 | 67130 | NDUFA6 | 0.661220996 | 0.000306974 |
| Q9D1R9 | 68436 | RPL34 | 0.661215035 | 0.000265083 |
| Q9WVS5 |  |  | 0.661198877 | 2.91061E-05 |
| P14148 | 19989 | RPL7 | 0.661177738 | 0.000231643 |
| Q3V460 | 228715 | Gm561 | 0.661172504 | 0.006710763 |
| Q8BTW7 | 227619 | MAN1B1 | 0.66104895 | 0.000296548 |
| Q3TKC5 | 231327 | PPAT | 0.661004106 | 0.001909667 |
| A0JLN6 | 213773 | TBL3 | 0.660869978 | 0.000661736 |
| Q6GQT9 | 211548 | nomo1 | 0.660777248 | 3.68153E-06 |
| Q3U0I9 | 231834 | SNX8 | 0.660751093 | 0.000809459 |
| Q5PRF0 | 320487 | HEATR5A | 0.660746644 | 0.001589879 |
| Q8VC94 | 67025 | RPL11 | 0.660725687 | 0.000138399 |
| O55142 | 57808 | RPL35A | 0.660691335 | 0.001743556 |
| P47915 | 19944 | RPL29 | 0.660564092 | 0.000340222 |
| Q62376 | 20637 | SNRNP70 | 0.660437483 | 0.003440376 |
| Q9D7V6 | 67973 | MPHOSPH10 | 0.660423093 | 0.000900384 |
| P62852 | 75617 | RPS25 | 0.660378733 | 0.000327387 |
| D3Z0M9 | 74351 | DDX23 | 0.660316774 | 0.000119203 |
| F6V084 | 72736 | TMX1 | 0.66011043 | 0.000388544 |
| Q9D8E6 | 67891 | RPL4 | 0.660107288 | 2.13577E-05 |
| J3QMC5 | 100019 | Mdn1 | 0.660075544 | 0.000256351 |
| Q9DCF9 | 67437 | SSR3 | 0.660021818 | 0.0035793 |
| O88569 | 53379 | HNRNPA2B1 | 0.659955974 | 4.48632E-07 |
| Q9EPE9 | 170759 | ATP13A1 | 0.659942744 | 0.000234455 |
| P05201 | 14718 | GOT1 | 0.659937144 | 0.00164348 |
| Q8CGF7 | 56070 | TCERG1 | 0.659863496 | 3.55075E-05 |
| F6XC25 | 319965 | CC2D1B | 0.659664269 | 0.002813963 |
| O88653 | 56692 | LAMTOR3 | 0.659657573 | 5.69915E-06 |
| F6ZQA3 | 101706 | NUMA1 | 0.659588822 | 0.007926127 |
| P39053 | 13429 | DNM1 | 0.659523233 | 0.000730804 |
| Q8BK67 | 108911 | RCC2 | 0.659471918 | 3.62687E-05 |
| P29341 | 18458 | PABPC1 | 0.659425601 | 1.02323E-05 |
| Q6PIP5 | 67429 | NUDCD1 | 0.65942338 | 0.000438958 |
| P62245 | 267019 | rps15a | 0.659294702 | 3.3817E-05 |
| Q921R2 | 68052 | RPS13 | 0.65923809 | 0.000665973 |
| Q5BKS2 | 19043 | PPM1B | 0.659182089 | 0.004661487 |
| Q3TJN6 | 69178 | SNX5 | 0.65917009 | 3.30054E-05 |
| Q8CGC6 | 68272 | RBM28 | 0.658918935 | 0.000536438 |
| Q8BL36 | 72722 | FAM98A | 0.65889142 | 0.000353797 |
| B2RTP7 | 16681 | KRT2 | 0.658865379 | 0.000266765 |
| Q6PDM2 | 110809 | SRSF1 | 0.658807375 | 2.40445E-05 |
| I4DCY6 | 18391 | SIGMAR1 | 0.6586174 | 0.001920882 |
| Q5EBG5 | 27176 | RPL7A | 0.658555336 | 0.000171425 |
| Q9D0R2 | 110960 | TARS | 0.658503736 | 1.0152E-05 |
| D3Z0B9 | 69748 | ALDH16A1 | 0.658232238 | 0.000497236 |
| D3Z4V1 | 53317 | plrg1 | 0.65820386 | 5.82733E-06 |
| Q9DCT2 | 68349 | NDUFS3 | 0.658184879 | 0.000133362 |
| Q61927 |  |  | 0.65794086 | 0.000244746 |
| Q8BGS0 | 67920 | MAK16 | 0.657846579 | 0.00030396 |
| E9QN31 | 110109 | NOP2 | 0.65772745 | 0.000558879 |
| Q8BHC7 | 76867 | RHBDD1 | 0.657709369 | 0.001199628 |
| P12265 | 110006 | GUSB | 0.657658555 | 0.002045436 |
| P17426 | 11771 | AP2A1 | 0.657653976 | 5.38187E-05 |
| Q5SSI6 | 217109 | UTP18 | 0.657647481 | 0.000655694 |
| Q8WTY4 | 109006 | CIAPIN1 | 0.657519308 | 0.009366306 |
| A2AN08 | 69116 | UBR4 | 0.657424293 | 6.34547E-05 |
| Q91WK1 | 66701 | spryd4 | 0.657404749 | 0.00130241 |
| Q3UXI9 | 67781 | ILF2 | 0.656890305 | 0.00052928 |
| Q9D880 | 66525 | TIMM50 | 0.656789551 | 0.000156256 |
| O70503 | 56348 | HSD17B12 | 0.656770717 | 1.78436E-05 |
| Q9CPP6 | 68202 | NDUFA5 | 0.656757087 | 0.000418824 |
| Q61655 | 13680 | DDX19A | 0.656613224 | 0.000164168 |
| Q6ZWU9 | 57294 | RPS27 | 0.656566074 | 0.00035937 |
| P62702 | 20102 | RPS4X | 0.656431869 | 2.44094E-05 |
| Q80UU6 | 72075 | OGFR | 0.656359662 | 0.003308205 |
| Q3UYV9 | 433702 | NCBP1 | 0.656272389 | 0.005438358 |
| P62900 | 114641 | RPL31 | 0.656083851 | 0.000125505 |
| A3KGQ6 |  |  | 0.655825722 | 0.000178303 |
| Q5XG71 | 70683 | UTP20 | 0.65580188 | 0.000404225 |
| Q3TAP5 | 101214 | TRA2A | 0.655655534 | 0.000428008 |
| Q3UGR5 | 76987 | HDHD2 | 0.655592768 | 0.000336075 |
| Q9CQW1 | 56418 | YKT6 | 0.655476914 | 0.001231993 |
| Q9R233 | 21356 | TAPBP | 0.655472038 | 0.000647047 |
| Q3UK38 | 66254 | DIMT1 | 0.655379661 | 0.001604742 |
| Q80SW1 | 229709 | AHCYL1 | 0.655297051 | 0.001198735 |
| Q5HZH2 | 68327 | TSR3 | 0.655168241 | 0.000538203 |
| Q9CWT6 | 71986 | DDX28 | 0.655165924 | 0.000442276 |
| Q8BKS9 | 52874 | PUM3 | 0.655055825 | 3.65911E-05 |
| Q9ERS2 | 67184 | NDUFA13 | 0.655041144 | 0.00084796 |
| F8VPX1 | 252870 | USP7 | 0.654780844 | 0.000824423 |
| Q9CXV9 | 76863 | DCUN1D5 | 0.654652623 | 0.006314697 |
| P61965 | 140858 | WDR5 | 0.654593074 | 0.004322111 |
| K7Q751 | 14083 | PTK2 | 0.654361486 | 0.000452971 |
| Q3U561 | 19896 | RPL10A | 0.654308315 | 3.11329E-05 |
| B8X349 | 70834 | SPAG9 | 0.654290274 | 0.00044445 |
| Q3UDX4 | 13204 | DHX15 | 0.654184529 | 0.000164049 |
| Q9ERR7 | 93684 | sep15 | 0.65418208 | 0.000651824 |
| Q3TZU7 | 66616 | SNX9 | 0.654058642 | 7.62379E-05 |
| Q9WVQ5 | 56369 | APIP | 0.653886419 | 0.001522255 |
| S4R1L5 | 12211 | BIRC6 | 0.653806634 | 0.001453759 |
| Q8R0A0 | 68705 | GTF2F2 | 0.653518107 | 0.002592442 |
| Q3UDM8 | 66646 | RPE | 0.653515432 | 0.001506399 |
| D3YWS7 | 69064 | FUOM | 0.653221576 | 0.000145111 |
| P13439 | 22247 | UMPS | 0.653127194 | 0.003708021 |
| Q9CZX8 | 20085 | RPS19 | 0.65305528 | 6.21626E-05 |
| Q9CQM5 | 52700 | TXNDC17 | 0.652973293 | 0.001074803 |
| B2RUG6 | 238130 | DOCK4 | 0.652839571 | 0.003703862 |
| Q8BH04 | 74551 | PCK2 | 0.652699251 | 0.006519684 |
| Q9WUV0 | 26429 | ORC5 | 0.652691631 | 0.008172603 |
| P58742 | 223921 | AAAS | 0.652418427 | 0.000721525 |
| P47857 | 18642 | PFKM | 0.652403713 | 0.001532511 |
| P62317 | 107686 | SNRPD2 | 0.652376187 | 0.0006483 |
| Q9DC50 | 74114 | CROT | 0.652243735 | 0.000452527 |
| P35293 | 19330 | RAB18 | 0.652137019 | 0.000387233 |
| Q9D7S7 | 68028 | RPL22L1 | 0.652077302 | 2.77135E-05 |
| A2BGI8 | 66101 | PPIH | 0.651974014 | 0.004038516 |
| Q3UGJ7 | 18226 | NUP62 | 0.651813405 | 0.000175313 |
| P62717 | 76808 | RPL18A | 0.651648089 | 0.000247468 |
| Q9JJI8 | 67671 | RPL38 | 0.651586028 | 0.000580351 |
| Q9QZ23 | 56748 | NFU1 | 0.651524022 | 0.003028629 |
| Q3TDD8 | 75705 | EIF4B | 0.651516203 | 0.000304332 |
| Q8BGB7 | 67870 | ENOPH1 | 0.651458905 | 0.000105601 |
| A2BE28 | 76130 | LAS1L | 0.65141038 | 0.000250653 |
| Q4FJZ2 | 16650 | KPNA6 | 0.651376579 | 0.006584976 |
| Q8BUM1 | 230908 | TARDBP | 0.651085306 | 1.10718E-05 |
| Q3U1J4 | 13194 | DDB1 | 0.650878005 | 1.29951E-05 |
| Q9CSN5 | 67418 | PPIL4 | 0.650630908 | 0.000159116 |
| P61290 | 19192 | PSME3 | 0.650587841 | 8.39053E-05 |
| Q3TE45 | 67680 | SDHB | 0.650495844 | 4.50459E-05 |
| Q3TX72 | 14229 | FKBP5 | 0.650068087 | 6.78218E-05 |
| Q8BK66 | 52513 | DDX56 | 0.64996952 | 0.000284622 |
| P62270 | 20084 | RPS18 | 0.649824979 | 2.2939E-06 |
| A2A547 | 19921 | RPL19 | 0.649726201 | 0.000702392 |
| Q99JX4 | 98221 | EIF3M | 0.649693628 | 5.69494E-05 |
| Q9CQN7 | 107733 | MRPL41 | 0.649620221 | 0.001681615 |
| P63276 | 20068 | RPS17 | 0.649609064 | 0.000237839 |
| P62918 | 26961 | RPL8 | 0.649584181 | 0.000437101 |
| Q03145 | 13836 | EPHA2 | 0.64943685 | 2.18534E-05 |
| Q80YR4 | 213753 | Zfp598 | 0.64934278 | 6.40884E-05 |
| Q3U5L3 | 67804 | SNX2 | 0.649253812 | 0.000641355 |
| Q3UHX2 | 231887 | PDAP1 | 0.649197621 | 4.93724E-05 |
| P61620 | 53421 | SEC61A1 | 0.648951091 | 0.005872452 |
| O70569 |  |  | 0.64887496 | 8.56318E-05 |
| Q91V55 | 20103 | RPS5 | 0.648820942 | 2.33505E-06 |
| Q9CTT4 | 17993 | NDUFS4 | 0.648815523 | 0.001244099 |
| O08582 | 14904 | GTPBP1 | 0.648648564 | 0.001549396 |
| D3YZC1 | 66904 | PCCB | 0.648505446 | 0.000645844 |
| Q9EPL8 | 233726 | IPO7 | 0.648410693 | 3.06225E-05 |
| Q9WV98 | 30056 | TIMM9 | 0.648366174 | 0.000926104 |
| Q80X95 | 68441 | RRAGA | 0.648329641 | 0.009947679 |
| Q9D0I9 | 104458 | RARS | 0.648129166 | 2.7753E-07 |
| Q4VA53 | 100710 | PDS5B | 0.648049051 | 5.07647E-05 |
| O08749 | 13382 | DLD | 0.647995763 | 0.000206346 |
| Q61686 | 12419 | CBX5 | 0.647995181 | 0.000200887 |
| Q8R059 | 74246 | GALE | 0.647847036 | 0.007258351 |
| Q91WQ3 | 107271 | YARS | 0.647530569 | 3.4019E-05 |
| Q9D967 | 67881 | MDP1 | 0.647457293 | 4.27076E-05 |
| Q6P9J9 | 105722 | ANO6 | 0.647077279 | 0.000960385 |
| Q3UEB3 | 67959 | PUF60 | 0.64692564 | 0.001264603 |
| Q9R1C7 | 56194 | PRPF40A | 0.64689329 | 0.001145574 |
| P83917 | 12412 | CBX1 | 0.646845012 | 0.000272111 |
| O35215 | 13202 | DDT | 0.646648555 | 0.002732241 |
| A0PJ90 | 53312 | NUB1 | 0.646594092 | 0.000151734 |
| F6QKD2 | 67755 | DDX47 | 0.646540474 | 0.006218145 |
| G3X8X7 | 80743 | VPS16 | 0.64629593 | 0.000108623 |
| Q921N6 | 228889 | DDX27 | 0.64606193 | 0.000153055 |
| Q3TK27 | 30877 | GNL3 | 0.646031164 | 8.25646E-05 |
| Q8VI75 | 75751 | IPO4 | 0.645934929 | 2.05133E-05 |
| Q3ULG4 | 19185 | PSMD4 | 0.6458484 | 6.52367E-05 |
| Q8BV40 | 74197 | GTF2E1 | 0.645755607 | 0.000152165 |
| P48758 | 12408 | CBR1 | 0.645570544 | 5.49493E-05 |
| A0A0A6YVU8 | 100043022 | Gm9774 | 0.645558523 | 0.000364761 |
| P70188 | 16579 | KIFAP3 | 0.645350405 | 0.000529291 |
| Q78JE5 | 71999 | FBXO22 | 0.64531104 | 0.000474332 |
| Q3UAY3 | 269252 | GTF3C4 | 0.645276839 | 0.000906685 |
| Q3TH64 | 66427 | CYB5B | 0.645170759 | 0.000272507 |
| Q9EQ61 | 64934 | PES1 | 0.645143702 | 0.00204034 |
| Q3V212 | 76457 | CCDC134 | 0.645089143 | 0.004225629 |
| A0A087WQE6 | 67923 | TCEB1 | 0.645080099 | 0.000200144 |
| Q3B7Z2 | 76303 | OSBP | 0.645058645 | 0.00091131 |
| Q91YS7 | 26396 | MAP2K2 | 0.644877338 | 0.000968503 |
| F6ZFU0 | 66656 | EEF1D | 0.644811799 | 0.000402614 |
| P49817 | 12389 | CAV1 | 0.644425497 | 0.000245966 |
| P54822 | 11564 | ADSL | 0.644356053 | 6.53382E-06 |
| Q7TT37 | 230233 | IKBKAP | 0.644242149 | 0.000375339 |
| P49962 | 27058 | SRP9 | 0.644093467 | 0.003036459 |
| Q6ZWZ6 | 20042 | RPS12 | 0.644081953 | 2.85055E-05 |
| Q9DD18 | 66044 | DTD1 | 0.644061131 | 0.000336329 |
| Q6PHN9 | 77407 | RAB35 | 0.643890839 | 0.00065682 |
| Q8VEE4 | 68275 | RPA1 | 0.643792448 | 0.000135266 |
| Q8BIF7 | 101612 | GRWD1 | 0.643596472 | 0.004048079 |
| A2AE27 | 109674 | AMPD2 | 0.643568554 | 0.000272316 |
| Q91VM5 | 19656 | RBMXL1 | 0.643513189 | 3.71587E-05 |
| E9Q715 | 192196 | LUC7L2 | 0.643390163 | 0.002263556 |
| Q9CYH6 | 59014 | RRS1 | 0.643268197 | 2.89226E-06 |
| A0JNY7 | 13690 | EIF4G2 | 0.642848599 | 5.34043E-05 |
| Q8R509 | 19205 | PTBP1 | 0.642763683 | 5.72695E-05 |
| P42128 | 17425 | FOXK1 | 0.642670023 | 0.009376113 |
| Q9DCB8 | 74316 | ISCA2 | 0.642327946 | 0.000541201 |
| Q99020 | 15384 | HNRNPAB | 0.642313459 | 2.97534E-08 |
| Q4FK16 | 22038 | PLSCR1 | 0.64216876 | 0.000530482 |
| Q8CE21 | 70769 | NOLC1 | 0.64213428 | 1.46472E-06 |
| P16254 | 20813 | SRP14 | 0.642010833 | 0.002386796 |
| Q99KD6 | 20463 | COX7A2L | 0.641983714 | 0.002140438 |
| Q8R2M2 | 99480 | DNTTIP2 | 0.641823074 | 0.000931365 |
| O88398 | 11567 | AVIL | 0.641593554 | 1.87152E-05 |
| P00375 | 13361 | DHFR | 0.641095602 | 0.000406652 |
| Q922D4 | 52036 | PPP6R3 | 0.640865063 | 1.57385E-05 |
| E9QAZ2 | 100039748 | Gm10020 | 0.640668334 | 0.001036657 |
| P97351 | 20091 | Rps3a1 | 0.64044175 | 4.74494E-05 |
| Z4YKM2 | 217149 | CISD3 | 0.640372655 | 0.000552204 |
| G3UYI5 |  |  | 0.640126841 | 0.001902967 |
| Q7TNE3 | 216873 | SPAG7 | 0.640061457 | 0.002319817 |
| E9Q585 | 20534 | SLC4A1AP | 0.639872366 | 0.00306288 |
| P10711 | 21399 | TCEA1 | 0.639824803 | 0.000445449 |
| Q9CSS1 | 72193 | SCAF11 | 0.639786093 | 0.001206954 |
| Q8CAC4 | 12572 | CDK7 | 0.639728275 | 0.000334097 |
| Q99J93 | 80876 | IFITM2 | 0.639613684 | 0.003228566 |
| Q62159 | 11853 | RHOC | 0.639573354 | 0.001023838 |
| F8WHV1 | 68523 | FAM96B | 0.639545669 | 0.000315206 |
| Q5SWD9 | 104662 | TSR1 | 0.63917196 | 1.13961E-05 |
| H3BJU7 | 16800 | ARHGEF2 | 0.638896537 | 0.002973113 |
| D3Z5I1 | 78781 | ZC3HAV1 | 0.638828772 | 3.27936E-05 |
| Q3TIJ4 | 66368 | RTCA | 0.638801683 | 0.002128338 |
| P41105 | 19943 | RPL28 | 0.638683199 | 0.00070258 |
| G3UWX1 | 19687 | RFC1 | 0.638643521 | 0.007238084 |
| Q9WVA3 | 12237 | BUB3 | 0.638455833 | 0.000793133 |
| Q9DBE9 | 56095 | FTSJ3 | 0.637734372 | 0.00012337 |
| Q8BY71 | 107435 | HAT1 | 0.637644301 | 2.22675E-05 |
| P56812 | 56330 | PDCD5 | 0.637483652 | 0.000152173 |
| Q9DBL7 | 71743 | COASY | 0.637122763 | 0.002060964 |
| Q9JJ94 | 68475 | SSNA1 | 0.636773438 | 0.00451842 |
| Q64261 | 12571 | CDK6 | 0.636408275 | 0.002162265 |
| Q9QYR9 | 171210 | ACOT2 | 0.636250865 | 1.31404E-05 |
| G3UWG1 |  |  | 0.636154173 | 4.23807E-06 |
| Q6A099 | 107338 | GBF1 | 0.635959075 | 0.000647802 |
| Q9CQ75 | 17991 | NDUFA2 | 0.635809211 | 0.000112105 |
| Q99JB8 | 80708 | PACSIN3 | 0.635742904 | 0.002374865 |
| E9PYJ6 | 230393 | FOCAD | 0.635205582 | 0.000115182 |
| I3PQW3 | 28036 | LARP7 | 0.634967239 | 0.002170608 |
| Q62383 | 20926 | Supt6 | 0.634957304 | 0.000678154 |
| P10852 | 17254 | SLC3A2 | 0.634482217 | 0.000383079 |
| Q3TI79 | 245474 | DKC1 | 0.634227144 | 0.001930541 |
| Q3UT19 | 70767 | PRPF3 | 0.633952743 | 0.000955171 |
| Q8VC28 | 27384 | Akr1c13 | 0.633861095 | 3.56924E-05 |
| Q8BR63 | 73385 | Fam177a | 0.633853603 | 3.86486E-05 |
| Q3TG58 | 22384 | EIF4H | 0.633639608 | 0.000599608 |
| Q9QZD9 | 54709 | EIF3I | 0.633134776 | 1.25969E-06 |
| Q61656 | 13207 | DDX5 | 0.633120646 | 0.000199648 |
| O88874 | 12454 | CCNK | 0.633065909 | 0.007050961 |
| Q3U468 | 12505 | CD44 | 0.632968545 | 0.000107345 |
| Q3UPG1 | 15288 | HMBS | 0.632700743 | 0.006145548 |
| P70388 | 19360 | RAD50 | 0.632492457 | 2.71123E-05 |
| Q3TMP1 | 98488 | GTF3C3 | 0.632248061 | 0.009286313 |
| P28271 | 11428 | ACO1 | 0.63212796 | 0.000205517 |
| Q04750 | 21969 | TOP1 | 0.632088991 | 0.000156992 |
| B2RXU2 | 66580 | ESF1 | 0.632019255 | 0.001509067 |
| Q9D9V3 | 52665 | ECHDC1 | 0.631649128 | 0.00249465 |
| B7ZWM8 | 70144 | LRCH3 | 0.63150979 | 0.002570989 |
| Q3TN42 | 105372 | UTP15 | 0.630866629 | 0.000568432 |
| Q6GU23 | 20848 | STAT3 | 0.630624157 | 0.000113511 |
| Q0VGU9 | 170791 | RBM39 | 0.630611138 | 0.001041909 |
| Q99ME9 | 69237 | GTPBP4 | 0.63039101 | 1.95048E-05 |
| Q9WUP7 | 56207 | UCHL5 | 0.630343999 | 0.001034395 |
| P62889 | 19946 | RPL30 | 0.630222985 | 2.38571E-07 |
| A0A087WSG5 | 17420 | MNAT1 | 0.630134252 | 0.001950485 |
| Q9D6J6 | 72900 | NDUFV2 | 0.629853199 | 1.50565E-05 |
| E9PYA3 | 14651 | HAGH | 0.628908256 | 0.000455084 |
| E0CXN5 | 14555 | GPD1 | 0.628673959 | 3.29248E-05 |
| Q9D892 | 16434 | ITPA | 0.628111482 | 0.000742892 |
| Q8K298 | 68743 | ANLN | 0.627867279 | 0.000969408 |
| Q3TV93 | 56150 | MAD2L1 | 0.627267509 | 1.93581E-05 |
| Q7TQK1 | 77065 | INTS7 | 0.626984446 | 0.000715545 |
| Q3UVI9 | 27407 | ABCF2 | 0.62674786 | 0.000191261 |
| P62307 | 69878 | SNRPF | 0.626561511 | 0.003692586 |
| Q8BP47 | 70223 | NARS | 0.626366474 | 7.6092E-06 |
| A0A068BIT8 | 16913 | PSMB8 | 0.626088701 | 0.001990439 |
| O54833 | 13000 | CSNK2A2 | 0.625871391 | 0.001436897 |
| Q8BWZ3 | 231713 | NAA25 | 0.625650615 | 1.0506E-05 |
| Q9D1E6 | 66411 | TBCB | 0.625633629 | 0.000262272 |
| Q9CR25 | 67728 | DPH2 | 0.624695177 | 0.000128616 |
| Q7TSC1 | 53761 | PRRC2A | 0.624460848 | 0.003693607 |
| Q3U6U7 | 22375 | WARS | 0.624342464 | 1.59048E-05 |
| Q9JI13 | 65961 | UTP3 | 0.624175148 | 0.000132627 |
| Q6NV83 | 67958 | U2SURP | 0.624132339 | 3.56915E-05 |
| Q7TPY3 | 18194 | NSDHL | 0.624107581 | 0.002052733 |
| H3BK68 | 212528 | TRMT1 | 0.624102469 | 0.000588527 |
| Q3U6Y9 | 12491 | CD36 | 0.623513583 | 0.001581697 |
| Q9CQB4 | 67530 | Uqcrb | 0.623437857 | 0.00027954 |
| Q8BFY9 | 238799 | TNPO1 | 0.623434002 | 4.71465E-05 |
| Q9Z130 | 50926 | HNRNPDL | 0.623249415 | 0.00034905 |
| Q9DC42 | 26364 | ADGRE5 | 0.621841575 | 0.000455963 |
| Q4VA28 | 19933 | RPL21 | 0.62141564 | 0.000168088 |
| Q9D2R0 | 78894 | AACS | 0.621041858 | 1.15864E-05 |
| R4H4V1 | 78891 | SCYL1 | 0.62080685 | 0.004478593 |
| P16125 | 16832 | LDHB | 0.620695391 | 8.1249E-05 |
| P08074 | 12409 | Cbr2 | 0.620618577 | 1.88607E-05 |
| Q3TMB5 | 22051 | TRIP6 | 0.619677212 | 0.000123442 |
| A2A9X5 | 50773 | NT5C | 0.61956022 | 0.005207465 |
| Q3TCE7 | 11867 | ARPC1B | 0.618891676 | 0.001131496 |
| D3Z795 | 56088 | PSMG1 | 0.618644415 | 0.006570547 |
| Q9D0L7 | 67211 | ARMC10 | 0.617700611 | 0.003783483 |
| Q3UMQ8 | 234344 | NAF1 | 0.617063923 | 0.00011767 |
| Q9JJX7 | 56196 | TDP2 | 0.616844182 | 0.000223337 |
| Q78RK2 | 170460 | STARD5 | 0.61671395 | 0.001208372 |
| Q7TMF3 | 66414 | NDUFA12 | 0.616580865 | 0.003401282 |
| Q99LJ6 | 67305 | GPX7 | 0.616264238 | 0.001204551 |
| Q99NB8 | 94232 | UBQLN4 | 0.615889733 | 2.01928E-05 |
| Q3UX26 | 68083 | PAK1IP1 | 0.615417607 | 3.78668E-05 |
| O54825 | 53414 | BYSL | 0.615222356 | 0.00023321 |
| Q8C2T9 | 24010 | IK | 0.615114135 | 0.007211138 |
| D3Z0G0 | 65102 | NIF3L1 | 0.6150448 | 0.000215462 |
| Q3TZK4 | 76614 | IMMT | 0.614926406 | 0.000339494 |
| Q80ZW2 | 223626 | THEM6 | 0.614826857 | 0.007353012 |
| Q9ET22 | 83768 | DPP7 | 0.61452354 | 0.001551967 |
| A0A0A6YWG8 | 56456 | ACTL6A | 0.613820971 | 0.009122738 |
| Q5SUF2 | 67684 | LUC7L3 | 0.613770667 | 0.000111995 |
| Q6ZWN5 | 76846 | RPS9 | 0.61374636 | 1.41496E-05 |
| Q61136 | 19134 | PRPF4B | 0.613540323 | 0.00014129 |
| Q8R5H1 | 14479 | USP15 | 0.613094679 | 0.008663537 |
| Q8CH72 | 69807 | TRIM32 | 0.612794255 | 0.003583379 |
| P18155 | 17768 | mthfd2 | 0.6123806 | 0.000184152 |
| Q91WG4 | 58523 | ELP2 | 0.612288134 | 0.001192876 |
| P19096 | 14104 | FASN | 0.612058373 | 2.04073E-06 |
| Q5SWU9 | 107476 | ACACA | 0.611860182 | 8.23893E-05 |
| Q99P31 | 66245 | HSPBP1 | 0.611518551 | 0.000139768 |
| Q4KMV6 | 72238 | TBC1D5 | 0.611029758 | 0.003770341 |
| Q3TDE6 | 74551 | PCK2 | 0.610413039 | 0.000614032 |
| Q0P6B2 | 51886 | FUBP1 | 0.610331255 | 3.21259E-05 |
| E0CX20 | 231889 | BUD31 | 0.609825928 | 0.00280153 |
| P59325 | 217869 | EIF5 | 0.609789545 | 7.7031E-05 |
| Q1W5W7 |  |  | 0.609359087 | 0.001108594 |
| P07607 | 22171 | TYMS | 0.609050479 | 6.92765E-05 |
| P52293 | 16647 | KPNA2 | 0.608523058 | 0.000259206 |
| E9PWQ3 | 12835 | COL6A3 | 0.608510975 | 0.000141881 |
| F7A1B4 | 13805 | ENG | 0.608310314 | 0.000725335 |
| Q3TC83 | 217011 | NLE1 | 0.60808956 | 0.000561482 |
| Q8BKT8 | 73738 | HAUS7 | 0.60800983 | 0.001537158 |
| F8WJG3 | 20462 | TRA2B | 0.607955676 | 0.001419369 |
| Q99LS3 | 100678 | PSPH | 0.607625165 | 0.000667424 |
| B1ARA5 | 19941 | RPL26 | 0.607501119 | 0.000339779 |
| Q9CPT5 | 28126 | NOP16 | 0.606637306 | 0.000178593 |
| Q6PJN8 | 67789 | DALRD3 | 0.606296408 | 0.009301006 |
| Q62425 | 17992 | NDUFA4 | 0.60540551 | 0.000373747 |
| S4R1E5 | 625249 | GPX4 | 0.605233351 | 0.000104619 |
| P97390 | 22365 | VPS45 | 0.605121024 | 0.002258032 |
| O08663 | 56307 | METAP2 | 0.604235843 | 7.01055E-05 |
| E9Q555 | 672511 | RNF213 | 0.602796021 | 1.76392E-05 |
| P52432 | 20016 | POLR1C | 0.602681871 | 0.000117272 |
| Q66L45 | 227695 | SPOUT1 | 0.602681507 | 0.000406935 |
| Q8VED9 | 216551 | LGALSL | 0.602131317 | 0.000125694 |
| Q3U6F1 | 19664 | RBPJ | 0.601444042 | 8.59462E-05 |
| Q3U5V2 | 12785 | CNBP | 0.601431066 | 0.003619915 |
| Q8R3C6 | 74111 | RBM19 | 0.600786966 | 0.000303774 |
| Q3U057 | 54451 | CPSF3 | 0.600138844 | 5.34431E-05 |
| A2A513 | 16661 | KRT10 | 0.599273844 | 0.002407901 |
| Q8BLN5 | 16987 | LSS | 0.598939699 | 0.000230102 |
| Q9CQ80 | 28084 | VPS25 | 0.597254712 | 0.000781135 |
| Q9CWZ5 | 56200 | DDX21 | 0.596138382 | 5.8157E-05 |
| E9PZK7 | 76688 | ARFRP1 | 0.59566838 | 0.003241118 |
| O35075 | 13185 | DSCR3 | 0.595539295 | 0.001935491 |
| E9PW15 | 52615 | SUZ12 | 0.592855366 | 0.002645225 |
| Q8VHZ7 | 27993 | IMP4 | 0.591358542 | 0.000456096 |
| Q9DBY8 | 67459 | NVL | 0.590848346 | 0.007884186 |
| Q9D8M4 | 66229 | RPL7L1 | 0.590191438 | 1.93593E-05 |
| Q9R062 | 27357 | Gyg | 0.589766089 | 0.001117799 |
| Q3TFF0 | 56445 | DNAJA2 | 0.588062549 | 0.001455727 |
| Q6NZM8 | 23971 | PAPSS1 | 0.584806765 | 1.49567E-06 |
| O08915 | 11632 | AIP | 0.584319426 | 0.000534011 |
| Q569X8 | 103468 | NUP107 | 0.583156281 | 0.000792148 |
| Q3TV20 | 27053 | ASNS | 0.581864937 | 1.20053E-06 |
| Q8CCX9 | 27393 | MRPL39 | 0.580707489 | 0.007154658 |
| Q3V300 | 110033 | KIF22 | 0.579719089 | 0.002771746 |
| O08759 | 22215 | UBE3A | 0.579225762 | 0.001385648 |
| Q80UZ2 | 231452 | SDAD1 | 0.578810671 | 0.004318637 |
| Q9JMD0 | 22680 | Zfp207 | 0.578381451 | 0.000157437 |
| D3YTQ9 | 20054 | RPS15 | 0.577464052 | 0.001548603 |
| A2ATP5 | 17876 | MYEF2 | 0.576308496 | 0.001254153 |
| Q3TEN9 | 14933 | gk | 0.575495072 | 0.00850134 |
| Q3US29 | 110196 | FDPS | 0.574454742 | 2.55348E-05 |
| Q9JKB3 | 56449 | YBX3 | 0.57340069 | 0.000124161 |
| Q9CZX0 | 74195 | ELP3 | 0.569548439 | 0.002066556 |
| Q62241 | 20630 | SNRPC | 0.568069263 | 0.000487107 |
| A0A0A6YXY1 | 66617 | NTMT1 | 0.567045905 | 0.000916752 |
| O35654 | 18972 | POLD2 | 0.565665452 | 0.002334762 |
| Q3U8R9 | 53382 | TXNL1 | 0.561973819 | 0.000512018 |
| A0A0A6YW28 | 22258 | USP4 | 0.56135714 | 0.000480429 |
| J3QK23 |  |  | 0.560398269 | 0.000101732 |
| D3YXP6 | 68603 | pmvk | 0.560019742 | 1.60273E-05 |
| Q9CX60 | 77889 | LBH | 0.55251072 | 0.000237952 |
| P07141 | 12977 | CSF1 | 0.550636763 | 0.001290243 |
| G3X9T8 | 12870 | CP | 0.536260855 | 0.002481256 |
| Q3THZ8 | 54342 | GNPNAT1 | 0.534470574 | 4.37475E-06 |
| Q8VCN5 | 107869 | CTH | 0.528430404 | 7.33126E-05 |
| Q5SSP3 | 55963 | SLC1A4 | 0.526175482 | 8.69306E-06 |
| P04104 | 16678 | KRT1 | 0.521981153 | 0.001206141 |
| Q3USG5 | 100952 | EMILIN1 | 0.501547291 | 0.001262691 |
| Q8VE10 | 70999 | NAA40 | 0.500478749 | 0.000545633 |
| Q3UFR4 | 20514 | slc1a5 | 0.49696861 | 0.003086043 |
| P09528 | 14319 | FTH1 | 0.490524622 | 6.51954E-07 |
| Q8BGZ7 | 109052 | KRT75 | 0.468026368 | 0.000245707 |
| Q3THE6 |  |  | 0.4616293 | 0.002693626 |
| Q8K0C4 | 13121 | CYP51 | 0.459850202 | 6.11623E-05 |
| Q64735 | 12946 | CR1L | 0.449113402 | 0.002257693 |

**Supplementary Tables S4:** The differential metabolites in L929 cells after treatment with 100 μM Ni^2+^ for 12h.

| **Name** | **ttest** | **fold** | **VIP** | **mz** | **(min)** |
| --- | --- | --- | --- | --- | --- |
| CMPF | 0.00 | 8.12 | 1.303 | 240.00 | 9.32 |
| 6-Deoxy-L-galactose | 0.00 | 7.41 | 1.300 | 164.00 | 9.32 |
| cAMP | 0.00 | 5.21 | 1.301 | 329.00 | 0.76 |
| ADP-glucose | 0.00 | 5.13 | 1.175 | 589.00 | 0.99 |
| Glycocholic Acid | 0.01 | 3.21 | 1.384 | 465.00 | 15.07 |
| D-erythro-1-(Imidazol-4-yl)glycerol 3-phosphate | 0.00 | 2.68 | 1.211 | 238.00 | 6.67 |
| L-Tyrosine | 0.01 | 2.36 | 1.121 | 181.00 | 6.66 |
| Phytic acid | 0.04 | 2.35 | 1.320 | 660.00 | 0.61 |
| Nicotinic acid adenine dinucleotide | 0.03 | 2.31 | 1.320 | 664.00 | 0.98 |
| Phosphocreatinine | 0.01 | 2.11 | 1.206 | 193.00 | 0.97 |
| Folic acid | 0.00 | 1.69 | 1.422 | 441.00 | 4.44 |
| Palmitoyl-L-carnitine | 0.01 | 1.42 | 1.233 | 399 | 13.1 |
| FAD | 0.00 | -0.25 | 1.493 | 785.00 | 4.47 |
| 3,4,5-Trimethoxyphenyl acetate | 0.01 | -0.62 | 1.122 | 226.00 | 3.76 |
| Prolyl-Threonine | 0.00 | -0.65 | 1.061 | 216.00 | 2.42 |
| 2-Oxo-4-methylthiobutanoic acid | 0.02 | -0.69 | 1.027 | 148.00 | 14.25 |
| N-(2,5-Dihydroxyphenyl)pyridinium(1+) | 0.00 | -0.70 | 1.153 | 188.00 | 3.76 |
| N-Formyl-4-amino-5-aminomethyl-2-methylpyrimidine | 0.01 | -0.72 | 1.245 | 166.00 | 1.96 |
| o-Methyldo-Methyldopamine (3-methoxytyramine) | 0.00 | -0.73 | 1.220 | 167.00 | 1.96 |
| Acetylcholine chloride | 0.01 | -0.74 | 1.190 | 181.00 | 1.03 |
| D-Mannitol | 0.00 | -0.77 | 1.172 | 182.00 | 1.03 |
| Niacinamide | 0.00 | -0.78 | 1.256 | 122.00 | 1.01 |
| Histidine | 0.00 | -1.04 | 1.232 | 155.00 | 0.66 |
| L-Cystathionine | 0.00 | -1.10 | 1.822 | 222.00 | 10.01 |
| Uridine 5'-diphosphoglucuronic acid | 0.00 | -1.25 | 1.541 | 580.00 | 1.18 |
| threo-3-Hydroxy-L-aspartate | 0.00 | -1.25 | 1.459 | 149.00 | 0.96 |
| GDP-glucose | 0.04 | -1.26 | 1.020 | 605.00 | 0.83 |
| Acetylcarnitine | 0.00 | -1.27 | 1.062 | 203.00 | 0.73 |
| Spermidine | 0.00 | -1.29 | 1.208 | 145.00 | 0.58 |
| L-Cys-Gly | 0.00 | -1.35 | 1.284 | 178.00 | 0.95 |
| 4-Phosphopantothenoylcysteine | 0.00 | -1.44 | 1.144 | 403.00 | 9.05 |
| Pyridoxine (Vitamin B6) | 0.00 | -1.47 | 1.164 | 169.00 | 0.87 |
| Inodxyl glucuronide | 0.02 | -1.65 | 1.356 | 309 | 0.95 |
| Oleamide | 0.00 | -1.66 | 1.109 | 281.00 | 11.78 |
| DL-Ornithino-L-alanine | 0.01 | -1.68 | 1.168 | 219.00 | 2.43 |
| Glutathione | 0.00 | -1.69 | 1.288 | 307.00 | 0.97 |
| 2-Oxo-4E-hexenoic acid | 0.02 | -2.00 | 1.438 | 128.00 | 0.69 |
| 1D-Myo-inositol 1,3-bisphosphate | 0.01 | -2.51 | 1.367 | 340.00 | 0.89 |
| Biotinyl-CoA | 0.01 | -2.68 | 1.352 | 993.00 | 0.97 |
| N-Acetyl-L-methionine | 0.01 | -3.15 | 1.272 | 191.00 | 1.16 |

**Supplementary Tables S5:** The differential metabolites in L929 cells after treatment with 100 μM Ni^2+^ for 24h.

| **Name** | **ttest** | **fold** | **VIP** | **mz** | **T(min)** |
| --- | --- | --- | --- | --- | --- |
| L-Ascorbic acid | 0.02 | 1.16 | 1.737 | 175.02 | 1.2 |
| 5'-CMP | 0.00 | 1.03 | 2.568 | 322.05 | 7.61 |
| dTMP | 0.00 | 1.01 | 2.481 | 321.04 | 7.61 |
| Hippuric acid | 0.02 | 0.86 | 1.762 | 178.05 | 6.59 |
| MG(24:6) | 0.03 | 0.72 | 1.600 | 431.66 | 13.53 |
| fumarylacetic acid | 0.02 | 0.66 | 1.751 | 157.01 | 1.18 |
| PC(O-13:0)/PE(O-16:0) | 0.003 | 0.64 | 2.085 | 438.30 | 13.56 |
| Pantothenic Acid | 0.011 | 0.60 | 1.786 | 220.01 | 2.62 |
| PI(40:3) | 0.029 | 0.579 | 1.3617 | 915.5831 | 13.62 |
| PE(P-16:0) | 0.002 | 0.569 | 2.13 | 436.284 | 13.63 |
| Linoleoyl Ethanolamide | 0.01 | 0.56 | 1.957 | 324.35 | 14.58 |
| DG(36:1) | 0.03 | 0.53 | 1.643 | 621.55 | 0.69 |
| Riboflavin (Vitamin B2) | 0.05 | 0.48 | 1.468 | 377.37 | 5.3 |
| Indan-1-ol | 0.01 | 0.48 | 1.882 | 135.02 | 11.79 |
| PG(33:0) | 0.017 | 0.431 | 1.75 | 735.5191 | 0.7 |
| 2-Hydroxyethylphosphonate | 0.01 | 0.41 | 1.844 | 125.00 | 1.05 |
| Riboflavin | 0.039 | 0.393 | 1.54 | 375.1318 | 5.3 |
| Homovanillic acid | 0.02 | 0.39 | 1.685 | 181.05 | 4 |
| 2-amino-hexadecanoic acid | 0.01 | 0.37 | 1.936 | 272.26 | 9.99 |
| dUMP | 0.03 | 0.36 | 1.718 | 309.33 | 1.07 |
| Furoic acid | 0.019 | 0.34 | 1.675 | 113.14 | 1.06 |
| TG(52:1) | 0.044 | 0.273 | 1.508 | 859.7949 | 0.63 |
| 2-Hydroxyisovaleric acid | 0.02 | 0.25 | 1.742 | 117.06 | 2.01 |
| L-Tyrosine | 0.03 | 0.24 | 1.555 | 182.46 | 1.09 |
| D-Sorbitol | 0.04 | 0.24 | 1.481 | 183.268 | 1.09 |
| PE(36:6) | 0.012 | 0.226 | 1.819 | 734.4687 | 0.7 |
| Phenylpyruvic acid | 0.04 | 0.22 | 1.508 | 165.36 | 1.09 |
| PE(31:0)/ PC(28:0) | 0.03 | 0.22 | 1.654 | 676.51 | 0.71 |
| PE(32:0) | 0.031 | 0.188 | 1.596 | 674.5173 | 0.71 |
| Sphingosine | 0.01 | 0.18 | 1.774 | 300.01 | 13.51 |
| Valine | 0.04 | 0.09 | 1.529 | 118.30 | 0.72 |
| Fructose/D-Glucose/D-Galactose | 0.05 | -0.17 | 1.493 | 179.06 | 0.68 |
| Caffeic acid sulfate | 0.02 | -0.19 | 1.724 | 258.98 | 0.73 |
| Oxoglutaric acid | 0.04 | -0.21 | 1.513 | 145.01 | 1.05 |
| L-Glutamate | 0.02 | -0.25 | 1.750 | 146.05 | 0.72 |
| L-Leucine | 0.01 | -0.27 | 1.954 | 130.09 | 1.22 |
| Homocysteinesulfinic acid | 0.03 | -0.28 | 1.650 | 166.02 | 0.76 |
| Citraconic acid | 0.00 | -0.29 | 2.014 | 129.02 | 0.75 |
| PC(17:1)/PE(20:1) | 0.02 | -0.30 | 1.684 | 506.33 | 13.69 |
| L-Histidine | 0.02 | -0.32 | 1.781 | 154.06 | 0.67 |
| L-Arginine | 0.02 | -0.33 | 1.630 | 175.63 | 0.69 |
| Indole | 0.01 | -0.34 | 1.959 | 116.05 | 3.82 |
| Uric acid | 0.016 | -0.366 | 1.757 | 167.021 | 1.05 |
| 2-Formylaminobenzaldehyde | 0.05 | -0.37 | 1.498 | 148.04 | 1.06 |
| 2-Aminoethylphosphonic acid | 0.02 | -0.37 | 1.771 | 124.01 | 1.05 |
| PI(19:0) | 0.014 | -0.4 | 1.787 | 613.335 | 12.83 |
| LysoPE(22:5) | 0.01 | -0.44 | 1.842 | 528.25 | 13.47 |
| 3-Dehydroquinic acid | 0.02 | -0.47 | 1.716 | 189.04 | 1.15 |
| Isocitrate | 0.00 | -0.53 | 2.343 | 191.02 | 0.75 |
| N-Acetyl-D-phenylalanine | 0.01 | -0.54 | 1.868 | 206.08 | 6.16 |
| Nonanedioic acid | 0.00 | -0.56 | 2.029 | 187.10 | 6.97 |
| PC(22:6) | 0.01 | -0.59 | 1.934 | 568.37 | 12.83 |
| LysoPC(22:5) | 0.00 | -0.60 | 1.964 | 570.00 | 13.54 |
| Taurine | 0.002 | -0.612 | 2.167 | 124.0108 | 0.73 |
| Phosphocreatinine | 0.033 | -0.619 | 1.585 | 192.023 | 1.06 |
| Proline betaine | 0.00 | -0.63 | 2.019 | 144.78 | 0.74 |
| Citric acid | 0.02 | -0.63 | 1.668 | 191.0195 | 1.06 |
| Se-Methylselenocysteine | 0.001 | -0.72 | 2.195 | 181.9713 | 0.63 |
| N-Acetylleucine | 0.01 | -0.82 | 1.900 | 172.10 | 5.7 |
| Gluconic acid/Galactonic acid | 0.00 | -0.90 | 2.336 | 195.05 | 0.72 |

**Supplementary Tables S6:** The differential metabolites in L929 cells after treatment with 100 μM Ni^2+^ for 48h.

| **Name** | **ttest** | **fold** | **VIP** | **mz** | **T(min)** |
| --- | --- | --- | --- | --- | --- |
| 2-Hydroxybutyric acid | 0.00 | 1.32 | 1.949 | 103.04 | 1.24 |
| Retinyl ester | 0 | 1.041 | 1.874 | 301.2171 | 15.74 |
| Hydroxyhydroquinone | 0.00 | 0.78 | 2.016 | 127.04 | 1.24 |
| Citric acid | 0.03 | 0.77 | 1.195 | 191.02 | 1.06 |
| Uridine | 0 | 0.769 | 1.603 | 243.0613 | 1.06 |
| Phosphocreatinine | 0.04 | 0.767 | 1.137 | 192.023 | 1.06 |
| PC(20:4)/ | 0.01 | 0.73 | 1.500 | 544.34 | 12.84 |
| LysoPE(22:5) | 0.00 | 0.73 | 1.616 | 528.31 | 13.47 |
| Furoic acid | 0.03 | 0.67 | 1.247 | 111.01 | 1.06 |
| PC(14:0)/PE(17:0) | 0.00 | 0.66 | 1.757 | 468.31 | 11.86 |
| Isocitrate | 0.02 | 0.65 | 1.330 | 191.02 | 0.75 |
| 2,3-Pyridinedicarboxylic acid | 0.00 | 0.61 | 1.602 | 166.02 | 0.76 |
| PE(20:4) | 0.01 | 0.59 | 1.485 | 502.29 | 12.78 |
| Oxoglutaric acid | 0.00 | 0.57 | 1.709 | 145.01 | 1.05 |
| N-Acetylneuraminic Acid | 0.01 | 0.57 | 1.295 | 308.10 | 0.76 |
| PE(22:6) | 0.01 | 0.54 | 1.488 | 526.29 | 12.77 |
| Gluconic acid/Galactonic acid | 0.02 | 0.48 | 1.252 | 195.05 | 0.72 |
| TG(54:0) | 0.01 | 0.468 | 1.399 | 889.8092 | 0.63 |
| PC(22:6) | 0.04 | 0.46 | 1.271 | 568.34 | 12.83 |
| TG(57:5) | 0 | 0.458 | 1.643 | 921.7925 | 0.63 |
| TG(55:2) | 0.01 | 0.455 | 1.429 | 899.8201 | 0.63 |
| Pyroglutamic acid | 0.02 | 0.418 | 1.305 | 128.0351 | 0.77 |
| PC(16:1)/PE(19:1) | 0.02 | 0.35 | 1.314 | 494.32 | 12.3 |
| TG(55:3) | 0.02 | 0.338 | 1.219 | 897.8098 | 0.63 |
| Caffeic acid sulfate | 0.02 | 0.33 | 1.344 | 258.98 | 0.73 |
| TG(57:4) | 0.04 | 0.324 | 1.23 | 923.8104 | 0.62 |
| Acetoacetic acid | 0.02 | 0.31 | 1.433 | 101.02 | 0.75 |
| 5'-Phosphoribosyl-N-formylglycinamide (FGAR | 0.02 | 0.31 | 1.316 | 313.05 | 1.05 |
| Lipoxin C4 | 0.02 | 0.30 | 1.265 | 640.29 | 14.79 |
| PC(20:3) | 0.03 | 0.30 | 1.255 | 546.35 | 14.78 |
| Linoleic acid | 0.02 | 0.30 | 1.272 | 278.83 | 0.67 |
| L-Glutamate | 0.04 | 0.20 | 1.163 | 146.05 | 0.72 |
| Quinoline | 0.05 | 0.20 | 1.300 | 130.07 | 3.82 |
| PS(18:1) | 0.03 | 0.20 | 1.272 | 524.30 | 13.28 |
| PC(18:3) | 0.03 | 0.09 | 1.207 | 518.32 | 13.28 |
| PE(18:3) | 0.01 | -0.21 | 1.421 | 476.28 | 13.2 |
| PE(16:0)/PC(13:0) | 0.03 | -0.24 | 1.325 | 454.2935 | 13.2 |
| PS(18:2) | 0 | -0.288 | 1.523 | 520.2665 | 13.19 |
| PE(32:0) | 0.04 | -0.29 | 1.172 | 674.52 | 0.71 |
| L-Phenylalanine | 0.00 | -0.30 | 1.578 | 166.09 | 2 |
| PE(16:0)/ PC(13:0) | 0.00 | -0.32 | 1.503 | 452.28 | 12.92 |
| PE(36:6) | 0.03 | -0.32 | 1.257 | 734.47 | 0.7 |
| 2-Phenylacetamide | 0.01 | -0.33 | 1.430 | 136.08 | 1.09 |
| 2-Hydroxyvaleric acid | 0.01 | -0.33 | 1.369 | 117.06 | 2.01 |
| PC(28:0) | 0.03 | -0.33 | 1.225 | 676.51 | 0.71 |
| D-Sorbitol | 0.01 | -0.35 | 1.428 | 183.08 | 1.09 |
| L-Valine | 0.01 | -0.36 | 1.439 | 118.09 | 1.05 |
| L-Arginine | 0.01 | -0.36 | 1.455 | 175.12 | 0.69 |
| PE(P-34:4) | 0.02 | -0.36 | 1.314 | 694.49 | 0.71 |
| L-Leucine | 0.00 | -0.37 | 1.642 | 130.09 | 1.22 |
| Pantothenic Acid | 0.020 | -0.37 | 1.293 | 218.10 | 2.61 |
| L-Tyrosine | 0.01 | -0.38 | 1.534 | 182.08 | 1.09 |
| Carnitine | 0.03 | -0.40 | 1.306 | 162.11 | 0.71 |
| 2-amino-hexadecanoic acid | 0.00 | -0.41 | 1.856 | 272.26 | 9.99 |
| Taurochenodeoxycholate | 0.05 | -0.413 | 1.087 | 498.2928 | 10.34 |
| Indan-1-ol | 0.01 | -0.44 | 1.442 | 135.08 | 11.79 |
| PE(20:3) | 0.03 | -0.45 | 1.142 | 502.29 | 10.34 |
| PG(33:0) | 0.01 | -0.469 | 1.483 | 735.5191 | 0.7 |
| D-Ribose | 0.00 | -0.48 | 1.880 | 151.06 | 1.05 |
| Gentisic acid | 0.01 | -0.48 | 1.317 | 153.0189 | 5.65 |
| Propionyl-L-carnitine | 0.03 | -0.49 | 1.248 | 218.14 | 1.28 |
| PE(P-16:0) | 0.010 | -0.50 | 1.426 | 438.30 | 13.63 |
| PI(40:3) | 0.05 | -0.507 | 1.106 | 915.5831 | 13.62 |
| L-Methionine | 0.00 | -0.52 | 1.902 | 150.06 | 1.05 |
| Normetanephrine | 0.02 | -0.59 | 1.347 | 184.10 | 0.68 |
| Leukotriene D4 | 0.03 | -0.60 | 1.178 | 495.26 | 9.21 |
| N-Acetylleucine | 0.02 | -0.63 | 1.324 | 172.10 | 5.7 |
| PS(16:0/0) | 0 | -0.724 | 1.504 | 496.2746 | 9.76 |
| L-Proline | 0.00 | -0.76 | 2.042 | 116.07 | 0.73 |
| PC(2:0) | 0.00 | -0.76 | 2.002 | 298.10 | 0.68 |
| Anthranilic acid | 0.00 | -0.80 | 2.058 | 138.05 | 0.73 |
| N-Acetyl-D-phenylalanine | 0.00 | -0.80 | 1.626 | 206.08 | 6.16 |
| KAPA | 0.00 | -0.86 | 2.122 | 186.11 | 5.68 |
| Shikimate 3-phosphate | 0 | -1.27 | 1.48 | 253.0176 | 6.18 |

**Supplementary Tables S7:** Metabolic pathways in which the proteins and metabolites participated together in the Ni^2+^-12 h group.

| **No.** | **Pathway** | **Metabolite** | **Protein** |
| --- | --- | --- | --- |
| 1 | Amino sugar and nucleotide sugar metabolism | cpd:C00167,  cpd:C01019 | mmu:22122, mmu:54342 |
| 2 | Aminoacyl-tRNA biosynthesis | cpd:C00135,  cpd:C00082 | mmu:70223, mmu:104458, mmu:97541, mmu:353172, mmu:234734, mmu:22375, mmu:107271 |
| 3 | Arginine and proline metabolism | cpd:C00315 | mmu:11671, mmu:20810, mmu:56454, mmu:66988 |
| 4 | Ascorbate and aldarate metabolism | cpd:C00167 | mmu:11671 |
| 5 | beta-Alanine metabolism | cpd:C00135,  cpd:C00315 | mmu:11671 |
| 6 | Cysteine and methionine metabolism | cpd:C01180,  cpd:C02291 | mmu:20810, mmu:107869, mmu:12035, mmu:107272, mmu:236539 |
| 7 | Fatty acid degradation | cpd:C02990 | mmu:11671 |
| 8 | Fructose and mannose metabolism | cpd:C01019 | mmu:319801, mmu:22122 |
| 9 | Glutathione metabolism | cpd:C00051,  cpd:C00315 | mmu:66988, mmu:14381, mmu:20810, mmu:20135 |
| 10 | Glycerophospholipid metabolism | cpd:C01996 | mmu:64899, mmu:52858 |
| 11 | Glycine, serine and threonine metabolism | cpd:C02291 | mmu:236539, mmu:107869, mmu:107272, mmu:100678, mmu:13382 |
| 12 | Histidine metabolism | cpd:C00135 | mmu:11671 |
| 13 | Inositol phosphate metabolism | cpd:C04062,  cpd:C01204 | mmu:269437, mmu:52858 |
| 14 | One carbon pool by folate | cpd:C00504 | mmu:17768 |
| 15 | Pantothenate and CoA biosynthesis | cpd:C04352 | mmu:12035 |
| 16 | Phosphatidylinositol signaling system | cpd:C04062,  cpd:C01204 | mmu:52858 |
| 17 | Purine metabolism | cpd:C00575 | mmu:20135 |
| 18 | Tyrosine metabolism | cpd:C00082 | mmu:14085 |

**Supplementary Tables S8:** Metabolic pathways in which the proteins and metabolites participated together in the Ni^2+^-24 h group.

| **No.** | **Pathway** | **Metabolite** | **Protein** |
| --- | --- | --- | --- |
| 1 | Alanine, aspartate and glutamate metabolism | cpd:C00025, cpd:C00158, cpd:C00026 | mmu:52633, mmu:27053, mmu:14718, mmu:14719, mmu:11898, mmu:11565, mmu:11566, mmu:109900, mmu:11564, mmu:69719, mmu:14661, mmu:14660, mmu:14583, mmu:231327 |
| 2 | Aminoacyl-tRNA biosynthesis | cpd:C00135, cpd:C00062, cpd:C00183, cpd:C00123, cpd:C00082, cpd:C00025 | mmu:70223, mmu:15115, mmu:23874, mmu:66590, mmu:104458, mmu:97541, mmu:27267, mmu:353172, mmu:226414, mmu:226539, mmu:20226, mmu:216443, mmu:22321, mmu:234734, mmu:85305, mmu:105148, mmu:107045, mmu:110960, mmu:272396, mmu:71807, mmu:22375, mmu:107271, mmu:107508, mmu:67417 |
| 3 | Arginine and proline metabolism | cpd:C00062, cpd:C00025 | mmu:11669, mmu:11671, mmu:56752, mmu:20810, mmu:17161, mmu:66054, mmu:14718, mmu:14719, mmu:66194, mmu:69051, mmu:18451, mmu:56454, mmu:66988 |
| 4 | Arginine biosynthesis | cpd:C00025, cpd:C00062, cpd:C00026 | mmu:14661, mmu:109900, mmu:11898, mmu:14660, mmu:14718, mmu:14719 |
| 5 | beta-Alanine metabolism | cpd:C00135 | mmu:11409, mmu:93747, mmu:97212, mmu:104776, mmu:11669, mmu:11671, mmu:56752, mmu:66054 |
| 6 | Butanoate metabolism | cpd:C00025, cpd:C00026 | mmu:67041, mmu:78894, mmu:15356, mmu:208715, mmu:110446, mmu:15107, mmu:93747, mmu:97212, mmu:11409, mmu:217666 |
| 7 | Citrate cycle (TCA cycle) | cpd:C00026, cpd:C00311, cpd:C00158 | mmu:13382, mmu:18293, mmu:239017, mmu:78920, mmu:20916, mmu:20917, mmu:56451, mmu:15926, mmu:269951, mmu:15929, mmu:170718, mmu:67834, mmu:104112, mmu:66052, mmu:66925, mmu:66945, mmu:67680, mmu:14194, mmu:11428, mmu:11429, mmu:12974, mmu:17448, mmu:17449, mmu:18563, mmu:18597, mmu:68263, mmu:235339, mmu:74551 |
| 8 | D-Glutamine and D-glutamate metabolism | cpd:C00025, cpd:C00026 | mmu:217830, mmu:14661, mmu:14660 |
| 9 | Fructose and mannose metabolism | cpd:C00794 | mmu:11677, mmu:14187, mmu:20322, mmu:15275, mmu:15277, mmu:319801, mmu:54128, mmu:22122, mmu:218138, mmu:69080, mmu:18641, mmu:56421, mmu:21991, mmu:11674, mmu:234730 |
| 10 | Galactose metabolism | cpd:C00794 | mmu:18641, mmu:56421, mmu:14387, mmu:11677, mmu:14187, mmu:15275, mmu:15277, mmu:66681, mmu:72157, mmu:74246, mmu:216558, mmu:14635, mmu:319625 |
| 11 | Glutathione metabolism | cpd:C00025 | mmu:66988, mmu:14775, mmu:67305, mmu:69590, mmu:625249, mmu:66073, mmu:14381, mmu:15926, mmu:269951, mmu:14782, mmu:14629, mmu:14630, mmu:103140, mmu:14860, mmu:14862, mmu:14863, mmu:14870, mmu:14873, mmu:68312, mmu:110208, mmu:20810, mmu:20135 |
| 12 | Glycerolipid metabolism | cpd:C00422 | mmu:13350, mmu:64899, mmu:225010, mmu:28169, mmu:14933, mmu:11677, mmu:14187, mmu:11669, mmu:11671, mmu:56752, mmu:58810, mmu:16956 |
| 13 | Glycerophospholipid metabolism | cpd:C00350, cpd:C04230 | mmu:14792, mmu:13026, mmu:64899, mmu:225010, mmu:28169, mmu:52858, mmu:14555, mmu:333433, mmu:14571, mmu:320951, mmu:77582 |
| 14 | Glyoxylate and dicarboxylate metabolism | cpd:C00158, cpd:C00025, cpd:C00311 | mmu:11428, mmu:11429, mmu:76238, mmu:12974, mmu:17448, mmu:17449, mmu:67078, mmu:110446, mmu:66904, mmu:17850, mmu:108037, mmu:20425, mmu:13382, mmu:12359 |
| 15 | Histidine metabolism | cpd:C00025, cpd:C00135 | mmu:66054, mmu:11669, mmu:11671, mmu:56752, mmu:17161 |
| 16 | Nitrogen metabolism | cpd:C00025 | mmu:71934, mmu:14661 |
| 17 | Pantothenate and CoA biosynthesis | cpd:C00864, cpd:C00183 | mmu:71743, mmu:12035, mmu:12036 |
| 18 | Phenylalanine metabolism | cpd:C00166, cpd:C01586, cpd:C00082 | mmu:17161, mmu:14718, mmu:14719, mmu:17319 |
| 19 | Phenylalanine, tyrosine and tryptophan biosynthesis | cpd:C00166, cpd:C00082 | mmu:14718, mmu:14719 |
| 20 | Phosphonate and phosphinate metabolism | cpd:C03557 | mmu:13026 |
| 21 | Porphyrin and chlorophyll metabolism | cpd:C00025 | mmu:110006, mmu:12870, mmu:15369, mmu:109778, mmu:233016, mmu:12892, mmu:22275, mmu:15288, mmu:17025, mmu:107508, mmu:67417, mmu:15159, mmu:94284, mmu:14151 |
| 22 | Primary bile acid biosynthesis | cpd:C00245 | mmu:15488,  mmu:20280 |
| 23 | Purine metabolism | cpd:C00366 | mmu:20135, mmu:22436, mmu:110639, mmu:328099, mmu:231327, mmu:14450, mmu:108147, mmu:11564, mmu:67054, mmu:207728, mmu:11637, mmu:11639, mmu:11565, mmu:11566, mmu:109674, mmu:107569, mmu:50773, mmu:11534, mmu:11486, mmu:18950, mmu:23917, mmu:23918, mmu:229363, mmu:15452, mmu:229589, mmu:18582, mmu:23971, mmu:237823, mmu:66681, mmu:72157, mmu:53893, mmu:18746, mmu:56248 |
| 24 | Pyrimidine metabolism | cpd:C00055, cpd:C00365, cpd:C00364 | mmu:20135, mmu:69719, mmu:22247, mmu:66588, mmu:51797, mmu:107569, mmu:50773, mmu:22271, mmu:72269, mmu:18950 |
| 25 | Riboflavin metabolism | cpd:C00255 | mmu:233016 |
| 26 | Selenocompound metabolism | cpd:C05689 | mmu:50493, mmu:109079, mmu:50880, mmu:216443, mmu:107869, mmu:229905, mmu:23971 |
| 27 | Sphingolipid metabolism | cpd:C00319 | mmu:11886, mmu:13244, mmu:70750, mmu:14466 |
| 28 | Tyrosine metabolism | cpd:C00082, cpd:C05582 | mmu:17161, mmu:11532, mmu:14085, mmu:14718, mmu:14719, mmu:17319 |
| 29 | Valine, leucine and isoleucine biosynthesis | cpd:C00123, cpd:C00183 | mmu:12035, mmu:12036 |
| 30 | Valine, leucine and isoleucine degradation | cpd:C00183, cpd:C00123 | mmu:13382, mmu:110446, mmu:208715, mmu:67041, mmu:15356, mmu:11992, mmu:72039, mmu:93747, mmu:97212, mmu:56357, mmu:11364, mmu:12035, mmu:12036, mmu:113868, mmu:231086, mmu:52538, mmu:15107, mmu:58875, mmu:11409, mmu:17850, mmu:66904, mmu:11669, mmu:11671, mmu:56752, mmu:104776, mmu:15108, mmu:78894 |

**Supplementary Tables S9:** Metabolic pathways in which the proteins and metabolites participated together in the Ni^2+^-48 h group.

| **No.** | **Pathway** | **Metabolite** | **Protein** |
| --- | --- | --- | --- |
| 1 | Alanine, aspartate and glutamate metabolism | cpd:C00025, cpd:C00158, cpd:C00026 | mmu:52633, mmu:27053, mmu:14718, mmu:14719, mmu:11898, mmu:11566, mmu:109900, mmu:11564, mmu:69719, mmu:14661, mmu:14660, mmu:14583, mmu:231327 |
| 2 | alpha-Linolenic acid metabolism | cpd:C00157 | mmu:113868 |
| 3 | Aminoacyl-tRNA biosynthesis | cpd:C00079, cpd:C00062, cpd:C00073, cpd:C00183, cpd:C00123, cpd:C00082, cpd:C00148, cpd:C00025 | mmu:70223, mmu:15115, mmu:23874, mmu:66590, mmu:104458, mmu:97541, mmu:27267, mmu:353172, mmu:226414, mmu:226539, mmu:20226, mmu:216443, mmu:22321, mmu:224805, mmu:234734, mmu:85305, mmu:105148, mmu:381314, mmu:107045, mmu:110960, mmu:71807, mmu:22375, mmu:107271, mmu:107508, mmu:67417 |
| 4 | Arachidonic acid metabolism | cpd:C00157, cpd:C05951 | mmu:67305, mmu:19224, mmu:16993, mmu:56351, mmu:96979, mmu:109857, mmu:12408, mmu:12409 |
| 5 | Arginine and proline metabolism | cpd:C00062, cpd:C00148, cpd:C00025 | mmu:11669, mmu:11671, mmu:56752, mmu:20810, mmu:66054, mmu:14718, mmu:14719, mmu:66194, mmu:69051, mmu:56454, mmu:66988 |
| 6 | Arginine biosynthesis | cpd:C00025, cpd:C00062, cpd:C00026 | mmu:14661, mmu:109900, mmu:11898, mmu:14660, mmu:14718, mmu:14719 |
| 7 | Biosynthesis of unsaturated fatty acids | cpd:C01595 | mmu:171210, mmu:70025 |
| 8 | Butanoate metabolism | cpd:C00164, cpd:C00025, cpd:C00026 | mmu:67041, mmu:78894, mmu:110446, mmu:110460, mmu:93747, mmu:97212, mmu:11409, mmu:217666 |
| 9 | Citrate cycle (TCA cycle) | cpd:C00026, cpd:C00311, cpd:C00158 | mmu:13382, mmu:18293, mmu:78920, mmu:20916, mmu:20917, mmu:56451, mmu:15926, mmu:269951, mmu:15929, mmu:170718, mmu:67834, mmu:104112, mmu:66052, mmu:66925, mmu:66945, mmu:67680, mmu:14194, mmu:11428, mmu:11429, mmu:12974, mmu:17448, mmu:17449, mmu:18563, mmu:18597, mmu:68263, mmu:235339, mmu:74551 |
| 10 | Cysteine and methionine metabolism | cpd:C00073 | mmu:56369, mmu:20810, mmu:107869, mmu:229905, mmu:108645, mmu:232087, mmu:11615, mmu:229709, mmu:13433, mmu:67870, mmu:14718, mmu:14719, mmu:22117, mmu:246221, mmu:16832, mmu:17448, mmu:17449, mmu:14630, mmu:12035, mmu:12036, mmu:107272, mmu:236539 |
| 11 | D-Glutamine and D-glutamate metabolism | cpd:C00025, cpd:C00026 | mmu:217830, mmu:14661, mmu:14660 |
| 12 | Fructose and mannose metabolism | cpd:C00794 | mmu:11677, mmu:14187, mmu:20322, mmu:319801, mmu:54128, mmu:22122, mmu:218138, mmu:69080, mmu:18642, mmu:234730 |
| 13 | Galactose metabolism | cpd:C00794 | mmu:18642, mmu:14387, mmu:11677, mmu:14187, mmu:74246, mmu:216558, mmu:14635, mmu:319625 |
| 14 | Glutathione metabolism | cpd:C00025, cpd:C01879 | mmu:66988, mmu:67305, mmu:625249, mmu:66073, mmu:14381, mmu:15926, mmu:269951, mmu:14782, mmu:14630, mmu:14862, mmu:14863, mmu:14870, mmu:14873, mmu:110208, mmu:20810 |
| 15 | Glycerolipid metabolism | cpd:C00422 | mmu:64899, mmu:28169, mmu:14933, mmu:11677, mmu:14187, mmu:11669, mmu:11671, mmu:56752, mmu:58810, mmu:16956 |
| 16 | Glycerophospholipid metabolism | cpd:C00350, cpd:C00157 | mmu:14792, mmu:68671, mmu:64899, mmu:28169, mmu:52858, mmu:14555, mmu:333433, mmu:14571, mmu:320951, mmu:77582 |
| 17 | Glyoxylate and dicarboxylate metabolism | cpd:C00158, cpd:C00025, cpd:C00311 | mmu:11428, mmu:11429, mmu:76238, mmu:12974, mmu:17448, mmu:17449, mmu:67078, mmu:110446, mmu:110460, mmu:66904, mmu:17850, mmu:108037, mmu:20425, mmu:13382, mmu:12359 |
| 18 | Histidine metabolism | cpd:C00025 | mmu:66054, mmu:11669, mmu:11671, mmu:56752 |
| 19 | Nicotinate and nicotinamide metabolism | cpd:C03722 | mmu:59027, mmu:68646, mmu:18115, mmu:68346, mmu:107569, mmu:50773, mmu:76952 |
| 20 | Nitrogen metabolism | cpd:C00025 | mmu:14661 |
| 21 | Pantothenate and CoA biosynthesis | cpd:C00864, cpd:C00183 | mmu:71743, mmu:12035, mmu:12036 |
| 22 | Pentose phosphate pathway | cpd:C00121 | mmu:110639, mmu:328099, mmu:21881, mmu:66646, mmu:18642, mmu:110208, mmu:66171, mmu:14381, mmu:21351 |
| 23 | Phenylalanine metabolism | cpd:C00079, cpd:C00082 | mmu:14718, mmu:14719 |
| 24 | Phenylalanine, tyrosine and tryptophan biosynthesis | cpd:C00079, cpd:C00082 | mmu:14718, mmu:14719 |
| 25 | Porphyrin and chlorophyll metabolism | cpd:C00025 | mmu:110006, mmu:12870, mmu:109778, mmu:233016, mmu:12892, mmu:22275, mmu:15288, mmu:17025, mmu:107508, mmu:67417, mmu:15159, mmu:94284, mmu:14151 |
| 26 | Primary bile acid biosynthesis | cpd:C05465 | mmu:15488, mmu:20280 |
| 27 | Propanoate metabolism | cpd:C05984 | mmu:104776, mmu:66904, mmu:52665, mmu:17850, mmu:20916, mmu:20917, mmu:56451, mmu:11409, mmu:110446, mmu:110460, mmu:107476, mmu:16832, mmu:93747, mmu:97212, mmu:13382 |
| 28 | Pyrimidine metabolism | cpd:C00299 | mmu:69719, mmu:22247, mmu:18102, mmu:66588, mmu:51797, mmu:107569, mmu:50773, mmu:76952, mmu:22271, mmu:72269, mmu:22171 |
| 29 | Synthesis and degradation of ketone bodies | cpd:C00164 | mmu:110446, mmu:110460, mmu:67041 |
| 30 | Tryptophan metabolism | cpd:C00108 | mmu:110446, mmu:110460, mmu:93747, mmu:97212, mmu:13382, mmu:78920, mmu:12359, mmu:229905, mmu:11669, mmu:11671, mmu:56752 |
| 31 | Tyrosine metabolism | cpd:C05589, cpd:C00082, cpd:C00164, cpd:C00628 | mmu:12846, mmu:11532, mmu:14085, mmu:14718, mmu:14719 |
| 32 | Valine, leucine and isoleucine biosynthesis | cpd:C00123, cpd:C00183 | mmu:12035, mmu:12036 |
| 33 | Valine, leucine and isoleucine degradation | cpd:C00164, cpd:C00183, cpd:C00123 | mmu:13382, mmu:110446, mmu:110460, mmu:67041, mmu:93747, mmu:97212, mmu:56357, mmu:11364, mmu:12035, mmu:12036, mmu:113868, mmu:231086, mmu:52538, mmu:58875, mmu:11409, mmu:17850, mmu:66904, mmu:11669, mmu:11671, mmu:56752, mmu:104776, mmu:15108, mmu:78894 |

**Supplementary Tables S10:** Metabolic pathways in which the proteins and metabolites had upstream and downstream relationships in the Ni^2+^-12 h group.

| **No.** | **Pathway** | **Upstream Protein** | **Downstream metabolite** |
| --- | --- | --- | --- |
| 1 | Arginine and proline metabolism | SRM | C00315 |
| 2 | Cysteine and methionine metabolism | Cth | C02291 |
| 3 | Glutathione metabolism | SRM | C00051 |
|  |  | LAP3 | C00315 |
| 4 | Glycerophospholipid metabolism | Lpin3 | C01996 |
| 5 | Glycine, serine and threonine metabolism | Cth, Dld, Phgdh, Psat1, Psph | C02291 |
| 6 | Inositol phosphate metabolism | Plch1 | C01204 |
|  |  | Cdipt | C01204 |
| 7 | One carbon pool by folate | Mthfd2 | C00504 |
| 8 | Phosphatidylinositol signaling system | Cdipt | C04062 |
|  |  | Cdipt | C01204 |

**Supplementary Tables S11:** Metabolic pathways in which the proteins and metabolites had upstream and downstream relationships in the Ni^2+^-24 h group.

| **No.** | **Pathway** | **Upstream Protein** | **Downstream metabolite** |
| --- | --- | --- | --- |
| 1 | Arginine biosynthesis | Gls, Glud1, Got1, Got2, | C00025 |
|  |  | Asl, Ass1, Gls, Glud1, Got1, Got2 | C00062 |
| 2 | Alanine, aspartate and glutamate metabolism | Gls, Glud1, Nit2 | C00025 |
|  |  | Gls, Glud1, Nit2 | C00026 |
| 3 | Arginine and proline metabolism | Aldh18a1, Lap3, Pycrl, Pycr2 | C00025 |
| 4 | beta-Alanine metabolism | Aldh2, Aldh3a2, Aldh9a1, Cndp2 | C00135 |
| 5 | Citrate cycle (TCA cycle) | Acly, Aco1, Aco2, Cs, Dlat, Dld, Dlst, Fh1, Mdh1, Mdh2, Sdha, Sdhb, Sdhc, Sdhd, Sucla2, Suclg1, Suclg2, Idh1, Idh2, Idh3a, Idh3b, Idh3g, Ogdh, Pcx, Pdha1, Pdhb, Ogdhl | C00026 |
|  |  | Acly, Aco1, Aco2, Cs, Dlat, Dld, Dlst, Fh1, Mdh1, Mdh2, Sdha, Sdhb, Sdhc, Sdhd, Sucla2, Suclg1, Suclg2, Idh1, Idh2, Idh3a, Idh3b, Idh3g, Ogdh, Pcx, Pdha1, Pdhb, Ogdhl | C00311 |
|  |  | Acly, Aco1, Aco2, Cs, Dlat, Dld, Dlst, Fh1, Mdh1, Mdh2, Sdha, Sdhb, Sdhc, Sdhd, Sucla2, Suclg1, Suclg2, Idh1, Idh2, Idh3a, Idh3b, Idh3g, Ogdh, Pcx, Pdha1, Pdhb, Ogdhl | C00158 |
| 6 | D-Glutamine and D-glutamate metabolism | Gls, Glud1 | C00025 |
|  |  | Gls, Glud1 | C00026 |
| 7 | Fructose and mannose metabolism | Akr1b3, Akr1b8, Sord | C00794 |
| 8 | Galactose metabolism | Akr1b3, Akr1b8, Gale, Galk1, Galm, Hk1, Hk2, Pgm2, Pgm1, Ugp2, | C00794 |
| 9 | Glutathione metabolism | G6pdx, Gclc, Gclm, Gpx1, Gpx4, Gpx7, Gpx8, Gsr, Gstt3, Gsta4, Gstm1, Gstm2, Gstp1, Gsto1, Gstm7, Idh1, Idh2, Lap3, Pgd, Txndc12 | C00025 |
| 10 | Glycerolipid metabolism | Agpat3, Akr1b3, Akr1b8, Aldh2, Aldh3a2, Aldh9a1, Akr1a1, Dgat1, Gk, Lclat1, Lpin3, Lpl | C00422 |
| 11 | Glycerophospholipid metabolism | Agpat3, Gpd1, Gpd1l, Gpd2, Lpin3, Lpcat3, Pcyt1a, Pisd | C00350 |
|  |  | Gpd1, Gpd2, Gpd1l, Lpcat3, Lpin3, Pcyt1a, Pisd | C04230 |
| 12 | Glyoxylate and dicarboxylate metabolism | Aco1, Aco2, Cs, Mdh1, Mdh2 | C00158 |
|  |  | Aco1, Aco2, Cs, Mdh1, Mdh2 | C00311 |
| 13 | Histidine metabolism | Cndp2 | C00025 |
|  |  | Cndp2 | C00135 |
| 14 | Nitrogen metabolism | Glud1 | C00025 |
| 15 | Pantothenate and CoA biosynthesis | Bcat1, Bcat2 | C00183 |
| 16 | Phenylalanine, tyrosine and tryptophan biosynthesis | Got1, Got2 | C00166 |
|  |  | Got1, Got2 | C00082 |
| 17 | Phenylalanine metabolism | Got1, Got2, Mif | C00166 |
|  |  | Got1, Got2, Mif | C00082 |
| 18 | Pyrimidine metabolism | Cad, Cda, Cmpk1, Ctps, Nt5c, Nt5c3, Rrm2, Pnp, Upp1, Umps | C00055 |
|  |  | Cad, Cda, Cmpk1, Ctps, Nt5c, Nt5c3, Rrm2, Pnp, Upp1, Umps | C00365 |
|  |  | Cad, Cda, Cmpk1, Ctps, Nt5c, Nt5c3, Rrm2, Pnp, Upp1, Umps | C00364 |
| 19 | Purine metabolism | Ada, Adk, Adsl, Adssl1, Adss, Ak2, Ak3, Ak4, Ampd2, Atic, Gart, Gmps, Hprt, Impdh1, Impdh2, Nt5c3, Nt5c, Nudt5, Paics, Pde2a, Pde6d, Pfas, Pgm1, Pgm2, Ppat, Pnp, Prune1, Prps2, Prps1l3, Pkm, Rrm2, Xdh | C00366 |
| 20 | Sphingolipid metabolism | Asah1, Degs1, Gba, Kdsr | C00319 |
| 21 | Tyrosine metabolism | Got1, Got2, Mif | C00082 |
|  |  | Got1, Got2, Maoa, Mif | C05582 |
| 22 | Valine, leucine and isoleucine degradation | Bcat1, Bcat2 | C00183 |
|  |  | Bcat1, Bcat2 | C00123 |
| 23 | Valine, leucine and isoleucine biosynthesis | Bcat1, Bcat2 | C00123 |
|  |  | Bcat1, Bcat2 | C00183 |

**Supplementary Tables S12:** Metabolic pathways in which the proteins and metabolites had upstream and downstream relationships in the Ni^2+^-48 h group.

| **No.** | **Pathway** | **Upstream Protein** | **Downstream metabolite** |
| --- | --- | --- | --- |
| 1 | Nitrogen metabolism | Glud1 | C00025 |
| 2 | Biosynthesis of unsaturated fatty acids | Acot2, Acot7 | C01595 |
| 3 | Primary bile acid biosynthesis | Hsd17b4, Scp2 | C05465 |
| 4 | Phenylalanine, tyrosine and tryptophan biosynthesis | Got1, Got2 | C00079 |
|  |  | Got1, Got2 | C00082 |
| 5 | Phenylalanine metabolism | Got1, Got2 | C00079 |
|  |  | Got1, Got2 | C00082 |
| 6 | Arginine biosynthesis | Got1, Got2, Gls, Glud1 | C00025 |
|  |  | Asl, Ass1, Gls, Glud1, Got1, Got2 | C00062 |
| 7 | Alanine, aspartate and glutamate metabolism | Gls, Glud1, Nit2 | C00025 |
|  |  | Gls, Glud1, Nit2 | C00026 |
| 8 | Arginine and proline metabolism | Aldh18a1, Lap3, Pycrl, Pycr2 | C00025 |
|  |  | Lap3, Pycrl, Pycr2 | C00148 |
| 9 | Butanoate metabolism | Aacs, Acads, Acat1, Acat2, Echs1, Hadha, Oxct1 | C00164 |
| 10 | Valine, leucine and isoleucine biosynthesis | Bcat1, Bcat2 | C00123 |
|  |  | Bcat1, Bcat2 | C00183 |
| 11 | Valine, leucine and isoleucine degradation | Bcat1, Bcat2 | C00183 |
|  |  | Bcat1, Bcat2 | C00123 |
|  |  | Aacs, Acaa1a, Acaa2, Acadm, Acads, Acat1, Acat2, Bcat1, Bcat2, Dld, Echs1, Hadha, Hadhb, Hsd17b10, Ivd, Oxct1 | C00164 |
| 12 | Synthesis and degradation of ketone bodies | Acat1, Acat2, Oxct1 | C00164 |
| 13 | D-Glutamine and D-glutamate metabolism | Gls, Glud1 | C00025 |
|  |  | Gls, Glud1 | C00026 |
| 14 | Histidine metabolism | Cndp2 | C00025 |
| 15 | Pantothenate and CoA biosynthesis | Bcat1, Bcat2 | C00183 |
| 16 | Pentose phosphate pathway | G6pdx, Pfkm, Pgd, Pgls, Prps1l3, Prps2, Rpe, Taldo1, Tkt | C00121 |
| 17 | Citrate cycle (TCA cycle) | Aco1, Aco2, Cs, Acly, Dlat, Dld, Dlst, Fh1, Idh1, Idh2, Idh3g, Idh3a, Idh3b, Mdh1, Mdh2, Ogdh, Pcx, Pdha1, Pdhb, Sdha, Sdhb, Sdhc, Sdhd, Sucla2, Suclg2, Suclg1 | C00026 |
|  |  | Aco1, Aco2, Cs, Acly, Dlat, Dld, Dlst, Fh1, Idh1, Idh2, Idh3g, Idh3a, Idh3b, Mdh1, Mdh2, Ogdh, Pcx, Pdha1, Pdhb, Sdha, Sdhb, Sdhc, Sdhd, Sucla2, Suclg2, Suclg1 | C00311 |
|  |  | Aco1, Aco2, Cs, Acly, Dlat, Dld, Dlst, Fh1, Idh1, Idh2, Idh3g, Idh3a, Idh3b, Mdh1, Mdh2, Ogdh, Pcx, Pdha1, Pdhb, Sdha, Sdhb, Sdhc, Sdhd, Sucla2, Suclg2, Suclg1 | C00158 |
| 18 | Tyrosine metabolism | Comt, Got1, Got2 | C05589 |
|  |  | Got1, Got2 | C00082 |
|  |  | Fah, Got1, Got2 | C00164 |
| 19 | Pyrimidine metabolism | Cad, Cda, Cmpk1, Ctps, Nme1, Nt5c, Nt5c2, Nt5c3, Umps, Upp1 | C00299 |
| 20 | Fructose and mannose metabolism | Akr1b3, Akr1b8, Sord | C00794 |
| 21 | Galactose metabolism | Akr1b3, Akr1b8, Galk1, Gale, Galm, Ugp2 | C00794 |
| 22 | Glutathione metabolism | G6pdx, Gclm, Gpx4, Gpx7, Gsr, Gstm1, Gstm2, Gstp1, Gsto1, Idh1, Idh2, Lap3, Pgd, Txndc12 | C00025 |
|  |  | G6pdx, Gclm, Gpx4, Gpx7, Gsr, Idh1, Idh2, Lap3, Pgd, Txndc12 | C01879 |
| 23 | Glycerolipid metabolism | Agpat3, Akr1a1, Akr1b3, Akr1b8, Aldh2, Aldh3a2, Aldh9a1, Gk, Lpin3, Lpl | C00422 |
| 24 | Glycerophospholipid metabolism | Agpat3, Gpd1, Gpd1l, Gpd2, Lpcat3, Lpin3, Pcyt2, Pisd | C00350 |
|  |  | Agpat3, Gpd1, Gpd1l, Gpd2, Lpcat3, Lpin3, Pcyt2, Pisd | C00157 |
| 25 | Glyoxylate and dicarboxylate metabolism | Aco1, Aco2, Cs, Mdh1, Mdh2 | C00158 |
|  |  | Aco1, Aco2, Cs, Mdh1, Mdh2 | C00311 |
| 26 | Cysteine and methionine metabolism | Ahcyl1, Apip, Dnmt1, Enoph1, Gm4737, Kyat3, Mat2a, Mat2b, Srm | C00073 |
| 27 | Propanoate metabolism | Ldhb | C05984 |
